# Competing Michael/Anti-Michael Addition of Silyl Ketene Acetals to β‑Nitrostyrenes Incorporating an Electron-Withdrawing Group

**DOI:** 10.1021/acs.joc.5c01852

**Published:** 2025-09-30

**Authors:** Mayte A. Martínez-Aguirre, Diego A. Cruz-Aguilar, Eduardo Hernández-Huerta, Dylan López-Barba, Ricardo Ballinas-Indili, Saulo César Rosales-Amezcua, Cecilio Álvarez-Toledano, Marcos Hernández-Rodríguez

**Affiliations:** † Instituto de Química, Universidad Nacional Autónoma de México Circuito Exterior s/n, Ciudad Universitaria, Coyoacán, C.P. 04510 Cd. Mx., México; ‡ Departamento de Ciencias Químicas, Facultad de Estudios Superiores Cuautitlán-UNAM, Campo 1, Avenida 1ro. de mayo s/n, Cuautitlán Izcalli, Estado de México C.P. 54740, México

## Abstract

β-Nitrostyrenes are archetypal Michael acceptors due to the strong electron-withdrawing nature of the nitro group. However, we found that β-nitrostyrenes substituted with electron-withdrawing groups react with silyl ketene acetals, activated by stoichiometric Lewis bases (K_2_CO_3_ or TBAF), to produce a mixture of Michael addition (M) and anti-Michael addition (AM) products. The yield strongly depends on the position and nature of the electron-withdrawing group within the phenyl ring (e.g., *p*-nitro: 8% AM 2% M; *o*-nitro 70% AM, 26% M). The formation of the unexpected AM product cannot be explained by a higher electrophilicity of the α-carbon compared to the β-carbon (Parr’s indexes). Furthermore, we demonstrated that the formation of the AM product is not influenced by external factors such as light, metal impurities, or the nature of the Lewis base. Instead, it appears from the intrinsic reactivity of the reacting partners. Based on theoretical and experimental evidence, we propose that the AM product is formed via a vinylic S_RN_1 mechanism rather than an ionic pathway. In this mechanism, the anionic enolate reduces the nitrostyrene through a single-electron transfer process initiating a radical mechanism that ultimately leads to the formation of the AM product.

## Graphical abstract

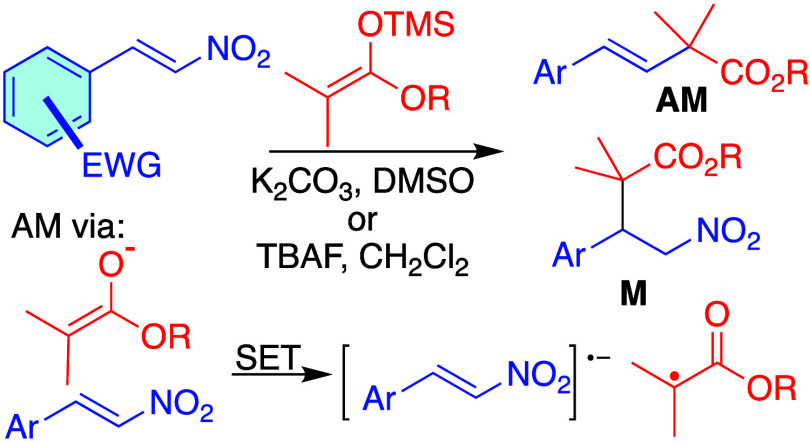

Michael addition takes place between electron-deficient alkenes conjugated to an electron-withdrawing group (EWG) and the anion formed by deprotonation of the nucleophile (stabilized by an EWG). The formation of a new bond at the β position of the α,β-unsaturated system occurs due to the stabilization of the anion by the EWG in the Michael acceptor.^1^ However, in some cases, a competitive addition occurs at the α carbon of the double bond, known as an anti-Michael addition.^2^ (Scheme 1a). The anti-Michael addition depends on the substrates and their electronic and steric environment. A common characteristic among such reactions is that the substituents at the β position of the alkene R^1^ possess strong electron-withdrawing properties, such as perfluorinated carbons, aryl groups with multiple electron-withdrawing substituents, other EWGs like β-nitroacrylates, or structures like sulfonylacetylenes^3^ (Scheme 1b). In these reports of anti-Michael addition ionic mechanisms are proposed. In this work, we reacted silyl ketene acetals with β-nitrostyrenes containing an EWG obtaining both Michael and anti-Michael additions (Scheme 1c). We propose that a radical pathway is responsible for the unexpected anti-Michael addition.

**Scheme 1:**
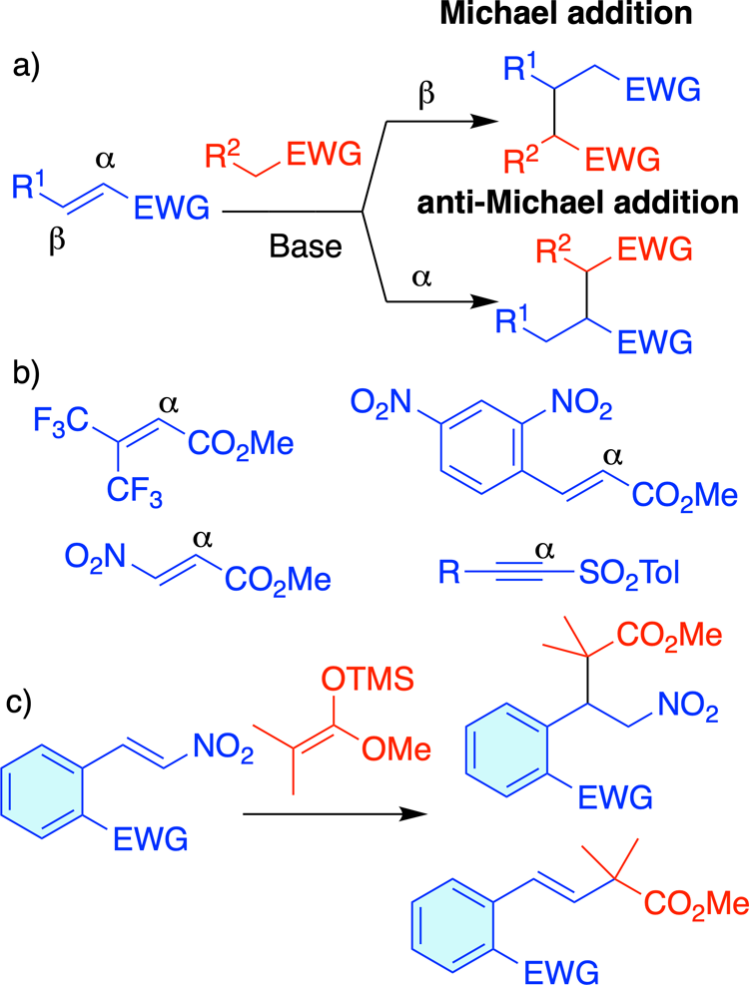
(a) Michael/anti-Michael Additions. (b) Reported Electrophiles that Proceed with an Anti-Michael Regioselectivity. (c) Competitive Michael/anti-Michael Addition Studied in this Work

## Results and Discussion

We recently reported a cascade reaction between *trans*-2-phenylethynyl-β-nitrostyrene **1a** and 1,3-dicarbonyl compounds promoted by potassium carbonate. This reaction afforded Indane-fused dihydrofurans **2** via a Michael-nitro-Conia-ene-S_N_2 sequence under mild conditions^4^ (Scheme 2a). Currently, we are exploring other dinucleophiles suitable for this cascade reaction. Among them, bistrimethylsilylketene acetals (known for their nucleophilicity at both carbon and oxygen atoms)^5^ are useful for synthesizing various scaffolds, including β-amino acids,^6a^ lactones fused to heterocycles,^6b,6c^ and allenes.^6d^ Therefore, we investigated the same cascade reaction with this dinucleophile (research in progress). We were pleased to find the formation of the Indane-fused lactone **4** in 44% yield. However, to our surprise, we also obtained the unexpected compound **5**, formed through an anti-Michael addition and nitrite elimination, with a 31% yield (Scheme 2b).

**Scheme 2:**
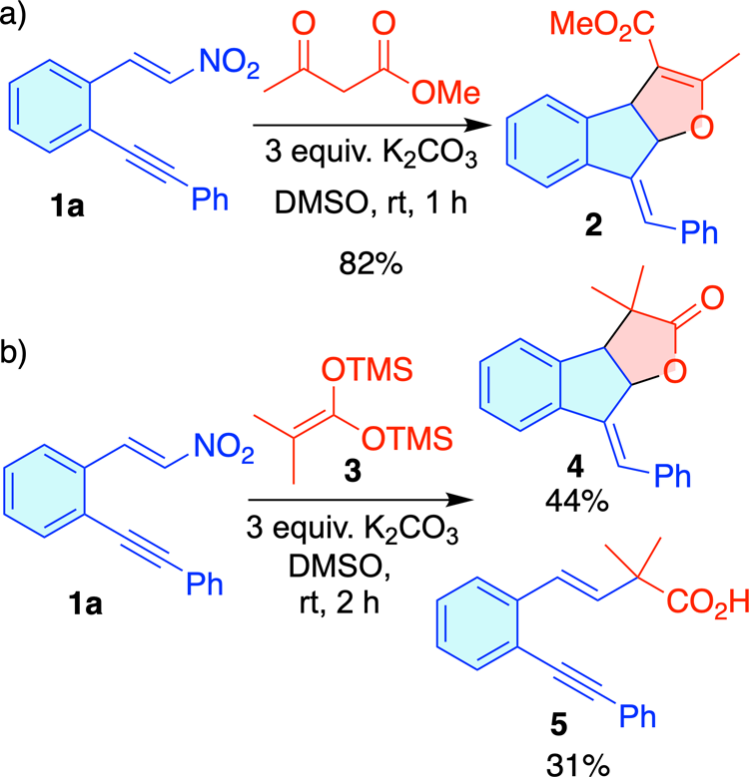
Cascade Reaction between Dinucleophiles and Nitroalkene **1a**

The addition of trimethylsilyl enol ethers derived from esters to β-nitrostyrenes is a well-known reaction.^7^ However, to the best of our knowledge, there are no reports of this reaction being promoted by a Lewis base when electron-withdrawing groups are present on the phenyl ring. The phenyl ethynyl group is known for its electron-withdrawing properties, as documented by Katz,^8^ and has been utilized in the synthesis of heterocycles using ortho-fluoro-ethynylbenzenes.^9^

Nevertheless, the nitro group is one of the strongest electron-withdrawing groups, making it challenging for an aryl group with an attached electron-withdrawing substituent to surpass its influence to alter the regioselectivity and promoting a competitive anti-Michael addition. To better understand this intriguing result, we explored the addition of silyl ketene acetals to substituted β-nitrostyrenes. As a model reaction for this study, we used 2-nitro-β-nitrostyrene **1b** and used enol ether **3′**. Under the same conditions, after 2 h, we observed the anti-Michael product **6b** in moderate yield, along with a small amount of the Michael product **7b**. NMR monitoring of the reaction revealed that both Michael **7b** and anti-Michael **6b** products were formed at the beginning of the reaction. While the anti-Michael product showed a slight increase, the Michael product **7b** gradually degraded until it completely disappeared (see Figure S1 in the SI). Additionally, under these conditions, we saw the formation of aromatic byproducts. To address this, we explored shorter reaction times and found that with reaction times under 10 min, there was no significant loss of the Michael product **7b**. Scheme 3 shows the yields of **6b** and **7b** at different reaction times.

**Scheme 3:**
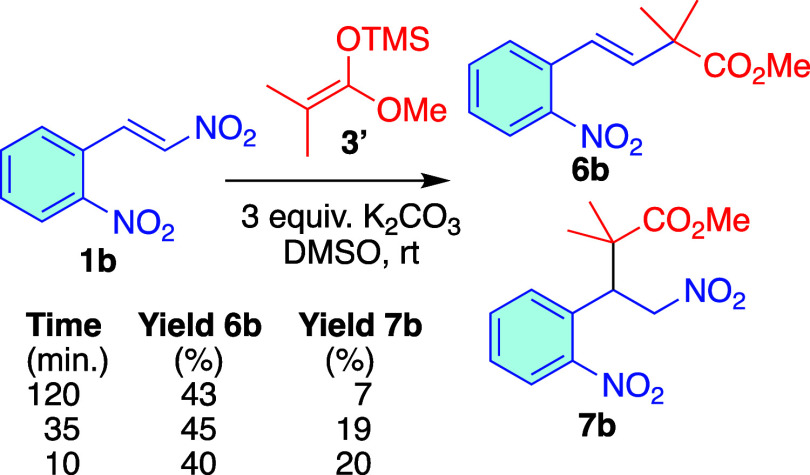
Michael/Anti-Michael Addition of **3′** to Nitroalkene **1b**

We aimed to identify conditions that would selectively produce **6b** over **7b**. However, under the tested conditions, yields consistently remained below 40%. Notably, we observed a shift in regioselectivity with the absence (or present in minimal amounts) of potassium carbonate (Table 1, also, Figure S2 in the SI shows the monitoring of the reaction without K_2_CO_3_ in DMSO). Moreover, the reaction also proceeded in DMF and using TBAT instead of potassium carbonate.

**Table 1:**
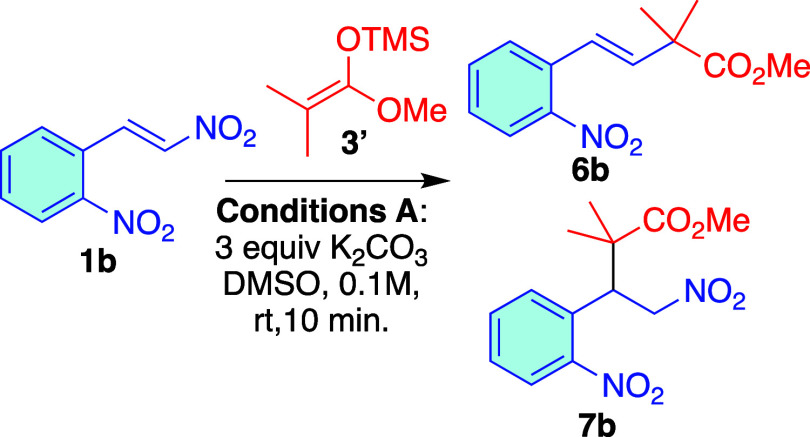
Exploration of Different Modifications to Conditions **A** ^a^

Exp.	Equiv K_2_CO_3_	Different from cond. **A**	**6b** (%)	**7b** (%)	**1b** (%)
1	3		40	20	0
2	6		39	16	0
3	1	20 min	34	18	0
4	0.5	20 min	37	22	0
5	0.1	1 h	20	34	10
6	0.01	1 h	9	26	36
7	0.01	20 h	1	30	6
8	0	21 h	1	25	50
9	0	10 M, 27 h	1	20	17
10	3	40 °C	41	20	0
11	3	DMF	42	24	0
12	3	DMF, −5 °C 2 h	46	22	0
13	0.1	DMF, 2 M	12	49	0
14	0.1^b^	TBAT, 1.5 h	17	40	12
15	0.1^b^	TBAT, 13 h	20	42	0

a Reaction yields of **6b** and **7b** and unreacted **1b** were obtained by ^1^H NMR.

b Tetrabutylammonium difluorotriphenylsilicate (TBAT) in DMSO used instead of K_2_CO_3_.

Before examining the scope of the competing Michael/anti-Michael addition, we explored an alternative reaction condition reported to activate these nucleophiles using a catalytic amount of tetrabutylammonium difluorotriphenylsilicate (TBAT) in THF,^6a^ labeled as Conditions **B** (Scheme 4). These conditions required a longer time to complete the reaction. In contrast to conditions **A** the Michael product **7b** is the major compound. Notably, ^1^H NMR monitoring under these conditions showed that the ratio of **6b** to **7b** remained unchanged over time (see Figure S3 in the SI).

**Scheme 4:**
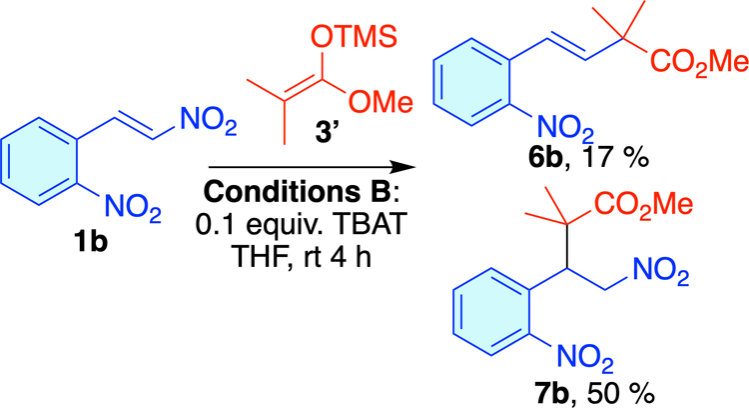
Addition of Ketene Silyl Acetal **3′** to Nitroalkene **1b** under Conditions **B**

The competitive Michael/anti-Michael addition of compound **3′** to nitroalkenes **1a–za** (Scheme 5) revealed the following trends: (i) ortho-substituted phenyl rings bearing electron-withdrawing groups, the anti-Michael product predominated under conditions **A** (**1a–d**, **1u–v**). (ii) Under the same conditions, when electron-withdrawing groups were positioned at the meta or para positions, the anti-Michael product **6** was either the major product or entirely absent from the reaction mixture altogether (**1g**–**n**). The Hammett parameter offers a qualitative insight into the formation of product **6**. For example, the *p*-CO_2_Me and *m*-CN have a Hammett σ value of 0.45 and 0.56 respectively favors anti-Michael addition, whereas halogen substituents in the para position (Cl, and Br) have a lower σ value of 0.23, do not promote the formation of product **6**. (iii) Unsubstituted phenyl ring, substituted with electron-donating groups, or nitroalkenes bonded to alkenyl and alkyl groups resulted in the exclusive formation of the Michael product **7** (**1o**–**q**, **1t**, **1w**). (iv) Perflurinated phenyl ring and pyridine instead of phenyl, also produced some anti-Michael product **6** (**1r**–**s**). We stress that the low yield of the products is not due to unreacted nitroalkene but rather to the formation of a mixture of aromatic compounds besides the two compounds described. This side product formation prevents a quantitative correlation between Hammett values and the yield of compound **6**. (see Figure S4 in the SI to see the comparison of the crude reaction mixture of **1b** and **1g**). The formation of these byproducts apparently decrease with methyl substitution at either the β- or α-position of the nitroalkenes (**1z** and **1za** vs **1i**), resulting in higher amounts of both compounds **6** and **7**. Furthermore, a comparison of two nitroalkenes with a phenyl ring containing an *ortho*-nitro group (**1b** vs **1y**) showed that methyl substitution at the α-position decreased the yield of the anti-Michael product **6**, possibly due to steric hindrance or the rupture of the planarity between the alkene and the aryl moieties. (v) Under conditions **B**, the Michael product **7** consistently emerged as the major compound, with the only exceptions being the strongly electron-withdrawing dinitroaryl derivative **1v** and the methyl-substituted **1za**. Therefore, conditions **B** could serve as an effective strategy for obtaining **7** when desired. Additionally, the trends observed among the nitrostyrenes under conditions **A** were also present under conditions **B**, although with varying magnitudes. For example, **1b** yielded more of the anti-Michael product than **1g** under both sets of conditions.

**Scheme 5:**
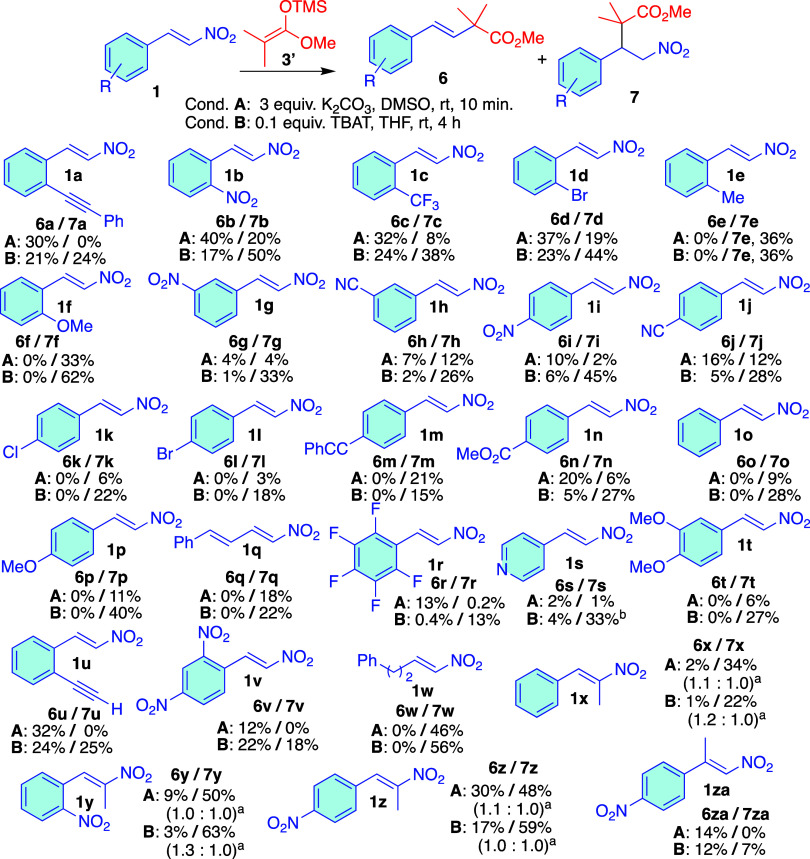
Michael/Anti-Michael Addition of **3′** to Different β-Nitrostyrenes **1** ^c^

a Diastereomeric ratio of Michael products **7** or *E*/*Z* ratio of product **6**.
b Isolated yield of **7s** by procedure **B** was 5%.
c The yield of the products was obtained by ^1^H NMR (see experimental details in the SI).

The reaction was further studied using various bistrimethylsilylketene acetals (**3a–h**). Most substrates performed poorly under conditions **A**. However, under conditions **B**, cyclic nucleophile **3b** exhibited similar preference for anti-Michael addition compared to the acyclic **3a**. The reaction did not proceed with nucleophiles that had a phenyl ring attached to the double bond (**3c**, **3h**). Also, the unsubstituted compound **3d** led exclusively to decomposition, as well as monosubstituted bistrimethylsilyl ketene acetals (**3e–h**), only with **3e** we were able to measure the yield with confidence and isolate pure compounds (Scheme 6).

**Scheme 6:**
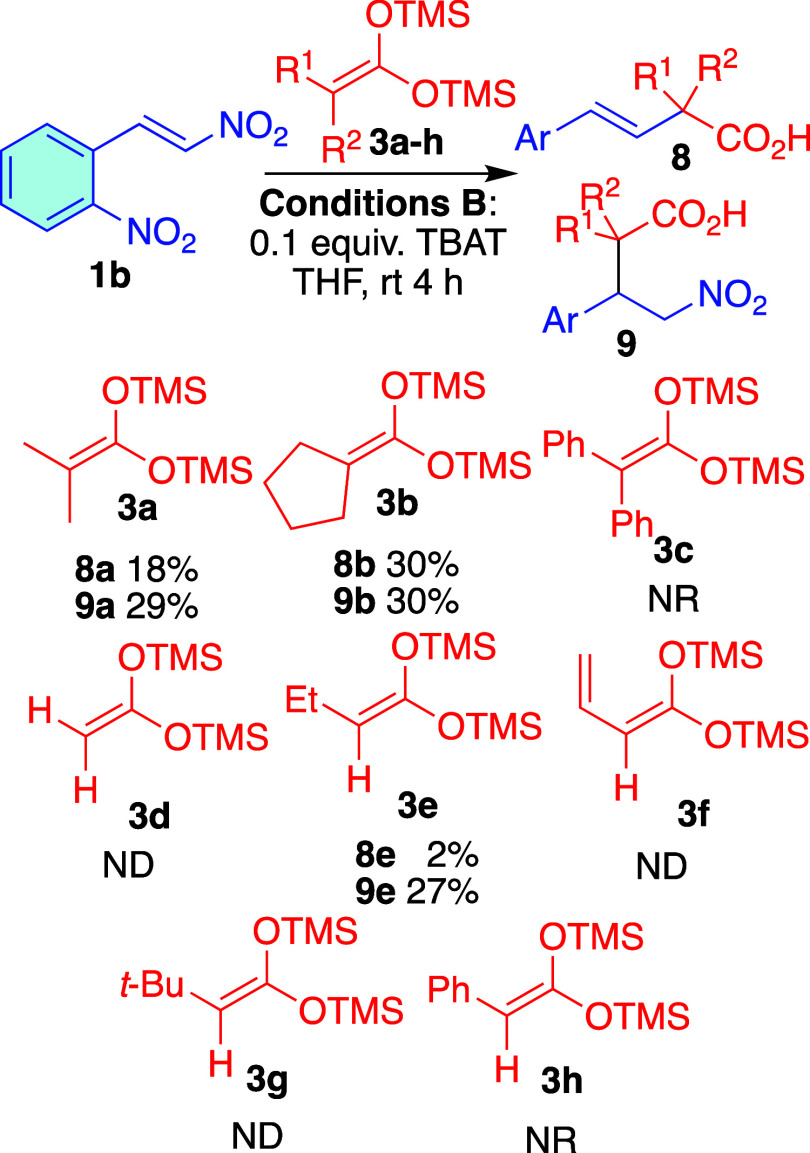
Michael/Anti-Michael Addition of **3a**–**h** to β-Nitrostyrene **1b** ^a^

a The yield of the reactions was obtained by ^1^H NMR (se experimental details in the SI). Compound **9e** dr 1:1.

To account for the unexpected anti-Michael addition, we explored the possible underlying mechanisms. The nucleophilic vinylic substitution^10^ (S_N_V) mechanism involves the nucleophilic attack on the double bond, leading either to the formation of an anionic intermediate (S_N_Vπ) or by a transition state that simultaneously forms and breaks sigma bonds (S_N_Vσ) (Scheme 7a,b). Additionally, it is known that a radical can be added at the α position of nitroalkenes.^11^ This radical may be generated by an oxidant like Iron­(III), which oxidizes the nucleophile before it adds to the nitroalkene, subsequently leading to the elimination of a nitrite radical^12^ (Scheme 7c). This radical pathway can also be achieved by the formation of an electron donor–acceptor complex followed a single-electron transfer (SET) from the nucleophile^13^ (Scheme 7d). Finally, the [4 + 2] cycloaddition leading to the formation of the Michael product **7** has been reported.^14^ However, explaining the formation of the anti-Michael product **6** would require the unlikely formation of the regioisomer followed by subsequent migrations (Scheme 7e).

**Scheme 7:**
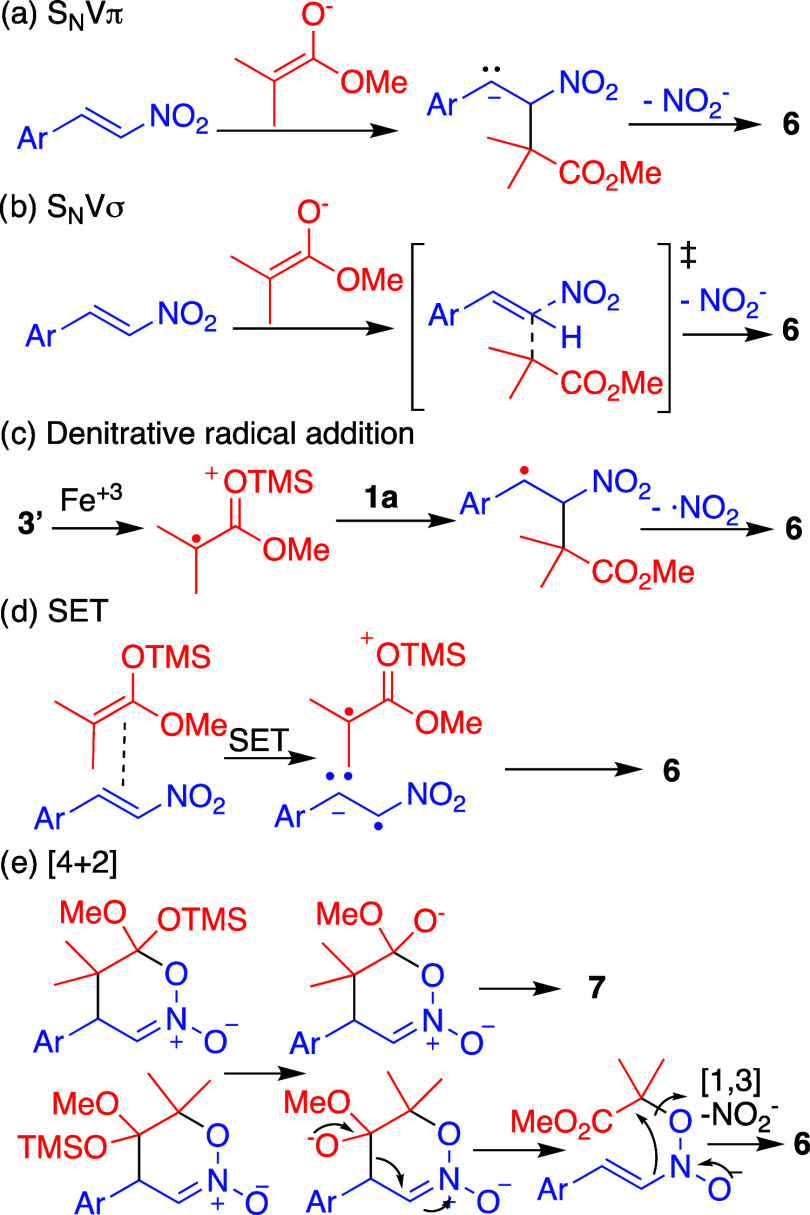
Possible Mechanism to the Formation of the Anti-Michael Product **6**

We conducted experiments trying to elucidate the pathway leading to compound **6b**. First, we aimed to isolate the reaction from external factors. To this end, we performed a control experiment without a stirring bar and using potassium carbonate of 99.995% trace metal purity under an argon atmosphere, shielded from light. The results remained the same as before (Table 2, exp. 1–2). Thus, contamination by iron, other metals, or oxygen can be ruled out as an oxidant to the nucleophile (Scheme 7c). We performed UV–vis experiments and found that the mixture of **1a** and **3′** in DMSO showed a new absorption peak around 500 nm, (Figure S5 in the SI). However, considering the control experiment carried out in the absence of light, as well as the reactions performed under green light and shielded with aluminum foil (to perform the experiment at the same temperature) yielded similar results, (Table 2, exp. 3–4), the involvement of an EDA complex initiating a radical pathway via light absorption can be ruled out.

**Table 2:**
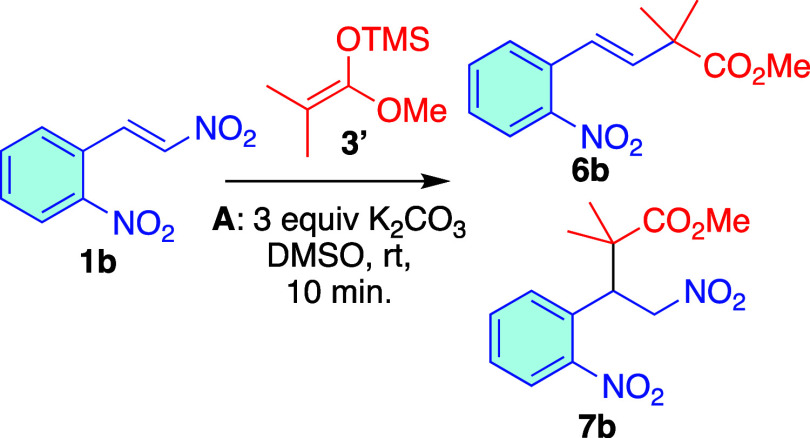
Designed Experiments to Elucidate the Mechanistic Pathway

			Yields (%)^a^		
Exp.	K_2_CO_3_ equiv.	Deviation from Cond. **A**.	**6b**	**7b**	**1b**
1	3		40	20	0
2	3	Control exp.^b^	40	20	0
3^c^	0.1	Green light, 30 min	39	26	1
4^c^	0.1	Shielded from green light 30 min	42	25	0
5^c^	0	2 h	4	41	40
6^c^	0	2 h then + **3′** 2 h	11	52	14
7	3	TEMPO (0.25)	41	17	0
8	3	TEMPO (2.5)	47	13	0
9	3	TEMPO (1.2), 3 min	52	30	0
10	3	TEMPO (1.2) DMF −5 °C	42	11	0
11	3	2,6(*t*-Bu)PhOH (1.2)	25	17	0

a All the yields were measured by ^1^H NMR with an added standard. The yield of **1b** represents unreacted compound.

b Experiment with K_2_CO_3_ 99.995% trace metal basis, without stirring bar, under argon, dry DMSO and shielded from light.

c Since the reaction proceeds very quickly, we compared conditions using a small amount of base and no base.

Monitoring the reaction by NMR, both with and without base (Figures S1 and S2 in the SI), revealed no cycloaddition product, allowing us to rule out this pathway as a feasible mechanism. Moreover, in the absence of base, we observed that the nucleophile was depleted after approximately 1.5 h. To investigate further, we conducted an experiment in which additional nucleophile was added after 2 h. This resulted in a slight increase in both **6b** and **7b** (Table 2, exp. 5–6), but it also revealed the presence of a background reaction that prevents the quantitative formation of the desired products. We support this observation because the combined yield of **6b**, **7b**, and **1b** decreased from 85 to 74% after the second addition of the nucleophile.

We performed an EPR experiment with a mixture of **1b** and **3′** in DMSO and detected a radical signal coupled with nitrogen (Figure S6 in the SI). This finding rules out an ionic pathway (at least for the anti-Michael compound), indicating that the reaction proceeds via a SET mechanism, generating a radical stabilized on both carbons, leading to competitive regioselectivity. Interestingly, the addition of the radical acceptor TEMPO increased the formation of anti-Michael product **6**, whereas 2,6-di*tert*-butylphenol diminished it (Table 2, exp. 7–11).

Furthermore, the solvent and/or the potassium carbonate, may contribute to the observed anti-Michael addition. To investigate this possibility, we conducted experiments under previously reported conditions using 2 equiv of TBAF in DCM at −40 °C.^14c^ With nitroalkene **1b**, the results were similar to those obtained under conditions **A**. However, when the reaction temperature was lowered to −78 °C, the yield of both products increased, with a particularly notable increase in **6b** (Table 3, experiments 1–2).

**Table 3:**
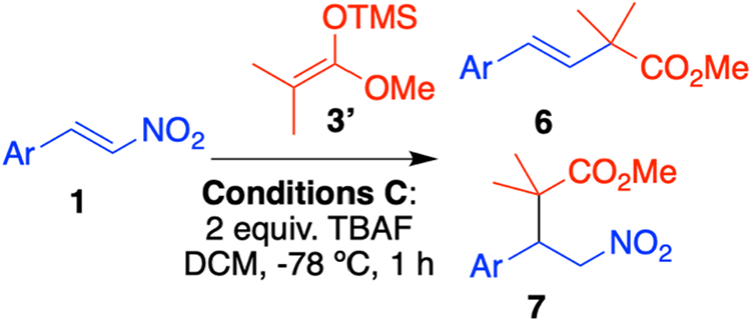
Michael and Anti-Michael Addition of **3′** to Nitrostyrenes under Conditions **C**

			Yields (%)^a^	
Exp.	Ar	Products **6**/**7**	**6**	**7**
1 (−40)	2-NO_2_C_6_H_4_	**6b**/**7b**	44	18
2	2-NO_2_C_6_H_4_	**6b**/**7b**	70	26
3	3-NO_2_C_6_H_4_	**6g**/**7g**	8	31
4	4-NO_2_C_6_H_4_	**6i**/**7i**	8	2
5	2-CF_3_C_6_H_4_	**6c**/**7c**	41	53
6	2-(CCPh)C_6_H_4_	**6a**/**7a**	26	67
7^b^	2-(CCPh)C_6_H_4_	**5/7a′**	5	39
8	3-CNC_6_H_4_	**6h**/**7h**	11	45
9	4-CNC_6_H_4_	**6j**/**7j**	31	44
10	Ph	**6o**/**7o**	0	23
11	2-MeC_6_H_4_	**6e**/**7e**	0	84

a All the yields were measured by ^1^H NMR with an added standard.

b It was used silyl ketene acetal **3** instead of **3′**.

Using these conditions, referred to as conditions **C**, we tested a range of representative nitroalkenes. In general, conditions **C** resulted in higher yields and cleaner reactions compared to conditions **A**. However, for substrates that yielded low amounts of product and significant impurities under conditions **A**, such as the *p*-nitro compound (**6i** and **7i**, Table 3), similar outcomes were observed under conditions **C**. Moreover, we scaled up the reaction with **1b** to a 1 mmol scale, obtaining 60% of **6b** and 16% of **7b** of isolated yield.

Additionally, various silyl ketene acetals **3** and enol ethers **3i** and **3j** were added to **1b** under conditions **C**. The disubstituted silyl ketene acetals **3a–b** produced a substantial amount of the anti-Michael product **6**, whereas the monosubstituted nucleophile **3e** yielded significantly less. Besides, the enol ethers from methylcyclopentanone **3i** and the 1,3-dicarbonyl compound **3j**, only the Michael product was detected. This suggests that nucleophilicity and HOMO energy play a crucial role in the formation of the anti-Michael product (Scheme 8).

**Scheme 8:**
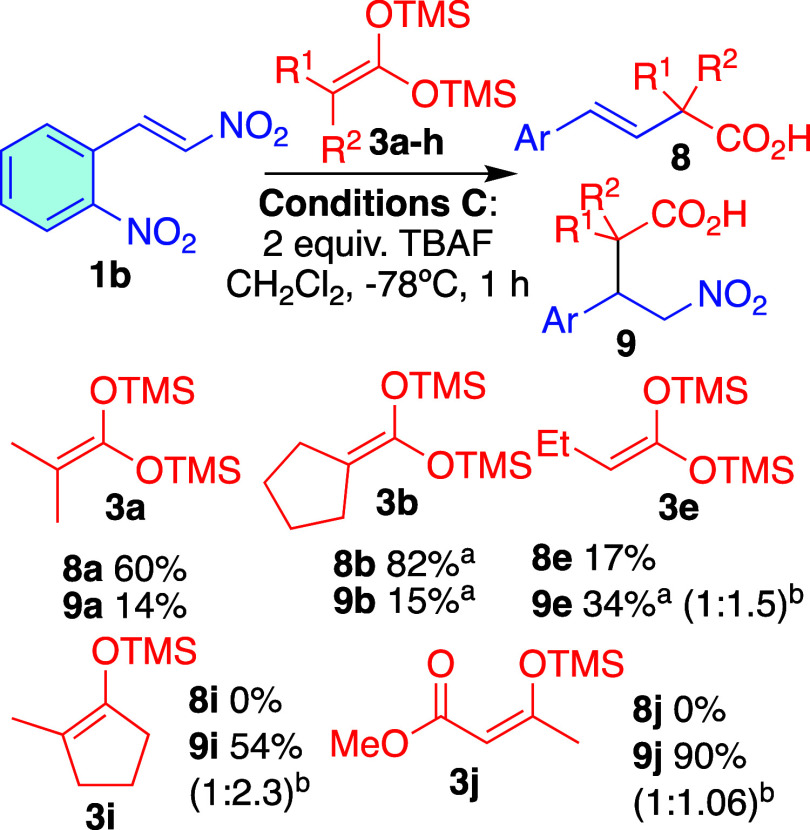
Michael and Anti-Michael Addition of Nucleophiles **3** to Nitrostyrene **1b**, Yields were Measured by ^1^H NMR with an Added Standard

a The isolated yield of **8b**, **9b**, and **9e** was 19, 9, and 21% respectively.
b Diastereomeric ratio of **9**.

The preference for anti-Michael addition observed under conditions **C** is comparable to or even greater than that observed under conditions **A**. This suggests that neither the solvent nor the Lewis base is essential for obtaining the anti-Michael product. Instead, the key requirements appear to be the use of a noncatalytic Lewis base (as in conditions **B**) and, as previously mentioned, the presence of an electron-withdrawing group (EWG) on the aryl ring. Since, in the absence of potassium carbonate only the Michael adduct is formed, this is a viable pathway to product **7** (Mechanism A in Scheme 9). Additionally, the free enolate can react with the nitroalkene through a typical ionic Michael addition (mechanism B, Scheme 9). However, when nitrostyrenes bear an EWG, a competing pathway is enabled, initiating with a single-electron transfer (SET) as the first step. This process generates the radical anion of the nitrostyrene and a radical at the α-position of the carbonyl group (mechanism C, Scheme 9). These radicals can potentially combine at either the α- or β-position. Combination at the β-position generates a nitronate intermediate, which subsequently protonates to form compound **7**. Bonding at the α-carbon, on the other hand, leads to the formation of an anion stabilized by the aryl group, followed by the elimination of nitrite to yield compound **6**. Furthermore, it is also plausible that the radical anion follows an S_RN_1 mechanism, generating a vinylic radical through the formation of a nitrite anion. This vinylic radical then adds to the enolate, forming a radical stabilized by neighboring oxygens. A subsequent single-electron transfer with another molecule of nitrostyrene **1** leads to the formation of another radical anion of **1** and ultimately results in the formation of product **6**. The proposal of a vinylic radical-nucleophilic substitution S_RN_1 (V) mechanism is supported by previous reports of this pathway.^13b,15^ Moreover, it has been demonstrated that S_RN_1 mechanisms can operate in reactions typically considered ionic, such as S_N_2 reactions and nucleophilic additions to carbonyl compounds.^16^ Therefore, if this is a broader mechanism, there is no reason why other electrophiles, such as nitrostyrenes, would be exceptions. The key requirement is the presence of a suitable nucleophile capable of initiating a SET reaction as the first step, thereby triggering this reaction. It is worth noting that the oxidation of silyl enol ethers via SET mechanisms has been reported.^17^ However, **3**′ has an oxidation potential of 0.90 V, which is insufficient to reduce the *p*-nitro derivatives **1i** and **1z** that yield the anti-Michael addition product (their reduction potentials at pH 10 are −0.399 and −0.405 V, respectively).^18^ Thus, only the “free enolate” is capable of facilitating this electron transfer. It is worth noting that nitrostyrenes that do not undergo anti-Michael addition (**1o**, **1p**, and **1x**) exhibit more negative reduction potentials of −0.483, −0.553, and −0.613 V, respectively, indicating a threshold potential for this mechanism to proceed.

**Scheme 9:**
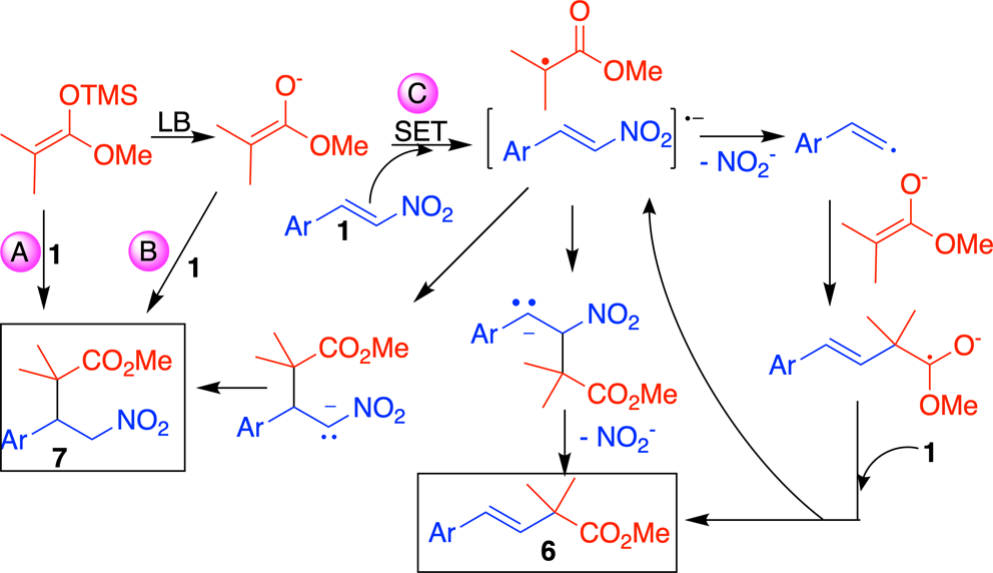
Mechanisms to Obtain Compounds **6** and **7** ^19^

We also investigated the regioisomeric nucleophilic addition using DFT calculations to assess the impact of structural modifications on the nitrostyrene to the selectivity observed. Specifically, we calculated the electrophilic Parr function at the α and β carbons as a local reactivity index^20^ (See Table S1 in the SI). The values of the Parr function were generally higher at the β position (except for the *p*-NO_2_ compounds **1i**, **1v**, and **1za**), indicating the inherent reactivity of these compounds at that site. However, examining the difference in the values between the α and β positions, a trend emerged (Figure 1). Specifically, if the difference exceeded 0.11 au, the compounds **1** consistently underwent exclusive Michael addition under conditions **A** (green bars in Figure 1). In contrast, when a mixture of products **6** and **7** was observed, the difference in Parr function values was below this threshold (maroon bars in Figure 1, with the exceptions of compounds **1m** and **1q**).

**Figure 1:**
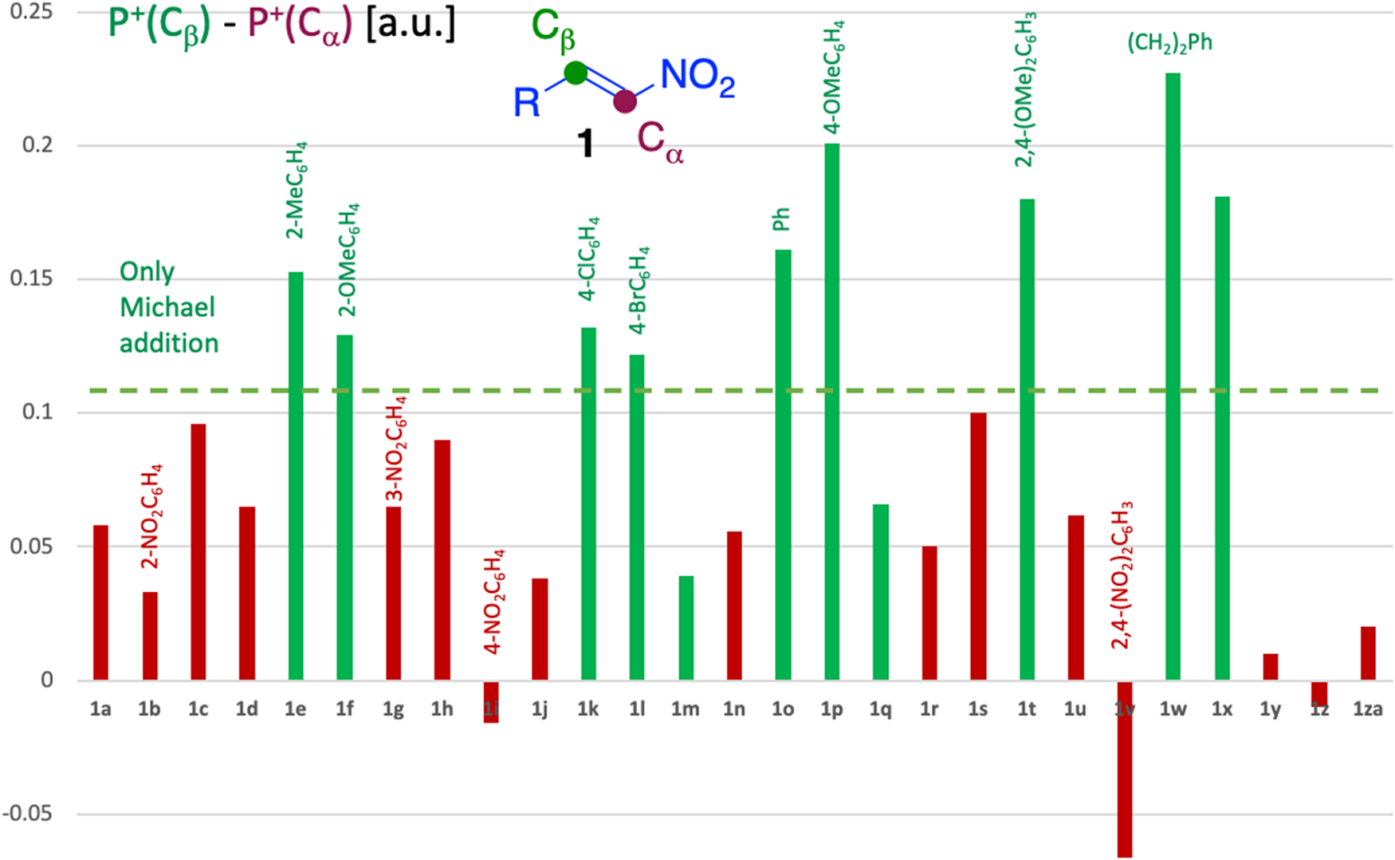
Difference in electrophilic Parr function values between the α and β carbons of nitrostyrenes.

In addition, we found another qualitative correlation between the regioselectivity under conditions **A** and the LUMO energy of the nitrostyrene **1**. This was aiming to find a relationship between the electron-accepting ability of compounds **1** and its propensity for single-electron transfer reactions. Using the LUMO energy of unsubstituted nitrostyrene (−1.98 eV) as a threshold, all nitrostyrenes with higher LUMO energies (e.g., o-OMeC_6_H_4_ **1f**, −1.72 eV) yielded exclusively Michael addition product. In contrast, nitrostyrenes with lower LUMO energies (e.g., 2-NO_2_C_6_H_4_, −2.37 eV) exhibited competitive anti-Michael addition (Figure 2 and Table S1 in the SI). Exceptions were again observed—namely, compounds **1m** and **1q**, as well as the para-halogenated derivatives **1k** and **1l**. Despite these general trends, neither this analysis nor the Parr function comparison produced a quantitative correlation between the **6**/**7** product ratio and the electronic properties studied. This lack of correlation is likely due to the formation of byproducts, which reduce the yields of the desired products to varying extents (as noted earlier, most nitrostyrenes 1 are consumed, but not fully converted into compounds **6** and **7**).

**Figure 2:**
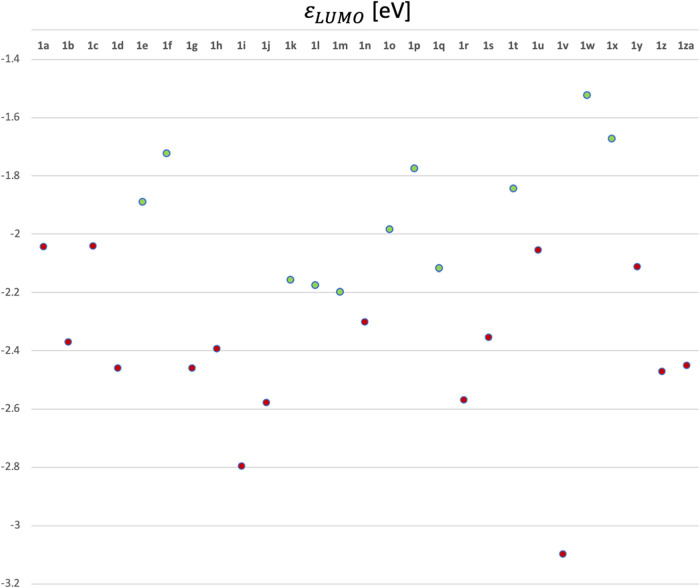
LUMO energies of nitroalkenes **1a-za**. Calculations were carried out using M062X-D3 exchange-correlation functional combined with the 6-311++G­(d,p) basis set.

In conclusion, we present a reaction of competitive anti-Michael/Michael addition of silyl ketene acetals with nitrostyrenes bearing electron-withdrawing groups under equimolar Lewis base activation. Our results suggest a potential single-electron transfer mechanism for the formation of the anti-Michael product. Furthermore, these findings raise intriguing and as-yet unanswered questions. For example, what are the byproducts obtained in these reactions? Why is there such a stark contrast in yield between p-nitro and o-nitro substituents on the aryl ring of the nitrostyrene? The formation of the anti-Michael product prompt us to question whether the observed results represent a unique and unanticipated phenomenon or whether they point to a broader, previously overlooked mechanism for other Michael additions. Maybe results that have disregarded as “unsuccessful experiments” were because a similar mechanism is taking place. Therefore, it would be crucial to fully understand the reaction to control it and take synthetic advantage of the anti-Michael product. These unexpected findings highlight the value of organic chemistry in revealing uncharted dimensions within systems previously believed to be well-understood.

## Experimental Section

### General Considerations

Starting materials were commercially available and used as received. Tetrahydrofuran was distilled from sodium benzophenone, dichloromethane was distilled from calcium hydride and dimethyl sulfoxide was dried over molecular sieves (4 Å). Reactions were monitored by thin-layer chromatography (TLC) on silica gel plates F_254_ and the spots were detected either by UV absorption, Seebach’s stain or KMnO_4_. Flash column chromatography and Dry Column Vacuum Chromatography (DCVC) were carried out with silica gel 60 (0.4/0.63 mm, 230–400 mesh). ^1^H and ^13^C NMR spectra were recorded at ambient temperature using 300 MHz, 400 or 700 MHz spectrometers. Chemical shifts (δ) are reported in ppm relative to residual solvent signals (CDCl_3_ = δ 7.26 for ^1^H NMR, δ 77.16 for ^13^C NMR. DMSO-*d*_6_ = δ 2.50 for ^1^H NMR, δ 39.52 for ^13^C NMR) or TMS as internal reference (δ 0.0) and coupling constants are in hertz (Hz). Mass spectra were obtained by direct analysis in real time (DART) with TOF mass analyzer or by EI.

#### General Procedure, Conditions A

In a 4 mL screw-cap vial with stirring bar was dissolved 0.2 mmol (1.0 equiv) of β-nitrostyrene **1** in 2 mL of dry DMSO (0.1 M). The corresponding silylketene acetal **3** or **3′** 0.4 mmol (2.0 equiv) and potassium carbonate 83 mg, 0.6 mmol (3.0 equiv) were added. The reaction was stirred at 20 °C for 10 min. The resulting black solution was diluted with 7 mL of water and acidified to pH ∼ 4 using a 10% hydrochloric acid solution. The products were extracted with EtOAc (10 mL × 3). The combined organic layers were washed with brine, dried over sodium sulfate (Na_2_SO_4_), filtered, and concentrated under reduced pressure. The crude reaction mixture was analyzed by ^1^H NMR, with 4-nitrobenzaldehyde (15–17% mol) used as the internal standard and purified by flash column chromatography or DVCV.

#### General Procedure, Conditions B

A stirred mixture of β-nitrostyrene **1** (0.2 mmol, 1.0 equiv) in 2 mL of dry THF was added the corresponding silylketene acetal **3** or **3′** (0.2 mol, 2.0 equiv) and tetrabutylammonium difluorotriphenylsilicate (TBAT) (10.8 mg, 0.02 mmol, 0.1 equiv). The reaction was stirred at 20 °C for 4 h, then quenched with 7 mL of water and acidified to pH ∼ 4 using a 10% hydrochloric acid solution. The mixture was then extracted with ethyl acetate (EtOAc) (7 mL × 3). The combined organic layers were washed with brine, dried over sodium sulfate (Na_2_SO_4_), and concentrated under reduced pressure. The crude reaction mixture was analyzed by ^1^H NMR, with 4-nitrobenzaldehyde (15–17% mol) used as the internal standard. It was purified by flash column chromatography or DVCV.

#### General Procedure, Conditions C

A screw-cap vial containing a stirred solution of β-nitrostyrene **1** (0.2 mmol, 1.0 equiv) was placed under inert atmosphere and dissolved in 2 mL of DCM. It was cooled to −78 °C, then added the corresponding silylketene acetal **3** or **3′** (0.4 mmol, 2.0 equiv) to the reaction mixture, followed by a dropwise addition of 0.4 mL (0.4 mmol, 2.0 equiv) of a 1 M solution of Bu_4_NF in THF. The reaction was stirred at −78 °C for 1 h, then quenched with 7 mL of water, acidified with 10% HCl to pH ∼ 4, and extracted with EtOAc (10 mL × 3). The combined organic layers were washed with brine, dried over Na_2_SO_4_, and concentrated under reduced pressure. The crude reaction mixture was analyzed by ^1^H NMR using 15–17 mol % of 4-nitrobenzaldehyde as an internal standard and products purified by flash column chromatography or DCVC.

##### (*Z*)-8-Benzylidene-3,3-dimethyl-3,3a,8,8a-tetrahydro-2*H*-indeno­[2,1-*b*]­furan-2-one (**4**)

Purification by flash column chromatography on silica gel using hexane/ethyl acetate (8:2). Physical state: pale yellow oil. Isolated yield: 44% (25.5 mg) from conditions **A**. ^1^H NMR (CDCl_3_, 300 MHz): δ 7.67–7.63 (m, 3H), 7.48–7.27 (m, 7H), 5.67 (dd, *J* = 6.0, 1.3 Hz, 1H), 3.72 (d, *J* = 6.0 Hz, 1H), 1.54 (s, 3H), 1.41 (s, 3H). ^13^C­{^1^H} NMR (CDCl_3_, 76 MHz): δ 181.3, 140.9, 140.5, 138.1, 136.1, 129.2, 128.8, 128.8, 128.6, 128.2, 127.6, 127.4, 121.0, 80.0, 54.8, 43.2, 28.1, 21.9. HRMS (EI): [M]^+^ Calcd. For C_20_H_18_O_2_: 290.1307; found: 290.1311.

##### (*E*)-2,2-Dimethyl-4-(2-(phenylethynyl)­phenyl)­but-3-enoic Acid (**5**)

Purification by flash column chromatography on silica gel using hexane/ethyl acetate (8:2). Physical state: yellow solid. Isolated yield: 31% (18 mg) from conditions **A**. m.p.: 98–100 °C. ^1^H NMR (400 MHz, CDCl_3_): δ 7.59–7.49 (m, 4H), 7.39–7.32 (m, 3H), 7.29 (td, *J* = 7.6, 1.6 Hz, 1H), 7.22 (td, *J* = 7.5, 1.3 Hz, 1H), 7.13 (d, *J* = 16.3 Hz, 1H), 6.53 (d, *J* = 16.3 Hz, 1H), 1.49 (s, 6H). ^13^C­{^1^H} NMR (100 MHz, CDCl_3_): δ 182.1, 138.6, 135.5, 132.5, 131.6, 128.6, 128.6, 128.5, 127.4, 127.1, 125.2, 123.5, 122.1, 94.4, 87.9, 44.7, 25.1. HRMS (DART/TOF): [M + H]^+^ Calcd. for C_20_H_19_O_2_: 291.1380, found: 291.1376.

##### Methyl (*E*)-2,2-Dimethyl-4-(2-(phenylethynyl)­phenyl)­but-3-enoate (**6a**)

Purification by DCVC using a solvent gradient from 60:40 to 47:53 hexane/DCM. Physical state: yellow oil. Isolated yield: 21% (12.7 mg) from conditions **B**. ^1^H NMR (CDCl_3_, 400 MHz): δ 7.59–7.49 (m, 4H), 7.40–7.33 (m, 3H), 7.32–7.27 (m, 1H), 7.24–7.16 (m, 1H), 7.07 (d, *J* = 16.3 Hz, 1H), 6.51 (d, *J* = 16.3 Hz, 1H), 3.69 (s, 3H), 1.47 (s, 6H). ^13^C­{^1^H} NMR (CDCl_3_, 100 MHz): δ 176.9, 138.7, 136.2, 132.5, 131.6, 128.6, 128.6, 128.5, 127.3, 126.5, 125.1, 123.5, 122.0, 94.3, 88.0, 52.3, 45.0, 25.3. HRMS (DART/TOF): [M + H]^+^ Calcd. for C_21_H_21_O_2_^+^: 305.1542; found: 305.1535.

##### Methyl 2,2-Dimethyl-4-nitro-3-(2-(phenylethynyl)­phenyl)­butanoate (**7a**)

Purification by two DCVC’s, the first one using a solvent gradient from 60:40 to 47:53 hexane/DCM. The second one using a solvent gradient from 1:0 to 80:20 DCM/AcOEt. Physical state: yellow oil. Isolated yield: 24% (17.0 mg) from conditions **B**. ^1^H NMR (CDCl_3_, 400 MHz) δ 7.63–7.58 (m, 2H), 7.56 (dd, *J* = 7.6, 1.5 Hz, 1H), 7.41–7.35 (m, 3H), 7.37–7.22 (m, 2H), 7.22 (dd, *J* = 7.8, 1.4 Hz, 1H), 5.01–4.74 (m, 3H), 3.73 (s, 3H), 1.31 (s, 3H), 1.22 (s, 3H). ^13^C­{^1^H} NMR (CDCl_3_, 100 MHz): δ 176.7, 138.5, 132.9, 131.7, 130.6, 128.6, 128.6, 127.8, 124.5, 123.3, 94.4, 88.0, 77.6, 52.4, 47.5, 46.4, 25.7, 21.8. HRMS (DART/TOF): [M + H]^+^ Calcd. for C_21_H_22_NO_4_^+^: 352.1549; found: 352.1547.

##### 2,2-Dimethyl-4-nitro-3-(2-(phenylethynyl)­phenyl)­butanoic Acid (**7a′**)

Purification by DCVC on silica gel using a gradient elution of hexane/ethyl acetate/AcOH (100:0:1 to 80:20:1) afforded the title compound as an orange oil (8.2 mg, 12% yield) from conditions **A**. ^1^H NMR (CDCl_3_, 400 MHz): δ 7.62–7.54 (m, 3H), 7.39–7.25 (m, 6H), 5.13–4.74 (m, 3H), 1.35 (s, 3H), 1.24 (s, 3H). ^13^C­{^1^H} NMR (CDCl_3_, 100 MHz): δ 181.89, 138.09, 132.98, 131.72, 128.70, 128.60, 127.97, 123.21, 94.60, 87.88, 46.13, 25.83. HRMS (DART/TOF): [M + H]^+^ Calcd. for C_20_H_20_NO_4_^+^: 338.1392; found: 338.1400.

##### Methyl (*E*)-2,2-Dimethyl-4-(2-nitrophenyl)­but-3-enoate (**6b**)

Purification by flash column chromatography on silica gel using hexane/ethyl acetate (9:1). Physical state: yellow oil. Isolated yield: 40% (19.9 mg) from conditions **A**. ^1^H NMR (CDCl_3_, 400 MHz): δ 7.93 (ddd, *J* = 8.2, 1.3, 0.5 Hz, 1H), 7.61–7.53 (m, 2H), 7.38 (ddd, *J* = 8.5, 6.9, 1.9 Hz, 1H), 6.93 (d, *J* = 16.0 Hz, 1H), 6.39 (d, *J* = 16.0 Hz, 1H), 3.72 (s, 3H), 1.44 (s, 6H). ^13^C­{^1^H} NMR (CDCl_3_, 100 MHz): δ 176.5, 148.0, 139.7, 133.2, 133.2, 128.9, 128.1, 124.7, 124.0, 52.4, 45.0, 25.1. HRMS (DART/TOF): *m*/*z* [M + H]^+^ Calcd. for C_13_H_16_NO_4_^+^: 250.1079; found: 250.1069.

##### Methyl 2,2-Dimethyl-4-nitro-3-(2-nitrophenyl)­butanoate (**7b**)

Purification by flash column chromatography on silica gel using hexane/ethyl acetate (9:1) followed by a second column using a gradient elution of hexane/dichloromethane (9:1 to 7:3). Physical state: yellow oil. Isolated yield: 50% (29.5 mg) from conditions **B**. ^1^H NMR (CDCl_3_, 400 MHz): δ 7.81 (dd, *J* = 8.1, 1.4 Hz, 1H), 7.57 (td, *J* = 7.7, 1.5 Hz, 1H), 7.46–7.38 (m, 2H), 5.03–4.88 (m, 2H), 4.65 (dd, *J* = 10.5, 4.3 Hz, 1H), 3.71 (s, 3H), 1.32 (s, 3H), 1.17 (s, 3H). ^13^C­{^1^H} NMR (CDCl_3_, 100 MHz): δ 176.1, 151.8, 132.7, 131.6, 129.0, 128.3, 125.3, 77.4, 52.6, 46.2, 43.8, 25.6, 23.0. HRMS (DART/TOF): *m*/*z* [M + H]^+^ Calcd. for C_13_H_17_N_2_O_6_^+^: 297.1087; found: 297.1086.

##### Methyl (*E*)-2,2-Dimethyl-4-(2-(trifluoromethyl)­phenyl)­but-3-enoate (**6c**)

Purification by flash column chromatography on silica gel using hexane/ethyl acetate (8:2). Physical state: colorless oil. Isolated yield: 32% (17.4 mg) from conditions **A**. ^1^H NMR (CDCl_3_, 400 MHz): δ 7.63–7.59 (m, 2H), 7.49 (t, *J* = 7.8 Hz, 1H), 7.32 (t, *J* = 7.5 Hz, 1H), 6.82 (dq, *J* = 16.0, 2.5 Hz, 1H), 6.36 (d, *J* = 16.0 Hz, 1H), 3.71 (s, 3H), 1.43 (s, 6H). ^13^C­{^1^H} (CDCl_3_, 100 MHz): δ 176.7, 138.7, 136.6, 131.9, 127.6, 127.3, 125.9 (q, *J* = 5.9 Hz), 124.6, 123.1, 52.3, 44.9, 25.2. HRMS (DART/TOF): *m*/*z* [M + H]^+^ Calcd. for C_14_H_16_F_3_O_2_^+^: 273.1102; found: 273.1097.

##### Methyl 2,2-Dimethyl-4-nitro-3-(2-(trifluoromethyl)­phenyl)­butanoate (**7c**)

Purification by flash column chromatography on silica gel using hexane/ethyl acetate (95:5) followed by a second column using a gradient elution of hexane/dichloromethane (9:1 to 7:3). Physical state: colorless oil. Isolated yield: 38% (24 mg) from conditions **B**. ^1^H NMR (CDCl_3_, 400 MHz): δ 7.70 (d, *J* = 7.8 Hz, 1H), 7.55 (t, *J* = 7.5 Hz, 1H), 7.45–7.36 (m, 2H), 5.02 (dd, *J* = 12.2, 4.1 Hz, 1H), 4.85 (dd, *J* = 12.2, 9.9 Hz, 1H), 4.18 (dd, *J* = 9.9, 4.2 Hz, 1H), 3.76 (s, 3H), 1.31 (s, 3H), 1.12 (s, 3H). ^13^C­{^1^H} (CDCl_3_, 100 MHz): δ 176.6, 136.6, 132.2, 128.3, 128.3, 127.1, 127.1, 79.0, 52.6, 46.5 (q, *J* = 2.2 Hz), 46.3, 26.4, 22.9. HRMS (DART/TOF): *m*/*z* [M + H]^+^ Calcd. for C_14_H_17_F_3_NO_4_^+^: 320.1110; found: 320.1117.

##### Methyl (*E*)-4-(2-Bromophenyl)-2,2-dimethylbut-3-enoate (**6d**)

Purification by flash column chromatography on silica gel using hexane/acetone (8:2). Physical state: pale yellow oil. Isolated yield: 37% (17.5 mg) from conditions **A**. ^1^H NMR (CDCl_3_, 400 MHz): δ 7.57–7.47 (m, 2H), 7.31–7.21 (m, 1H), 7.14–7.03 (m, 1H), 6.79 (d, *J* = 16.1 Hz, 1H), 6.35 (d, *J* = 16.1 Hz, 1H), 3.71 (s, 3H), 1.44 (s, 6H). ^13^C­{^1^H} (CDCl_3_, 100 MHz): δ 176.7, 137.4, 137.1, 133.0, 128.8, 127.6, 127.3, 127.2, 123.9, 52.4, 44.9, 25.2. HRMS (DART/TOF): *m*/*z* [M + H]^+^ Calcd. for C_13_H_16_BrO_2_^+^: 283.0334; found: 283.0325.

##### Methyl 3-(2-Bromophenyl)-2,2-dimethyl-4-nitrobutanoate (**7d**)

Purification by flash column chromatography on silica gel using hexane/ethyl acetate (98:2) followed by a second column using a gradient elution of hexane/dichloromethane (9:1 to 7:3). Physical state: colorless oil. Isolated yield: 44% (28.8 mg) from conditions **B**. ^1^H NMR (CDCl_3_, 400 MHz): δ 7.60 (dd, *J* = 8.1, 1.3 Hz, 1H), 7.33–7.27 (m, 1H), 7.19 (dd, *J* = 7.9, 1.8 Hz, 1H), 7.13 (td, *J* = 7.9, 1.7 Hz, 1H), 4.97–4.83 (m, 2H), 4.57 (dd, *J* = 10.7, 4.4 Hz, 1H), 3.74 (s, 3H), 1.32 (s, 3H), 1.19 (s, 3H). ^13^C­{^1^H} (CDCl_3_, 100 MHz): δ 176.4, 136.5, 133.9, 129.5, 128.0, 127.8, 127.6, 77.7, 52.5, 48.5, 46.4, 25.6, 22.1. HRMS (DART/TOF): *m*/*z* [M + H]^+^ Calcd. for C_13_H_17_BrNO_4_^+^: 330.0341; found: 330.0326.

##### Methyl 2,2-Dimethyl-4-nitro-3-(*o*-tolyl)­butanoate (**7e**)

Purification by DCVC using a solvent gradient from 1:0 to 90:10 hexane/AcOEt. Physical state: yellow oil. Isolated yield: 36% (19.1 mg) from conditions **A**. ^1^H NMR (CDCl_3_, 400 MHz): δ 7.21–7.12 (m, 3H), 7.12–7.07 (m, 1H), 4.92 (dd, *J* = 13.2, 11.0 Hz, 1H), 4.81 (dd, *J* = 13.2, 4.1 Hz, 1H), 4.18 (dd, *J* = 11.0, 4.1 Hz, 1H), 3.71 (s, 3H), 2.41 (s, 3H), 1.24 (s, 3H), 1.18 (s, 3H). ^13^C­{^1^H} (CDCl_3_, 100 MHz): δ 176.8, 138.3, 135.2, 131.2, 127.8, 126.3, 78.2, 52.4, 46.3, 45.2, 25.4, 22.1, 20.4. HRMS (DART/TOF): [M + H]^+^ Calcd. for C_14_H_20_NO_4_^+^: 266.1392; found: 266.1379.

##### Methyl 3-(2-Methoxyphenyl)-2,2-dimethyl-4-nitrobutanoate (**7f**)

Purification by flash column chromatography on silica gel using hexane/ethyl acetate (8:2). Physical state: yellow oil. Isolated yield: 33% (18.4 mg) from conditions **A**. ^1^H NMR (CDCl_3_, 400 MHz): δ 7.26–7.21 (m, 1H), 7.08 (dd, *J* = 7.6, 1.8 Hz, 1H), 6.93–6.86 (m, 2H), 4.99 (dd, *J* = 13.1, 10.8 Hz, 1H), 4.82 (dd, *J* = 13.1, 4.4 Hz, 1H), 4.30 (m, 1H), 3.81 (s, 3H), 3.70 (s, 3H), 1.24 (s, 3H), 1.12 (s, 3H). ^13^C­{^1^H} (CDCl_3_, 100 MHz): δ 176.8, 158.1, 129.1, 125.0, 120.7, 111.4, 55.7, 52.2, 45.8, 25.6, 22.3. HRMS (DART/TOF): *m*/*z* [M + H]^+^ Calcd. for C_14_H_20_NO_5_^+^: 282.1342; found: 282.1337.

##### Methyl (*E*)-2,2-Dimethyl-4-(3-nitrophenyl)­but-3-enoate (**6g**)

Purification by flash column chromatography on silica gel using hexane/ethyl acetate (85:15). Physical state: yellow oil. Isolated yield: 4% (2.0 mg) from conditions **B**. ^1^H NMR (CDCl_3_, 400 MHz): δ 8.27–8.20 (m, 1H), 8.07 (m, 1H), 7.69–7.65 (m, 1H), 7.48 (t, *J* = 8.0 Hz, 1H), 6.56 (d, *J* = 16.2 Hz, 1H), 6.48 (d, *J* = 16.3 Hz, 1H), 3.72 (s, 3H), 1.44 (s, 6H). ^13^C­{^1^H} (CDCl_3_, 100 MHz): δ 176.4, 148.8, 139.1, 137.8, 132.5, 129.6, 126.2, 122.2, 121.1, 52.5, 44.8, 25.2. HRMS (DART/TOF): *m*/*z* [M + H]^+^ Calcd. for C_13_H_16_NO_4_^+^: 250.1079; found: 250.1084.

##### Methyl 2,2-Dimethyl-4-nitro-3-(3-nitrophenyl)­butanoate (**7g**)

Purification by flash column chromatography on silica gel using hexane/ethyl acetate (9:1) followed by a second column using a gradient elution of hexane/dichloromethane (9:1 to 7:3). Physical state: colorless oil. Isolated yield: 33% (19.3 mg) from conditions **B**. ^1^H NMR (CDCl_3_, 400 MHz): δ 8.17 (dt, *J* = 7.1, 2.2 Hz, 1H), 8.11–8.05 (m, 1H), 7.57–7.49 (m, 2H), 5.01 (dd, *J* = 13.5, 11.3 Hz, 1H), 4.86 (dd, *J* = 13.5, 3.9 Hz, 1H), 3.88 (dd, *J* = 11.3, 3.9 Hz, 1H), 3.73 (s, 3H), 1.26 (s, 3H), 1.19 (s, 3H). ^13^C­{^1^H} (CDCl_3_, 100 MHz): δ 175.8, 148.4, 138.7, 135.4, 129.7, 123.7, 123.4, 76.7, 52.7, 51.4, 45.6, 25.0, 23.0. HRMS (DART/TOF): *m*/*z* [M + H]^+^ Calcd. for C_13_H_17_N_2_O_6_^+^: 297.1087; found: 297.1075.

##### Methyl (*E*)-4-(3-Cyanophenyl)-2,2-dimethylbut-3-enoate (**6h**)

Purification by flash column chromatography on silica gel using hexane/ethyl acetate (95:5). Physical state: colorless oil. Isolated yield: 7% (3 mg) from conditions **C**. ^1^H NMR (CDCl_3_, 400 MHz): δ 7.69–7.62 (m, 1H), 7.59 (dt, *J* = 7.9, 1.6 Hz, 1H), 7.50 (dt, *J* = 7.7, 1.4 Hz, 1H), 7.41 (t, *J* = 7.8 Hz, 1H), 6.48 (d, *J* = 16.2 Hz, 1H), 6.40 (d, *J* = 16.2 Hz, 1H), 3.71 (s, 3H), 1.42 (s, 6H). ^13^C­{^1^H} (CDCl_3_, 100 MHz): δ 176.5, 138.5, 137.3, 130.8, 130.7, 130.0, 129.5, 126.2, 77.4, 52.5, 44.8, 29.5, 25.1. HRMS (DART/TOF): *m*/*z* [M + H]^+^ Calcd. for C_14_H_16_NO_2_^+^: 230.1181; found: 230.1175.

##### Methyl 3-(3-Cyanophenyl)-2,2-dimethyl-4-nitrobutanoate (**7h**)

Purification by flash column chromatography on silica gel using hexane/ethyl acetate (8:2) followed by a second column using a gradient elution of hexane/dichloromethane (9:1 to 7:3). Physical state: pale yellow oil. Isolated yield: 26% (14.4 mg) from conditions **B**. ^1^H NMR (CDCl_3_, 400 MHz): δ 7.60 (ddd, *J* = 5.4, 3.8, 1.6 Hz, 1H), 7.49 (m, 1H), 7.48–7.41 (m, 2H), 4.95 (dd, *J* = 13.4, 11.3 Hz, 1H), 4.81 (dd, *J* = 13.4, 4.0 Hz, 1H), 3.79 (dd, *J* = 11.3, 4.0 Hz, 1H), 3.71 (s, 3H), 1.23 (s, 3H), 1.17 (s, 3H). ^13^C­{^1^H} (CDCl_3_, 100 MHz): δ 175.9, 138.1, 133.5, 132.7, 132.0, 129.6, 118.5, 113.1, 76.7, 52.6, 51.3, 45.5, 25.1, 22.8. HRMS (DART/TOF): *m*/*z* [M + H]^+^ Calcd. for C_14_H_17_N_2_O_4_^+^: 277.1188; found: 277.1199.

##### Methyl (*E*)-2,2-Dimethyl-4-(4-nitrophenyl)­but-3-enoate (**6i**)

Purification by flash column chromatography on silica gel using hexane/ethyl acetate (8:2). Physical state: yellow oil. Isolated yield: 10% (5 mg) from conditions **A**. ^1^H NMR (CDCl_3_, 300 MHz): δ 8.17 (d, *J* = 8.9 Hz, 2H), 7.51 (d, *J* = 8.9 Hz, 2H), 6.61 (d, *J* = 16.2 Hz, 1H), 6.49 (d, *J* = 16.2 Hz, 1H), 3.72 (s, 3H), 1.44 (s, 6H). ^13^C­{^1^H} (CDCl_3_, 75 MHz): δ 176.3, 143.8, 139.4, 127.1, 126.5, 124.1, 52.5, 45.0, 25.1. HRMS (EI): *m*/*z* [M]^+^ Calcd. for C_13_H_15_NO_4_^+^: 249.1001; found: 249.1000.

##### Methyl 2,2-Dimethyl-4-nitro-3-(4-nitrophenyl)­butanoate (**7i**)

Purification by flash column chromatography on silica gel using hexane/ethyl acetate (8:2) followed by a second column using a gradient elution of hexane/dichloromethane (10:0 to 6:4). Physical state: yellow oil. Isolated yield: 45% (26.5 mg) from conditions **B**. ^1^H NMR (CDCl_3_, 300 MHz): δ 8.18 (d, *J* = 8.8 Hz, 2H), 7.38 (d, *J* = 8.8 Hz, 2H), 5.00 (dd, *J* = 13.5, 11.2 Hz, 1H), 4.84 (dd, *J* = 13.5, 4.0 Hz, 1H), 3.88 (dd, *J* = 11.3, 4.0 Hz, 1H), 3.70 (s, 3H), 1.24 (s, 3H), 1.18 (s, 3H). ^13^C­{^1^H} (CDCl_3_, 75 MHz): δ 175.8, 147.8, 144.0, 130.1, 123.8, 76.7, 52.6, 51.4, 45.6, 25.1, 22.9. HRMS (DART/TOF): *m*/*z* [M + H]^+^ Calcd. for C_13_H_17_N_2_O_6_^+^: 297.1087; found: 297.1079.

##### Methyl (*E*)-4-(4-Cyanophenyl)-2,2-dimethylbut-3-enoate (**6j**)

Purification by flash column chromatography on silica gel using hexane/ethyl acetate (8:2). Physical state: colorless oil. Isolated yield: 16% (7.2 mg) from conditions **A**. ^1^H NMR (CDCl_3_, 400 MHz): δ 7.59 (d, *J* = 8.6 Hz, 2H), 7.45 (d, *J* = 8.1 Hz, 2H), 6.55 (d, *J* = 16.2 Hz, 1H), 6.43 (d, *J* = 16.2 Hz, 1H), 3.71 (s, 3H), 1.42 (s, 6H). ^13^C­{^1^H} (CDCl_3_, 100 MHz): δ 176.4, 141.8, 138.5, 132.5, 127.0, 126.8, 119.1, 110.8, 52.5, 44.9, 25.1. HRMS (DART/TOF): *m*/*z* [M + H]^+^ Calcd. for C_14_H_16_NO_2_^+^: 230.1181; found: 230.1174.

##### Methyl 3-(4-Cyanophenyl)-2,2-dimethyl-4-nitrobutanoate (**7j**)

Purification by flash column chromatography on silica gel using hexane/ethyl acetate (8:2) followed by a second column using a gradient elution of hexane/dichloromethane (9:1 to 7:3). Physical state: colorless oil. Isolated yield: 28% (15.5 mg) from conditions **B**. ^1^H NMR (CDCl_3_, 400 MHz): δ 7.62 (d, *J* = 8.6 Hz, 2H), 7.32 (d, *J* = 8.2 Hz, 2H), 4.97 (dd, *J* = 13.5, 11.3 Hz, 1H), 4.82 (dd, *J* = 13.4, 3.9 Hz, 1H), 3.82 (dd, *J* = 11.3, 4.0 Hz, 1H), 3.70 (s, 3H), 1.23 (s, 3H), 1.16 (s, 3H). ^13^C­{^1^H} (CDCl_3_, 100 MHz): δ 175.9, 141.9, 132.4, 130.0, 118.4, 112.4, 76.7, 52.6, 51.6, 45.6, 25.2, 22.8. HRMS (DART/TOF): *m*/*z* [M + H]^+^ Calcd. for C_14_H_17_N_2_O_4_^+^: 277.1188; found: 277.1182.

##### Methyl 3-(4-Chlorophenyl)-2,2-dimethyl-4-nitrobutanoate (**7k**)

Purification by flash column chromatography on silica gel using hexane/ethyl acetate (95:5) followed by a second column using a gradient elution of hexane/dichloromethane (9:1 to 6:4). Physical state: pale yellow oil. Isolated yield: 22% (12.5 mg) from conditions **B**. ^1^H NMR (400 MHz, CDCl_3_): δ 7.29 (d, *J* = 8.5 Hz, 2H), 7.11 (d, *J* = 8.5 Hz, 2H), 4.92 (dd, *J* = 13.1, 11.3 Hz, 1H), 4.77 (dd, *J* = 13.2, 4.1 Hz, 1H), 3.75 (dd, *J* = 11.3, 4.1 Hz, 1H), 3.70 (s, 3H), 1.21 (s, 3H), 1.16 (s, 3H). ^13^C­{^1^H} (CDCl_3_, 100 MHz): δ 176.3, 134.7, 134.2, 130.4, 128.9, 77.1, 52.5, 51.0, 45.6, 25.2, 22.6. HRMS (DART/TOF): *m*/*z* [M + H]^+^ Calcd. for C_13_H_17_ClNO_4_^+^: 286.0846; found: 286.0850.

##### Methyl 3-(4-Bromophenyl)-2,2-dimethyl-4-nitrobutanoate (**7l**)

Purification by two DCVC’s, the first one using a solvent gradient from 98:2 to 86:14 hexane/AcOEt. The second one using a solvent gradient from 55:45 to 52:48 hexane/DCM. Physical state: yellow oil. Isolated yield: 18% (12.1 mg) from conditions **B**. ^1^H NMR (400 MHz, CDCl_3_): δ 7.45 (d, *J* = 8.5 Hz, 2H), 7.05 (d, *J* = 8.5 Hz, 2H), 4.92 (dd, *J* = 13.2, 11.3 Hz, 1H), 4.77 (dd, *J* = 13.2, 4.1 Hz, 1H), 3.73 (dd, *J* = 11.3, 4.1 Hz, 1H), 3.70 (s, 3H), 1.21 (s, 3H), 1.16 (s, 3H). ^13^C­{^1^H} (CDCl_3_, 100 MHz): δ 176.3, 135.3, 131.9, 130.8, 122.4, 77.0, 52.5, 51.1, 45.5, 25.2, 22.6. HRMS (DART/TOF): [M + H]^+^ Calcd. for C_13_H_17_BrNO_4_^+^: 330.0341; found: 330.0350.

##### Methyl 2,2-Dimethyl-4-nitro-3-(4-(phenylethynyl)­phenyl)­butanoate (**7m**)

Purification by two DCVC’s, the first one using a solvent gradient from 95:5 to 85:15 hexane/AcOEt. The second one using a solvent gradient from 60:40 to 45:55 hexane/DCM. Physical state: yellow oil. Isolated yield: 21% (13.6 mg) from conditions **A**. ^1^H NMR (CDCl_3_, 300 MHz) δ 7.54–7.46 (m, 4H), 7.37–7.32 (m, 3H), 7.16 (d, *J* = 8.4 Hz, 2H), 4.96 (dd, *J* = 13.2, 11.1 Hz, 1H), 4.79 (dd, *J* = 13.2, 4.1 Hz, 1H), 3.79 (dd, *J* = 11.1, 4.1 Hz, 1H), 3.70 (s, 3H), 1.22 (s, 3H), 1.18 (s, 3H). ^13^C­{^1^H} NMR (CDCl_3_, 75 MHz): δ 176.3, 136.4, 131.8, 131.8, 130.6, 129.2, 128.5, 124.5, 123.3, 90.3, 88.8, 77.0, 52.4, 51.4, 45.6, 25.2, 22.5. HRMS (DART/TOF): [M + H]^+^ Calcd. for C_21_H_22_NO_4_^+^: 352.1549; found: 352.1559.

##### Methyl (*E*)-4-(4-Methoxy-3,3-dimethyl-4-oxobut-1-en-1-yl)­benzoate (**6n**)

Purification by flash column chromatography on silica gel using hexane/ethyl acetate (8:2). Physical state: yellow oil. Isolated yield: 20% (10.3 mg) from conditions **A**. ^1^H NMR (CDCl_3_, 400 MHz): δ 7.98 (d, *J* = 8.3 Hz, 2H), 7.43 (d, *J* = 8.3 Hz, 2H), 6.53 (d, *J* = 16.2 Hz, 1H), 6.46 (d, *J* = 16.3 Hz, 1H), 3.91 (s, 3H), 3.71 (s, 3H), 1.42 (s, 6H). ^13^C­{^1^H} NMR (CDCl_3_, 100 MHz): δ 176.6, 167.1, 141.7, 137.2, 130.1, 129.0, 127.4, 126.4, 52.4, 52.2, 44.8, 25.2. HRMS (DART/TOF): *m*/*z* [M + H]^+^ Calcd. for C_15_H_19_O_4_^+^: 263.1283; found: 263.1289.

##### Methyl 4-(4-Methoxy-3,3-dimethyl-1-nitro-4-oxobutan-2-yl)­benzoate (**7n**)

Purification by flash column chromatography on silica gel using hexane/ethyl acetate (98:2) followed by a second column using a gradient elution of hexane/acetone (100:0 to 95:5). Physical state: colorless oil. Isolated yield: 27% (17 mg) from conditions **B**. ^1^H NMR (CDCl_3_, 400 MHz): δ 7.98 (d, *J* = 8.4 Hz, 2H), 7.26 (d, *J* = 8.3 Hz, 2H), 4.97 (dd, *J* = 13.3, 11.2 Hz, 1H), 4.81 (dd, *J* = 13.3, 4.0 Hz, 1H), 3.90 (s, 3H), 3.83 (dd, *J* = 11.2, 4.1 Hz, 1H), 3.69 (s, 3H), 1.21 (s, 3H), 1.17 (s, 3H). ^13^C­{^1^H} NMR (CDCl_3_, 100 MHz): δ 176.2, 166.7, 141.5, 130.1, 129.9, 129.2, 76.9, 52.5, 52.3, 51.4, 45.6, 25.2, 22.6. HRMS (DART/TOF): *m*/*z* [M + H]^+^ Calcd. for C_15_H_20_NO_6_^+^: 310.1291; found: 310.1292.

##### Methyl 2,2-Dimethyl-4-nitro-3-phenylbutanoate (**7o**)

Purification by flash column chromatography on silica gel using hexane/ethyl acetate (8:2) followed by a second column using a gradient elution of hexane/dichloromethane (9:1 to 7:3). Physical state: yellow oil. Isolated yield: 28% (14 mg) from conditions **B**. ^1^H NMR (CDCl_3_, 400 MHz): δ 7.33–7.27 (m, 3H), 7.16 (dd, *J* = 7.7, 1.8 Hz, 2H), 4.95 (dd, *J* = 13.1, 11.1 Hz, 1H), 4.78 (dd, *J* = 13.1, 4.1 Hz, 1H), 3.77 (dd, *J* = 11.1, 4.1 Hz, 1H), 3.70 (s, 3H), 1.21 (s, 3H), 1.17 (s, 3H). ^13^C­{^1^H} NMR (CDCl_3_, 100 MHz): δ 176.6, 136.2, 129.1, 128.7, 128.2, 77.3, 52.4, 51.5, 45.6, 25.2, 22.4. HRMS (DART/TOF): *m*/*z* [M + H]^+^ Calcd. for C_13_H_18_NO_4_^+^: 252.1236; found: 252.1250.

##### Methyl 3-(4-Methoxyphenyl)-2,2-dimethyl-4-nitrobutanoate (**7p**)

Purification by flash column chromatography on silica gel using hexane/ethyl acetate (8:2) followed by a second column using a gradient elution of hexane/dichloromethane (9:1 to 7:3). Physical state: colorless oil. Isolated yield: 40% (22.9 mg) from conditions **B**. ^1^H NMR (CDCl_3_, 400 MHz): δ 7.08 (d, *J* = 8.7 Hz, 2H), 6.83 (d, *J* = 8.8 Hz, 2H), 4.90 (dd, *J* = 12.9, 11.3 Hz, 1H), 4.75 (dd, *J* = 12.9, 4.2 Hz, 1H), 3.78 (s, 3H), 3.72 (dd, *J* = 6.1, 5.1 Hz, 1H), 3.69 (s, 3H), 1.19 (s, 3H), 1.16 (s, 3H). ^13^C­{^1^H} NMR (CDCl_3_, 100 MHz): δ 176.6, 159.4, 130.1, 128.0, 114.0, 77.4, 55.3, 52.3, 50.9, 45.7, 25.1, 22.4. HRMS (DART/TOF): *m*/*z* [M + H]^+^ Calcd. for C_14_H_20_NO_5_^+^: 282.1342; found: 282.1340.

##### Methyl (*E*)-2,2-Dimethyl-3-(nitromethyl)-5-phenylpent-4-enoate (**7q**)

Purification by two DCVC’s, the first one using a solvent gradient from 1:0 to 94:6 hexane/AcOEt. The second one using a solvent gradient from 60:40 to 52:48 hexane/DCM. Physical state: yellow oil. Isolated yield: 22% (12.0 mg) from conditions **B**. ^1^H NMR (CDCl_3_, 400 MHz): δ 7.35–7.23 (m, 5H), 6.50 (d, *J* = 15.7 Hz, 1H), 6.00 (dd, *J* = 15.7, 9.8 Hz, 1H), 4.57 (dd, *J* = 11.9, 3.9 Hz, 1H), 4.49 (dd, *J* = 12.0, 10.6 Hz, 1H), 3.72 (s, 3H), 3.27 (td, *J* = 10.2, 3.9 Hz, 1H), 1.28 (s, 3H), 1.25 (s, 3H). ^13^C­{^1^H} NMR (CDCl_3_, 100 MHz): δ 176.3, 136.4, 136.2, 128.7, 128.2, 126.7, 123.9, 77.5, 52.4, 50.1, 44.8, 24.5, 22.6. HRMS (DART/TOF): [M + H]^+^ Calcd. for C_15_H_20_NO_4_^+^: 278.1392; found: 278.1396.

##### Methyl (*E*)-2,2-Dimethyl-4-(2,3,4,5,6-pentafluorophenyl)­but-3-enoate (**6r**)

Purification by two DCVCs first one using a solvent gradient from 100:0 to 92:8 hexane/AcOEt. The second one using a solvent gradient from 80:20 to 57:43 hexane/DCM. Physical state: yellow oil. Isolated yield: 0.4% (2.5 mg) from conditions **B**. ^1^H NMR (CDCl_3_, 400 MHz): δ 6.76 (d, *J* = 16.7 Hz, 1H), 6.32 (d, *J* = 16.7 Hz, 1H), 3.72 (s, 3H), 1.43 (s, 6H). ^13^C­{^1^H} NMR (CDCl_3_, 100 MHz): δ 176.1, 143.7 (t, *J* = 8.8 Hz), 142.5 (t, *J* = 11.0 Hz), 123.2, 112.9, 52.5, 45.6, 24.8. HRMS (DART/TOF): [M + H]^+^ Calcd. for C_13_H_12_F_5_O_2_+: 295.0752; found: 295.0740.

##### Methyl 2,2-Dimethyl-4-nitro-3-(2,3,4,5,6-pentafluorophenyl)­butanoate (**7r**)

Purification by two DCVCs first one using a solvent gradient from 1:0 to 92:8 hexane/AcOEt. The second one using a solvent gradient from 80:20 to 57:43 hexane/DCM. Physical state: yellow oil. Isolated yield: 0.2% (1.3 mg) from conditions **A**. ^1^H NMR (CDCl_3_, 400 MHz): δ 5.01 (dd, *J* = 13.9, 10.9 Hz, 1H), 4.87 (dd, *J* = 14.0, 4.4 Hz, 1H), 4.27 (dd, *J* = 10.9, 4.3 Hz, 1H), 3.75 (s, 3H), 1.32 (s, 3H), 1.21 (s, 3H). ^13^C­{^1^H} NMR (CDCl_3_, 100 MHz): δ 175.0, 139.2, 74.5, 52.9, 45.4, 42.3, 25.4, 22.6. HRMS (DART/TOF): [M + H]^+^ Calcd. for C_13_H_13_F_5_NO_4_^+^: 342.0759; found: 342.0746.

##### Methyl (*E*)-2,2-Dimethyl-4-(pyridin-4-yl)­but-3-enoate (**6s**)

Purification by flash column chromatography on silica gel using hexane/ethyl acetate (8:2) followed by a second column using a gradient elution of hexane/dichloromethane (9:1 to 7:3). Physical state: colorless oil. Isolated yield: 2% (2.3 mg) from conditions **A** (mixed with product **7s**). ^1^H NMR (CDCl_3_, 400 MHz): δ 8.56–8.51 (m, 2H), 7.26–7.23 (m, 2H), 6.64 (d, *J* = 16.2 Hz, 1H), 6.37 (d, *J* = 16.2 Hz, 1H), 3.72 (s, 3H), 1.43 (s, 6H). ^13^C­{^1^H} NMR (CDCl_3_, 100 MHz): δ 171.3, 148.8, 140.7, 125.8, 52.5, 45.0, 25.0. HRMS (DART/TOF): [M + H]^+^ Calcd. for C_12_H_16_NO_2_^+^: 206.1181; found: 206.1174.

##### Methyl 2,2-Dimethyl-4-nitro-3-(pyridin-4-yl)­butanoate (**7s**)

Purification by DCVC on silica gel using a gradient elution of hexane/ethyl acetate (55:45 to 45:55) followed by a second DCVC using a gradient elution of dichloromethane/ethyl acetate (100:0 to 80:20). Physical state: colorless oil. Isolated yield: 5% (5.1 mg) from conditions **B**. ^1^H NMR (CDCl_3_, 400 MHz): δ 8.62–8.53 (m, 2H), 7.15–7.09 (m, 2H), 4.97 (dd, *J* = 13.5, 11.1 Hz, 1H), 4.81 (dd, *J* = 13.6, 3.9 Hz, 1H), 3.76 (dd, *J* = 11.2, 3.9 Hz, 1H), 3.71 (s, 3H), 1.24 (s, 3H), 1.18 (s, 3H). ^13^C­{^1^H} NMR (CDCl_3_, 100 MHz): δ 175.9, 150.2, 145.6, 124.3, 76.4, 52.6, 50.9, 45.3, 25.2, 22.6. HRMS (DART/TOF): [M + H]^+^ Calcd. for C_12_H_17_N_2_O_2_^+^: 253.1183; found: 253.1192.

##### Methyl 3-(3,4-Dimethoxyphenyl)-2,2-dimethyl-4-nitrobutanoate (**7t**)

Purification by flash column chromatography on silica gel using hexane/ethyl acetate (8:2) followed by a second column using a gradient elution of hexane/dichloromethane (9:1 to 7:3). Physical state: colorless oil. Isolated yield: 27% (16.7 mg) from conditions **B**. ^1^H NMR (CDCl_3_, 400 MHz): δ 6.80 (d, *J* = 8.2 Hz, 1H), 6.71 (d, *J* = 8.3 Hz, 1H), 6.67 (s, 1H), 4.93 (ddd, *J* = 12.7, 11.2, 1.3 Hz, 1H), 4.77 (dd, *J* = 12.9, 5.5 Hz, 1H), 3.86 (s, 6H), 3.70 (s, 3H), 1.22 (s, 3H), 1.18 (s, 3H). ^13^C­{^1^H} NMR (CDCl_3_, 100 MHz): δ 176.5, 148.7, 148.7, 128.4, 121.0, 112.4, 111.0, 77.3, 55.9, 55.8, 52.2, 51.2, 45.6, 25.1, 22.5. HRMS (DART/TOF): *m*/*z* [M + H]^+^ Calcd. for C_15_H_22_NO_6_^+^: 312.1447, Found: 312.1448.

##### Methyl (*E*)-4-(2-Ethynylphenyl)-2,2-dimethylbut-3-enoate (**6u**)

Purification by flash column chromatography on silica gel using hexane/ethyl acetate (85:15). Physical state: yellow oil. Isolated yield: 32% (14.5 mg) from conditions **A**. ^1^H NMR (CDCl_3_, 400 MHz): δ 7.55 (dd, *J* = 8.0, 1.5 Hz, 1H), 7.47 (dd, *J* = 7.7, 1.5 Hz, 1H), 7.30 (td, *J* = 7.5, 1.4 Hz, 1H), 7.18 (td, *J* = 7.6, 1.3 Hz, 1H), 6.96 (d, *J* = 16.3 Hz, 1H), 6.48 (d, *J* = 16.3 Hz, 1H), 3.71 (s, 3H), 3.32 (s, 1H), 1.44 (s, 6H). ^13^C­{^1^H} NMR (CDCl_3_, 100 MHz): δ 176.9, 139.3, 136.5, 133.3, 129.1, 127.2, 126.1, 124.9, 120.9, 82.1, 81.9, 52.3, 44.9, 25.2. HRMS (DART/TOF): *m*/*z* [M + H]^+^ Calcd. for C_15_H_17_O_2_^+^: 229.1228; found: 229.1220.

##### Methyl 3-(2-Ethynylphenyl)-2,2-dimethyl-4-nitrobutanoate (**7u**)

Purification by flash column chromatography on silica gel using hexane/acetone (95:5) followed by a second column using a gradient elution of hexane/dichloromethane (9:1 to 6:4). Physical state: colorless oil. Isolated yield: 25% (13.9 mg) from conditions **B**. ^1^H NMR (CDCl_3_, 400 MHz): δ 7.52 (d, *J* = 7.5 Hz, 1H), 7.33 (t, *J* = 7.6 Hz, 1H), 7.24 (t, *J* = 7.0 Hz, 1H), 7.19 (d, *J* = 7.9 Hz, 1H), 4.95 (t, *J* = 11.9 Hz, 1H), 4.86 (dd, *J* = 12.8, 4.6 Hz, 1H), 4.60 (dd, *J* = 10.9, 4.4 Hz, 1H), 3.72 (s, 3H), 3.35 (s, 1H), 1.30 (s, 3H), 1.18 (s, 3H). ^13^C­{^1^H} NMR (CDCl_3_, 100 MHz): δ 176.6, 139.1, 133.8, 129.1, 127.8, 82.3, 82.1, 77.5, 52.4, 47.6, 46.4, 25.7, 22.1. HRMS (DART/TOF): *m*/*z* [M + H]^+^ Calcd. for C_15_H_18_NO_4_^+^: 276.1230; found: 276.1226.

##### Methyl (*E*)-4-(2,4-Dinitrophenyl)-2,2-dimethylbut-3-enoate (**6v**)

Purification by flash column chromatography on silica gel using hexane/ethyl acetate (95:5) followed by a second column using a gradient elution of hexane/dichloromethane (9:1 to 7:3). Physical state: colorless oil. Isolated yield: 22% (13 mg) from conditions **B**. ^1^H NMR (CDCl_3_, 400 MHz): δ 8.80 (d, *J* = 2.5 Hz, 1H), 8.39 (dd, *J* = 8.6, 2.3 Hz, 1H), 7.81 (d, *J* = 8.7 Hz, 1H), 6.98 (d, *J* = 16.1 Hz, 1H), 6.62 (d, *J* = 16.1 Hz, 1H), 3.74 (s, 3H), 1.46 (s, 6H). ^13^C­{^1^H} NMR (CDCl_3_, 100 MHz): δ 175.9, 144.1, 139.0, 130.0, 127.2, 122.5, 120.6, 52.6, 45.4, 25.0. HRMS (DART/TOF): *m*/*z* [M + H]^+^ Calcd. for C_13_H_15_N_2_O_6_^+^: 295.0930; found: 295.0925.

##### Methyl 3-(2,4-Dinitrophenyl)-2,2-dimethyl-4-nitrobutanoate (**7v**)

Purification by flash column chromatography on silica gel using hexane/ethyl acetate (95:5). Physical state: colorless oil. Isolated yield: 18% (12.4 mg) from conditions **B**. ^1^H NMR (CDCl_3_, 400 MHz): δ 8.69 (d, *J* = 2.4 Hz, 1H), 8.42 (dd, *J* = 8.7, 2.4 Hz, 1H), 7.73 (d, *J* = 8.8 Hz, 1H), 5.09–4.94 (m, 2H), 4.67 (dd, *J* = 10.9, 3.9 Hz, 1H), 3.74 (s, 3H), 1.39 (s, 3H), 1.20 (s, 3H). ^13^C­{^1^H} NMR (CDCl_3_, 100 MHz): δ 175.7, 151.7, 147.3, 139.1, 130.1, 126.8, 120.7, 77.0, 52.9, 46.3, 44.3, 25.8, 23.4. HRMS (DART/TOF): *m*/*z* [M + H]^+^ Calcd. for C_13_H_16_N_3_O_8_^+^: 342.0937; found: 342.0928.

##### Methyl 2,2-Dimethyl-3-(nitromethyl)-5-phenylpentanoate (**7w**)

Purification by flash column chromatography on silica gel using hexane/ethyl acetate (95:5). Physical state: colorless oil.

Isolated yield: 46% (25.8 mg) from conditions **A**. ^1^H NMR (CDCl_3_, 400 MHz): δ 7.32 (dd, *J* = 13.3, 5.9 Hz, 3H), 7.19 (d, *J* = 6.7 Hz, 2H), 4.63 (dd, *J* = 13.2, 5.1 Hz, 1H), 4.39 (dd, *J* = 13.3, 6.5 Hz, 1H), 3.68 (s, 3H), 2.78–2.58 (m, 3H), 1.80 (tdd, *J* = 10.1, 7.0, 2.9 Hz, 1H), 1.64 (td, *J* = 9.6, 5.1 Hz, 1H), 1.22 (s, 6H). ^13^C­{^1^H} NMR (CDCl_3_, 100 MHz): δ 176.8, 141.2, 128.7, 128.5, 126.4, 77.6, 52.2, 45.6, 44.2, 34.4, 32.4, 23.3, 22.5. HRMS (DART/TOF): *m*/*z* [M+NH_4_]^+^ Calcd. for C_15_H_25_N_2_O_4_^+^: 297.1814; found: 297.1808.

##### Methyl 2,2-Dimethyl-4-nitro-3-phenylpentanoate (**7x**)

Purification by flash column chromatography on silica gel using hexane/ethyl acetate (9:1) followed by a second column using a gradient elution of hexane/dichloromethane (9:1 to 7:3). Physical state: colorless oil. Isolated yield: 22% (11.6 mg) from conditions **B**. ^1^H NMR (CDCl_3_, 400 MHz): δ 7.35–7.28 (m, 3H), 7.13 (dd, *J* = 7.8, 2.0 Hz, 2H), 5.27 (dq, *J* = 10.2, 6.7 Hz, 1H), 3.68 (d, *J* = 10.2 Hz, 1H), 3.61 (s, 3H), 1.27 (s, 3H), 1.24 (d, *J* = 6.6 Hz, 3H), 1.08 (s, 3H). ^13^C­{^1^H} NMR (CDCl_3_, 100 MHz): δ 176.8, 136.9, 129.8, 128.8, 128.0, 83.8, 56.4, 52.1, 45.8, 25.3, 22.1, 20.9. HRMS (DART/TOF): *m*/*z* [M + H]^+^ Calcd. for C_14_H_20_NO_4_^+^: 266.1392; found: 266.1380.

##### Methyl (*E*)-2,2,3-Trimethyl-4-(2-nitrophenyl)­but-3-enoate (**6y**)

Purification by flash column chromatography on silica gel using hexane/ethyl acetate (8:2). Physical state: yellow oil. Isolated yield: 9% (4.5 mg) from conditions **A**. ^1^H NMR (CDCl_3_, 400 MHz): δ 8.00 (dd, *J* = 8.2, 1.3 Hz, 1H), 7.56 (td, *J* = 7.5, 1.3 Hz, 1H), 7.40 (td, *J* = 7.8, 2.2 Hz, 1H), 7.32 (d, *J* = 7.6 Hz, 1H), 6.65 (s, 1H), 3.71 (s, 3H), 1.61 (d, *J* = 1.3 Hz, 3H), 1.44 (s, 6H). ^13^C­{^1^H} NMR (CDCl_3_, 100 MHz): δ 177.0, 142.6, 133.9, 132.8, 132.3, 127.7, 124.6, 121.1, 52.3, 48.9, 24.7, 15.4. HRMS (DART/TOF): *m*/*z* [M + H]^+^ Calcd. for C_14_H_18_NO_4_^+^: 264.1236; found: 264.1239.

##### Methyl 2,2-Dimethyl-4-nitro-3-(2-nitrophenyl)­pentanoate (**7y**)

Purification by flash column chromatography on silica gel using hexane/ethyl acetate (8:2). Physical state: pale yellow oil. Isolated yield: 50% (31 mg) from conditions **A**. ^1^H NMR (CDCl_3_, 400 MHz, diastereomer mixture, *d*.*r*. = 1:1): δ 7.75 (dd, *J* = 11.4, 8.3 Hz, 2H), 7.59–7.53 (m, 3H), 7.48–7.40 (m, 2H), 7.33 (dd, *J* = 7.9, 1.4 Hz, 1H), 5.39–5.31 (m, 1H), 5.20–5.10 (m, 1H), 4.75 (d, *J* = 11.0 Hz, 1H), 4.46 (d, *J* = 10.1 Hz, 1H), 3.78 (s, 3H), 3.60 (s, 3H), 1.60 (d, *J* = 6.6 Hz, 3H), 1.34 (d, *J* = 6.7 Hz, 3H), 1.27 (s, 3H), 1.19 (s, 3H), 1.16 (s, 3H), 1.11 (s, 3H). ^13^C­{^1^H} NMR (CDCl_3_, 100 MHz, diastereomer mixture): δ 177.3, 176.1, 152.3, 151.9, 132.4, 132.2, 131.4, 131.2, 129.1, 128.9, 128.9, 128.8, 125.2, 124.7, 86.9, 83.5, 52.7, 52.4, 48.3, 46.9, 46.3, 44.8, 27.1, 24.4, 23.8, 20.9, 20.4, 19.0. HRMS (DART/TOF): *m*/*z* [M + H]^+^ Calcd. for C_14_H_19_N_2_O_6_^+^: 311.1243; found: 311.1231.

##### Methyl 2,2,3-Trimethyl-4-(4-nitrophenyl)­but-3-enoate (**6z**)

Purification by flash column chromatography on silica gel using hexane/ethyl acetate (8:2). Physical state: colorless oil. Isolated yield: 30% (15.8 mg) from conditions **A**. ^1^H NMR (CDCl_3_, 400 MHz, *E*/*Z* mixture, 1:1): δ 8.18 (d, *J* = 8.8 Hz, 2H), 8.13 (d, *J* = 8.4 Hz, 2H), 7.39 (d, *J* = 8.2 Hz, 2H), 7.25 (d, *J* = 8.2 Hz, 2H), 6.46 (s, 1H), 6.42 (s, 1H), 3.70 (s, 3H), 3.16 (s, 3H), 1.96 (d, *J* = 1.5 Hz, 3H), 1.83 (d, *J* = 1.3 Hz, 3H), 1.44 (s, 6H), 1.33 (s, 6H). ^13^C­{^1^H} NMR (CDCl_3_, 100 MHz, *E*/*Z* mixture): δ 176.8, 176.6, 146.4, 146.2, 145.4, 145.3, 145.1, 143.2, 129.9, 129.8, 125.2, 123.5, 123.1, 122.9, 52.4, 51.7, 49.5, 46.8, 26.4, 24.8, 22.9, 16.1. HRMS (EI): *m*/*z* [M]^+^ Calcd. for C_14_H_17_NO_4_^+^: 263.1158; found: 263.1161.

##### Methyl 2,2-Dimethyl-4-nitro-3-(4-nitrophenyl)­pentanoate (**7z**)

Purification by flash column chromatography on silica gel using hexane/ethyl acetate (8:2) followed by a second column using a gradient elution of hexane/dichloromethane (9:1 to 7:3). Physical state: white solid, m.p.: 92–93 °C. Isolated yield: 28% (17.3) mg, minor diastereomer and 31% (19.5 mg) major diastereomer from conditions **B**. ^1^H NMR (CDCl_3_, 400 MHz, minor diastereomer, *d*.*r*. = 1.1:1): δ 8.20 (d, *J* = 8.8 Hz, 2H), 7.36 (d, *J* = 8.8 Hz, 2H), 5.36–5.28 (m, 1H), 3.83 (d, *J* = 9.7 Hz, 1H), 3.63 (s, 3H), 1.25 (s, 6H), 1.13 (s, 3H). ^13^C­{^1^H} NMR (CDCl_3_, 100 MHz, minor diastereomer): δ 176.1, 147.7, 144.5, 130.8, 123.9, 83.0, 56.2, 52.4, 45.9, 25.1, 22.8, 20.6. HRMS (DART/TOF): *m*/*z* [M + H]^+^ Calcd. for C_14_H_19_N_2_O_6_^+^: 311.12431; found: 311.12564. ^1^H NMR (CDCl_3_, 400 MHz, major diastereomer): δ 8.17 (d, *J* = 8.8 Hz, 2H), 7.43 (d, *J* = 8.7 Hz, 2H), 5.15 (dq, *J* = 11.3, 6.7 Hz, 1H), 4.04 (d, *J* = 11.3 Hz, 1H), 3.78 (s, 3H), 1.59 (d, *J* = 6.7 Hz, 3H), 1.26 (s, 3H), 0.91 (s, 3H). ^13^C­{^1^H} NMR (CDCl_3_, 100 MHz, major diastereomer): δ 177.3, 147.8, 144.2, 130.4, 123.7, 86.3, 54.5, 52.7, 44.0, 28.2, 19.2, 18.6. HRMS (EI): *m*/*z* [M]^+^ Calcd. for C_14_H_18_N_2_O_6_^+^: 310.1243; found: 310.1249.

##### Methyl (*E*)-2,2-Dimethyl-4-(4-nitrophenyl)­pent-3-enoate (**6za**)

Purification by flash column chromatography on silica gel using hexane/ethyl acetate (8:2). Physical state: colorless oil. Isolated yield: 14% (7.4 mg) from conditions **A**. ^1^H NMR (CDCl_3_, 400 MHz): δ 8.16 (d, *J* = 8.9 Hz, 2H), 7.50 (d, *J* = 9.1 Hz, 2H), 5.90 (q, *J* = 1.3 Hz, 1H), 3.74 (s, 3H), 1.96 (d, *J* = 1.3 Hz, 3H), 1.44 (s, 6H). ^13^C­{^1^H} NMR (CDCl_3_, 100 MHz): δ 177.7, 150.5, 146.9, 137.1, 135.3, 126.7, 123.7, 52.4, 43.5, 27.2, 16.4. HRMS (DART/TOF): *m*/*z* [M + H]^+^ Calcd. for C_14_H_18_NO_4_^+^: 264.1236; found: 264.1228.

##### Methyl 2,2,3-Trimethyl-4-nitro-3-(4-nitrophenyl)­butanoate (**7za**)

Purification by flash column chromatography on silica gel using hexane/ethyl acetate (8:2). Physical state: colorless oil.

Isolated yield: 19% (11.6 mg) from conditions **C** (It was not possible to obtain a clean sample under procedure B). ^1^H NMR (CDCl_3_, 400 MHz): δ 8.19 (d, *J* = 9.1 Hz, 2H), 7.49 (d, *J* = 9.0 Hz, 2H), 5.49 (d, *J* = 12.8 Hz, 1H), 4.97 (d, *J* = 12.8 Hz, 1H), 3.64 (s, 3H), 1.68 (s, 3H), 1.14 (s, 3H), 1.07 (s, 3H). ^13^C­{^1^H} NMR (CDCl_3_, 100 MHz): δ 175.6, 147.1, 146.9, 128.8, 123.0, 81.4, 52.4, 49.6, 47.2, 22.3, 21.5, 20.0. HRMS (DART/TOF): *m*/*z* [M + H]^+^ Calcd. for C_14_H_19_N_2_O_6_^+^: 311.1243; found: 311.1251.

##### (*E*)-2,2-Dimethyl-4-(2-nitrophenyl)­but-3-enoic Acid (**8a**)

Purification by DCVC using a solvent gradient from 95:05:1 to 70:30:1 hexane/AcOEt/AcOH. Physical state: colorless oil. Isolated yield: 18% (8.5 mg) from conditions **B**. ^1^H NMR (CDCl_3_, 400 MHz): δ 11.24 (br, 1H), 7.92 (dd, *J* = 8.2, 1.3 Hz, 1H), 7.62–7.51 (m, 2H), 7.37 (ddd, *J* = 8.5, 7.1, 1.8 Hz, 1H), 6.97 (d, *J* = 16.0 Hz, 1H), 6.39 (d, *J* = 16.0 Hz, 1H), 1.46 (s, 6H). ^13^C­{^1^H} NMR (CDCl_3_, 100 MHz): δ 182.8, 147.9, 138.8, 133.3, 133.1, 129.0, 128.2, 124.7, 124.6, 44.8, 24.8. HRMS (DART/TOF): [M + H]^+^ Calcd. for C_12_H_14_NO_4_^+^: 236.0917; found: 236.0925.

##### 2,2-Dimethyl-4-nitro-3-(2-nitrophenyl)­butanoic Acid (**9a**)

Purification by DCVC using a solvent gradient from 95:05:1 to 70:30:1 hexane/AcOEt/AcOH. Physical state: colorless oil. Isolated yield: 29% (16.5 mg) from conditions **B**. ^1^H NMR (CDCl_3_, 400 MHz): δ 7.84 (dd, *J* = 8.2, 1.3 Hz, 1H), 7.67–7.57 (m, 1H), 7.54–7.43 (m, 2H), 5.06–4.91 (m, 2H), 4.75 (dd, *J* = 10.5, 4.4 Hz, 1H), 1.37 (s, 3H), 1.23 (s, 3H). ^13^C­{^1^H} NMR (CDCl_3_, 75 MHz): δ 180.8, 151.8, 132.9, 131.3, 129.2, 128.4, 125.4, 77.3, 45.8, 43.3, 25.6, 22.6. HRMS (DART/TOF): [M + H]^+^ Calcd. for C_12_H_15_N_2_O_6_^+^: 283.0925; found: 283.0934.

##### (*E*)-1-(2-Nitrostyryl)­cyclopentane-1-carboxylic Acid (**8b**)

Purification by DCVC using a solvent gradient from 95:05:1 to 65:35:1 hexane/AcOEt/AcOH. Physical state: colorless oil. Isolated yield: 19% (9.8 mg) from conditions **C**. ^1^H NMR (CDCl_3_, 400 MHz): δ 7.94 (dd, *J* = 8.2, 1.3 Hz, 1H), 7.65–7.50 (m, 2H), 7.39 (ddd, *J* = 8.5, 7.0, 1.8 Hz, 1H), 6.97 (d, *J* = 16.0 Hz, 1H), 6.40 (d, *J* = 16.0 Hz, 1H), 2.40–2.20 (m, 2H), 2.03–1.88 (m, 2H), 1.86–1.75 (m, 4H). ^13^C­{^1^H} NMR (CDCl_3_, 100 MHz): δ 181.6, 147.9, 137.3, 133.3, 133.2, 129.1, 128.2, 125.2, 124.7, 56.3, 36.0, 24.3. HRMS (DART/TOF): [M + H]^+^ Calcd. for C_14_H_16_NO_4_^+^: 262.1079; found: 262.1076.

##### 1-(2-Nitro-1-(2-nitrophenyl)­ethyl)­cyclopentane-1-carboxylic Acid (**9b**)

Purification by DCVC using a solvent gradient from 95:05:1 to 65:35:1 hexane/AcOEt/AcOH. Physical state: colorless oil. Isolated yield: 9% (5.7 mg) from conditions **C**. ^1^H NMR (CDCl_3_, 400 MHz): δ 7.83 (d, *J* = 8.1 Hz, 1H), 7.66–7.52 (m, 1H), 7.53–7.41 (m, 2H), 5.17–4.90 (m, 2H), 4.76–4.60 (m, 1H), 2.37–2.20 (m, 1H), 2.08–1.95 (m, 1H), 1.82–1.64 (m, 5H), 1.65–1.52 (m, 1H). ^13^C­{^1^H} NMR (CDCl_3_, 100 MHz): δ 179.9, 151.4, 133.1, 132.2, 129.0, 128.3, 125.3, 78.4, 57.9, 43.6, 37.7, 33.7, 24.5, 24.3. HRMS (DART/TOF): [M + H]^+^ Calcd. for C_14_H_17_N_2_O_6_^+^: 309.1087; found: 309.1079.

##### (*E*)-2-Ethyl-4-(2-nitrophenyl)­but-3-enoic Acid (**8e**)

Purification by DCVC using a solvent gradient from 95:05:1 to 65:35:1 hexane/AcOEt/AcOH. Physical state: yellow oil. Isolated yield: 17% (8.1 mg) from conditions **C**. ^1^H NMR (CDCl_3_, 400 MHz): δ 7.92 (dd, *J* = 8.2, 1.3 Hz, 1H), 7.60 (dd, *J* = 7.9, 1.6 Hz, 1H), 7.55 (td, *J* = 7.8, 1.3 Hz, 1H), 7.38 (ddd, *J* = 8.5, 7.2, 1.6 Hz, 1H), 6.99 (d, *J* = 15.7 Hz, 1H), 6.19 (dd, *J* = 15.7, 9.0 Hz, 1H), 3.19 (q, *J* = 7.7 Hz, 1H), 1.94 (dp, *J* = 14.3, 7.3 Hz, 1H), 1.73 (dt, *J* = 13.5, 7.4 Hz, 1H), 1.01 (t, *J* = 7.4 Hz, 3H). ^13^C­{^1^H} NMR (CDCl_3_, 100 MHz): δ 179.5, 147.9, 133.3, 132.7, 132.3, 129.0, 128.4, 128.4, 124.7, 50.9, 25.9, 11.7. HRMS (DART/TOF): [M + H]^+^ Calcd. for C_12_H_14_NO_4_^+^: 236.0923; found: 236.0914.

##### 2-Ethyl-4-nitro-3-(2-nitrophenyl)­butanoic Acid (**9e**)

Purification by DCVC using a solvent gradient from 95:05:1 to 65:35:1 hexane/AcOEt/AcOH. Physical state: yellow oil. Isolated yield: 21% (12.0 mg) from conditions **C**. ^1^H NMR (CDCl_3_, 400 MHz, diastereomer mixture, *d*.*r*. = 3:2): δ 7.91–7.85 (m, 1H), 7.59 (q, *J* = 7.8 Hz, 1H), 7.49–7.34 (m, 2H), 4.97–4.84 (m, 1.4H), 4.78 (dd, *J* = 13.7, 4.1 Hz, 0.6H), 4.47–4.29 (m, 1H), 3.03 (td, *J* = 9.6, 3.9 Hz, 0.4H), 2.91 (td, *J* = 9.3, 4.5 Hz, 0.6H), 1.90–1.80 (m, 0.4H), 1.77–1.59 (m, 1H), 1.48 (ddd, *J* = 13.4, 7.3, 4.9 Hz, 0.6H), 0.98 (t, *J* = 7.4 Hz, 1.2H), 0.91 (t, *J* = 7.4 Hz, 1.8H). ^13^C­{^1^H} NMR (CDCl_3_, 100 MHz, diastereomer mixture): δ 179.2, 178.6, 150.7, 150.1, 133.5, 133.3, 132.3, 132.1, 129.2, 125.5, 125.5, 77.3, 76.7, 49.6, 49.2, 24.2, 22.8, 11.5. HRMS (DART/TOF): [M + H]^+^ Calcd. for C_12_H_15_N_2_O_6_^+^: 283.0925; found: 283.0933.

##### 2-Methyl-2-(2-nitro-1-(2-nitrophenyl)­ethyl)­cyclopentan-1-one (**9i**)

Purification by DCVC using a solvent gradient from 85:15 to 67:33 hexane/AcOEt. Physical state: yellow oil. Isolated yield: 40% (21.6 mg), major diastereomer and 14% (8.2 mg), minor diastereomer from conditions **C**. ^1^H NMR (CDCl_3_, 300 MHz, major diastereomer, d.r. = 2.3:1): δ 7.83 (dd, *J* = 8.1, 1.5 Hz, 1H), 7.57 (td, *J* = 7.7, 1.5 Hz, 1H), 7.44 (ddd, *J* = 8.1, 7.4, 1.4 Hz, 1H), 7.38 (dd, *J* = 7.9, 1.4 Hz, 1H), 5.18 (dd, *J* = 14.0, 11.4 Hz, 1H), 4.89 (dd, *J* = 14.0, 3.9 Hz, 1H), 4.53 (dd, *J* = 11.4, 3.8 Hz, 1H), 2.47–2.32 (m, 1H), 2.13–1.78 (m, 5H), 1.21 (s, 3H). ^13^C­{^1^H} NMR (CDCl_3_, 75 MHz, major diastereomer) δ 222.0, 151.6, 133.2, 132.7, 128.9, 128.0, 125.2, 76.8, 51.8, 42.8, 38.8, 34.9, 22.2, 18.8. HRMS (DART/TOF): [M + H]^+^ Calcd. for C_14_H_17_N_2_O_5_^+^: 293.1137; found: 293.1130. ^1^H NMR (CDCl_3_, 300 MHz, minor diastereomer): δ 7.82 (dd, *J* = 8.1, 1.4 Hz, 1H), 7.73 (dd, *J* = 8.0, 1.4 Hz, 1H), 7.62 (td, *J* = 7.7, 1.4 Hz, 1H), 7.45 (ddd, *J* = 8.1, 7.3, 1.5 Hz, 1H), 5.18 (dd, *J* = 13.8, 4.0 Hz, 1H), 4.86 (dd, *J* = 13.7, 11.3 Hz, 1H), 4.53 (dd, *J* = 11.3, 4.0 Hz, 1H), 2.43–2.26 (m, 2H), 2.04–1.91 (m, 2H), 1.61 (dtd, *J* = 8.1, 4.2, 2.0 Hz, 2H), 1.10 (s, 3H). ^13^C­{^1^H} NMR (CDCl_3_, 75 MHz, minor diastereomer) δ 220.8, 132.7, 131.7, 129.5, 128.9, 125.3, 76.3, 50.0, 40.0, 37.7, 36.3, 19.0, 18.4. HRMS (DART/TOF): [M + H]^+^ Calcd. for C_14_H_17_N_2_O_5_^+^: 293.1137; found: 293.1145.

##### Methyl 2-Acetyl-4-nitro-3-(2-nitrophenyl)­butanoate (**9j**)

Purification by flash column chromatography on silica gel using hexane/ethyl acetate (8:2). Physical state: yellow oil. Isolated yield: 90% (56 mg) from conditions **C**. ^1^H NMR (CDCl_3_, 300 MHz, diastereomer mixture, *d*.*r*. = 1:1) δ 7.91 (ddd, *J* = 8.3, 6.1, 1.4 Hz, 2H), 7.57 (tdd, *J* = 7.6, 4.8, 1.4 Hz, 2H), 7.51–7.40 (m, 3H), 7.31 (dd, *J* = 7.9, 1.4 Hz, 1H), 5.17 (dd, *J* = 14.1, 8.6 Hz, 1H), 5.04–4.96 (m, 2H), 4.91 (dd, *J* = 13.1, 3.8 Hz, 1H), 4.75–4.63 (m, 2H), 4.50 (d, *J* = 9.6 Hz, 1H), 4.37 (d, *J* = 6.9 Hz, 1H), 3.76 (s, 3H), 3.55 (s, 3H), 2.36 (s, 3H), 2.27 (s, 3H). ^13^C­{^1^H} NMR (CDCl_3_, 75 MHz, diastereomer mixture) δ 200.9, 200.2, 168.0, 167.0, 150.1, 150.0, 133.5, 133.4, 132.0, 131.6, 129.4, 129.3, 129.1, 128.7, 125.7, 125.5, 76.8, 75.9, 60.8, 60.5, 53.2, 53.2, 37.2, 36.8, 30.9, 29.9. HRMS (DART/TOF): [M + H]^+^ Calcd. for C_13_H_15_N_2_O_7_^+^: 311.0879; found: 311.0868.

#### Scale-up, Conditions C

In a 25 mL flask with stirring bar was dissolved 195 mg (1.0 mmol, 1.0 equiv) of β-nitrostyrene **1b** in 10 mL of dry DCM, and the reaction mixture was cooled to −78 °C. The silylketene acetal **3′** 350 mg (2.0 mmol, 2.0 equiv) was added and later 2 mL of TBAF 1 M in THF (2.0 equiv, 2.0 mmol) were added. The reaction was stirred at −78 °C for 1 h. The resulting black solution was diluted with 7 mL of water and acidified to pH ∼ 4 using a 10% hydrochloric acid solution. The products were extracted with EtOAc (10 mL × 3). The combined organic layers were washed with brine, dried over sodium sulfate (Na_2_SO_4_), filtered, and concentrated under reduced pressure. The crude reaction mixture was purified by flash column chromatography using hexane/ethyl acetate (8:2) as eluent obtaining 149.6 mg (60% yield) of **6b** and 47.4 mg (16% yield) of **7b**. The spectra of the compounds is the same as the previous reactions.

## Computational Details

Density Functional Theory (DFT) calculations were carried out using M062X-D3 exchange-correlation functional combined with the 6-311++G­(d,p) basis set. Geometries were optimized and confirmed as energy minima through frequency calculations. Electrophilic Parr functions, P^+^(C_*k*_) donde *k* = α*y*β, were obtained through NBO atomic spin density (ASD) analysis of the radical anion and cation. These analyses were performed via single-point energy calculations on the optimized neutral geometries, employing the unrestricted UM062X-D3 formalism for radical species. All calculations were conducted using Gaussian 16.

## Supplementary Material



## Data Availability

The data underlying this study are available in the published article and its Supporting Information.

## Notes

Spectra of all compounds, Electrophilic Parr functions, frontier molecular orbital energies and computational details for the modeled nitroalkenes, Spectroscopic experiments (^1^H NMR, UV–vis, and EPR), and experimental procedure for starting materials **1** (PDF)
