# Supplementary material for: Competing Michael/Anti-Michael Addition of Silyl Ketene Acetals to β‑Nitrostyrenes Incorporating an Electron-Withdrawing Group
Source: J Org Chem. 2025 Sep 30;90(40):14232–45. doi: 10.1021/acs.joc.5c01852 (PMC12519467; doi:10.1021/acs.joc.5c01852)
Supplement: Supplementary file 1 [file jo5c01852_si_001.pdf]

# Supporting information

## Competing Michael/anti-Michael addition of silyl ketene acetals to β-nitrostyrenes incorporating an electron-withdrawing group

Mayte A. Martínez-Aguirre, Diego A. Cruz-Aguilar, Eduardo Hernández-Huerta, Dylan F. Lopez-Barba, Ricardo Ballinas-Indili, Saulo César Rosales-Amezcua, Cecilio Álvarez-Toledano, Marcos Hernández-Rodríguez*

**Table of Contents**

1. Spectroscopic experiments ($^1$NMR monitoring, $^1$HNMR of crude reaction mixture, UV-VIS, EPR, Figures S1-S6)……(S2)
2. Electrophilic Parr functions and frontier molecular orbital energies of nitroalkenes **1**…… (S5)
3. Experimental procedure of starting materials……(S6)
4. $^1$H NMR, $^{13}$C NMR spectra of products……(S10)
5. References……(S68)
6. XYZ coordinates and energies of the calculated structures……(S69)

## 1. **Spectroscopic experiments**

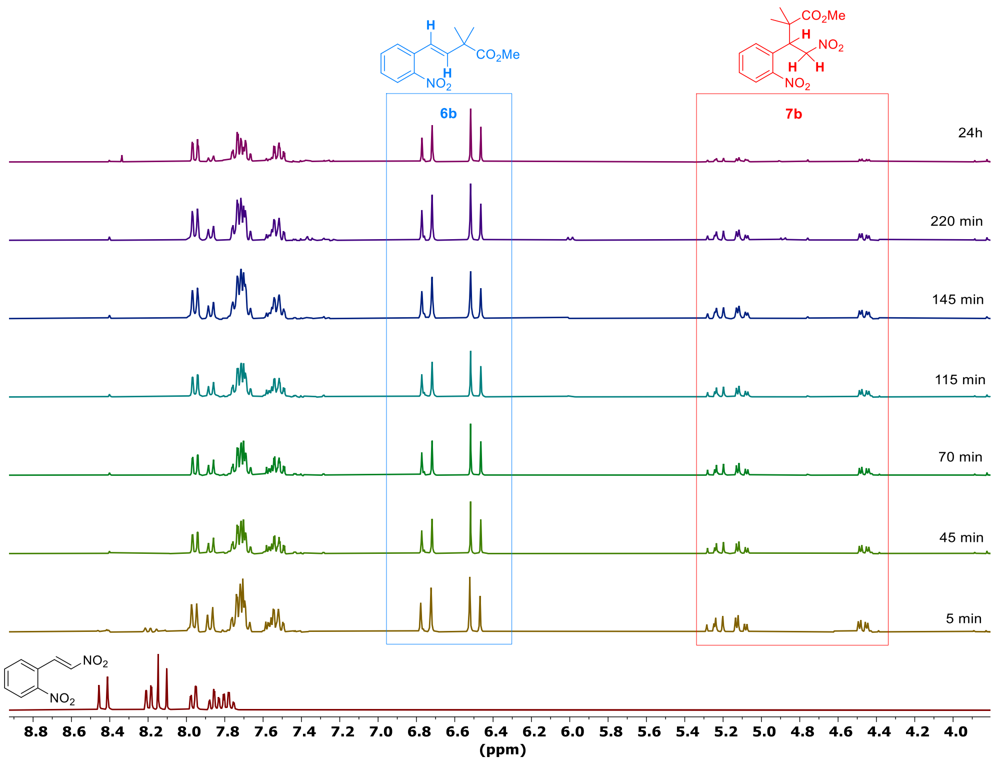


**Figure S1**. $^1$H NMR (300 MHz) monitoring of the reaction between **1b** and **3'** under conditions A. (3 equiv. $K_2CO_3$, DMSO-*d6* at room temperature). The spectrum of compound **1b** is also shown at the bottom. The boxed signals corresponding to both products **6b** and **7b**.

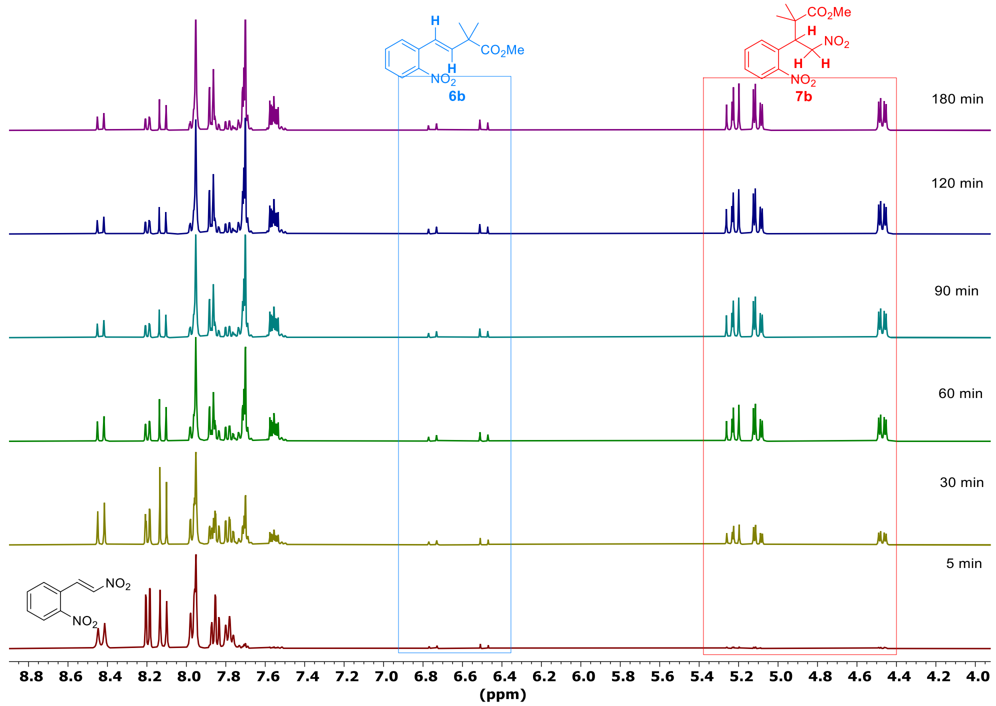


**Figure S2**. $^1$H NMR (400 MHz) monitoring of the reaction between **1b** and **3'** in DMSO-*d6* (without $K_2CO_3$). The spectrum of compound **1b** is also shown at the bottom. The boxed signals corresponding to both products **6b** and **7b**.

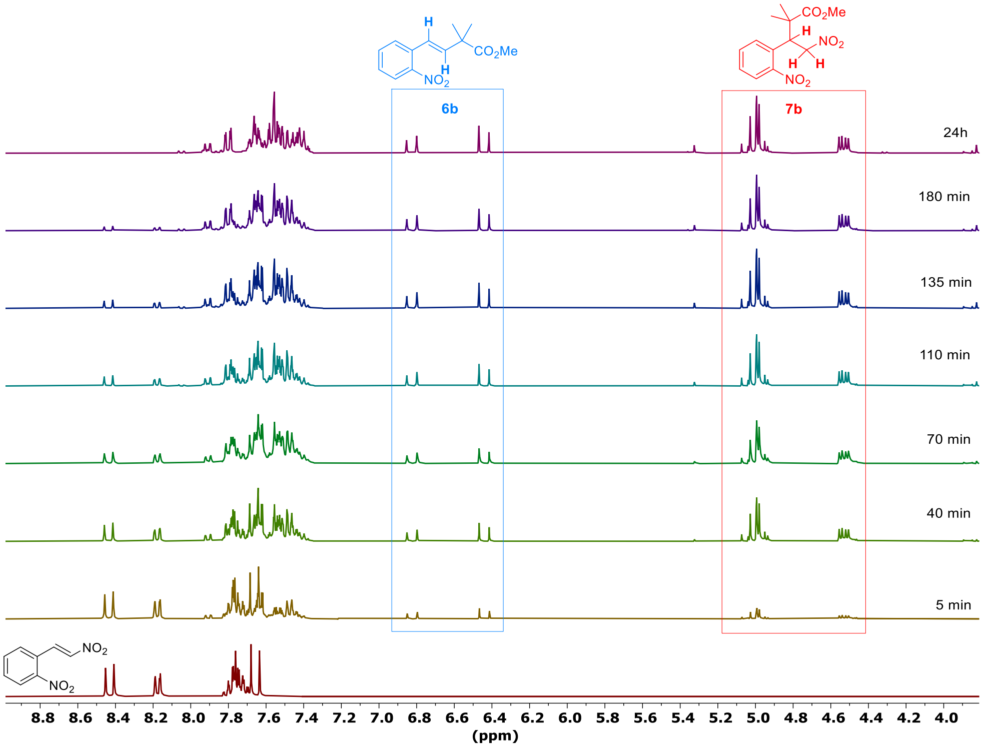


**Figure S3**. ¹H NMR (300 MHz) monitoring of the reaction between **1b** and **3'** under conditions B. (0.1 equiv. TBAT, $CD_3CN$ at room temperature). The spectrum of compound **1b** is also shown at the bottom. The boxed signals corresponding to both products **6b** and **7b**.

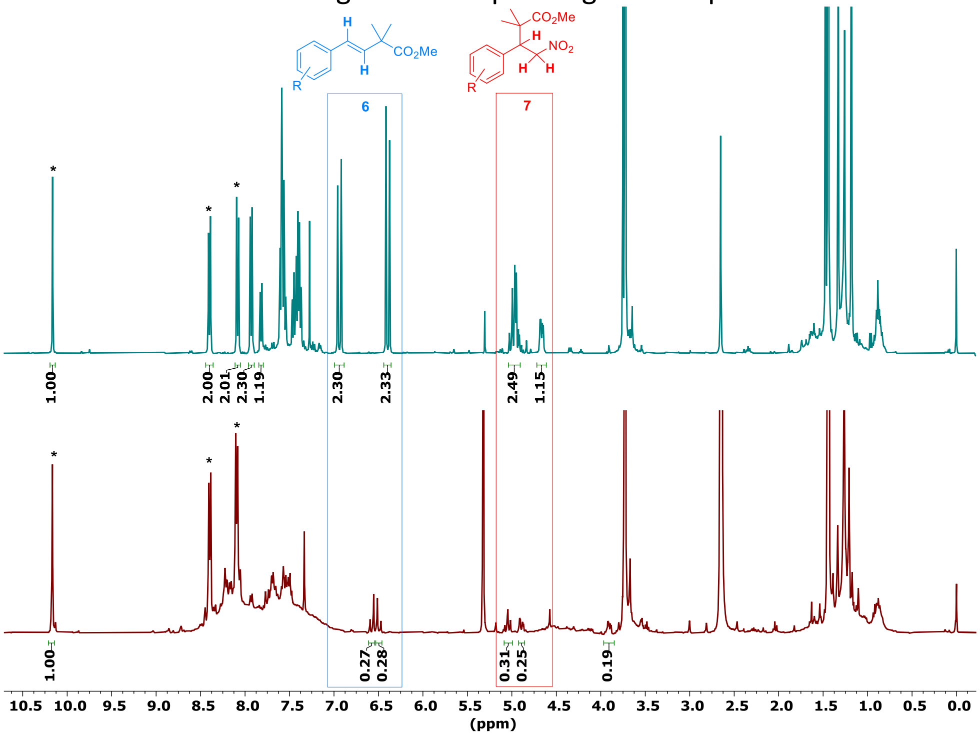


**Figure S4**. ¹H NMR (400 MHz, $CDCl_3$) of the crude reaction mixture of **1b** (top) and **1g** (bottom) under conditions **A** with 17% mol above and 16% mol below of 4-nitrobenzaldehyde as internal standard (* signals of 4-nitrobenzaldehyde). The boxed signals correspond to products **6** and **7**, respectively.

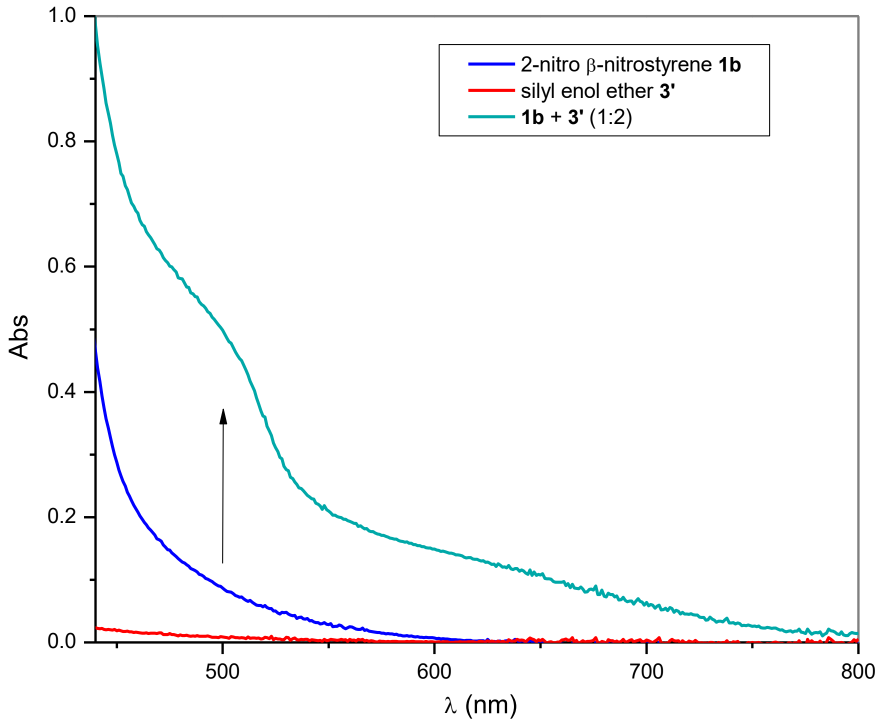


**Figure S5**. UV-VIS spectra of **1b** (0.1M), **3’** (0.2M) and the mixture of the reactants (1:2) in DMSO.

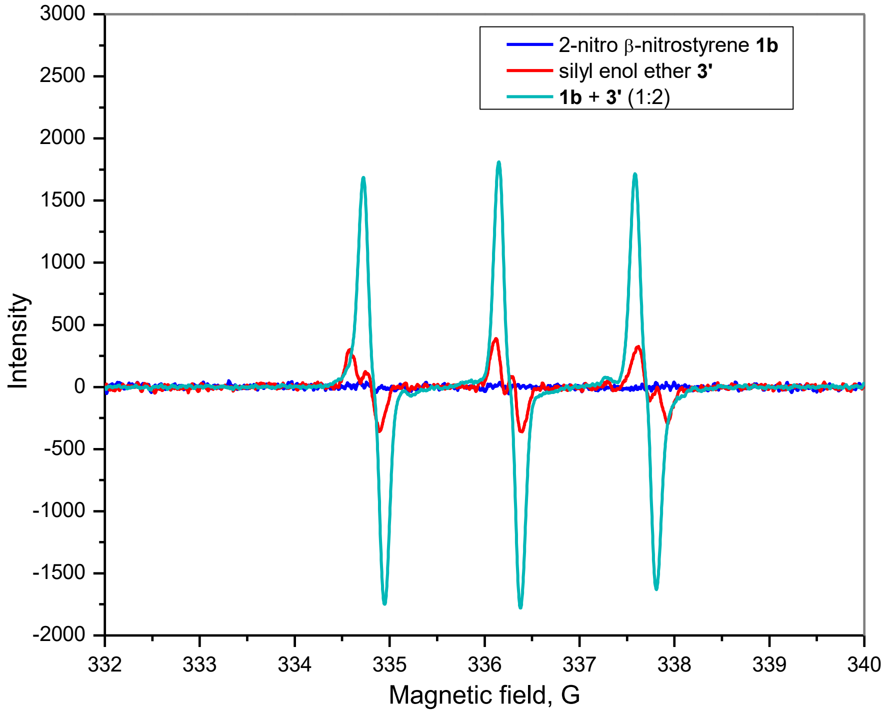


**Figure S6**. EPR spectra of **1b** (0.1M), **3’** (0.2M) in DMSO and EPR spectrum of the 1:2 mixture of **1b** and **3’** in DMSO (0.2M).

## 2. **Electrophilic Parr functions and frontier molecular orbital energies of nitroalkenes**

**Table S1**. Electrophilic Parr functions in a.u. computed by NBO ASD analyses for the carbon atoms of the double bond motif and frontier molecular orbital (FMO) energies (in eV) for the nitrostyrenes investigated in this study.

| System | $P^+(C_\alpha)$ | $P^+(C_\beta)$ | $\Delta P^+(C_k)$ | $\varepsilon_{HOMO}$ [eV] | $\varepsilon_{LUMO}$ [eV] | $\Delta\varepsilon$ [eV] |
|---|---|---|---|---|---|---|
| **1a** | 0.113 | 0.117 | 0.058 | -7.68640 | -2.04249 | 5.64392 |
| **1b** | 0.119 | 0.152 | 0.033 | -9.28126 | -2.36984 | 6.91142 |
| **1c** | 0.115 | 0.211 | 0.096 | -8.66520 | -2.04086 | 6.62434 |
| **1d** | 0.120 | 0.185 | 0.065 | -9.26303 | -2.45909 | 6.80394 |
| **1e** | 0.084 | 0.237 | 0.153 | -8.48914 | -1.88956 | 6.59958 |
| **1f** | 0.088 | 0.217 | 0.129 | -8.08532 | -1.72330 | 6.36203 |
| **1g** | 0.120 | 0.185 | 0.065 | -9.26303 | -2.45909 | 6.80394 |
| **1h** | 0.122 | 0.213 | 0.090 | -9.15718 | -2.39379 | 6.76339 |
| **1I** | 0.131 | 0.115 | -0.016 | -9.37133 | -2.79652 | 6.57482 |
| **1j** | 0.135 | 0.173 | 0.038 | -9.06004 | -2.57692 | 6.48312 |
| **1k** | 0.103 | 0.235 | 0.132 | -8.53649 | -2.15678 | 6.37971 |
| **1l** | 0.107 | 0.229 | 0.122 | -8.46655 | -2.17446 | 6.29209 |
| **1m** | 0.122 | 0.161 | 0.039 | -7.63334 | -2.19923 | 5.43412 |
| **1n** | 0.122 | 0.178 | 0.056 | -8.80506 | -2.30154 | 6.50352 |
| **1o** | 0.088 | 0.249 | 0.161 | -8.58465 | -1.98317 | 6.60149 |
| **1p** | 0.069 | 0.270 | 0.201 | -7.91879 | -1.77554 | 6.14325 |
| **1q** | 0.097 | 0.163 | 0.066 | -7.96995 | -2.11677 | 5.85317 |
| **1r** | 0.150 | 0.197 | 0.050 | -9.35909 | -2.56876 | 6.79033 |
| **1s** | 0.120 | 0.221 | 0.100 | -9.41433 | -2.35324 | 7.06109 |
| **1t** | 0.079 | 0.260 | 0.180 | -7.73538 | -1.84303 | 5.89236 |
| **1u** | 0.120 | 0.182 | 0.062 | -8.48179 | -2.05446 | 6.42733 |
| **1v** | 0.109 | 0.043 | -0.066 | -10.08645 | -3.09775 | 6.98870 |
| **1w** | 0.001 | 0.228 | 0.227 | -8.45594 | -1.52357 | 6.93238 |
| **1x** | 0.062 | 0.243 | 0.181 | -8.43526 | -1.67268 | 6.76258 |
| **1y** | 0.085 | 0.092 | 0.010 | -9.07146 | -2.11269 | 6.95877 |
| **1z** | 0.101 | 0.090 | -0.010 | -9.19310 | -2.47161 | 6.72149 |
| **1za** | 0.085 | 0.101 | 0.020 | -9.33678 | -2.44957 | 6.88721 |

## 3. Experimental procedure of starting materials

**Synthesis of nitroalkenes 1**

Nitroalkenes **1b-1l**, **1n**, **1p**, **1q**, **1s**, **1t** and **1w** were prepared following General Procedure **D**, in accordance with previously reported methods. The $^{1}H$ and $^{13}C$ NMR data obtained were consistent with those reported in the literature.[1]

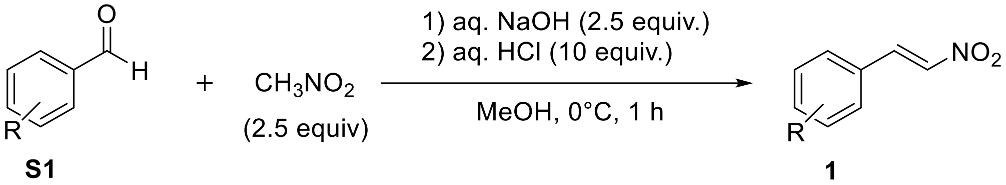


**General Procedure D:** To a solution of the appropriate aldehyde **S1** (1.0 equiv) and nitromethane ($CH_3NO_2$, 2.5 equiv) in methanol (MeOH, 0.2 M), an aqueous solution of sodium hydroxide (NaOH, 10 M, 2.5 equiv) was added dropwise at 0 °C. The reaction mixture was stirred at 0 °C for 1 h. Crushed ice was then added, and the mixture was slowly poured into vigorously stirred aqueous hydrochloric acid (HCl, 5 M, 10 equiv) at 0 °C. The resulting precipitate was collected by filtration, washed with water (×2), and dried under reduced pressure to afford the nitroalkene.If precipitation d id not occur, the mixture was extracted with ethyl acetate (EtOAc), and the combined organic layers were washed with brine, dried over anhydrous sodium sulfate ($Na_2SO_4$), and concentrated under reduced pressure. The crude product was purified by flash chromatography using a hexane/EtOAc (8:2) mixture to yield the corresponding nitroalkene.

Nitroalkenes **1a**, **1m** and **1u** were prepared according to General Procedure **E** in accordance with previously reported methods. The $^{1}H$ and $^{13}C$ NMR data obtained were consistent with those reported in the literature.[2]

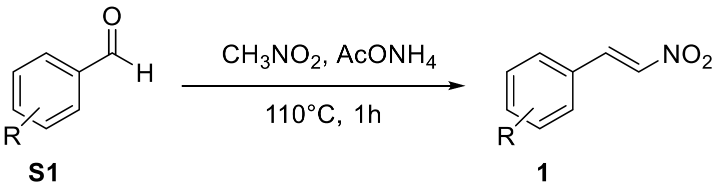


**General procedure E:** A solution of the corresponding aldehyde **S1** (1.0 equiv) and ammonium acetate ($AcONH_4$) (1.0 equiv) in nitromethane (1 M) was stirred at 110 °C. Upon completion of the reaction, as monitored by TLC (~1h), the reaction mixture was cooled to room temperature. The mixture was then concentrated, and the residue was partitioned between ethyl acetate (EtOAc) and water. The aqueous layer was further extracted with EtOAc, and the combined organic layers were washed with brine, dried over sodium sulfate ($Na_2SO_4$), and concentrated under reduced

pressure. The crude product was purified by flash chromatography using a hexane/EtOAc (99:1) mixture as the eluent.

(*E*)-1-(2-Nitrovinyl)-4-(phenylethynyl)benzene (**S-1m**). Physical state: pale yellow solid, Isolated yield: 41% (276 mg). $^{1}$H NMR (400 MHz, $CDCl_3$): δ 8.00 (d, *J* = 13.7 Hz, 1H), 7.63 – 7.51 (m, 7H), 7.41 – 7.35 (m, 3H). $^{13}$C{$^{1}$H} NMR (100 MHz, $CDCl_3$): δ 138.3, 137.5, 132.5, 131.9, 129.8, 129.2, 129.1, 128.6, 127.4, 122.7, 93.1, 88.7. HRMS (EI): $[M]^+$ calcd. For $C_{16}H_{11}NO_2$: 249.0790, found: 249.0787.

Nitroalkenes **1y**[3] and **1z**[4] were prepared according to General Procedure **F** as previously reported procedure[5] and $^{1}$H and $^{13}$C NMR data were consistent with those reported in the literature.

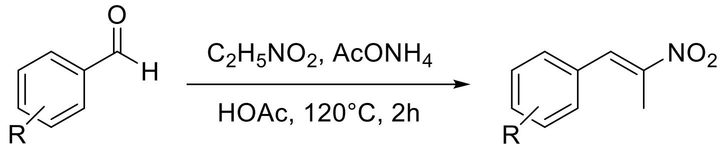


**S1** **1**

**General procedure F:** A solution of the corresponding aldehyde **S1** (1.0 equiv), nitroethane ($C_2H_5NO_2$) (2.0 equiv) and ammonium acetate (1.1 equiv) in acetic acid (HOAc) (2.0 M) was stirred at 120°C. Upon completion of the reaction, as monitored by TLC (~2h), the reaction mixture was poured into ice water, extracted with EtOAc (3 ×). The combined organic layers were washed with saturated $NaHCO_3$ (2 ×) and dried over $Na_2SO_4$, solvent was evaporated, and the product was purified by flash chromatography (hexane/ EtOAc, 9:1).

**Nitroalkenes obtained by other methodologies**

*(E)-1,2,3,4,5-Pentafluoro-6-(2-nitrovinyl)benzene* **(1r)**

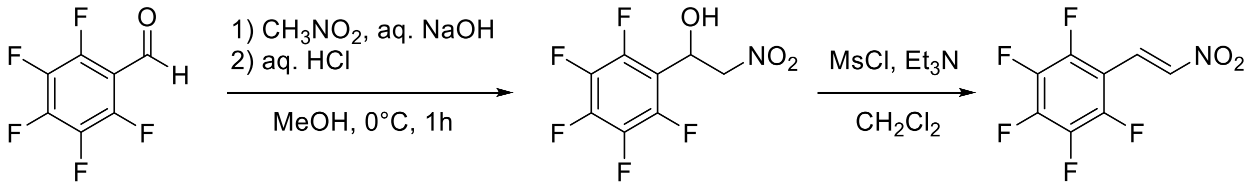


**S2** **1r**

According to a reviously reported procedure[6] 2,3,4,5,6-pentafluorobenzaldehyde **S2** (600 mg, 3 mmol, 1.0 equiv) and nitromethane (0.4 mL, 7.5 mmol, 2.5 equiv) were dissolved in MeOH (15 mL, 0.2 M), and the solution was cooled to 0°C. NaOH (1 M in $H_2O$; 3.4 mL, 3.4 mmol, 1.13 equiv) was added dropwise and the reaction mixture was stirred for 1 h. at 0°C. The mixture was then poured into HCl (1 M in $H_2O$; 7.7 mL, 7.7 mmol, 2.26 equiv) and stirred thoroughly. The resulting emulsion was extracted with $CH_2Cl_2$ (50 mL) and the organic layer was washed with $H_2O$ (25 mL) and saturated aqueous NaCl (25 mL), then dried over $Na_2SO_4$, filtered, and concentrated in vacuo. The crude nitro-alcohol product (533 mg) was directly used for the next step without further

purification.2-Nitro-1-(per fluorophenyl)ethanol (533 mg, 2.07 mmol, 1 equiv) was dissolved in $CH_2Cl_2$ (10 mL, 0.2 M) and the solution was cooled to –20°C. $Et_3N$ (0.88 mL, 6.21 mmol, 3.0 equiv) was added, followed by dropwise addition of methanesulfonyl chloride (MsCl) (0.48 mL, 6.21 mmol, 3.0 equiv). The reaction mixture was stirred at –20°C for 1 h and then quenched with $H_2O$ (15 ml). The mixture was extracted with $CH_2Cl_2$ (30 mL), and the organic layer was washed with $H_2O$ (2 × 15 mL) and saturated aqueous NaCl (15 mL), then dried over $Na_2SO_4$, filtered, and concentrated in vacuo. The crude product was purified by flash chromatography using a hexane/EtOAc (9:1) mixture as the eluent. Physical state: yellow oil. Isolated yield: 61% (438 mg). $^1$H NMR (700 MHz, $CDCl_3$): δ 8.02 (d, *J* = 14.0 Hz, 1H), 7.81 (d, *J* = 14.0 Hz, 1H). $^{13}$C{$^1$H} NMR (176 MHz, $CDCl_3$): δ 146.8 (ddt, *J* = 11.4, 7.5, 3.9 Hz), 145.3 (ddt, *J* = 11.3, 7.4, 3.9 Hz), 144.0 (td, *J* = 13.2, 6.6 Hz), 142.6 – 142.1 (m), 139.4 – 138.6 (m), 137.8 – 137.2 (m), 123.2, 106.3 (td, *J* = 14.0, 4.2 Hz). HRMS (EI): $[M]^+$ calcd. For $C_8H_2F_5NO_2$: 239.0006, found: 239.0003.

(*E*)-2,4-Dinitro-1-(2-nitrovinyl)benzene (**1v**)

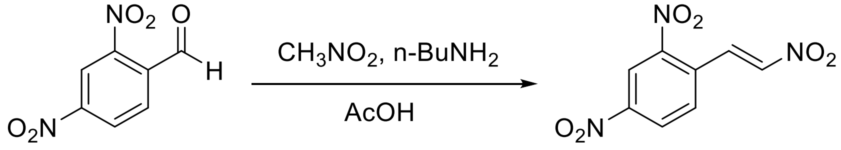


**S3** **1v**

A solution of 2,4-dinitrobenzaldehyde **S3** (500 mg, 2.5 mmol, 1.0 equiv), nitromethane (0.27 mL, 5 mmol, 2.5 equiv), and *n*-butylamine (0.28 mL, 2.8 mmol, 1.1 equiv) in acetic acid (HOAc) (2.5 mL, 1.0 M) was stirred at 120°C. Upon completion of the reaction (monitored by TLC, ~2 h), the reaction mixture was poured into ice water and extracted with EtOAc (3 × 10 mL). The combined organic layers were washed with saturated $NaHCO_3$ (2 × 10 mL), dried over $Na_2SO_4$, and concentrated under reduced pressure. The crude product was purified by flash chromatography (hexane/EtOAc, 8:2). Physical state: yellow solid, Isolated yield: 39% (233.0 mg). m.p.: 105 – 106 °C. $^1$H NMR (400 MHz, $CDCl_3$): δ 9.05 (d, *J* = 2.3 Hz, 1H), 8.58 (dd, *J* = 8.5, 2.3 Hz, 1H), 8.53 (d, *J* = 13.6 Hz, 1H), 7.84 (d, *J* = 8.5 Hz, 1H), 7.47 (d, *J* = 13.6 Hz, 1H). $^{13}$C{$^1$H} NMR (100 MHz, $CDCl_3$): δ 149.3, 141.8, 133.4, 132.3, 131.4, 128.4, 121.3. HRMS (EI): $[M]^+$ calcd. For $C_8H_5N_3O_6$: 239.0178, found: 239.0172.

(*E*)-1-nitro-4-(1-nitroprop-1-en-2-yl)benzene (1za)

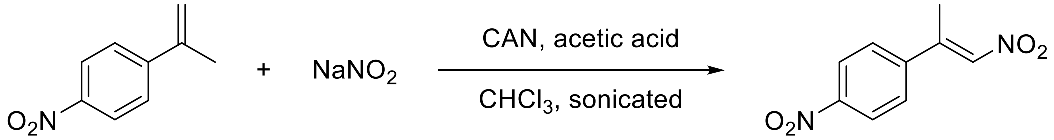


**S4** **1az**

It was prepared by a reported procedure and $^1$H and $^{13}$C{$^1$H} NMR data were consistent with those reported in the literature.[7] Acetic acid (6.6 mmol, 12 equiv) was added to a suspension of 4-nitro-α-methylstyrene **S4** (0.55 mmol, 1.0 equiv), $NaNO_2$ (5.5 mmol, 10 equiv), and cerium(IV) ammonium nitrate (CAN, 0.55 mmol, 1.0 equiv) in $CHCl_3$ (0.1 M), and the mixture was sonicated

in a sealed flask connected to a bubbler until completion of the reaction, as monitored by TLC (30–60 min). The solution was then diluted with $CHCl_3$, washed with saturated $NaHCO_3$ and water, and dried over sodium sulfate ($Na_2SO_4$). The solvent was evaporated, and the product was purified by flash chromatography on silica gel using hexane/EtOAc (9:1) as the eluent to afford **1za** in 80% yield (91.6 mg).

The synthesis bistrimethylsilylketenes **3a-f** and enol ether **3'** were done following reported procedures.[8]

# 4. $^{1}$H NMR, $^{13}$C{$^{1}$H} NMR spectra of products

## (*E*)-1-(2-Nitrovinyl)-4-(phenylethynyl)benzene (1m)

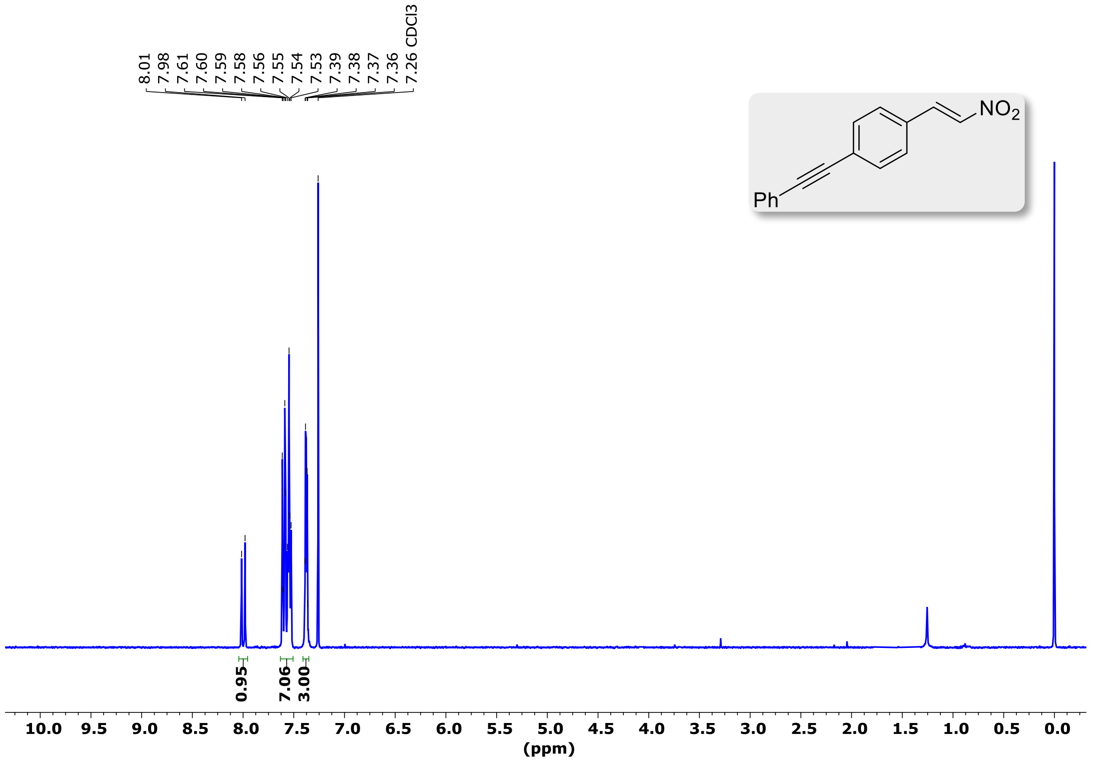


$^{1}$H NMR, $CDCl_3$, 400 MHz (ppm)

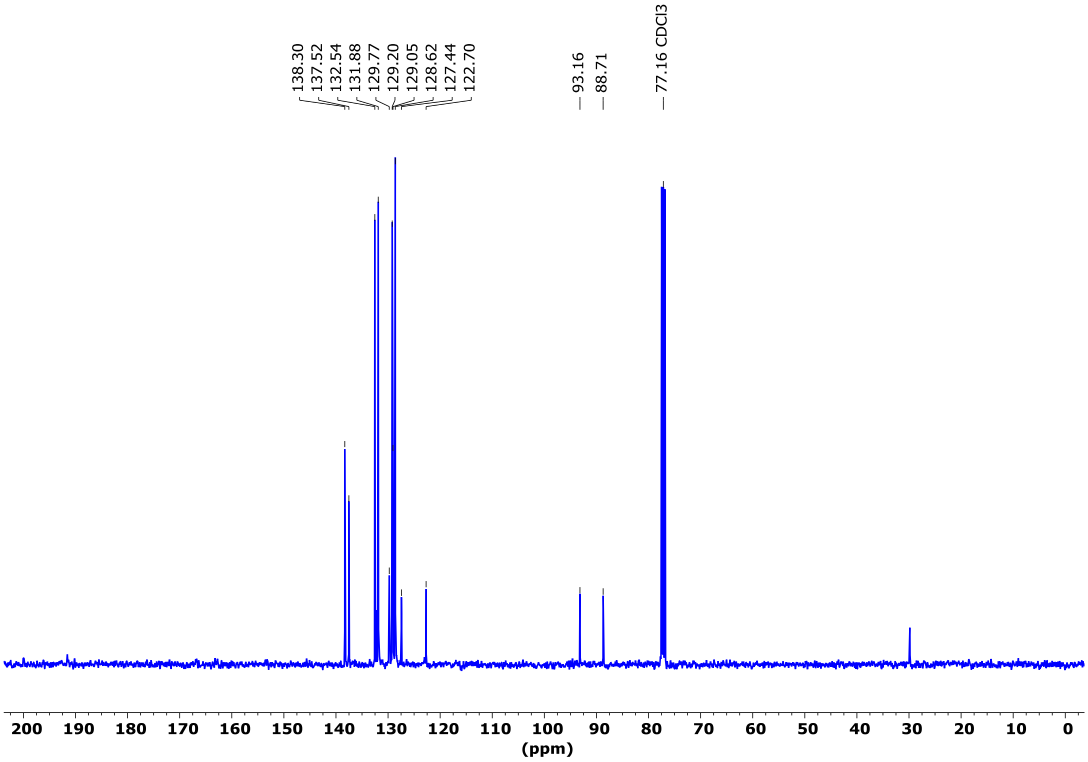


$^{13}$C{$^{1}$H} NMR, $CDCl_3$, 100 MHz (ppm)

## (*E*)-1,2,3,4,5-Pentafluoro-6-(2-nitrovinyl)benzene (1r)

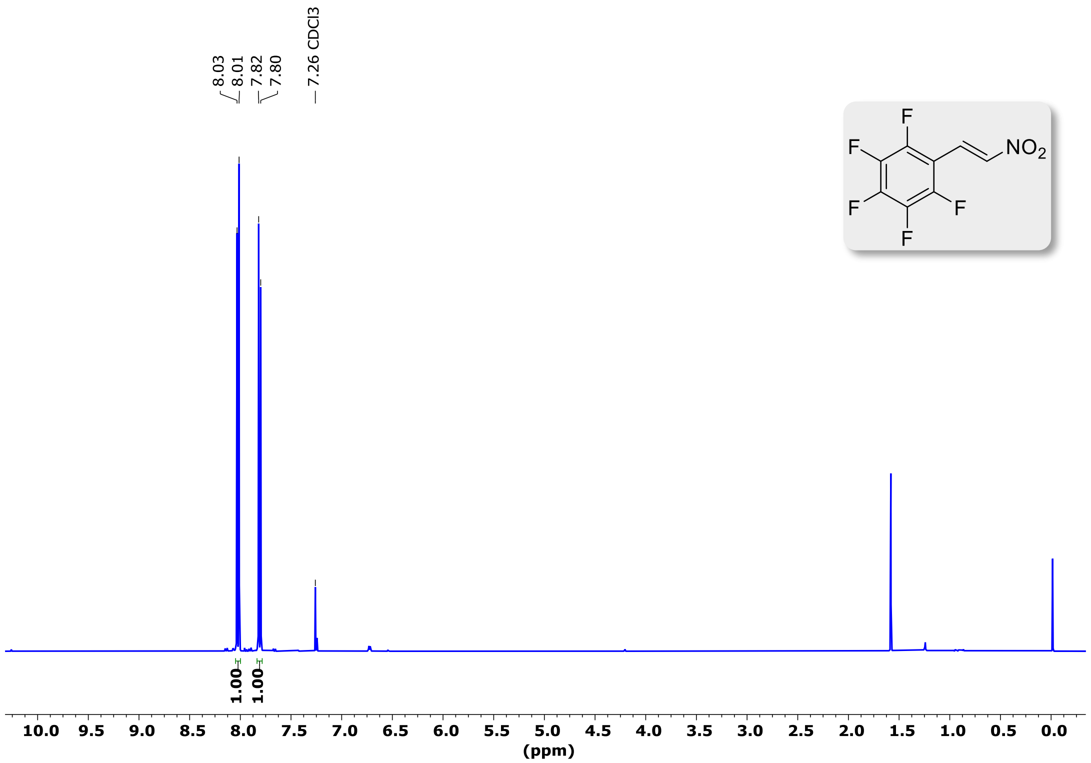


$^{1}H$ NMR, $CDCl_3$, 700 MHz (ppm)

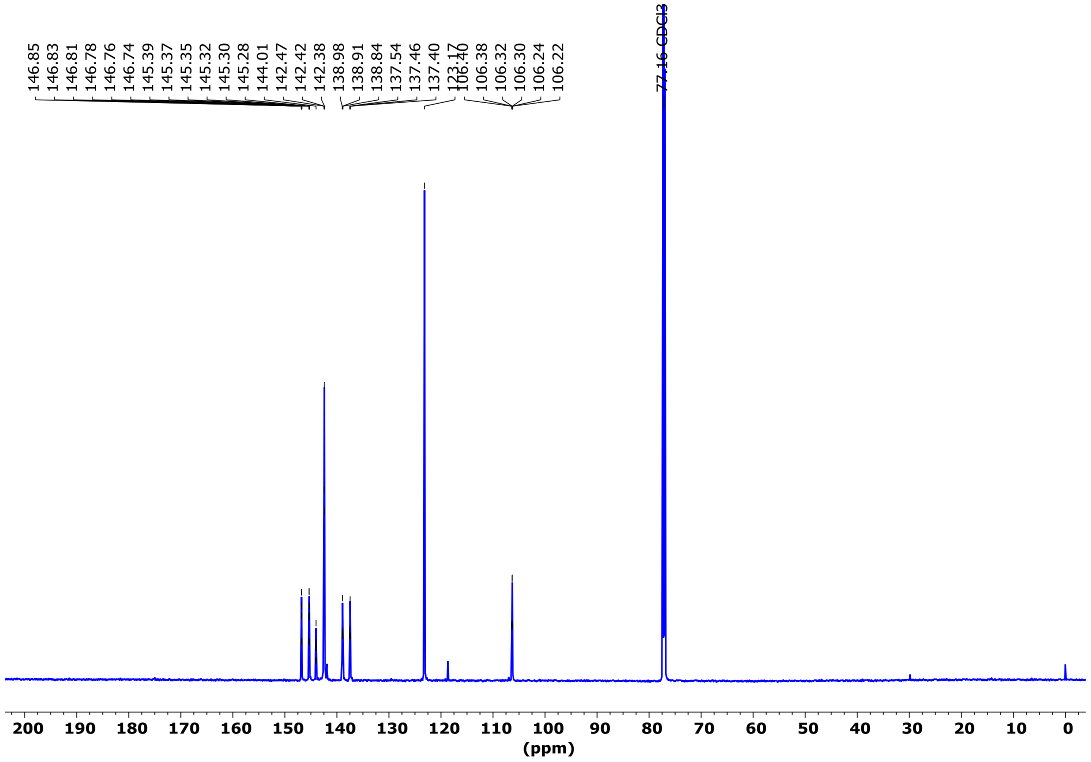


$^{13}C\{^{1}H\}$ NMR, $CDCl_3$, 176 MHz (ppm)

## (*E*)-2,4-Dinitro-1-(2-nitrovinyl)benzene (1v)

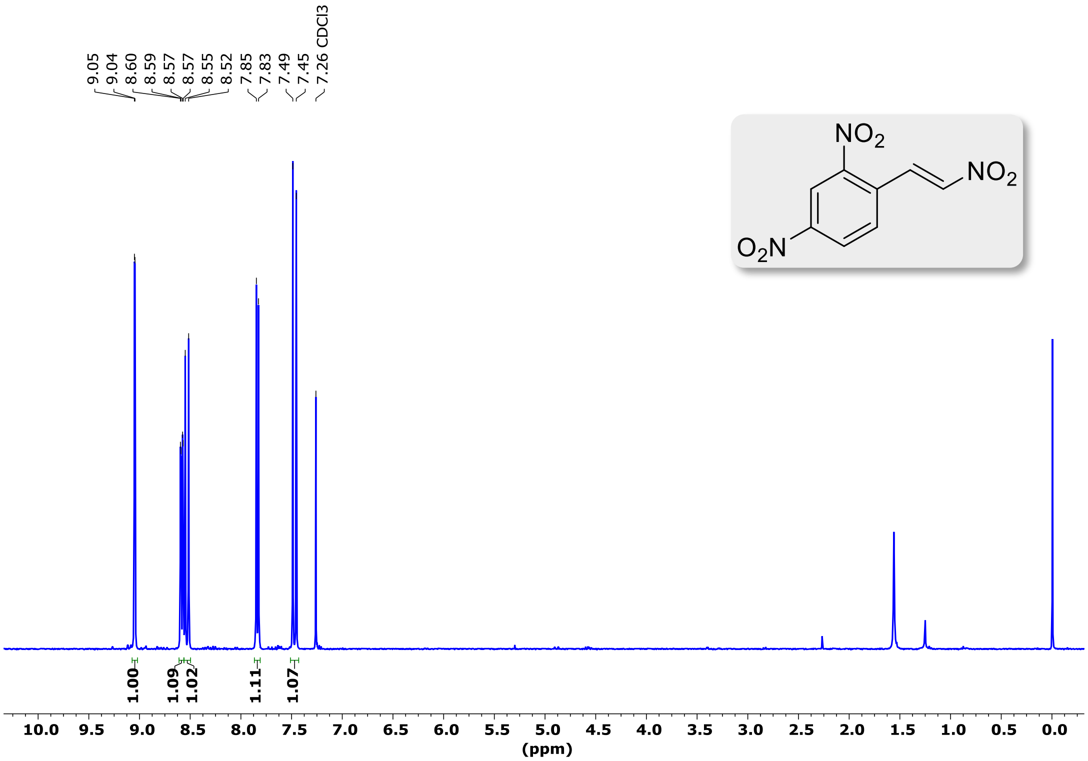


$^{1}H$ NMR, $CDCl_3$, 400 MHz (ppm)

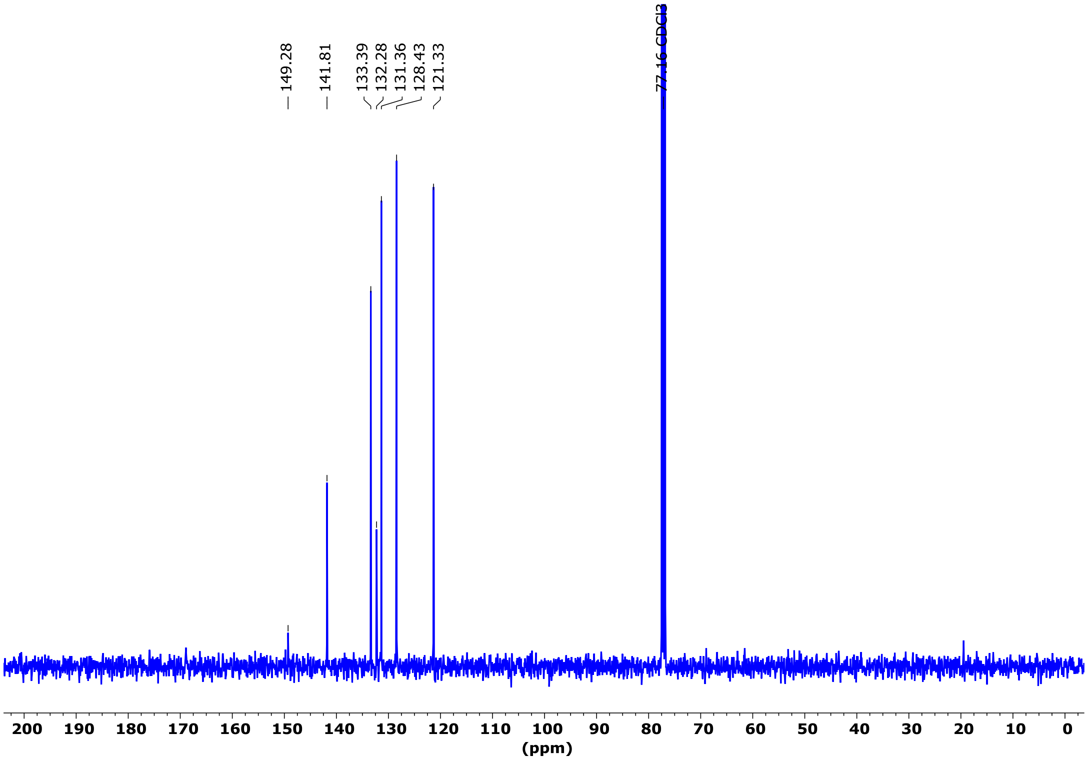


$^{13}C\{^{1}H\}$ NMR, $CDCl_3$, 100 MHz (ppm)

## (*Z*)-8-Benzylidene-3,3-dimethyl-3,3a,8,8a-tetrahydro-2H-indeno[2,1-b]furan-2-one (4)

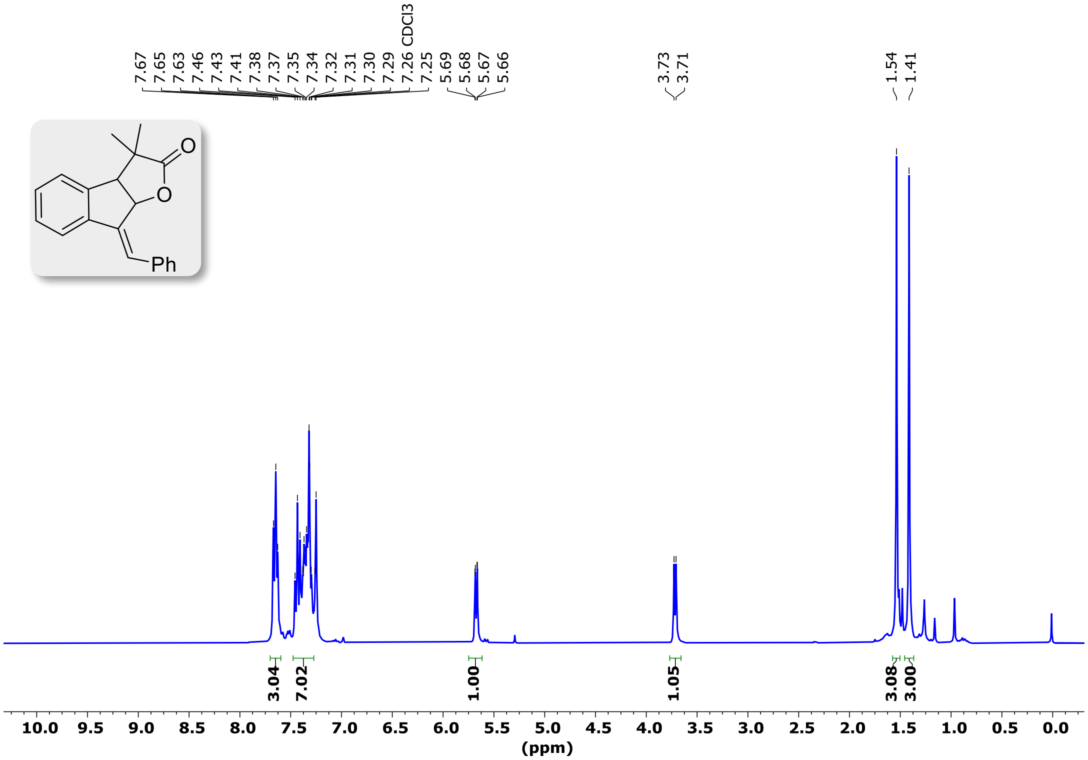


$^{1}H$ NMR, $CDCl_3$, 400 MHz (ppm)

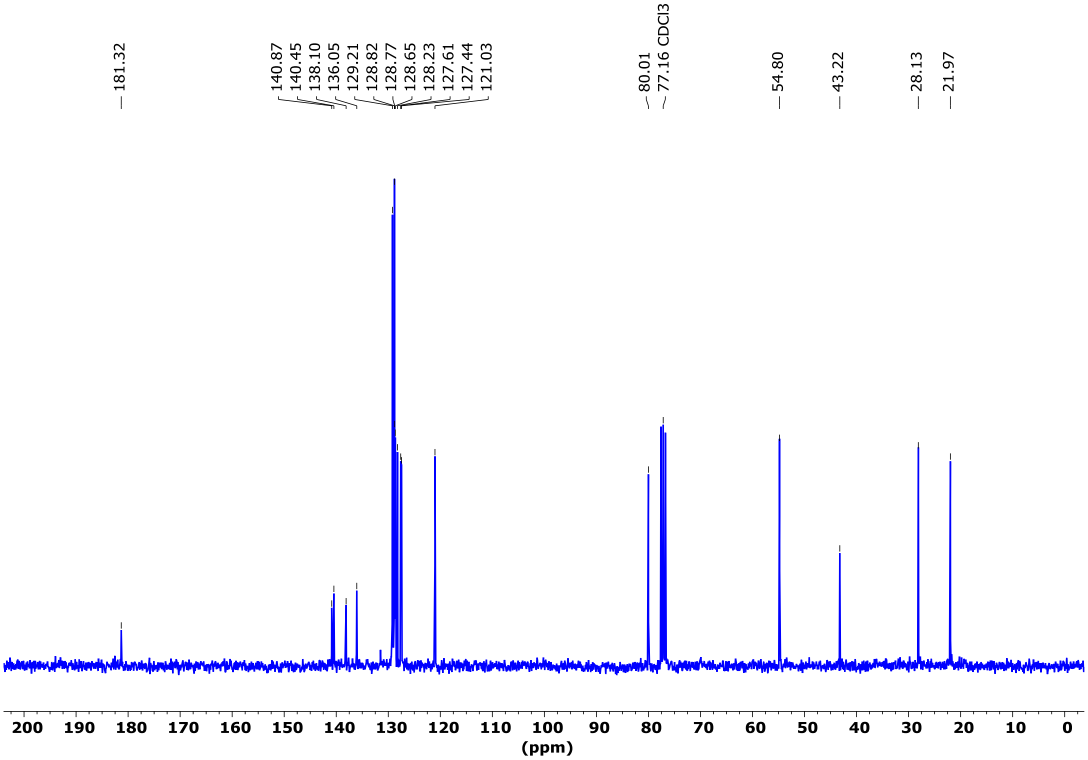


$^{13}C\{^{1}H\}$ NMR, $CDCl_3$, 100 MHz (ppm)

## (*E*)-2,2-Dimethyl-4-(2-(phenylethynyl)phenyl)but-3-enoic acid (5)

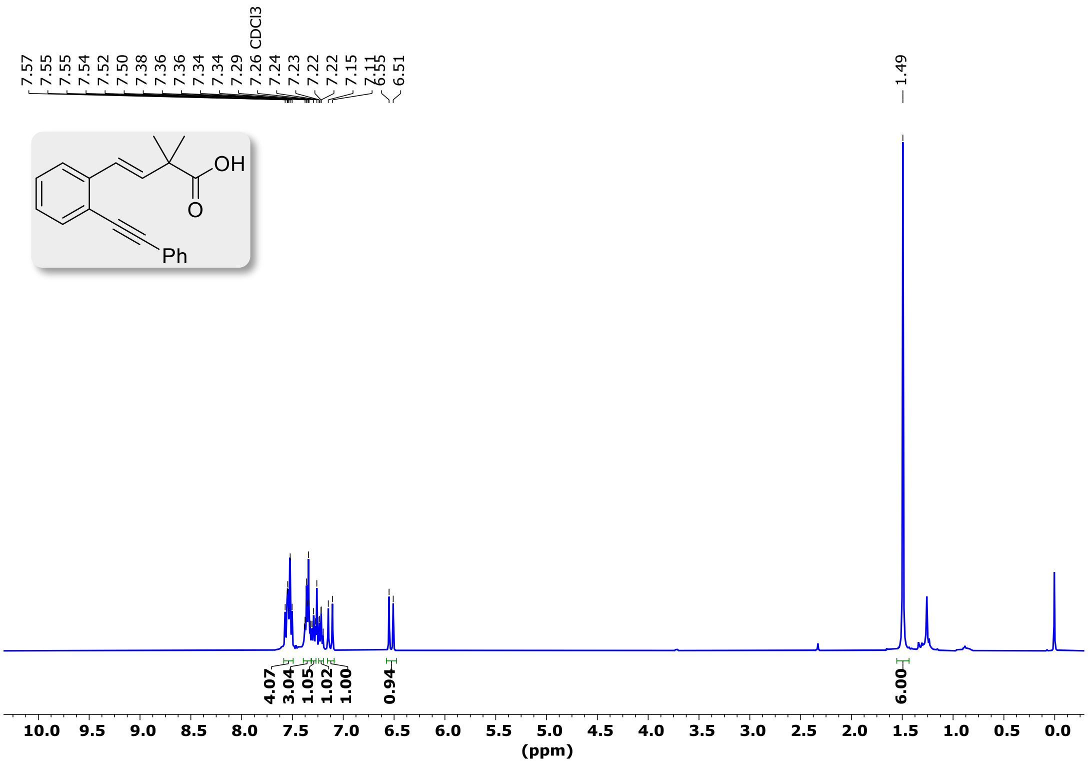


$^{1}$H NMR, $CDCl_3$, 400 MHz (ppm)

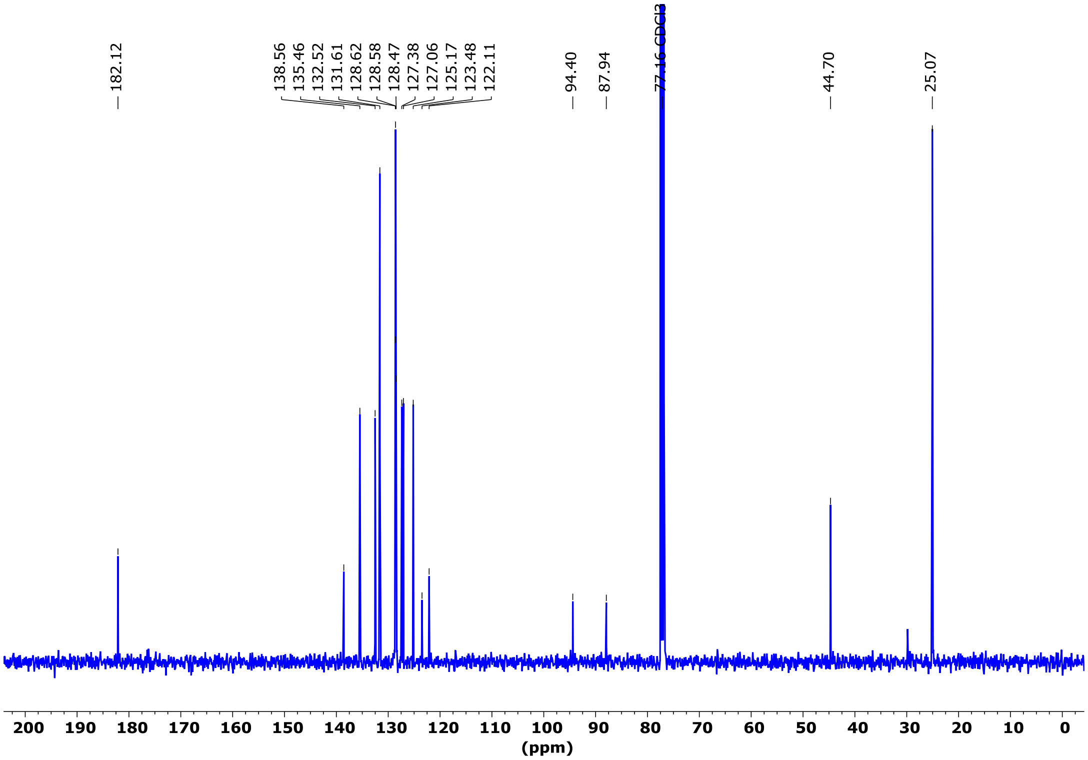


$^{13}$C{$^{1}$H} NMR, $CDCl_3$, 100 MHz (ppm)

## 2,2-Dimethyl-4-nitro-3-(2-(phenylethynyl)phenyl)butanoic acid (7a’)

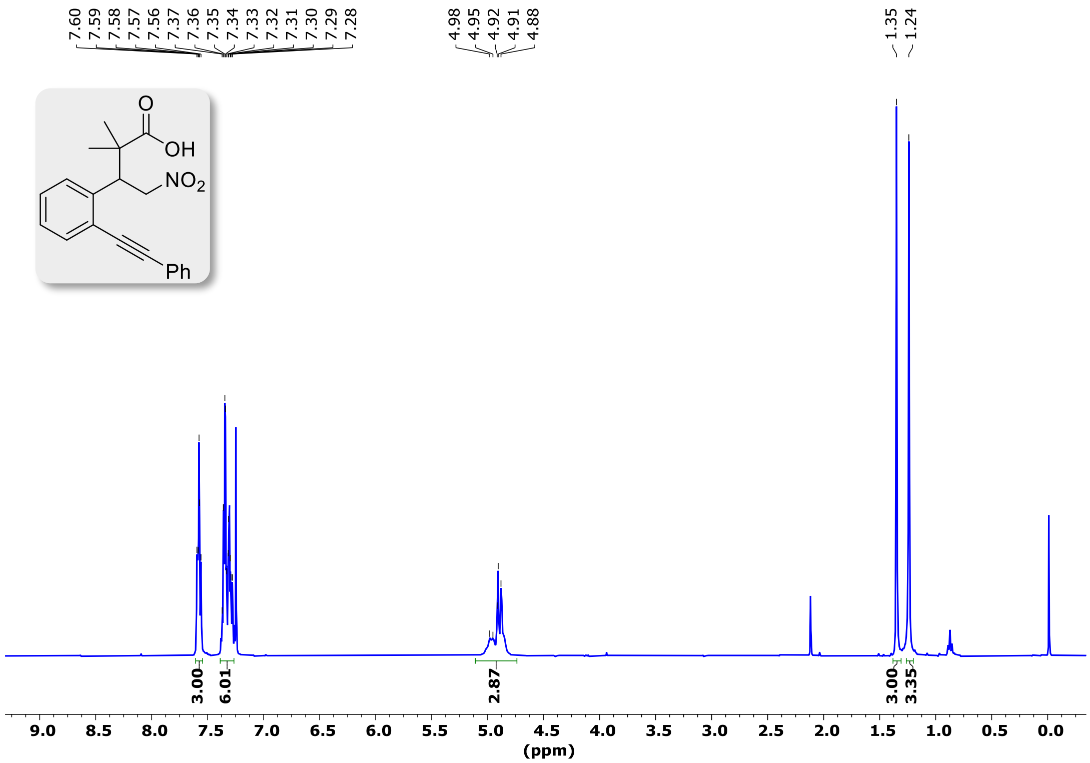


$^{1}$H NMR, CDCl$_3$, 400 MHz (ppm)

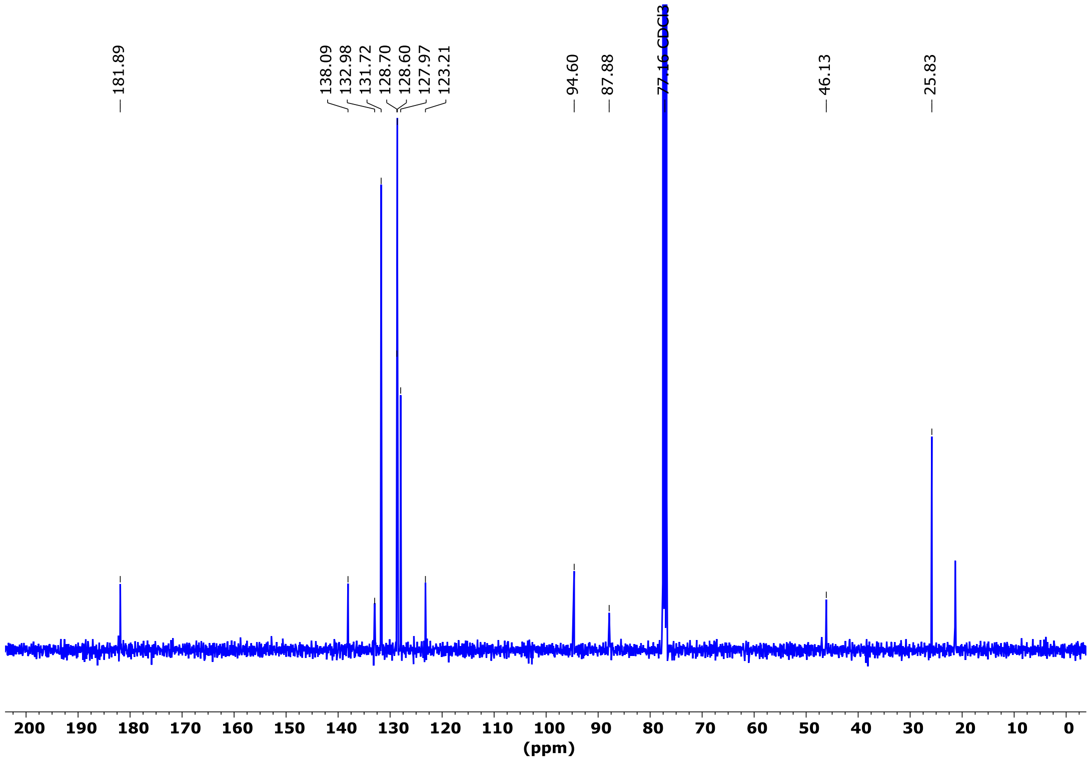


$^{13}$C{$^{1}$H} NMR, CDCl$_3$, 100 MHz (ppm)

## Methyl (*E*)-2,2-dimethyl-4-(2-(phenylethynyl)phenyl)but-3-enoate (6a)

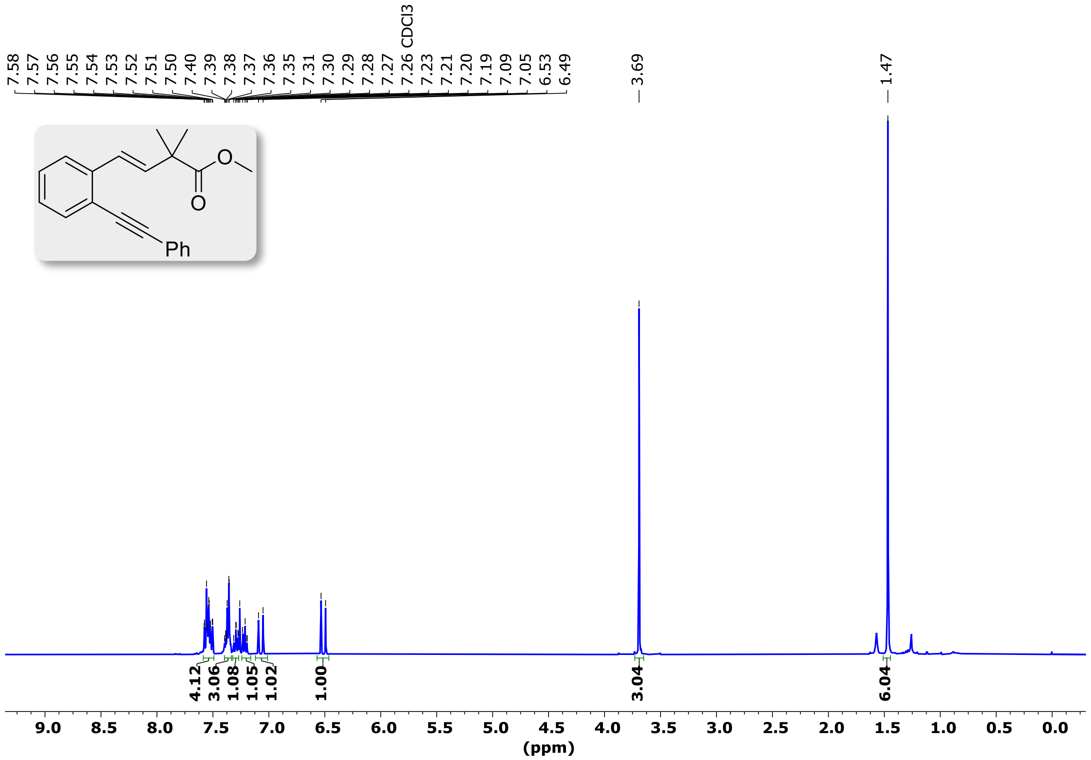


$^{1}H$ NMR, $CDCl_3$, 400 MHz (ppm)

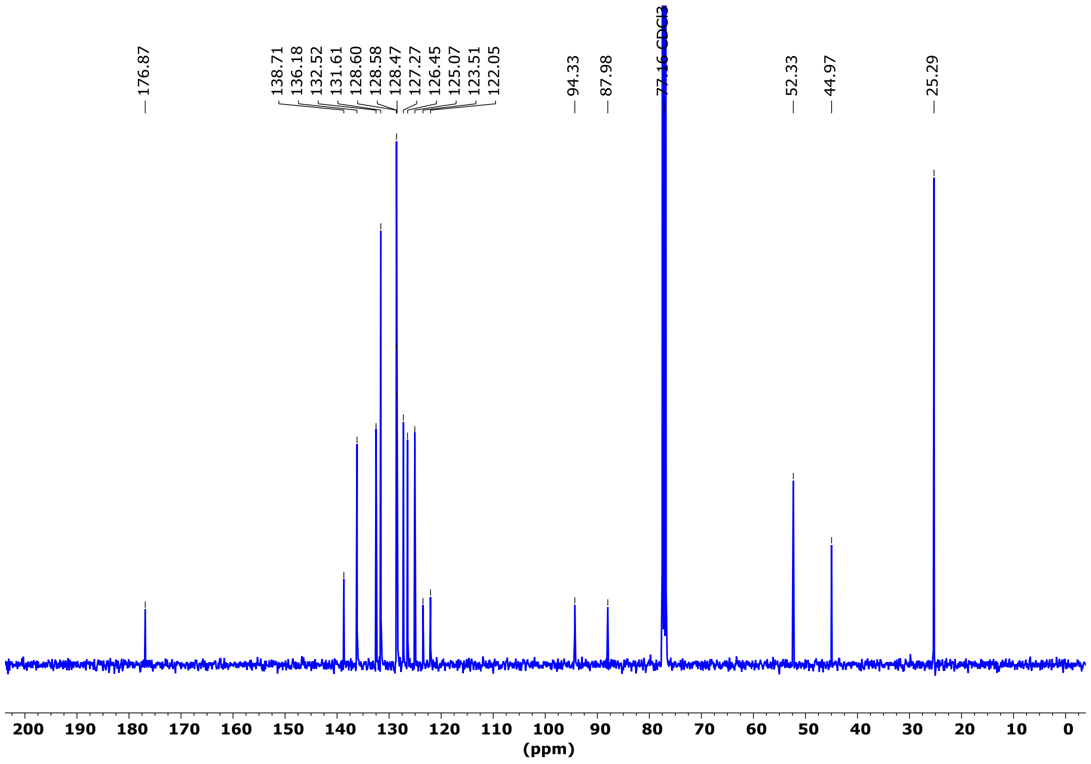


$^{13}C\{^{1}H\}$ NMR, $CDCl_3$, 100 MHz (ppm)

## Methyl 2,2-dimethyl-4-nitro-3-(2-(phenylethynyl)phenyl)butanoate (7a)

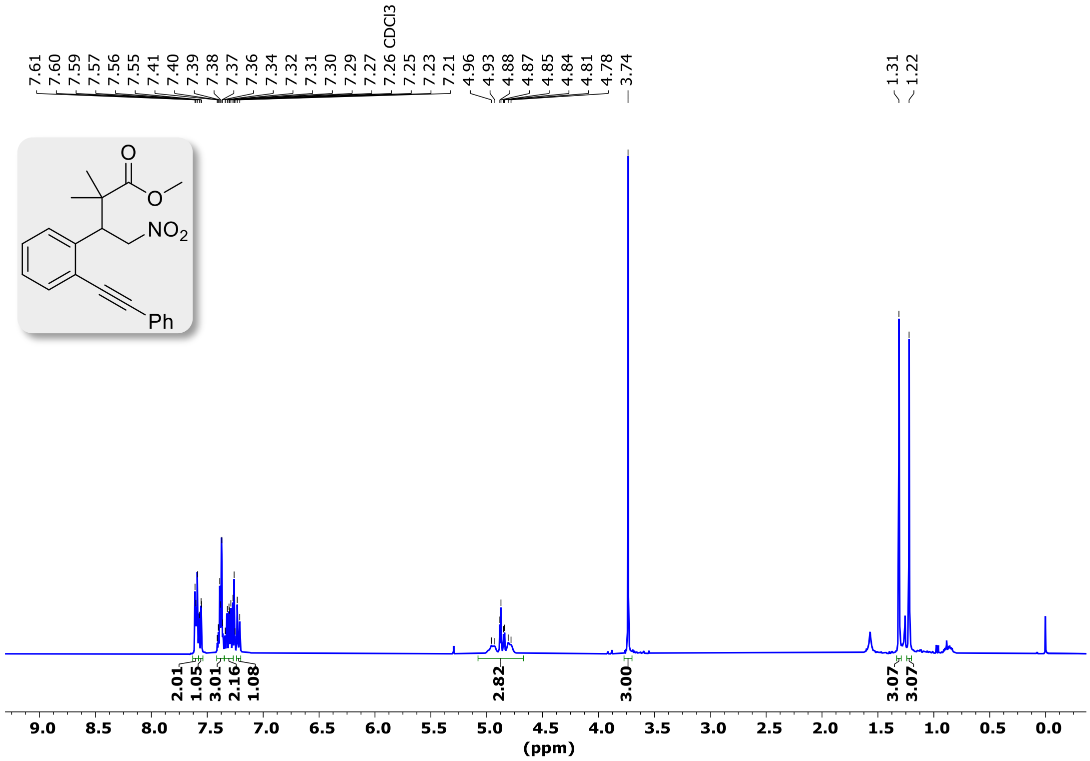


$^{1}H$ NMR, $CDCl_3$, 400 MHz (ppm)

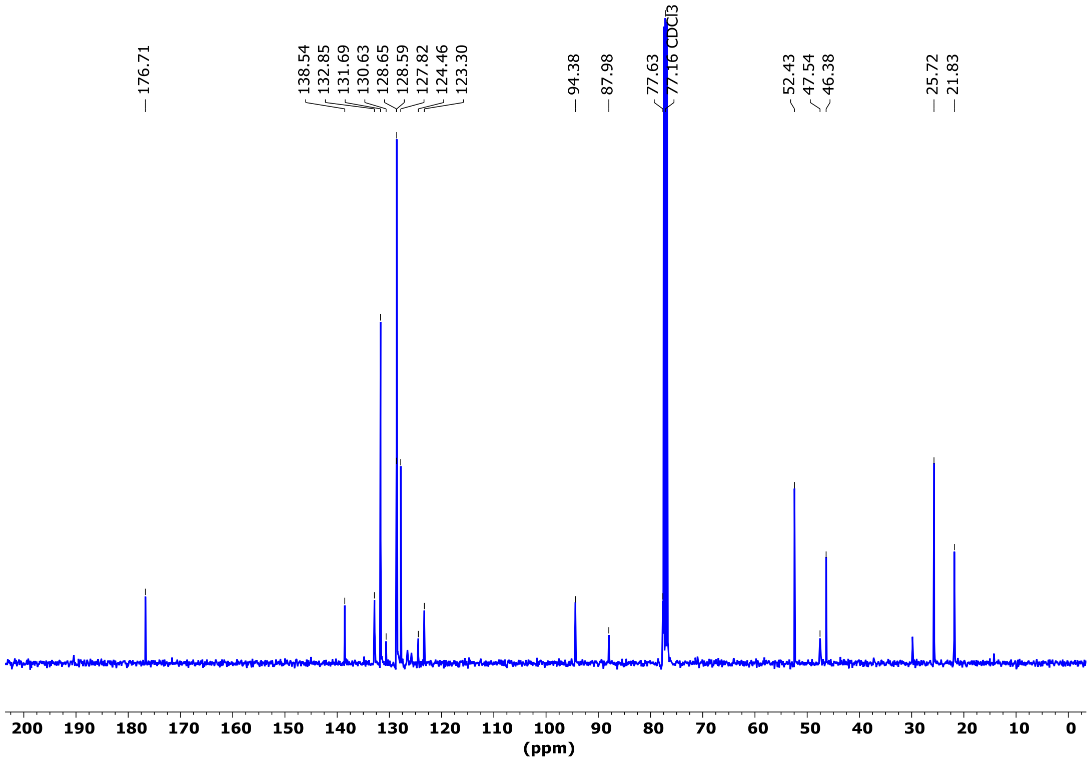


$^{13}C\{^{1}H\}$ NMR, $CDCl_3$, 100 MHz (ppm)

## Methyl (*E*)-2,2-dimethyl-4-(2-nitrophenyl)but-3-enoate (6b)

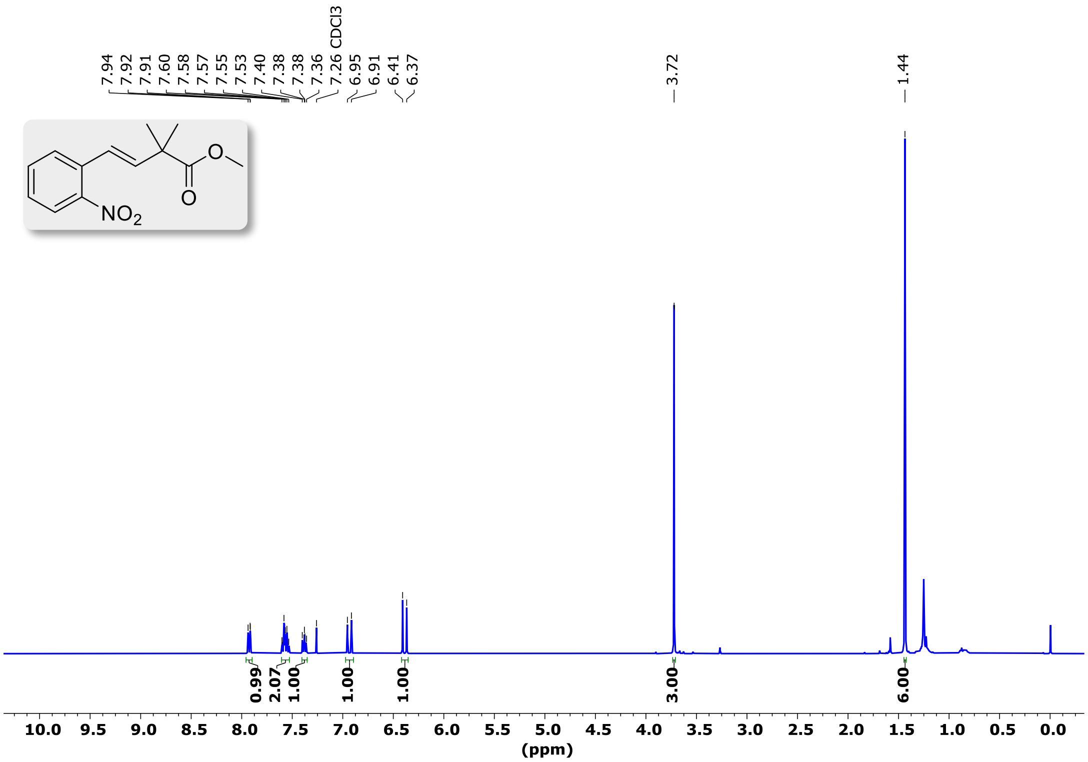


$^{1}H$ NMR, $CDCl_3$, 400 MHz (ppm)

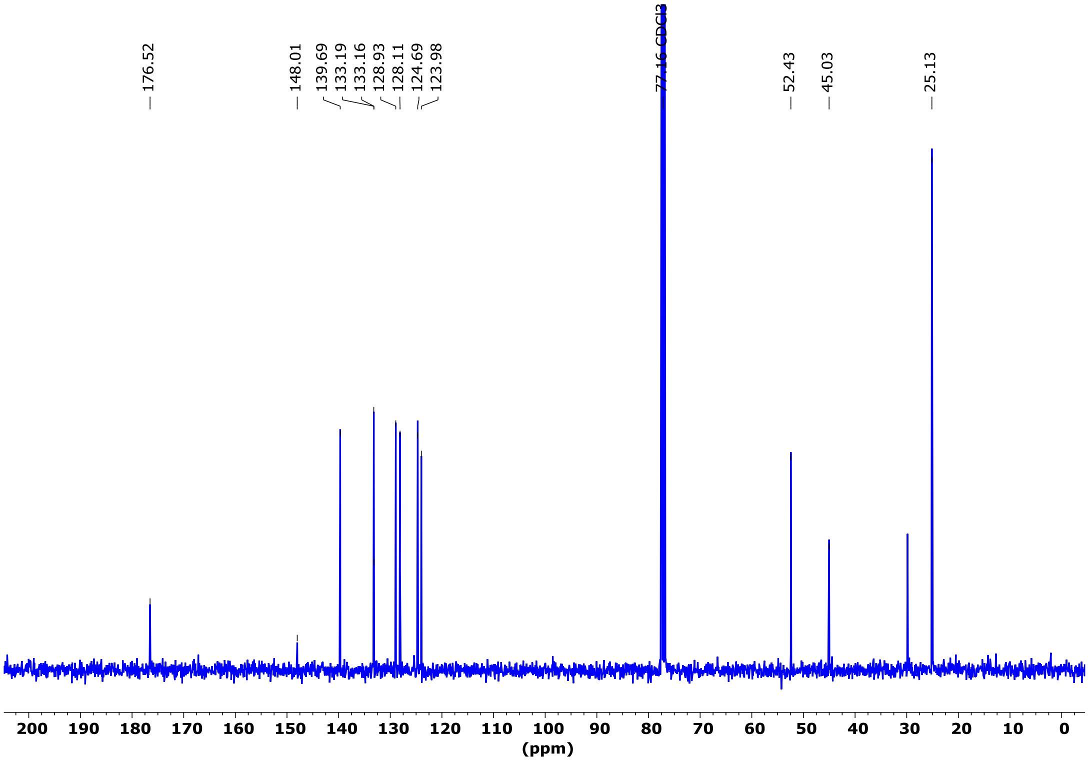


$^{13}C\{^{1}H\}$ NMR, $CDCl_3$, 100 MHz (ppm)

## Methyl 2,2-dimethyl-4-nitro-3-(2-nitrophenyl)butanoate (7b)

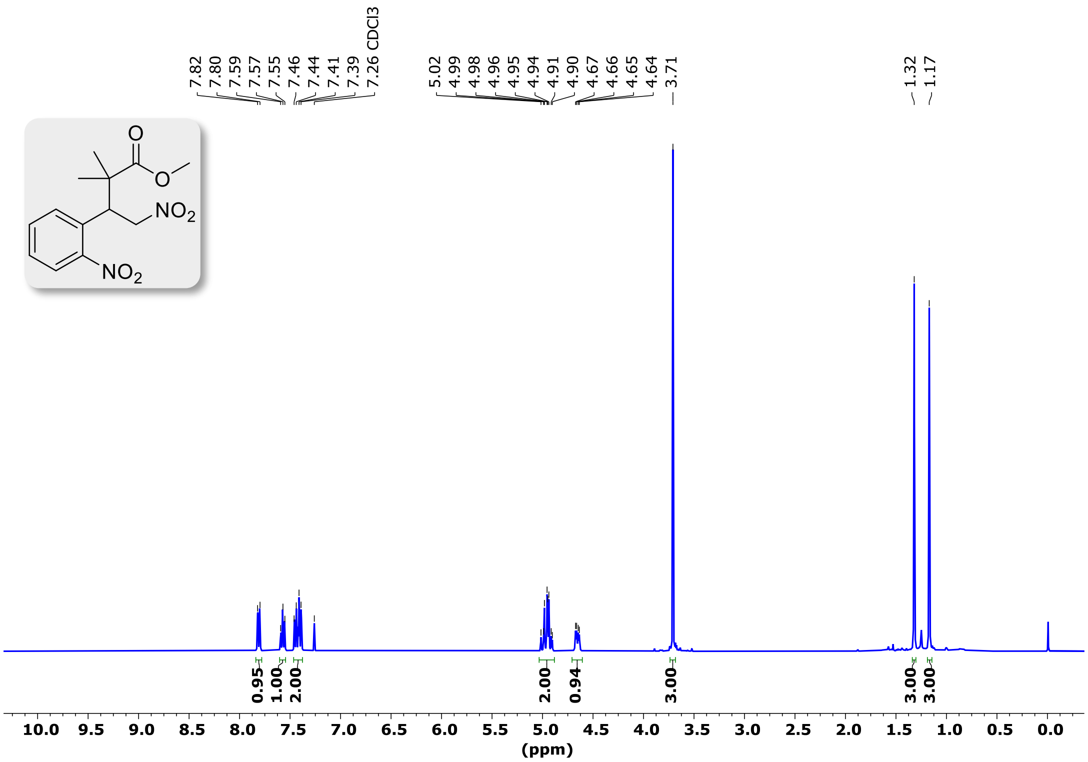


$^{1}H$ NMR, $CDCl_3$, 400 MHz (ppm)

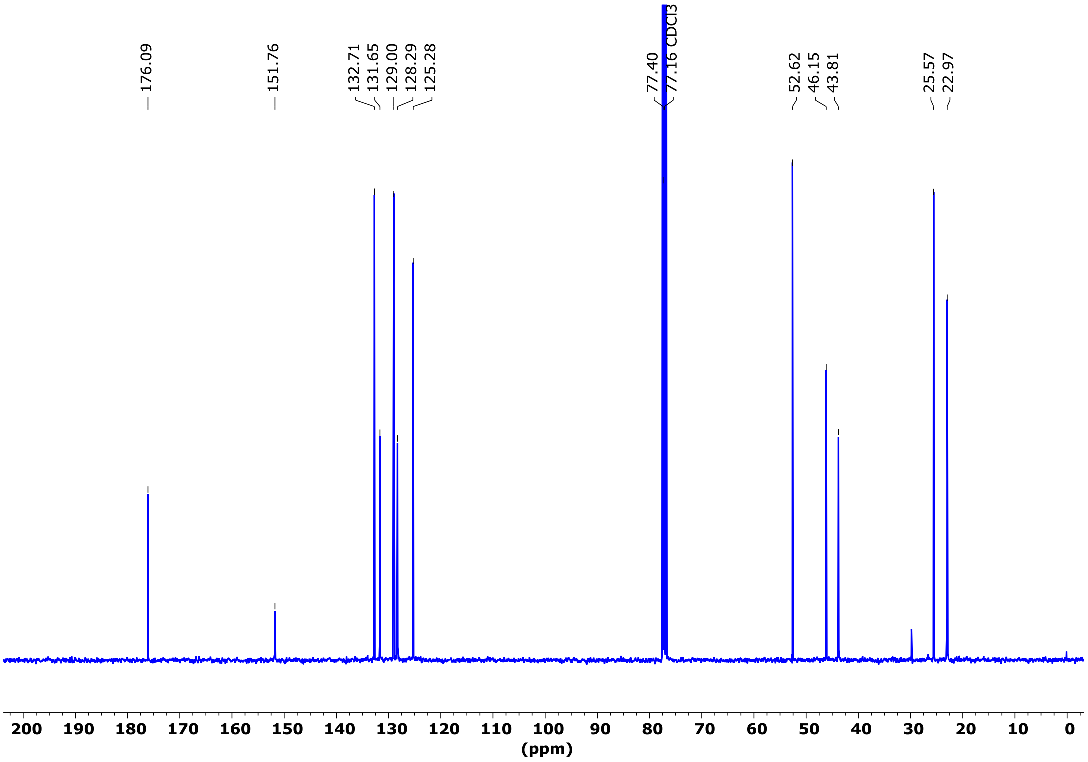


$^{13}C\{^{1}H\}$ NMR, $CDCl_3$, 100 MHz (ppm)

## Methyl (*E*)-2,2-dimethyl-4-(2-(trifluoromethyl)phenyl)but-3-enoate (6c)

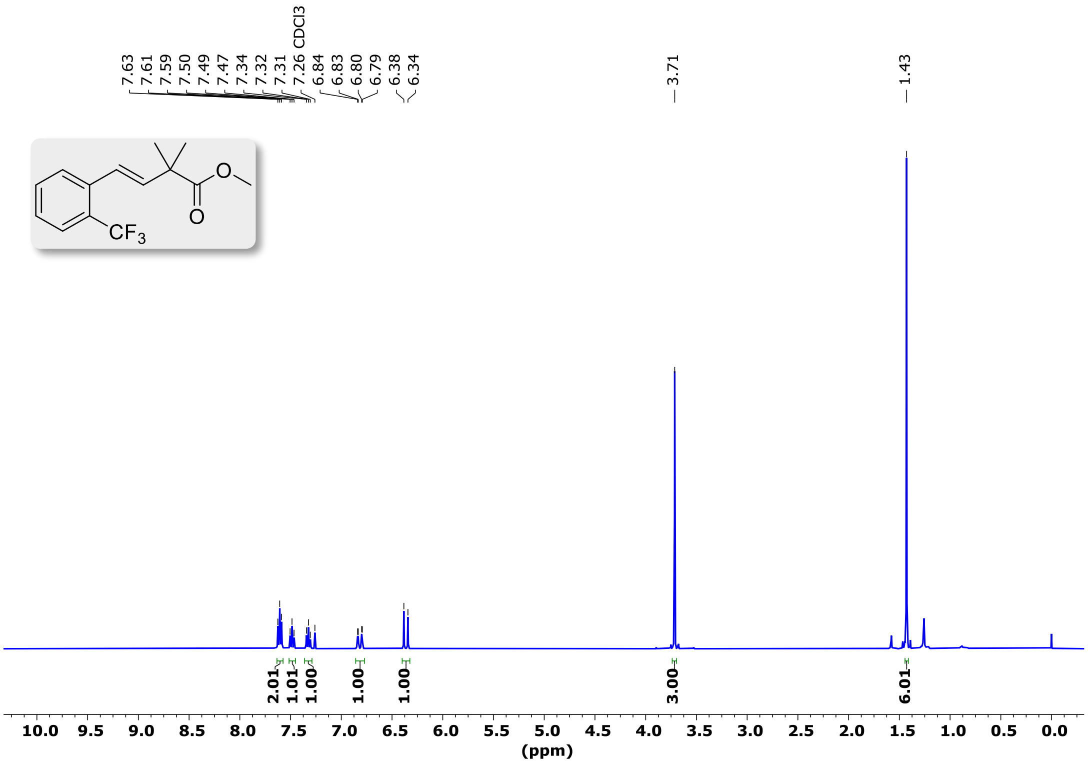


$^{1}H$ NMR, $CDCl_3$, 400 MHz (ppm)

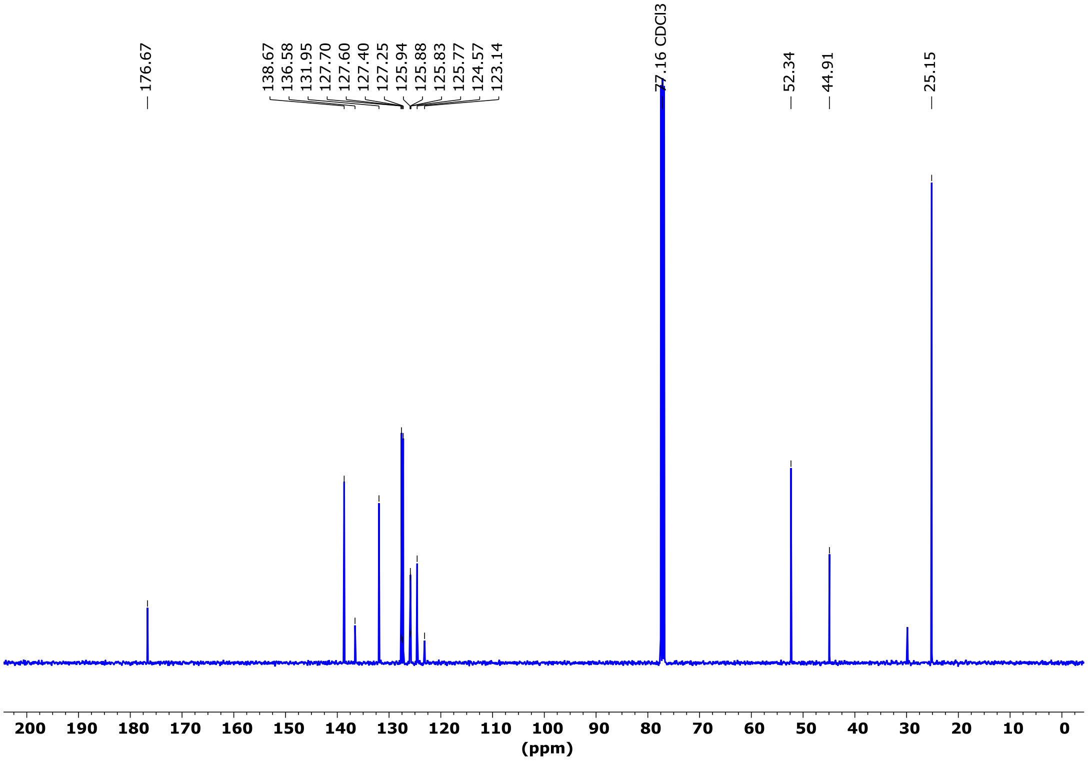


$^{13}C\{^{1}H\}$ NMR, $CDCl_3$, 100 MHz (ppm)

## Methyl 2,2-dimethyl-4-nitro-3-(2-(trifluoromethyl)phenyl)butanoate (7c)

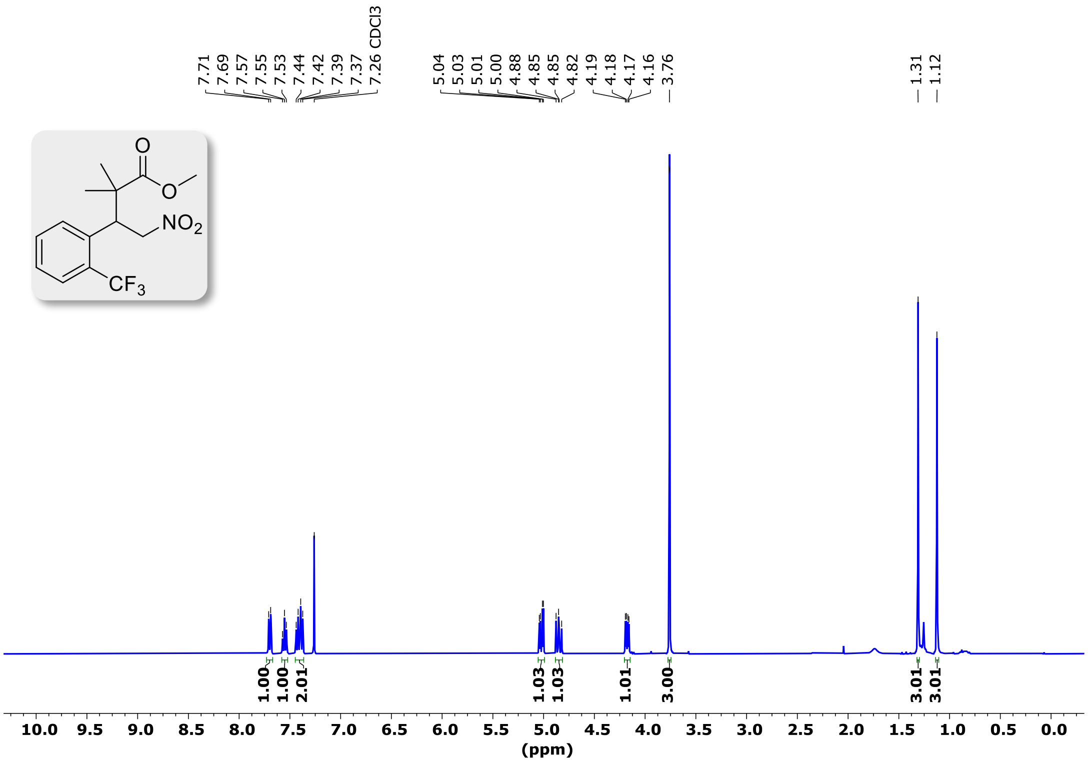


$^{1}$H NMR, $CDCl_3$, 400 MHz (ppm)

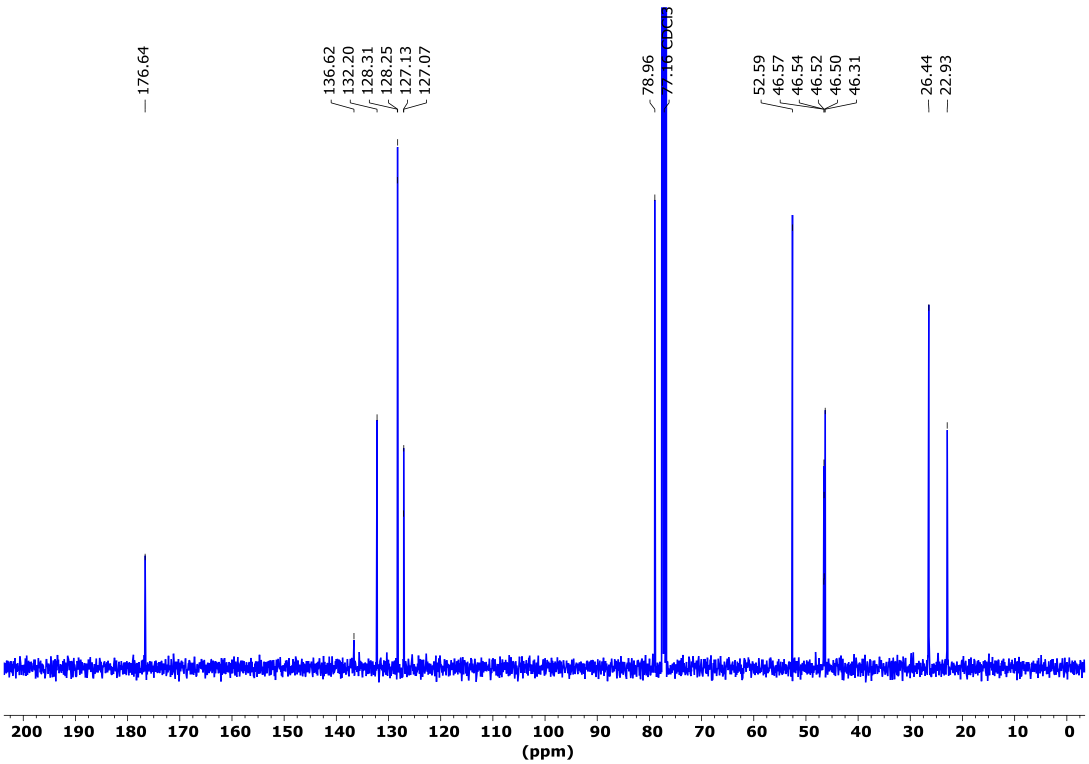


$^{13}$C{$^{1}$H} NMR, $CDCl_3$, 100 MHz (ppm)

## Methyl (*E*)-4-(2-bromophenyl)-2,2-dimethylbut-3-enoate (6d)

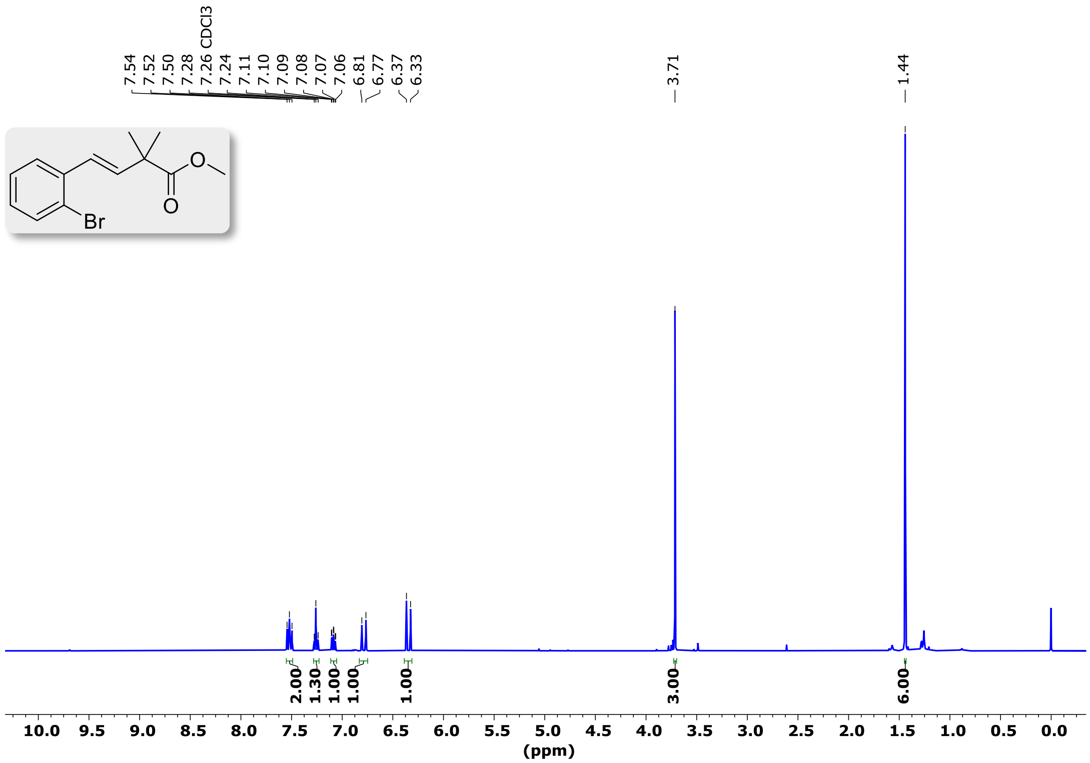


$^{1}H$ NMR, $CDCl_3$, 400 MHz (ppm)

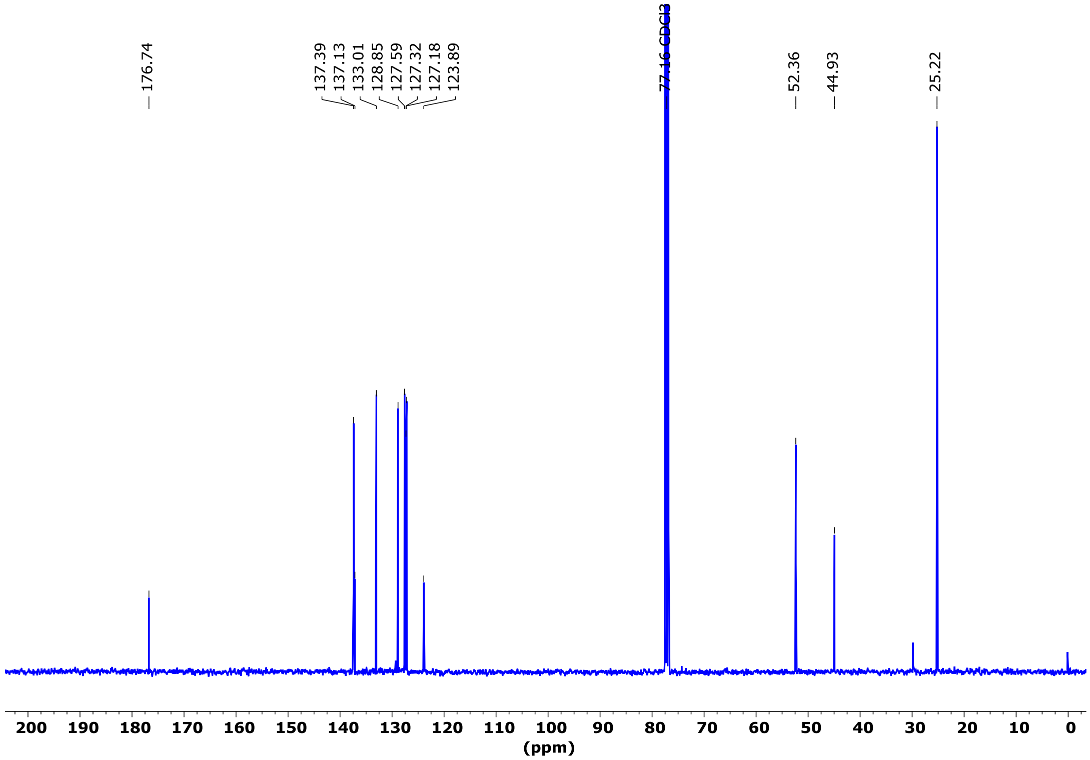


$^{13}C\{^{1}H\}$ NMR, $CDCl_3$, 100 MHz (ppm)

## Methyl 3-(2-bromophenyl)-2,2-dimethyl-4-nitrobutanoate (7d)

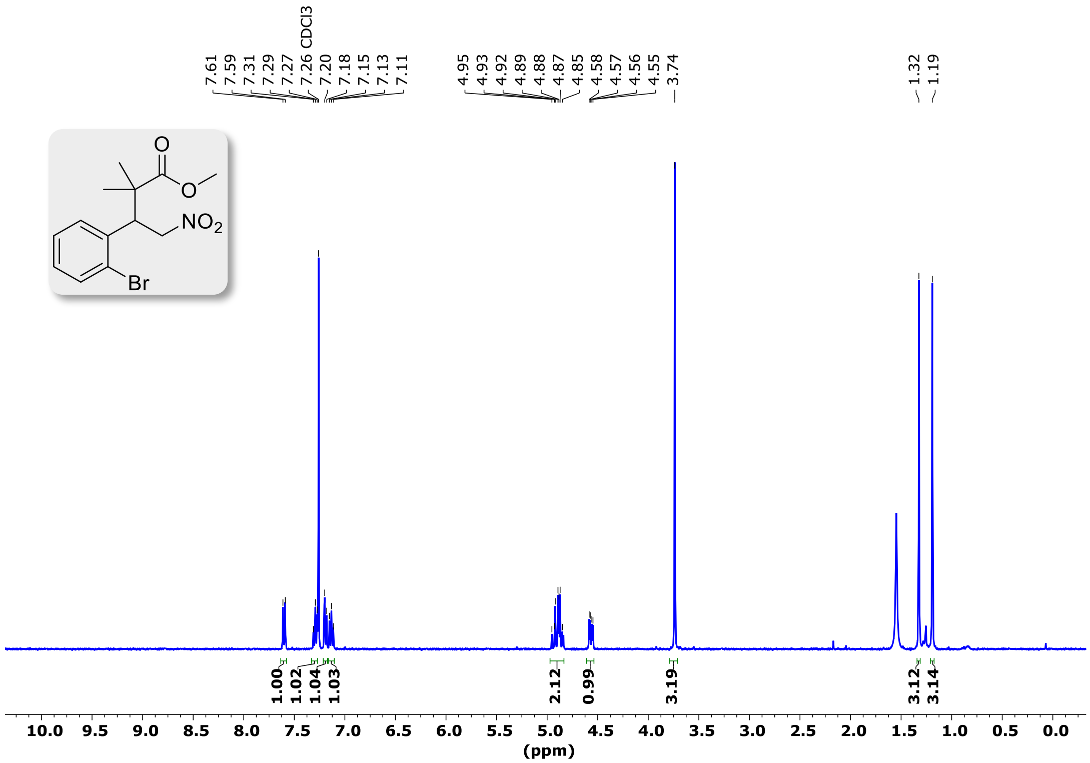


$^{1}H$ NMR, $CDCl_3$, 400 MHz (ppm)

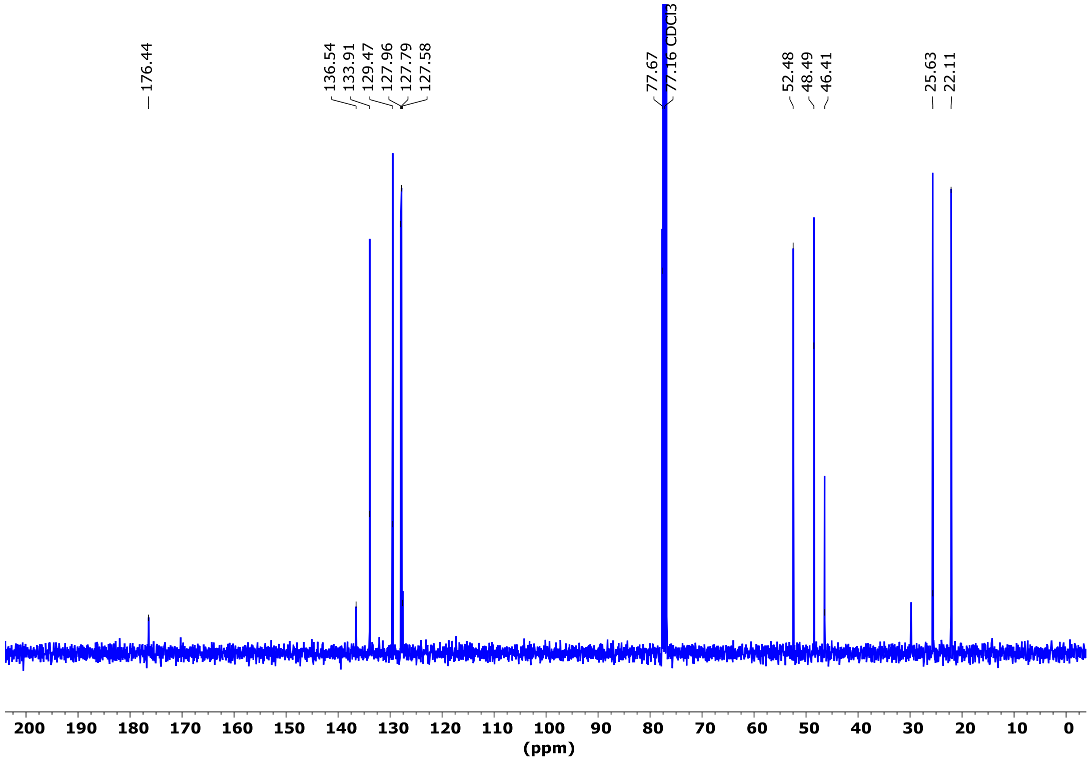


$^{13}C\{^{1}H\}$ NMR, $CDCl_3$, 100 MHz (ppm)

# Methyl 2,2-dimethyl-4-nitro-3-(o-tolyl)butanoate (7e)

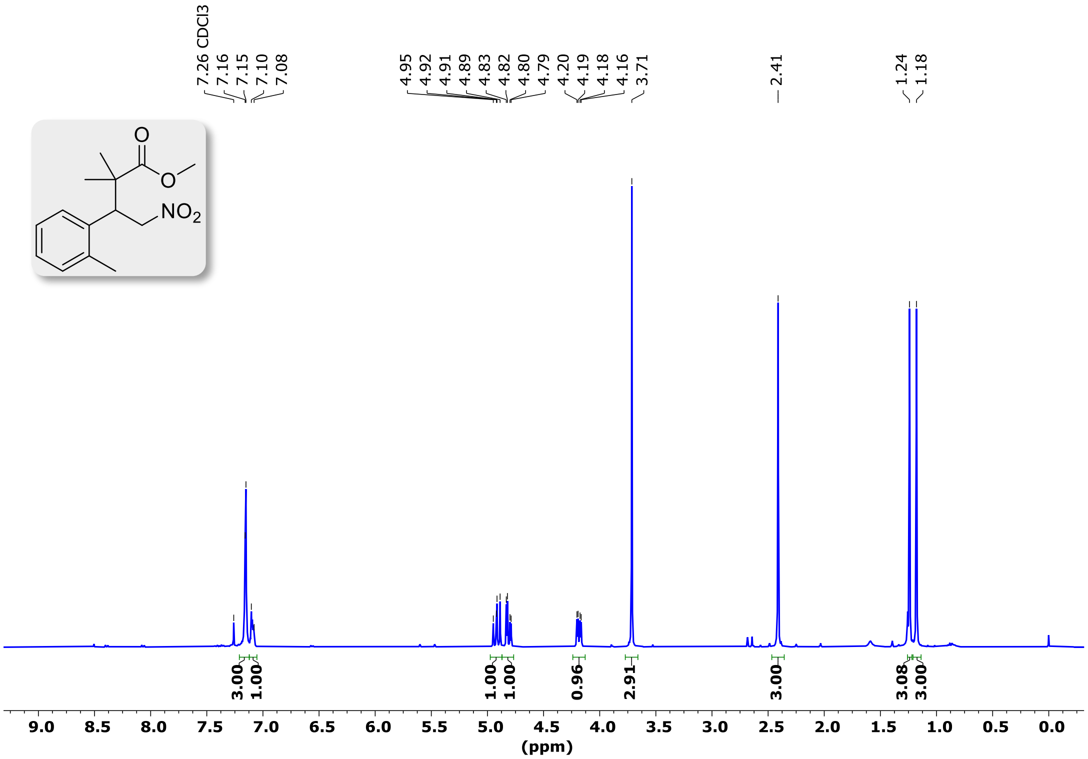


$^{1}H$ NMR, $CDCl_3$, 400 MHz (ppm)

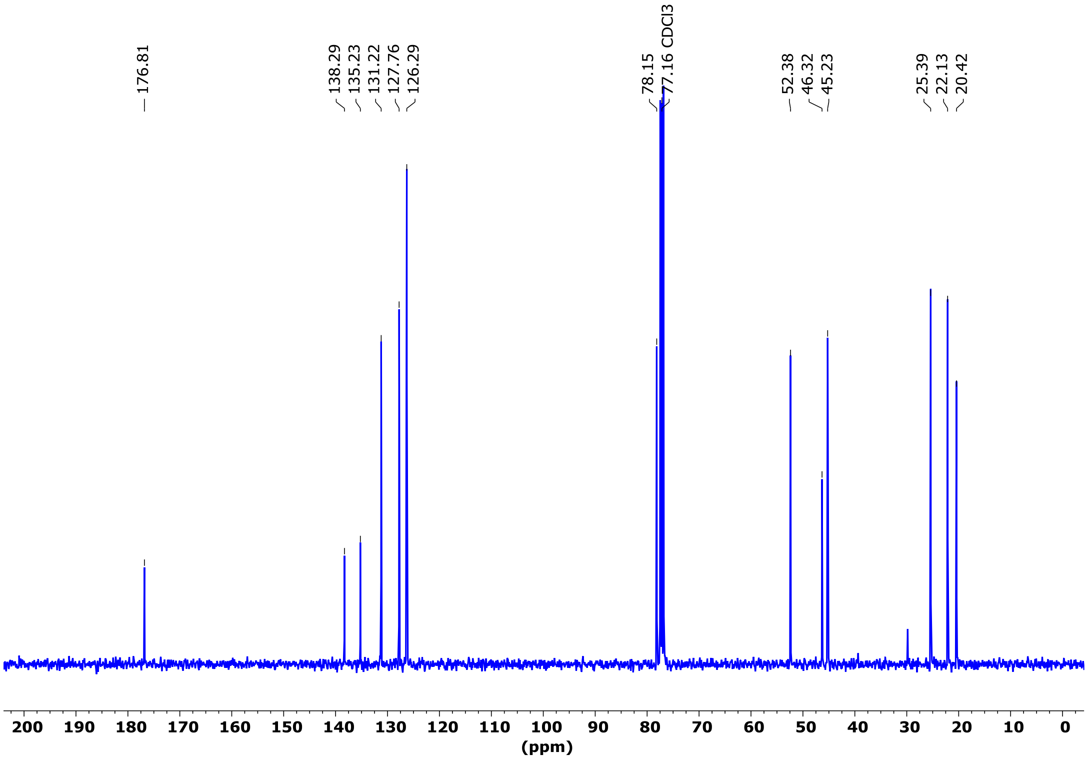


$^{13}C\{^{1}H\}$ NMR, $CDCl_3$, 100 MHz (ppm)

**Methyl 3-(2-methoxyphenyl)-2,2-dimethyl-4-nitrobutanoate (7f)**

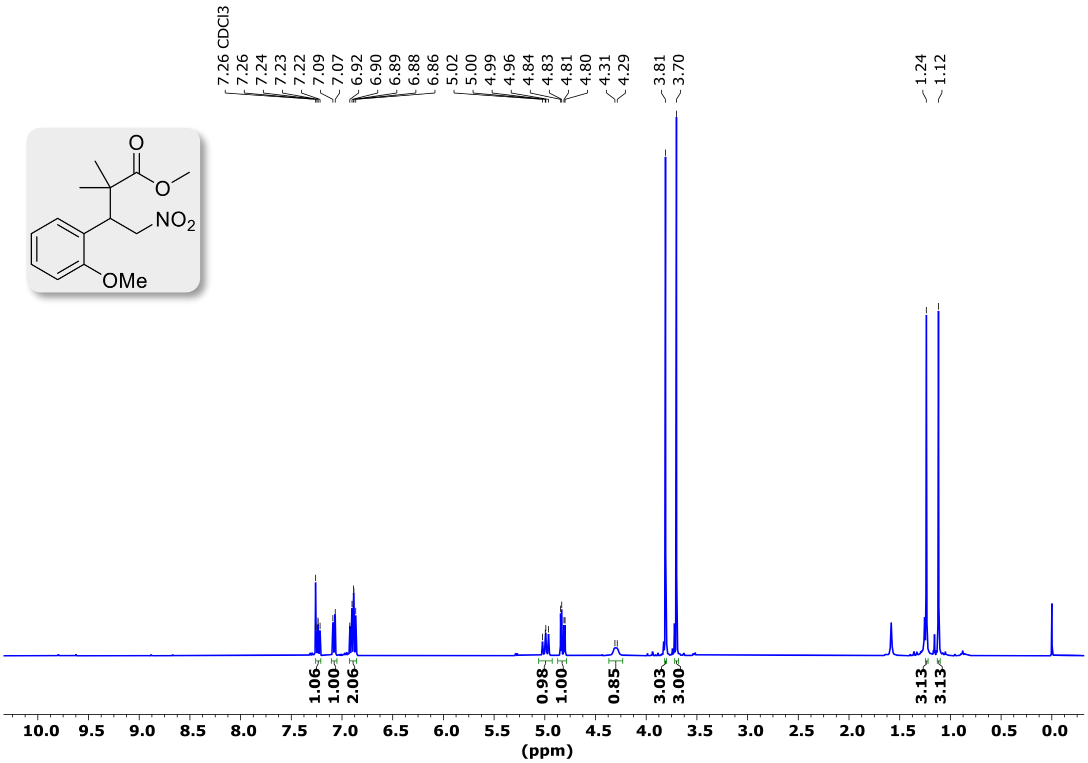


$^1$H NMR, $CDCl_3$, 400 MHz (ppm)

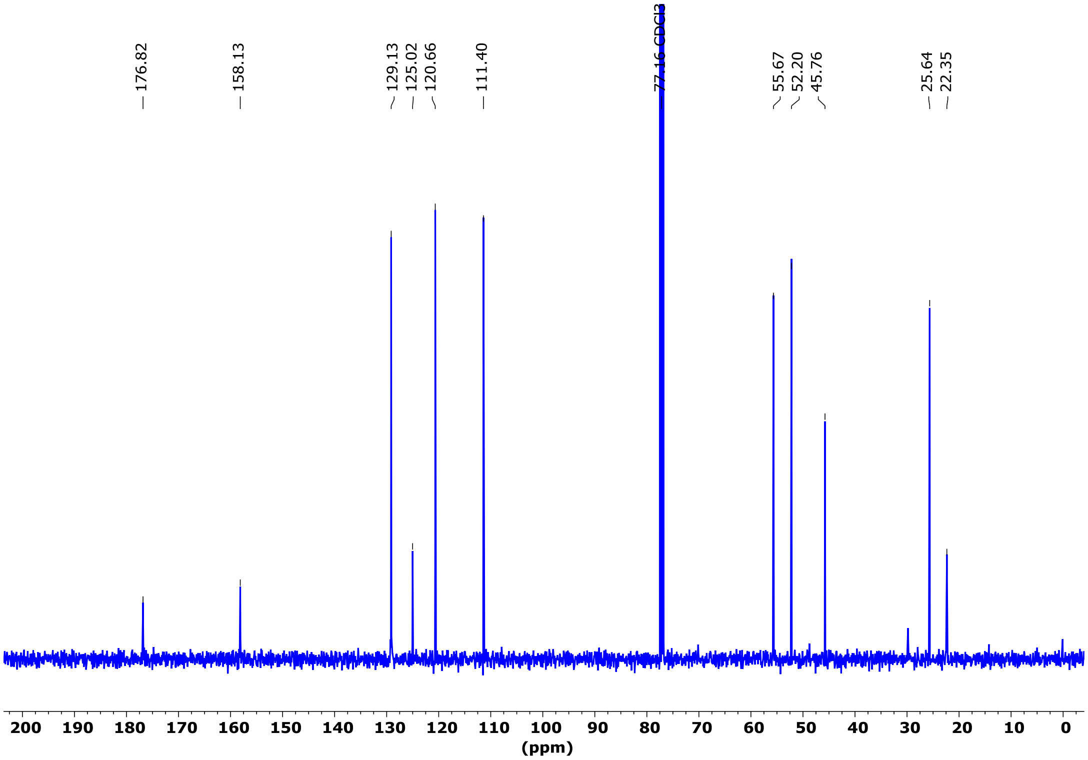


$^{13}$C{$^1$H} NMR, $CDCl_3$, 100 MHz (ppm)

## Methyl (*E*)-2,2-dimethyl-4-(3-nitrophenyl)but-3-enoate (6g)

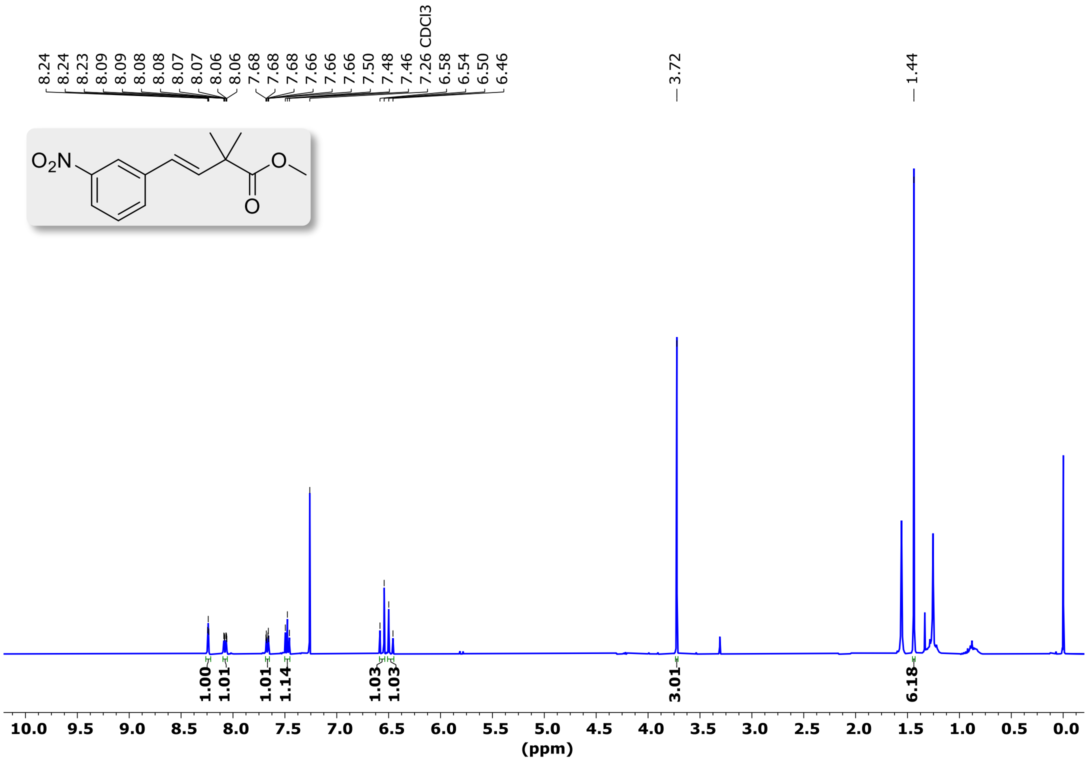


$^{1}$H NMR, CDCl$_3$, 400 MHz (ppm)

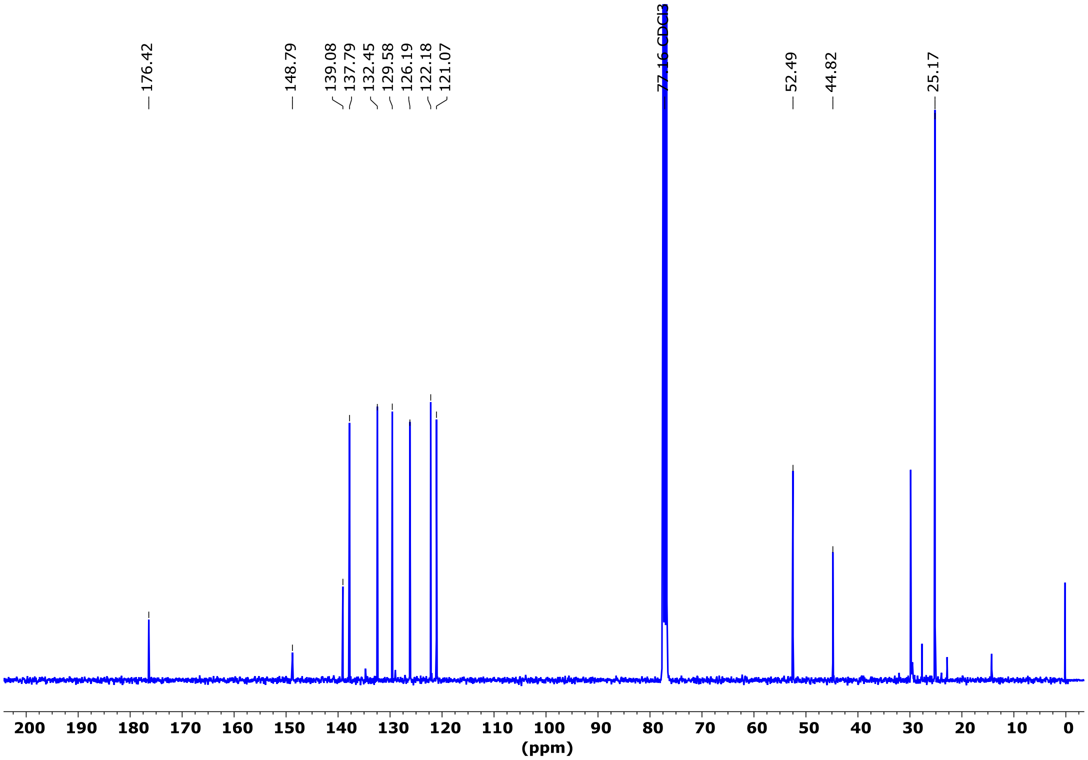


$^{13}$C{$^{1}$H} NMR, CDCl$_3$, 100 MHz (ppm)

## Methyl 2,2-dimethyl-4-nitro-3-(3-nitrophenyl)butanoate (7g)

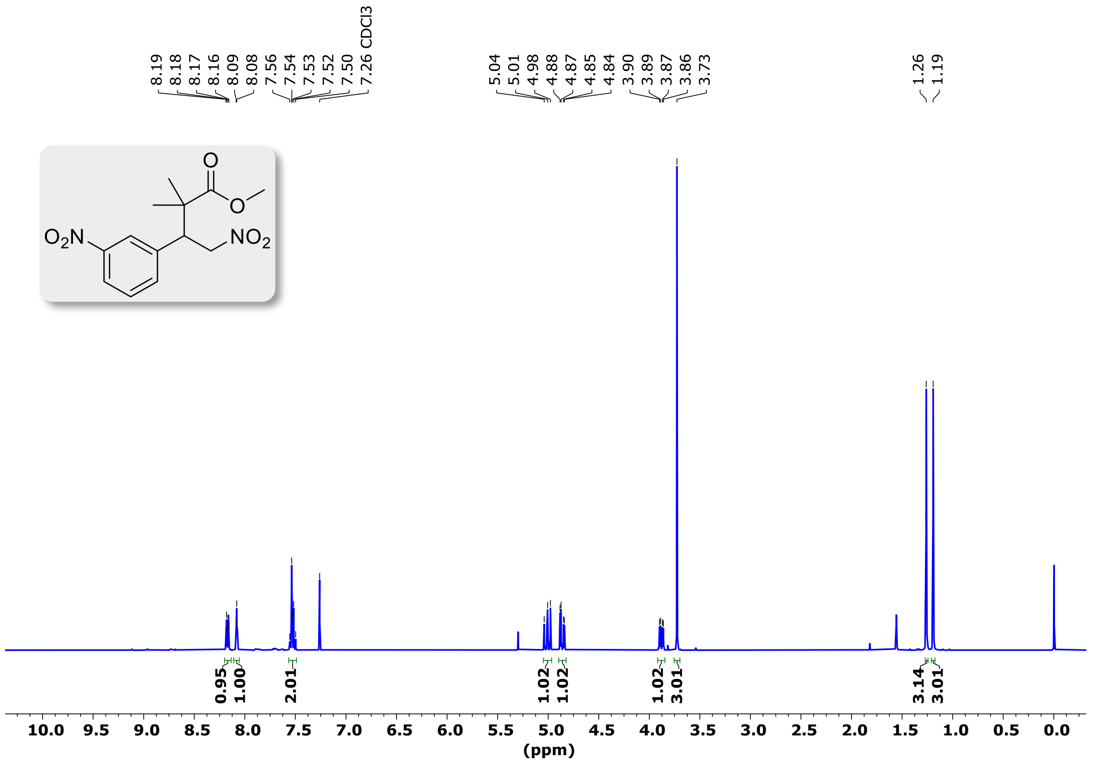


**$^1H$ NMR, $CDCl_3$, 400 MHz (ppm)**

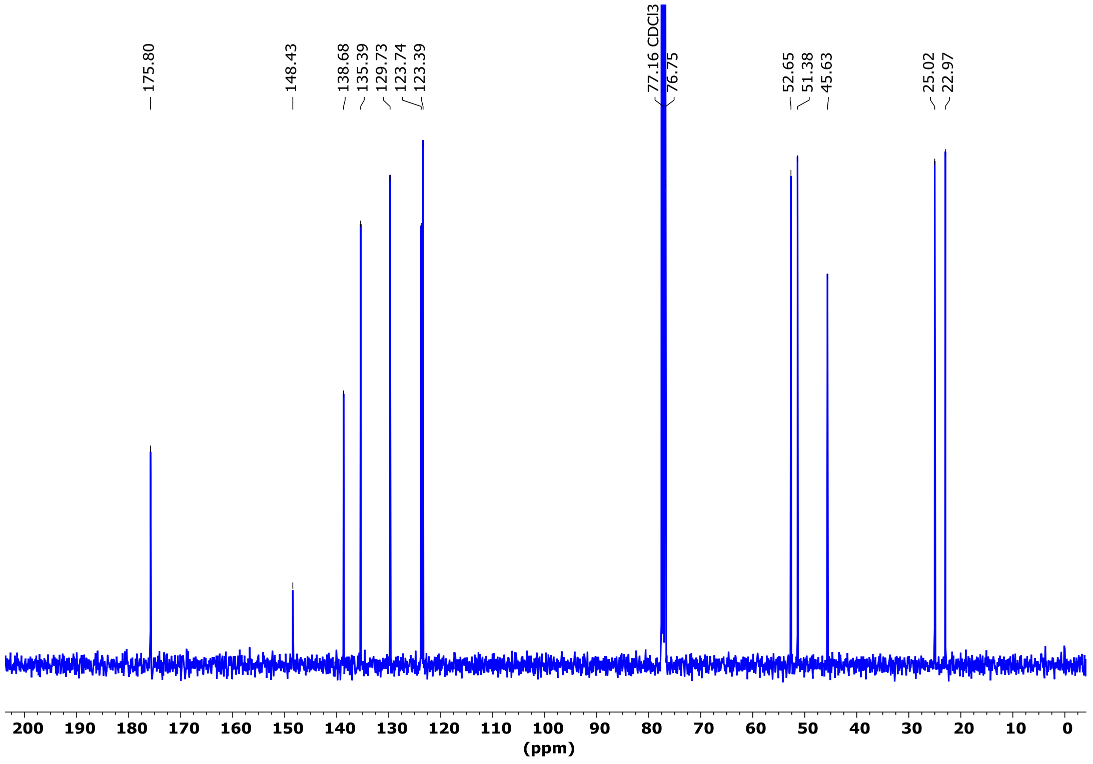


**$^{13}C\{^1H\}$ NMR, $CDCl_3$, 100 MHz (ppm)**

**Methyl (*E*)-4-(3-cyanophenyl)-2,2-dimethylbut-3-enoate (6h)**

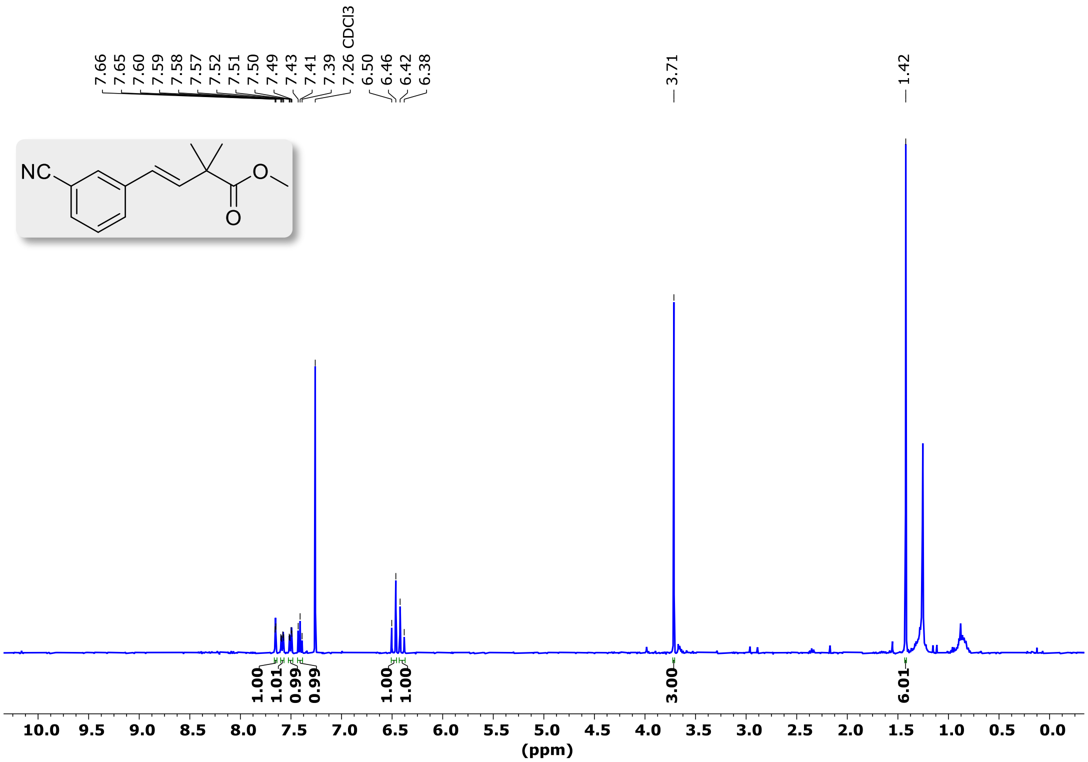


$^{1}H$ NMR, $CDCl_3$, 400 MHz (ppm)

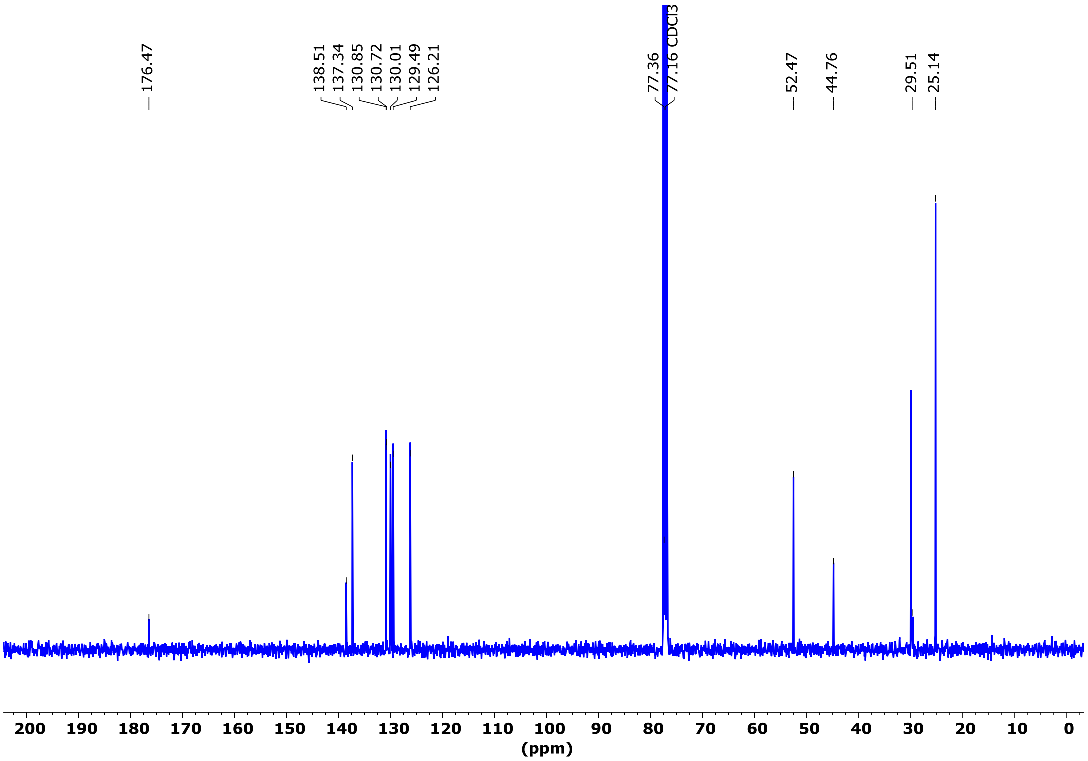


$^{13}C\{^{1}H\}$ NMR, $CDCl_3$, 100 MHz (ppm)

## Methyl 3-(3-cyanophenyl)-2,2-dimethyl-4-nitrobutanoate (7h)

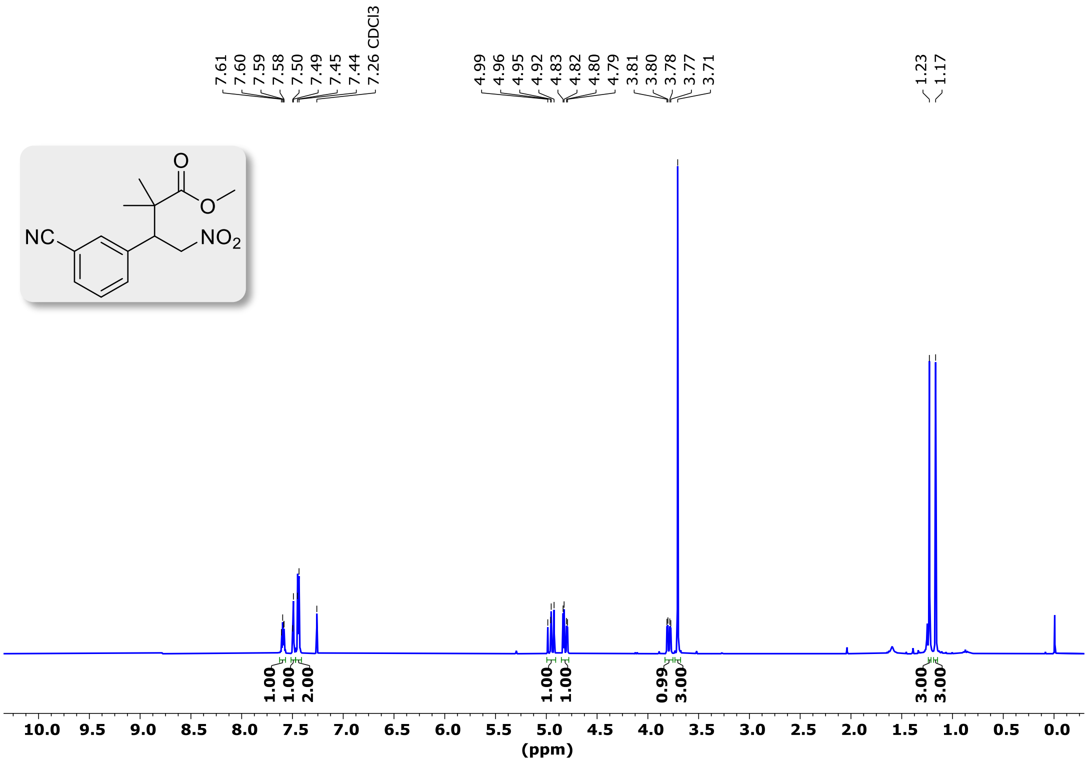


$^{1}$H NMR, $CDCl_3$, 400 MHz (ppm)

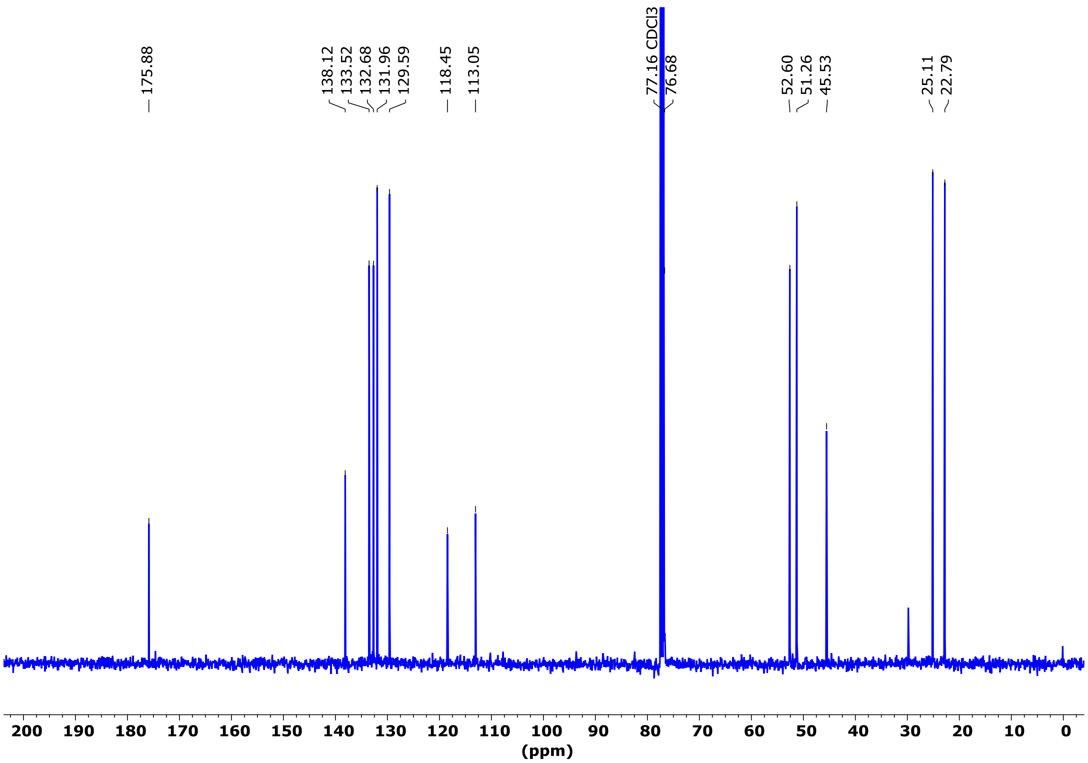


$^{13}C\{^{1}H\}$ NMR, $CDCl_3$, 100 MHz (ppm)

## Methyl (*E*)-2,2-dimethyl-4-(4-nitrophenyl)but-3-enoate (6i)

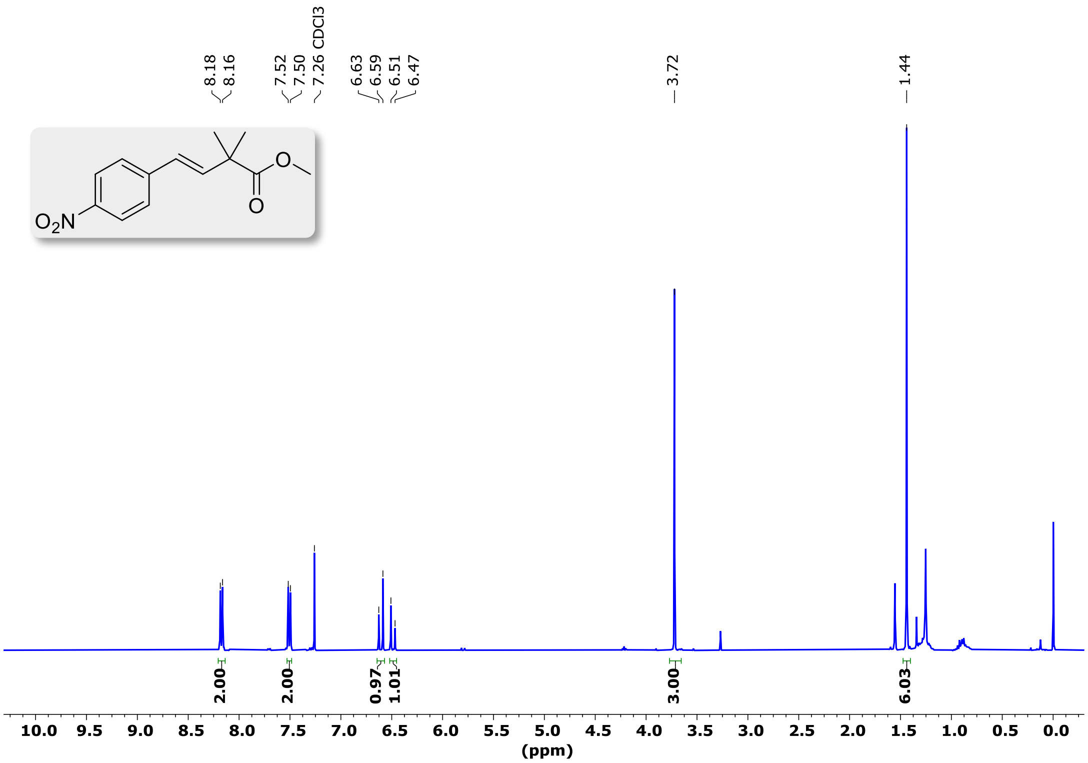


$^{1}H$ NMR, $CDCl_3$, 400 MHz (ppm)

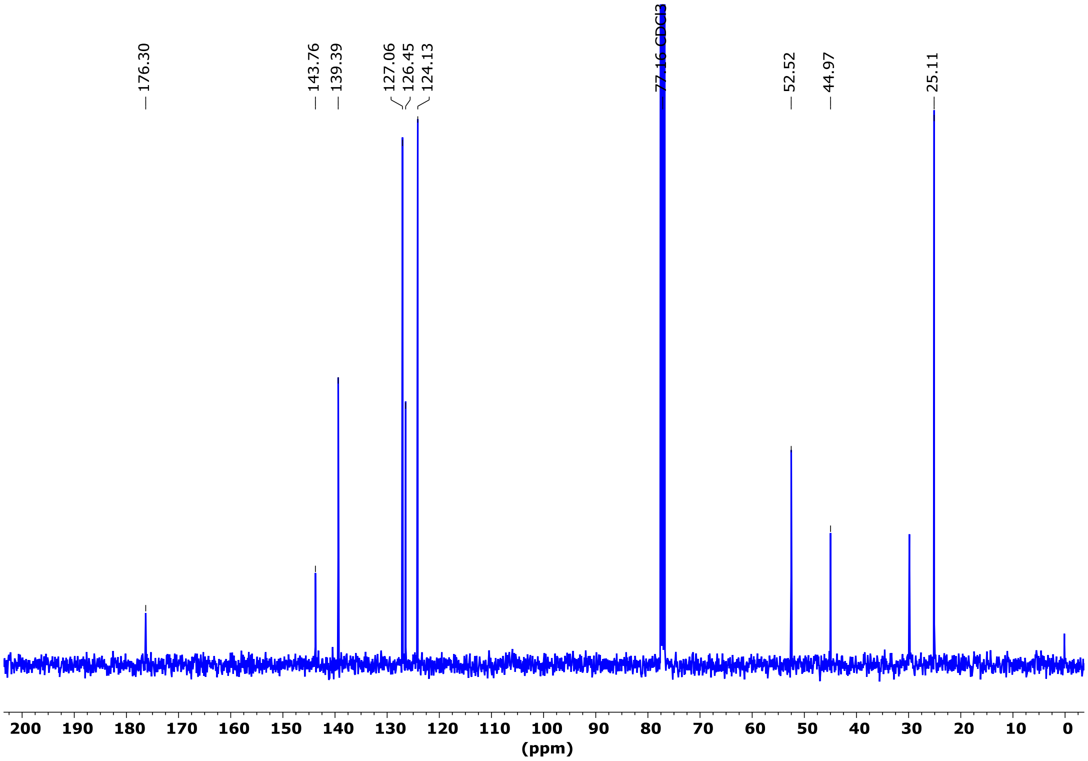


$^{13}C\{^{1}H\}$ NMR, $CDCl_3$, 100 MHz (ppm)

**Methyl 2,2-dimethyl-4-nitro-3-(4-nitrophenyl)butanoate (7i)**

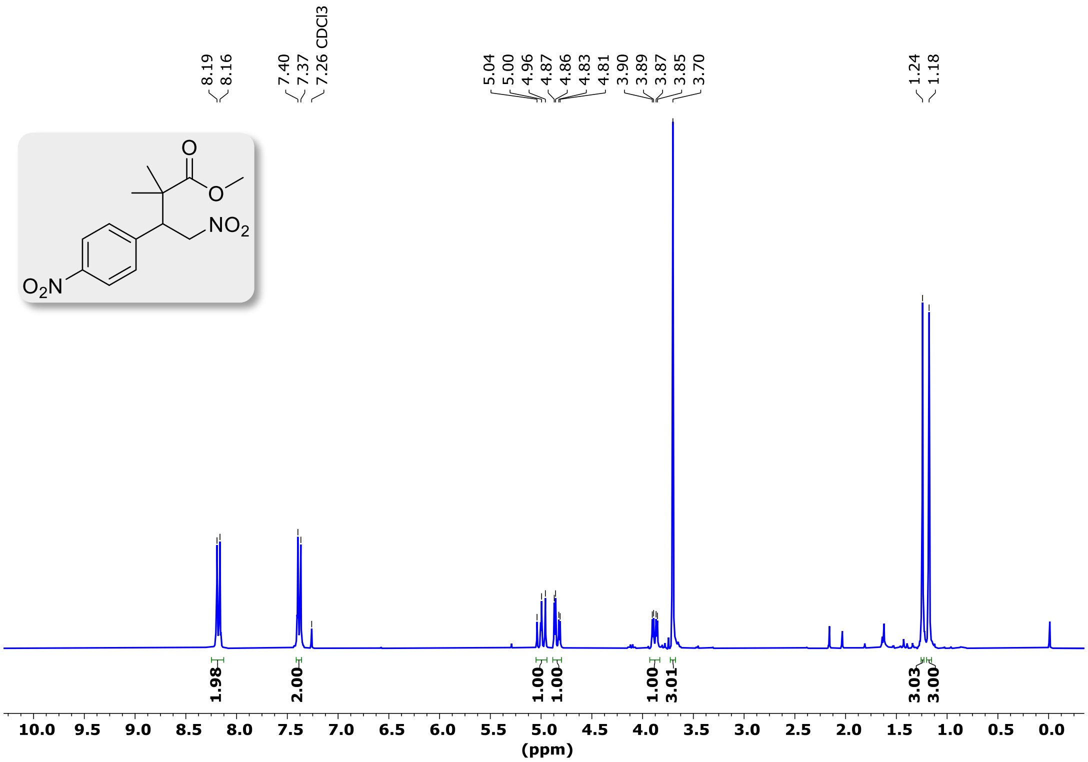


$^1H$ NMR, $CDCl_3$, 400 MHz (ppm)

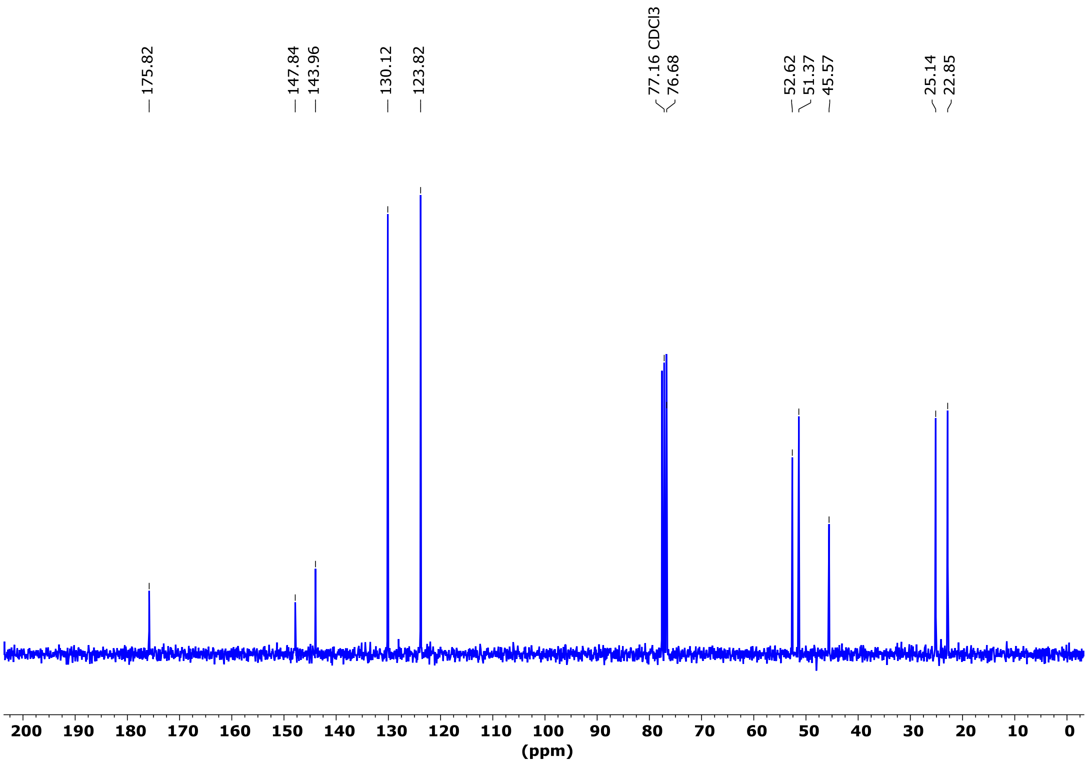


$^{13}C\{^1H\}$ NMR, $CDCl_3$, 100 MHz (ppm)

**Methyl (*E*)-4-(4-cyanophenyl)-2,2-dimethylbut-3-enoate (6j)**

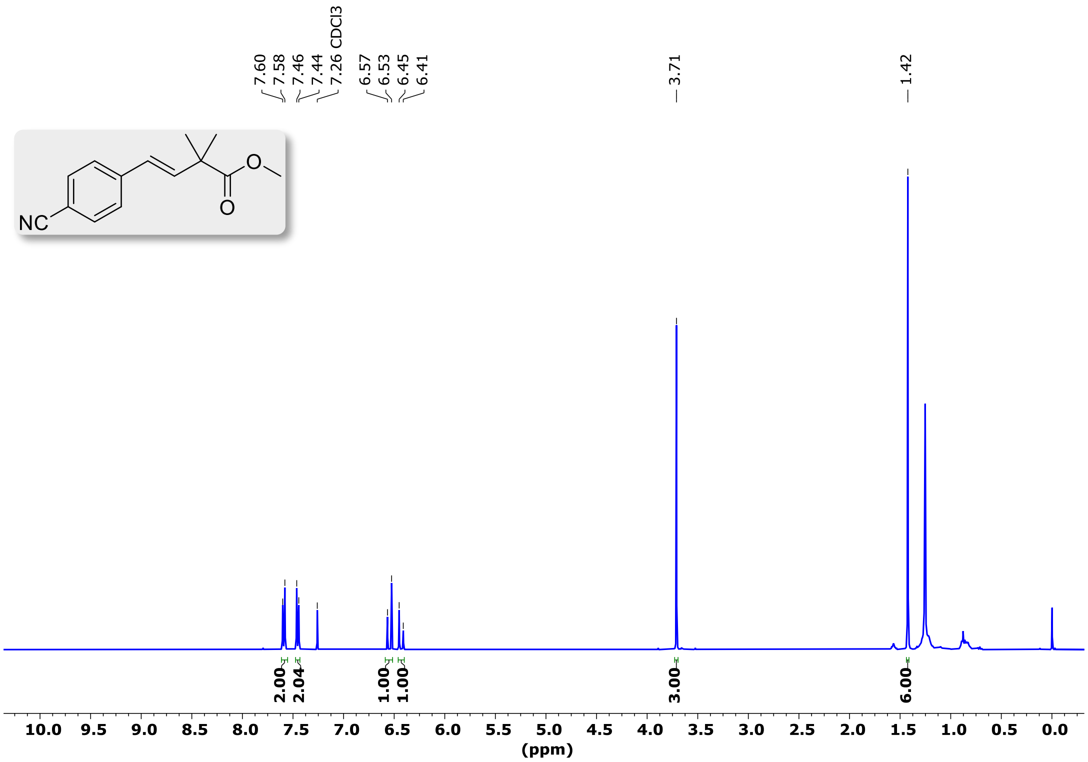


$^{1}H$ NMR, $CDCl_3$, 400 MHz (ppm)

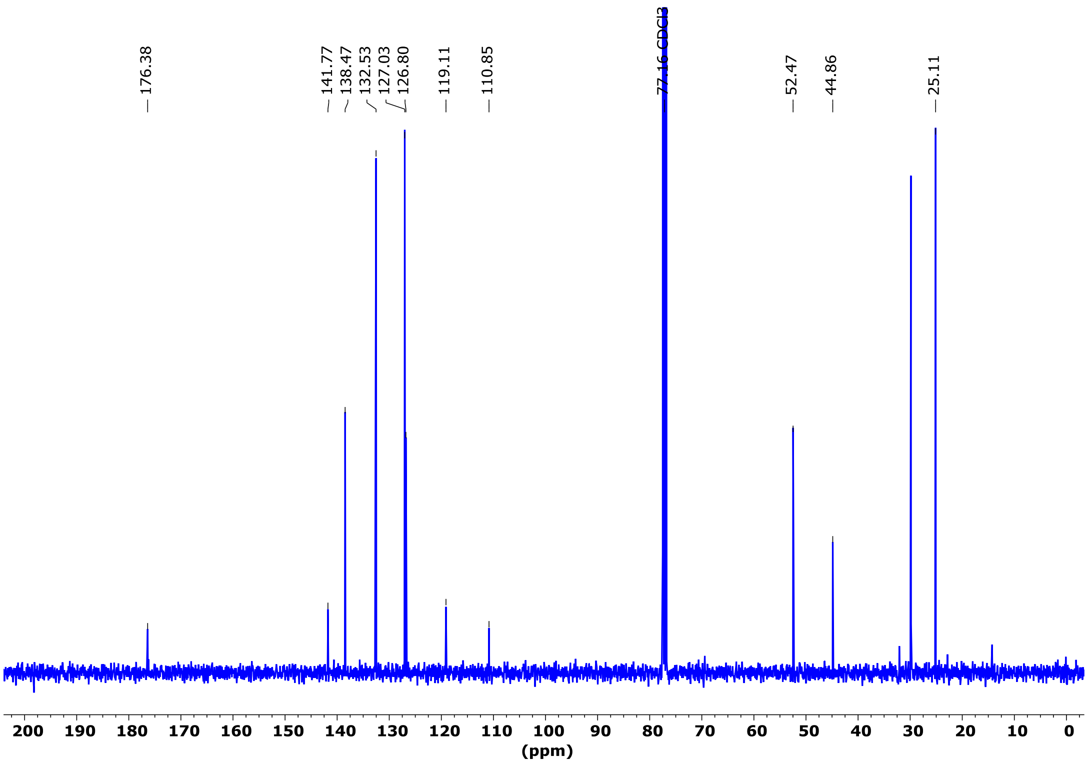


$^{13}C\{^{1}H\}$ NMR, $CDCl_3$, 100 MHz (ppm)

## Methyl 3-(4-cyanophenyl)-2,2-dimethyl-4-nitrobutanoate (7j)

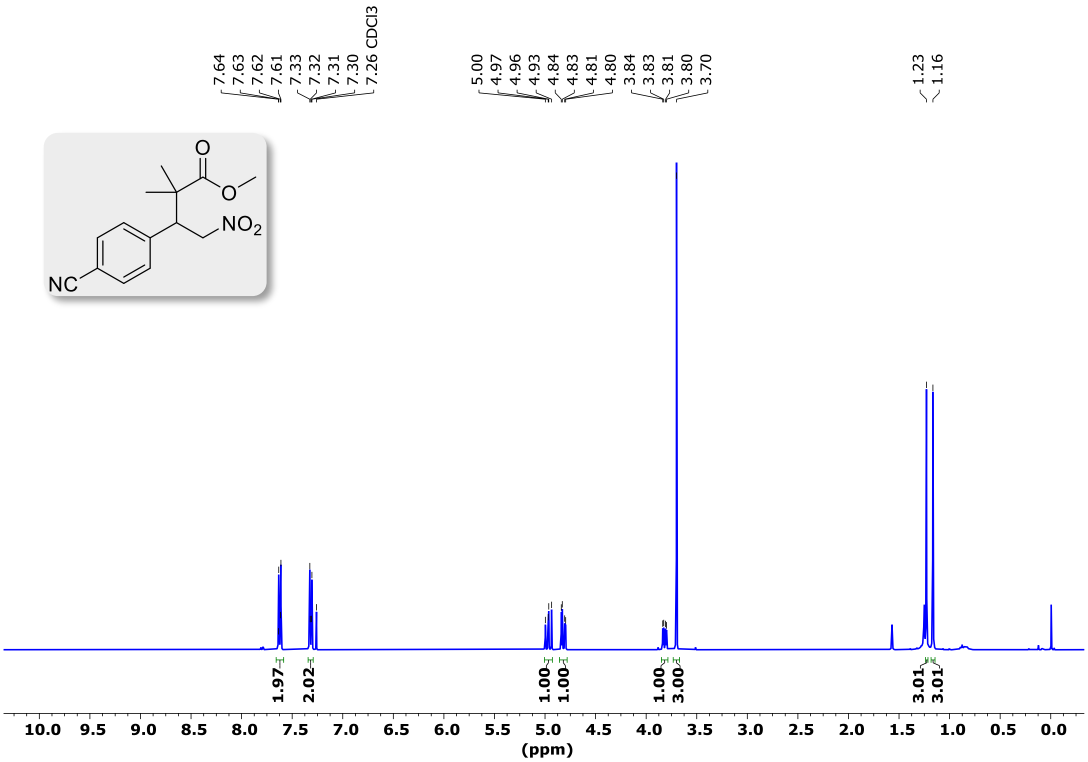


$^1$H NMR, $CDCl_3$, 400 MHz (ppm)

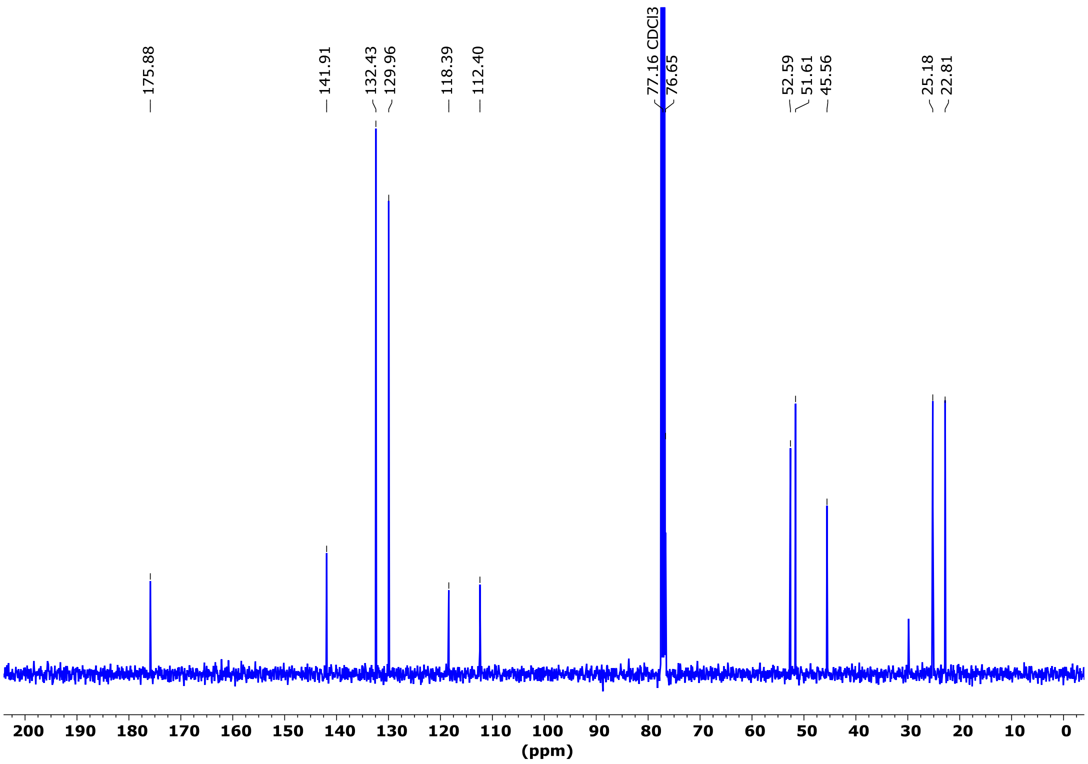


$^{13}$C{$^1$H} NMR, $CDCl_3$, 100 MHz (ppm)

## Methyl 3-(4-chlorophenyl)-2,2-dimethyl-4-nitrobutanoate (7k)

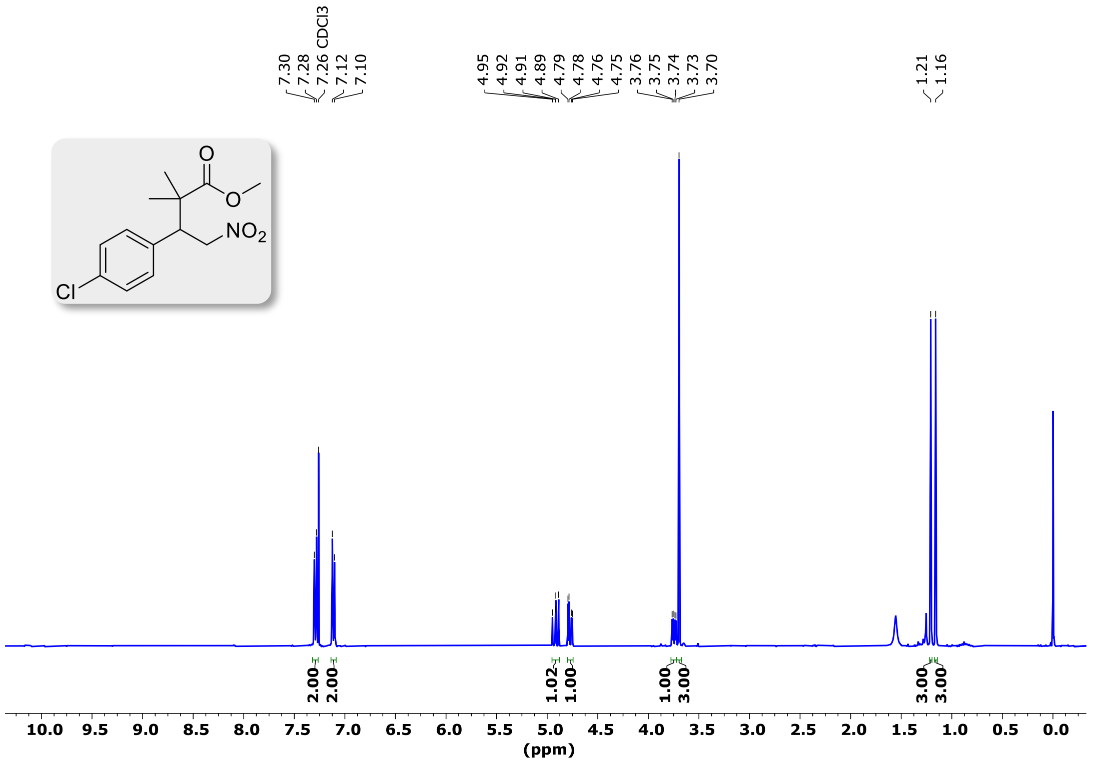


$^{1}H$ NMR, $CDCl_3$, 400 MHz (ppm)

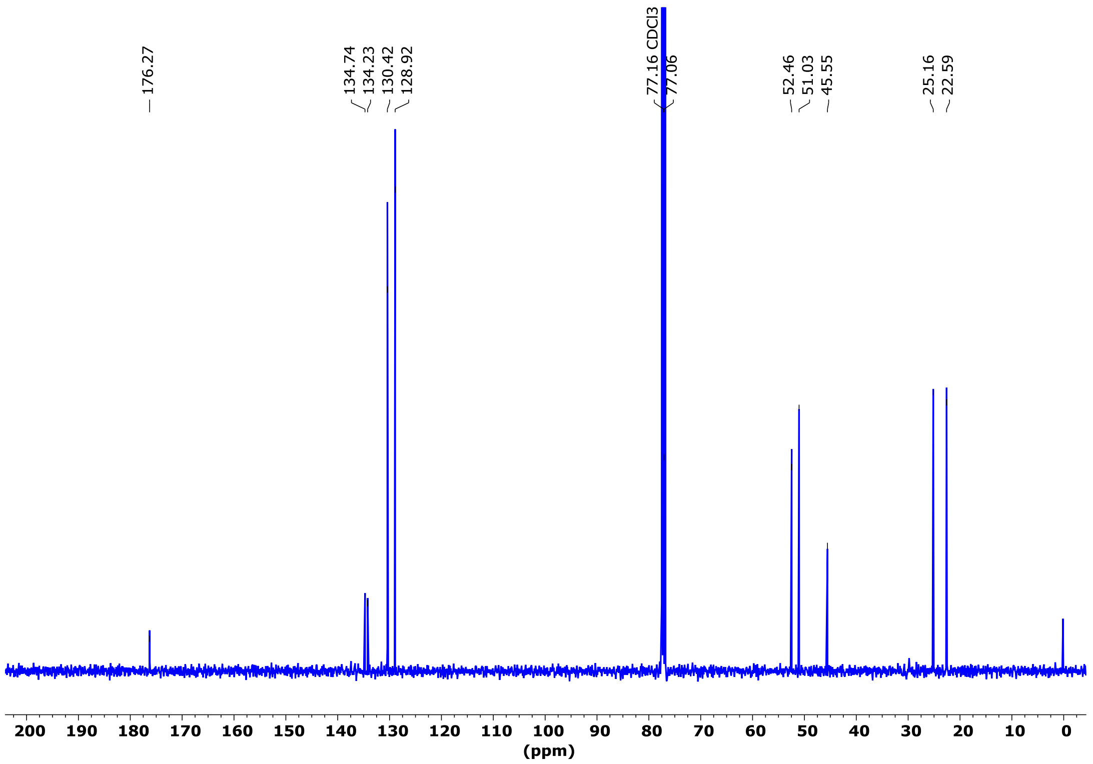


$^{13}C\{^{1}H\}$ NMR, $CDCl_3$, 100 MHz (ppm)

## Methyl 3-(4-bromophenyl)-2,2-dimethyl-4-nitrobutanoate (7l)

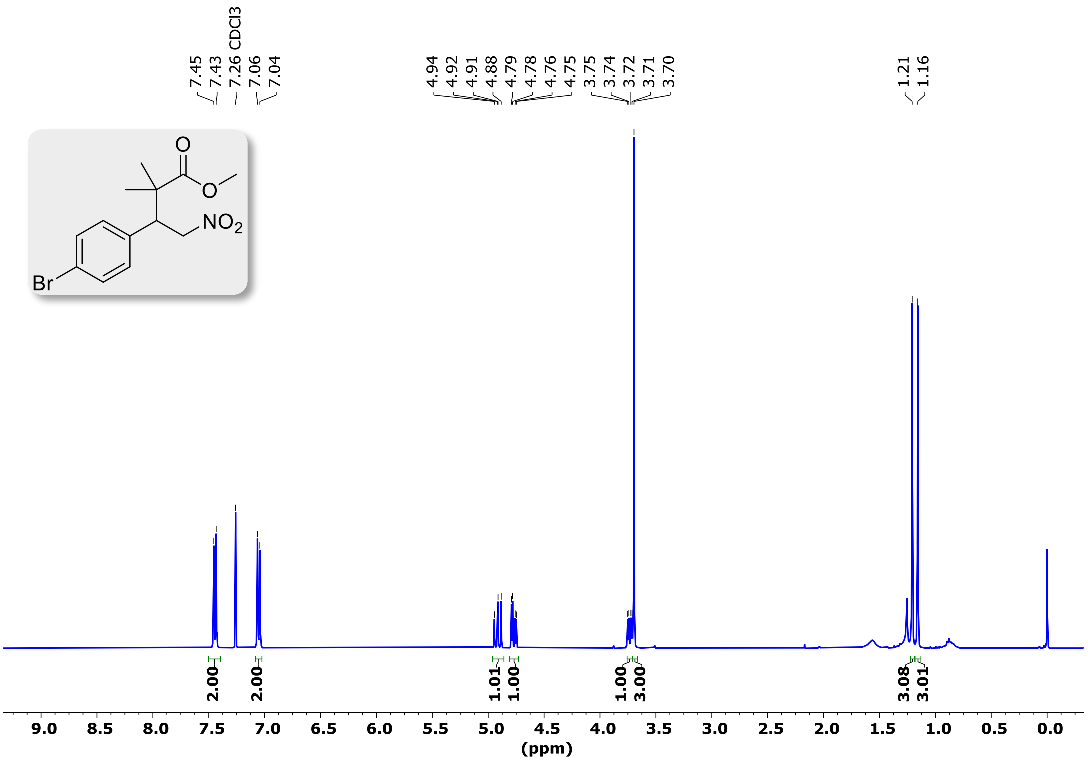


$^{1}H$ NMR, $CDCl_3$, 400 MHz (ppm)

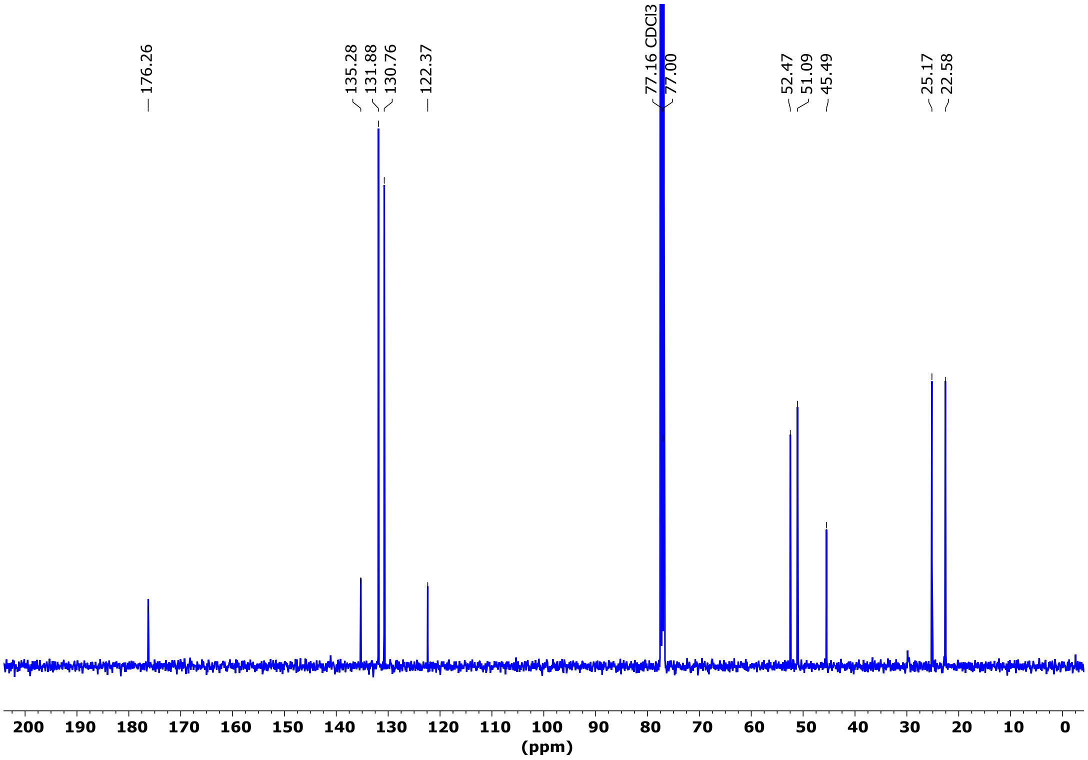


$^{13}C\{^{1}H\}$ NMR, $CDCl_3$, 100 MHz (ppm)

## Methyl 2,2-dimethyl-4-nitro-3-(4-(phenylethynyl)phenyl)butanoate (7m)

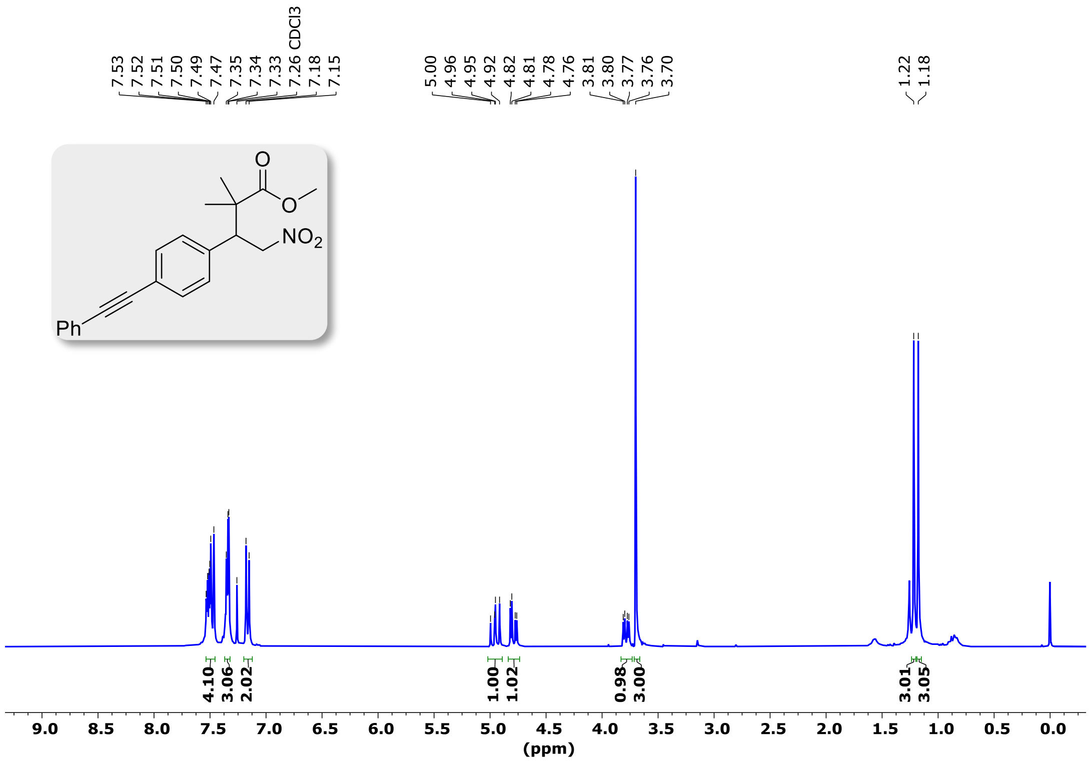


$^{1}H$ NMR, $CDCl_3$, 400 MHz (ppm)

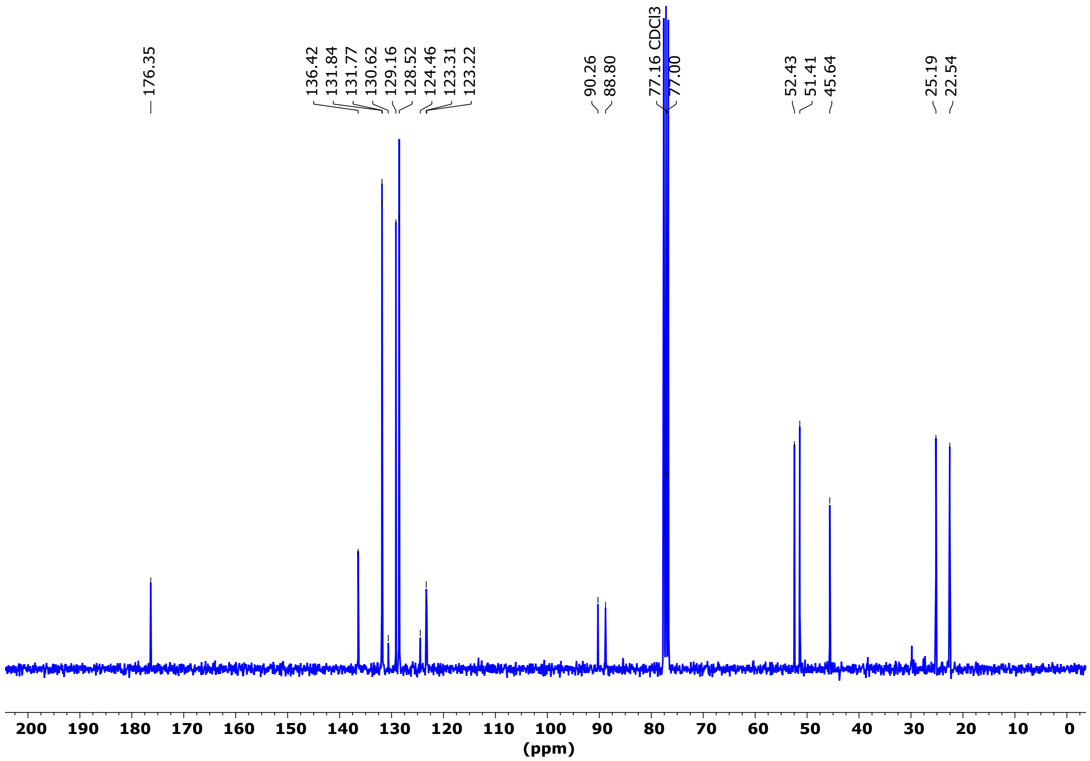


$^{13}C\{^{1}H\}$ NMR, $CDCl_3$, 100 MHz (ppm)

## Methyl (*E*)-4-(4-methoxy-3,3-dimethyl-4-oxobut-1-en-1-yl)benzoate (6n)

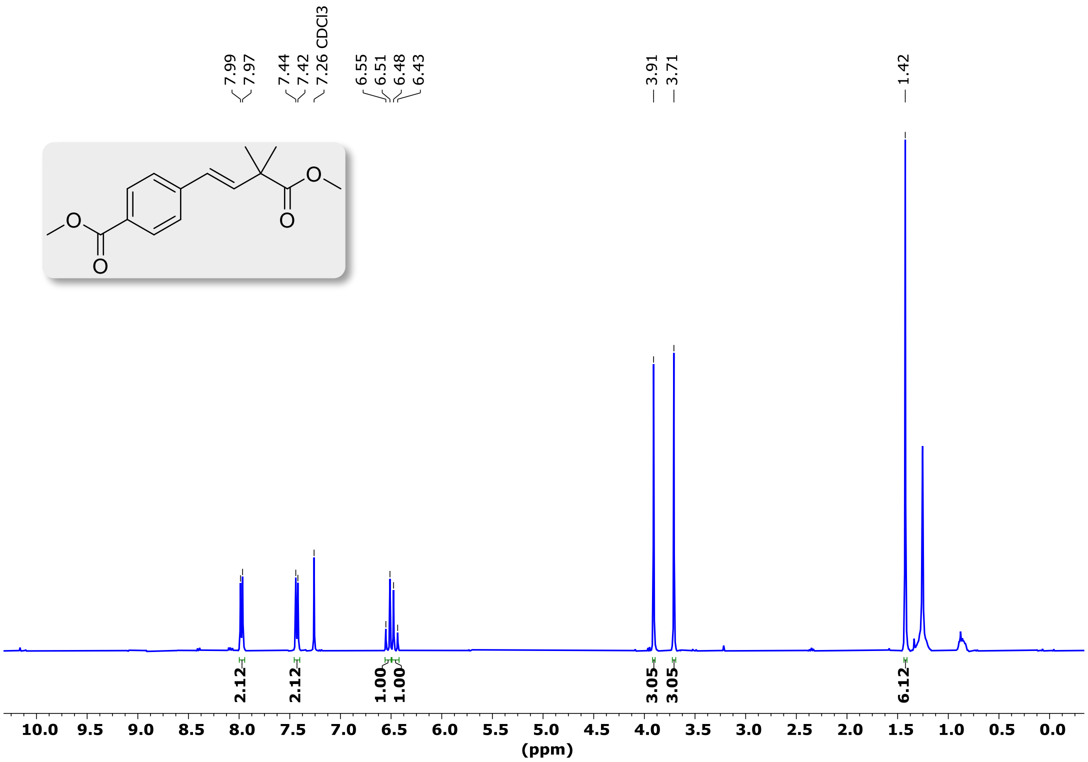


$^{1}H$ NMR, $CDCl_3$, 400 MHz (ppm)

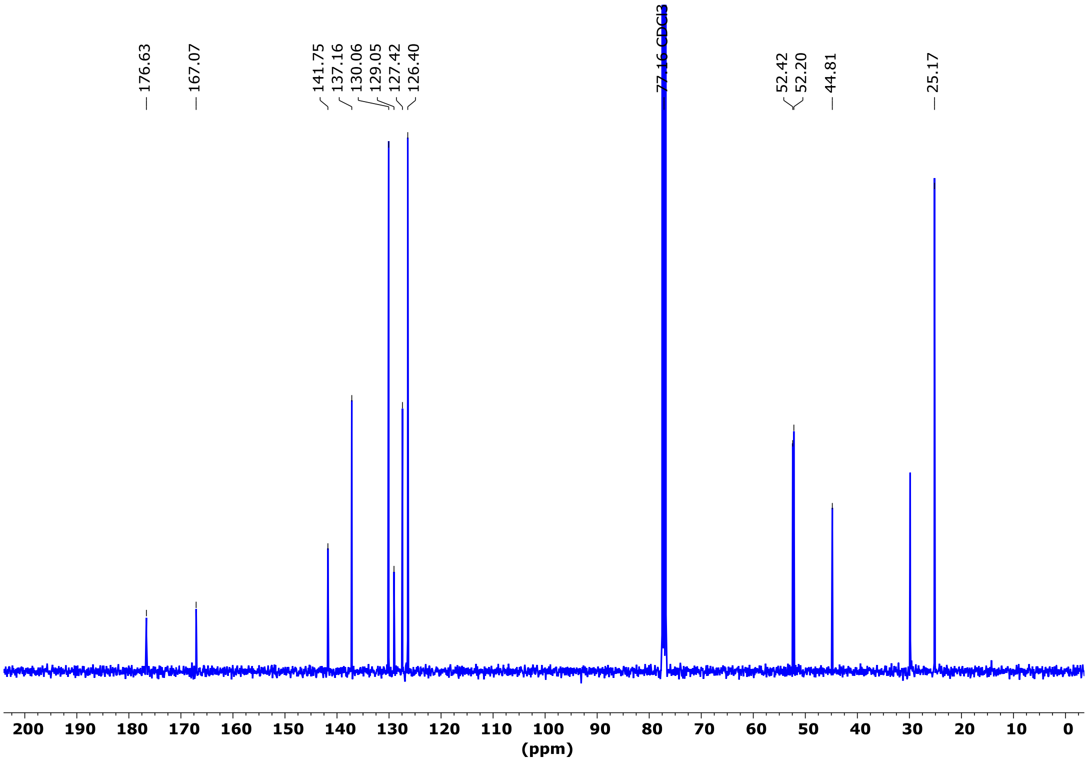


$^{13}C\{^{1}H\}$ NMR, $CDCl_3$, 100 MHz (ppm)

## Methyl 4-(4-methoxy-3,3-dimethyl-1-nitro-4-oxobutan-2-yl)benzoate (7n)

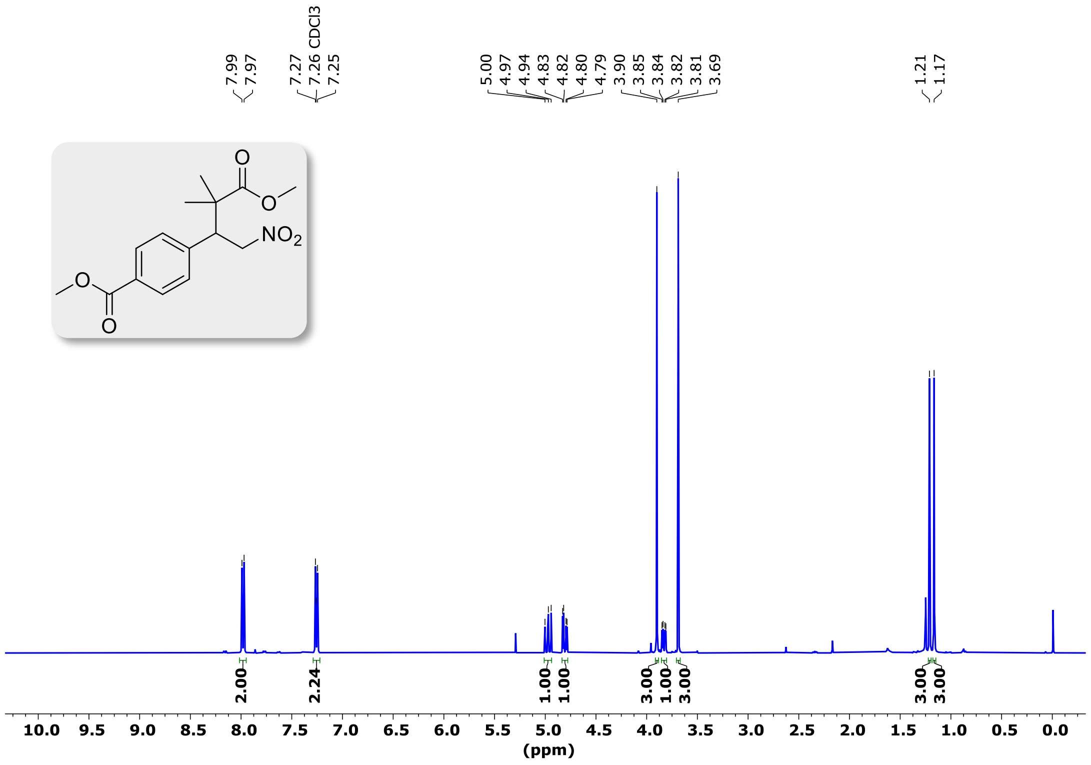


$^{1}H$ NMR, $CDCl_3$, 400 MHz (ppm)

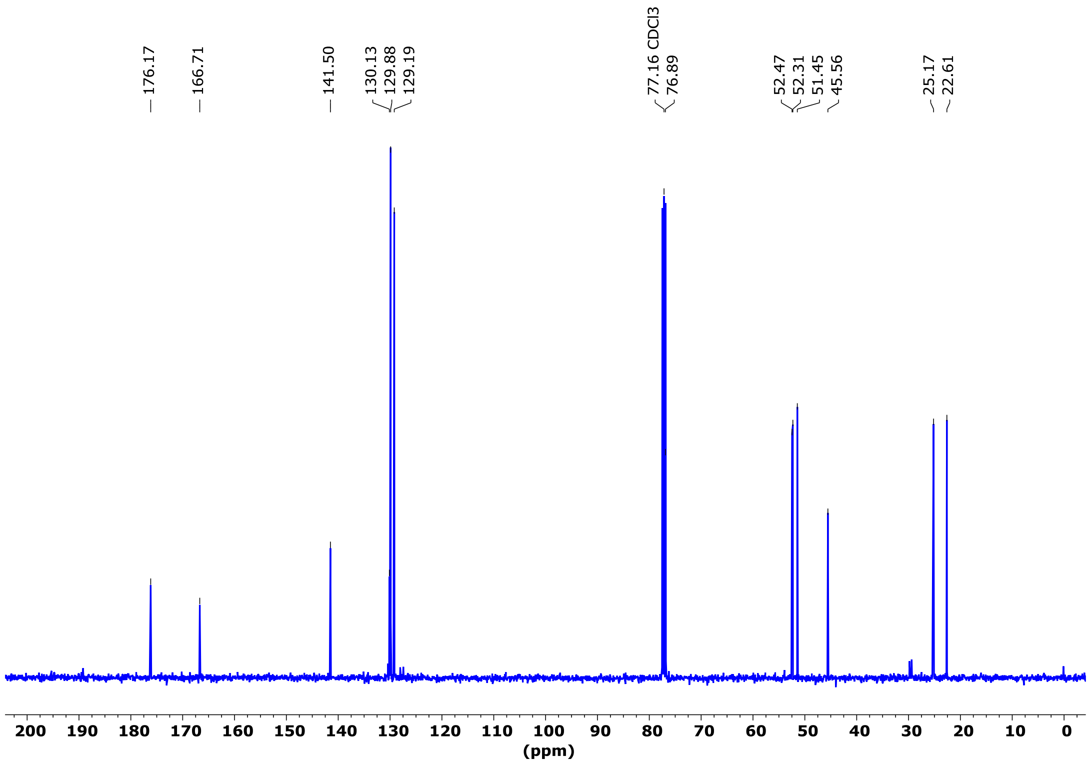


$^{13}C\{^{1}H\}$ NMR, $CDCl_3$, 100 MHz (ppm)

## Methyl 2,2-dimethyl-4-nitro-3-phenylbutanoate (7o)

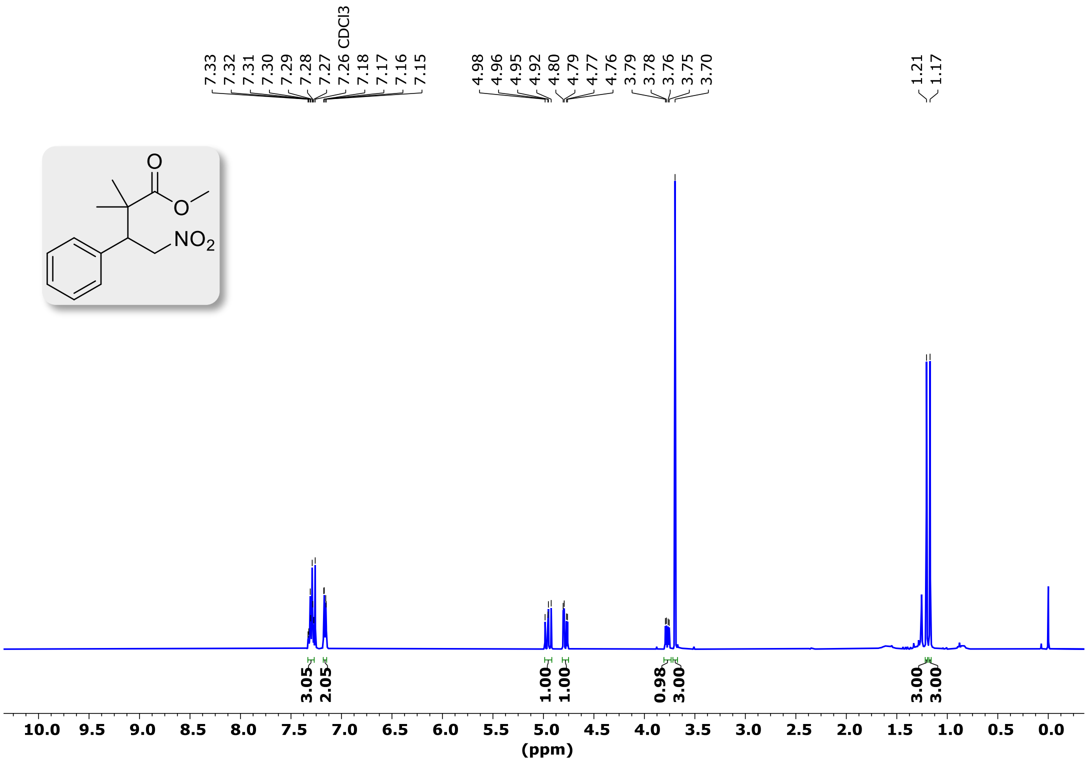


$^1$H NMR, $CDCl_3$, 400 MHz (ppm)

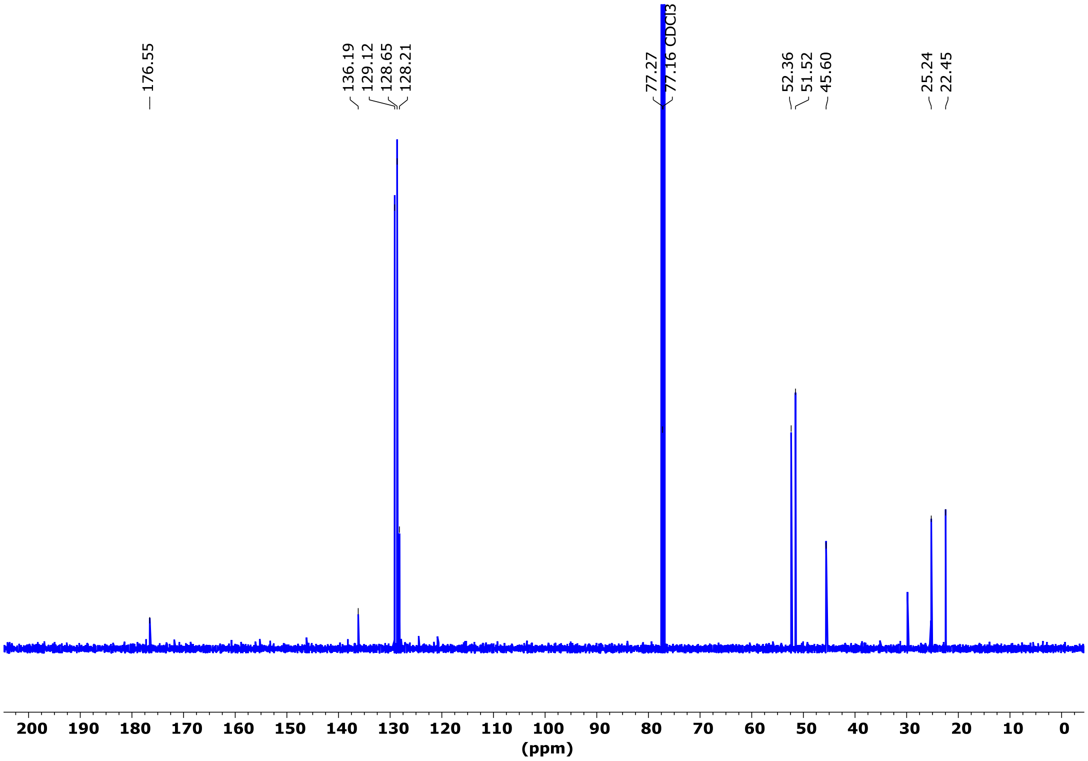


$^{13}$C{$^1$H} NMR, $CDCl_3$, 100 MHz (ppm)

## Methyl 3-(4-methoxyphenyl)-2,2-dimethyl-4-nitrobutanoate (7p)

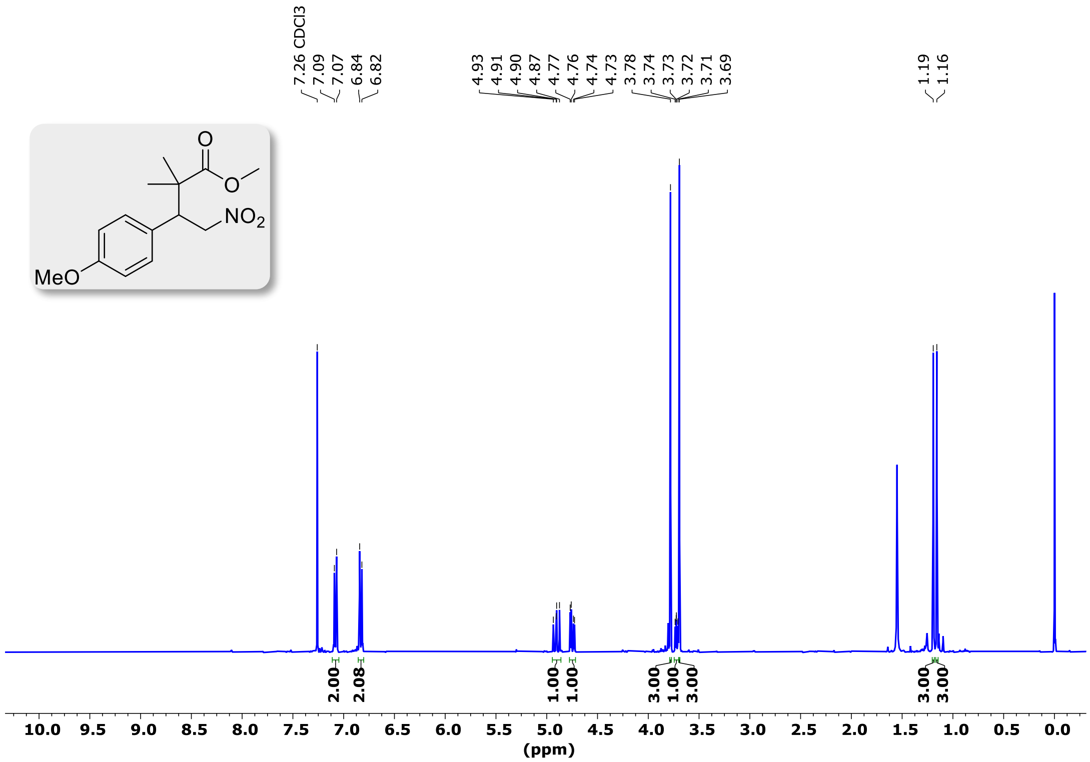


$^{1}H$ NMR, $CDCl_3$, 400 MHz (ppm)

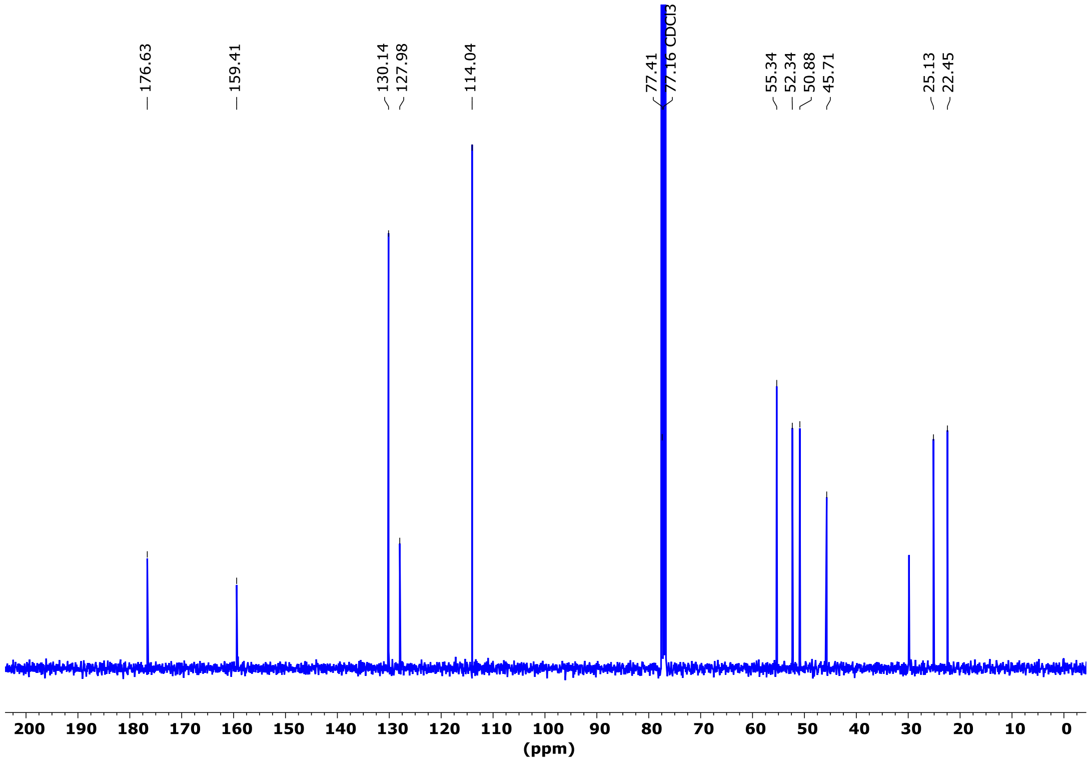


$^{13}C\{^{1}H\}$ NMR, $CDCl_3$, 100 MHz (ppm)

## Methyl (*E*)-2,2-dimethyl-3-(nitromethyl)-5-phenylpent-4-enoate (7q)

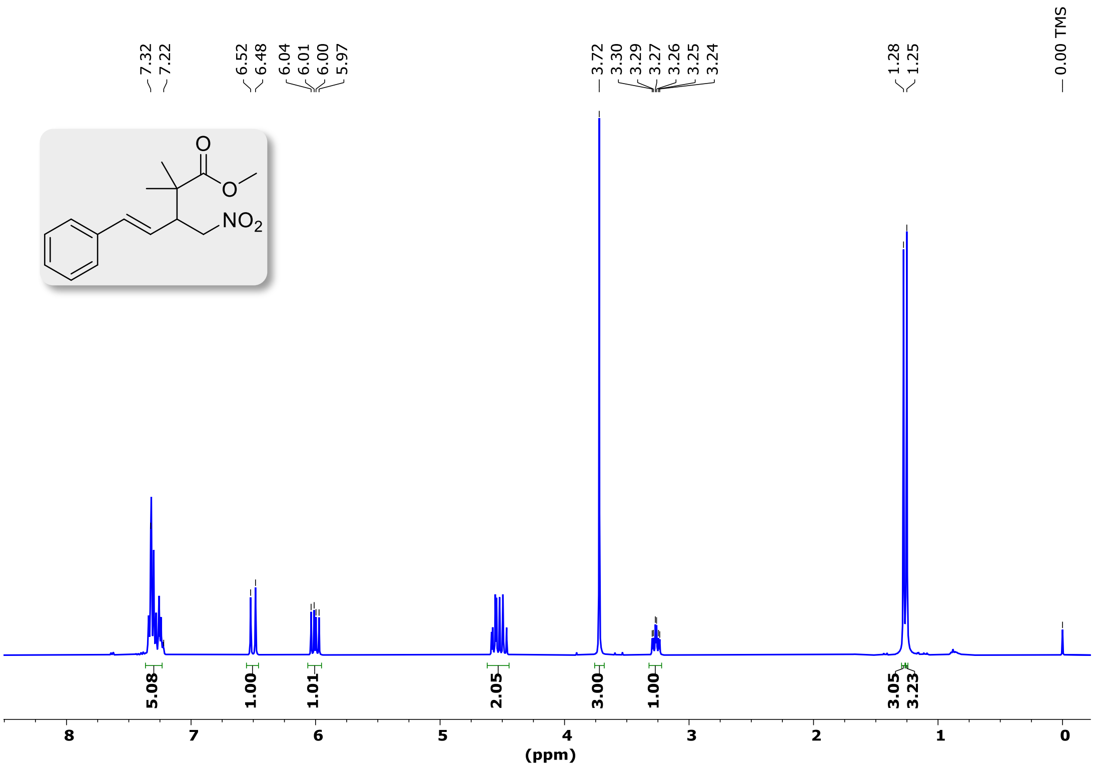


$^{1}H$ NMR, $CDCl_3$, 400 MHz (ppm)

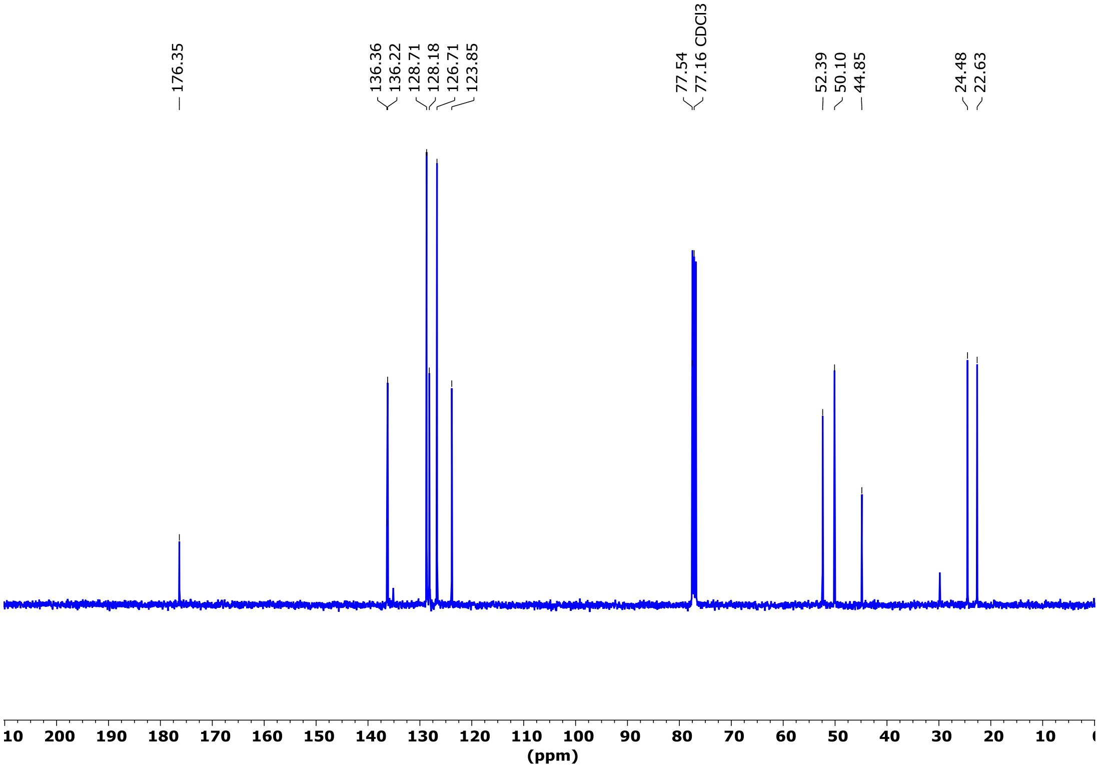


$^{13}C\{^{1}H\}$ NMR, $CDCl_3$, 100 MHz (ppm)

# Methyl (*E*)-2,2-dimethyl-4-(2,3,4,5,6-pentafluorophenyl)but-3-enoate (6r)

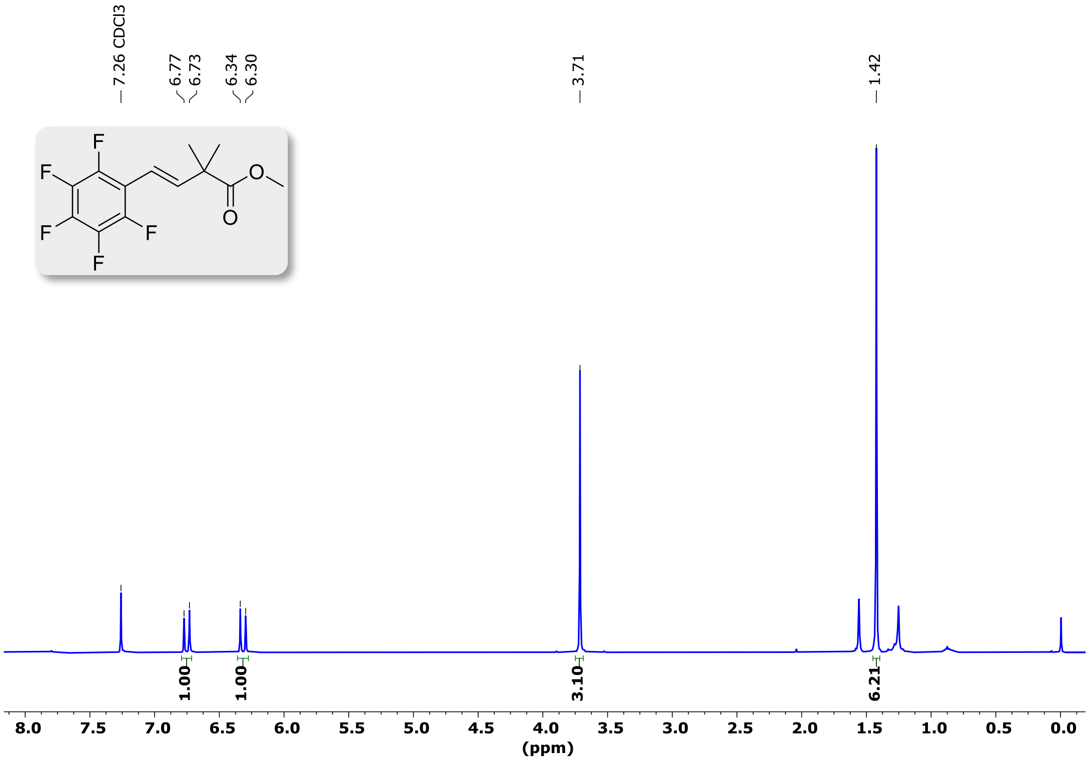


$^{1}$H NMR, $CDCl_3$, 400 MHz (ppm)

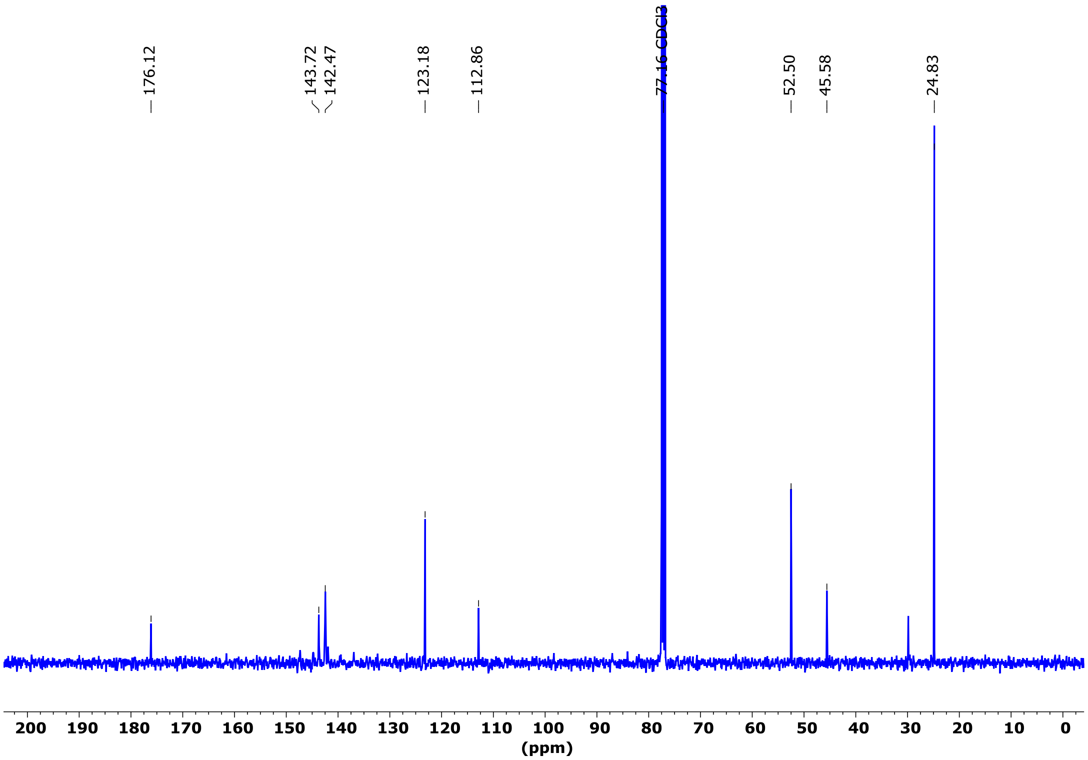


$^{13}$C{$^{1}$H} NMR, $CDCl_3$, 100 MHz (ppm)

## Methyl 2,2-dimethyl-4-nitro-3-(2,3,4,5,6-pentafluorophenyl)butanoate (7r)

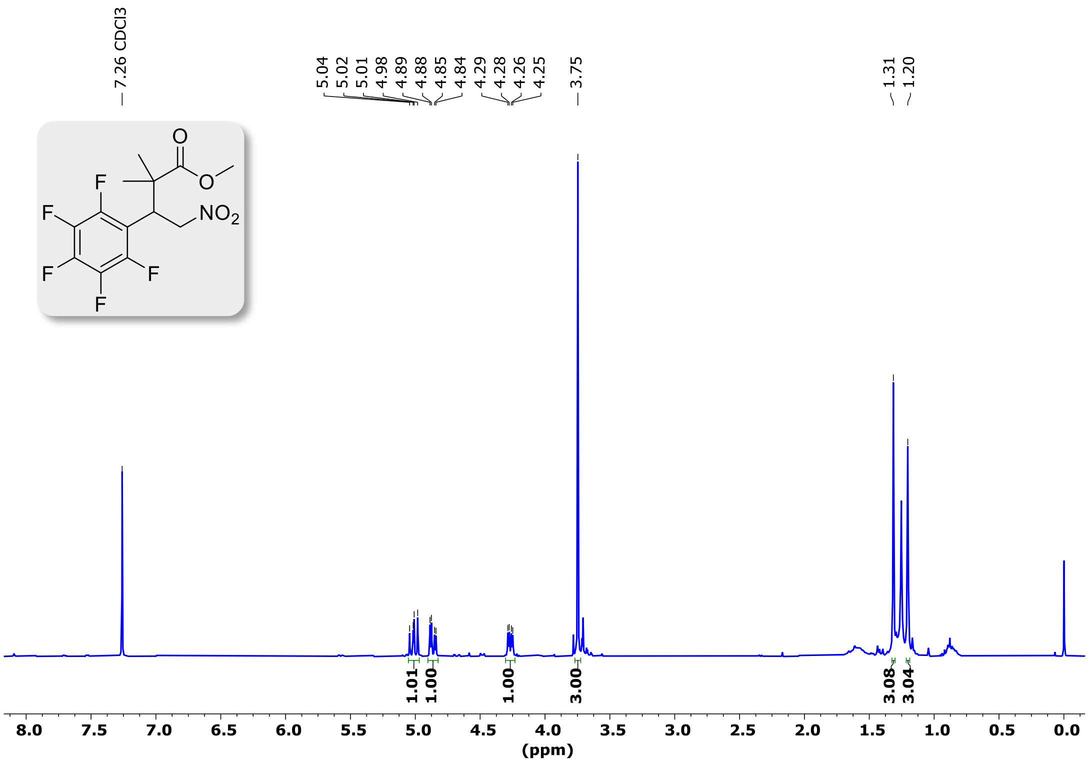


**$^{1}H$ NMR, $CDCl_3$, 400 MHz (ppm)**

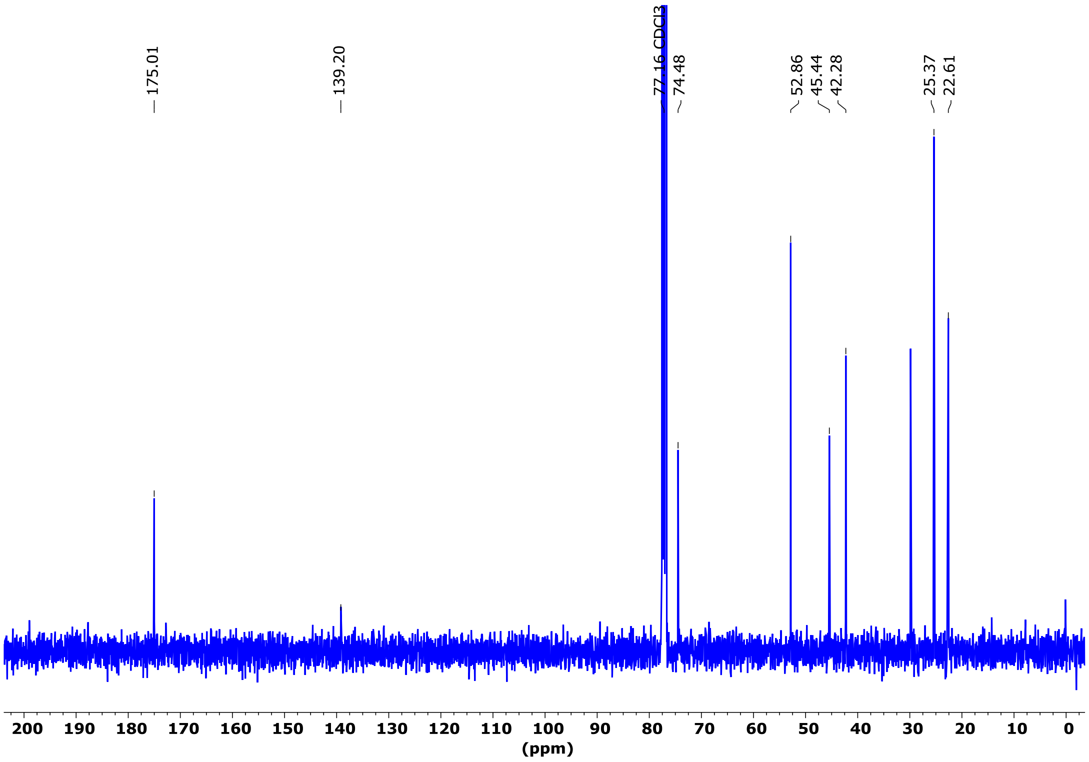


**$^{13}C\{^{1}H\}$ NMR, $CDCl_3$, 100 MHz (ppm)**

## Methyl (*E*)-2,2-dimethyl-4-(pyridin-4-yl)but-3-enoate (6s)
## (mixed with product 7s)

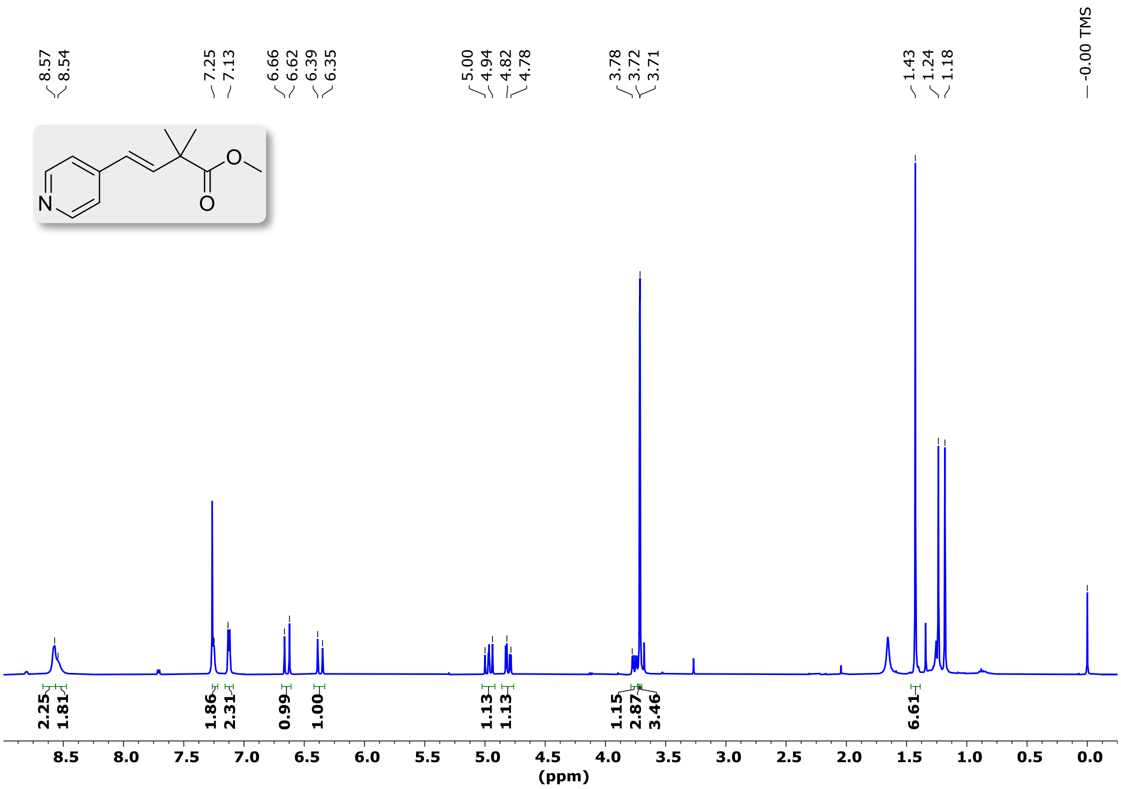


$^{1}H$ NMR, $CDCl_3$, 400 MHz (ppm)

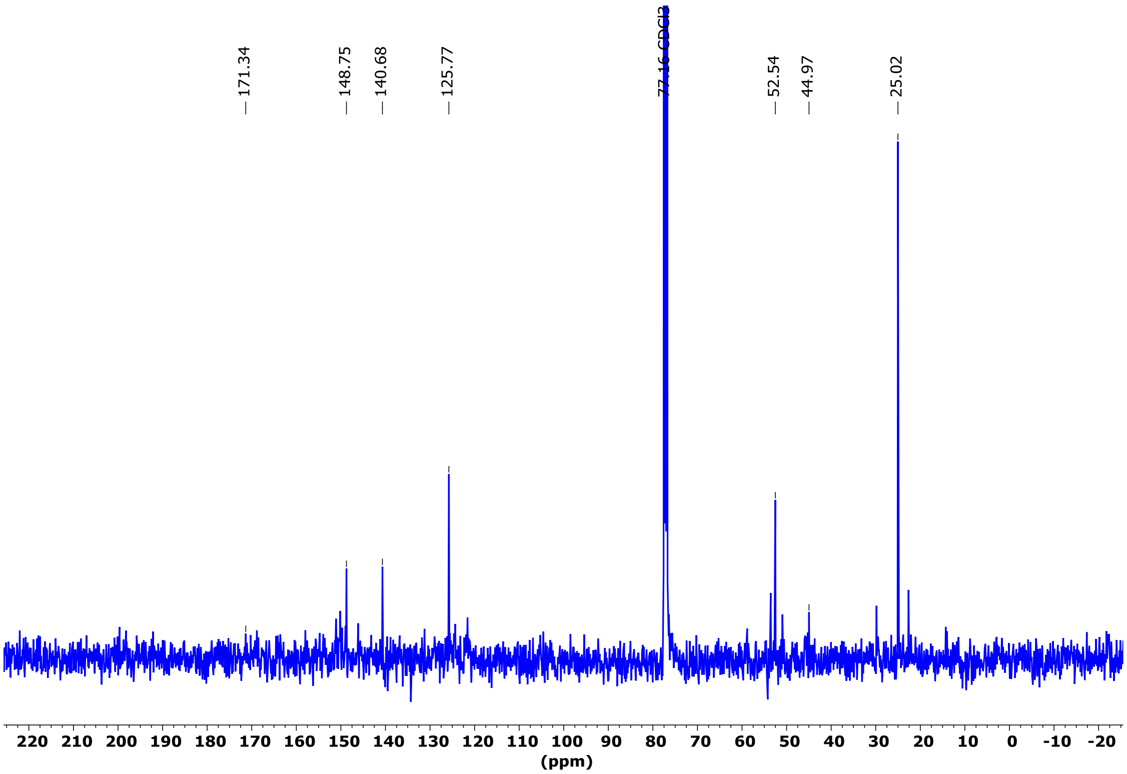


$^{13}C\{^{1}H\}$ NMR, $CDCl_3$, 100 MHz (ppm)

## Methyl 2,2-dimethyl-4-nitro-3-(pyridin-4-yl)butanoate (7s)

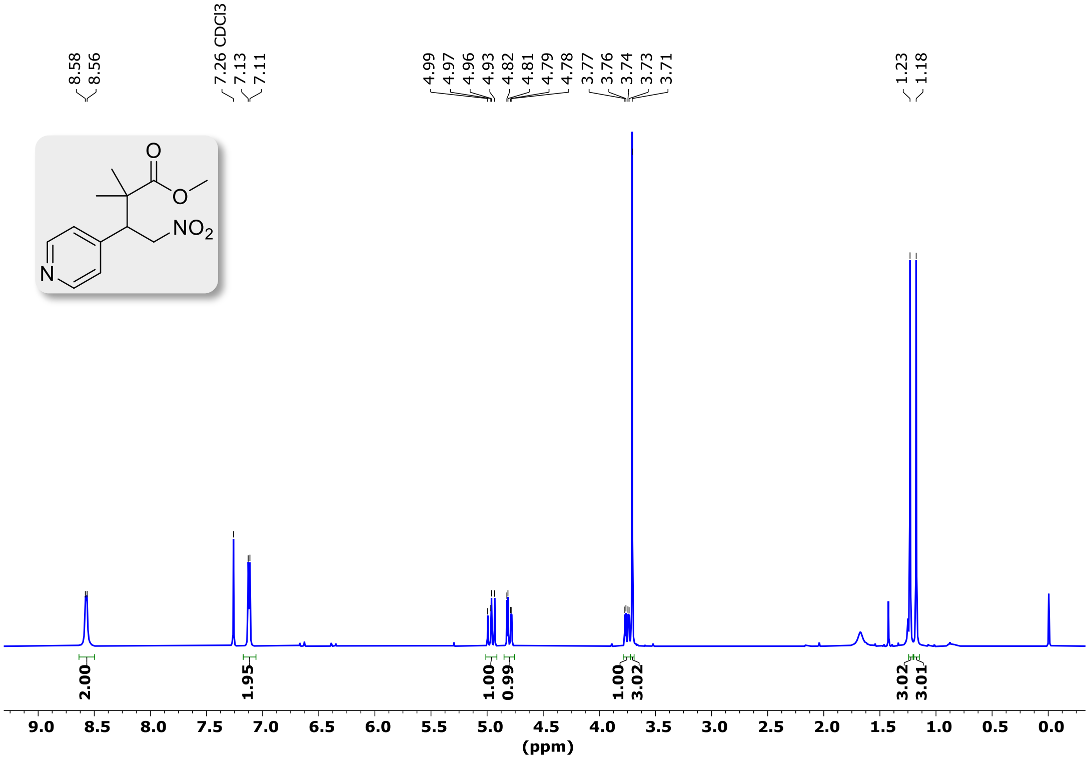


$^{1}H$ NMR, $CDCl_3$, 400 MHz (ppm)

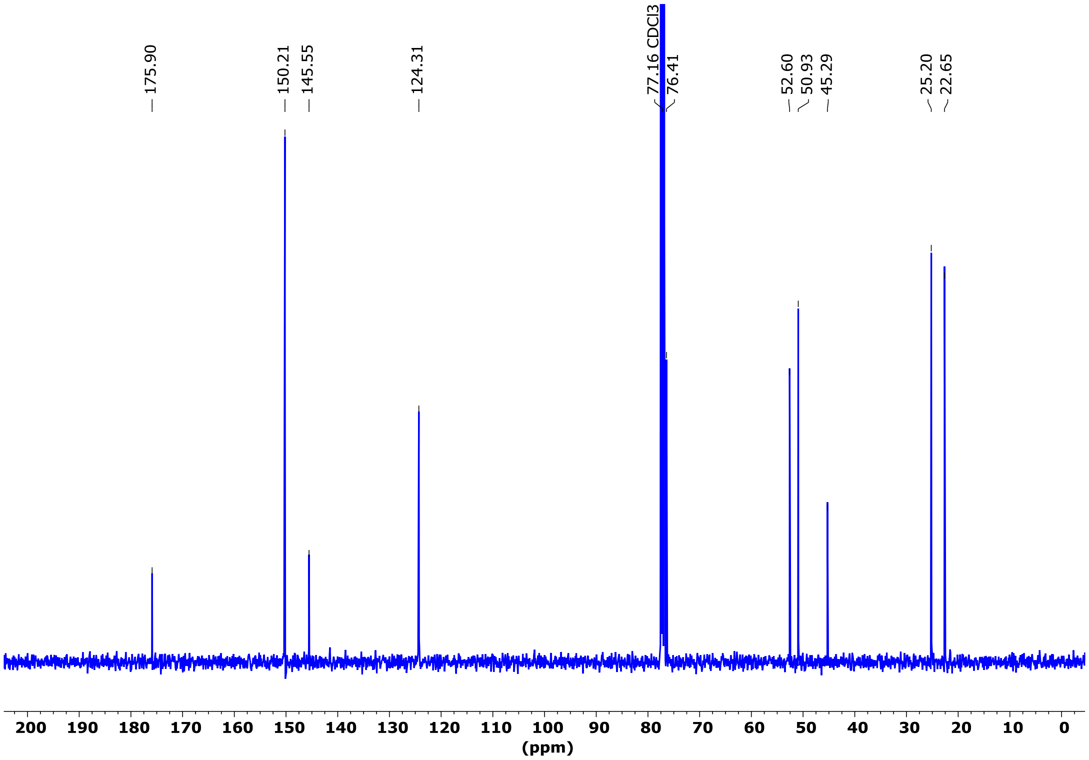


$^{13}C\{^{1}H\}$ NMR, $CDCl_3$, 100 MHz (ppm)

## Methyl 3-(3,4-dimethoxyphenyl)-2,2-dimethyl-4-nitrobutanoate (7t)

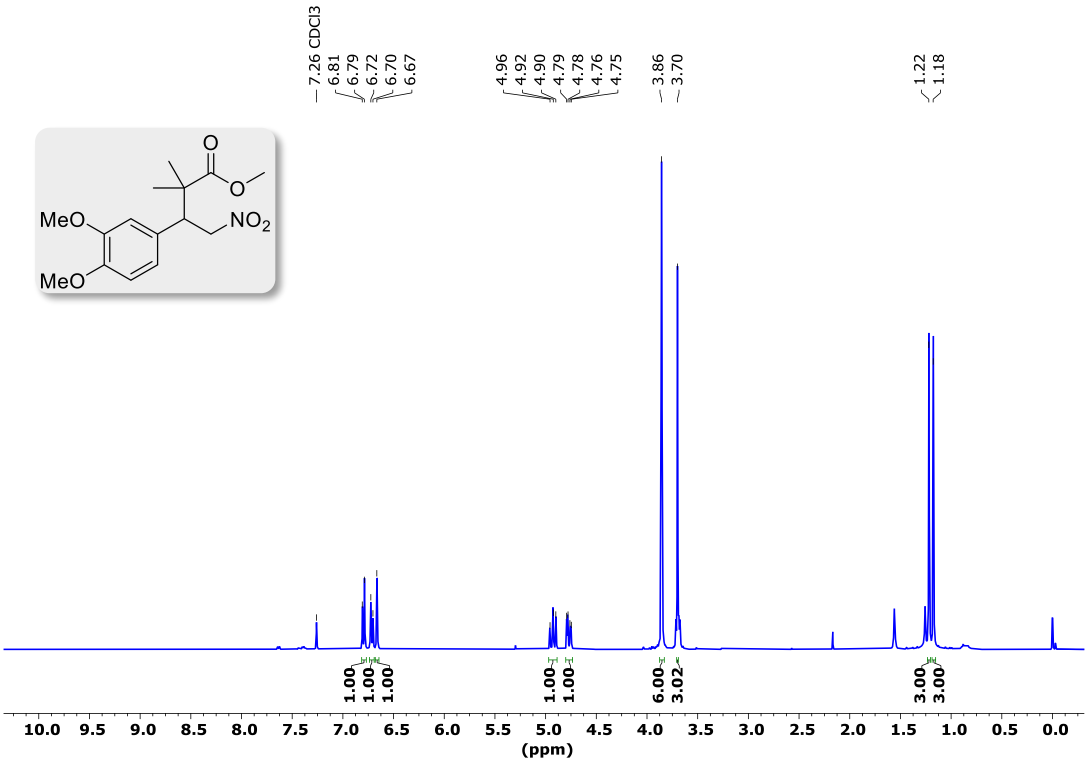


$^{1}H$ NMR, $CDCl_3$, 400 MHz (ppm)

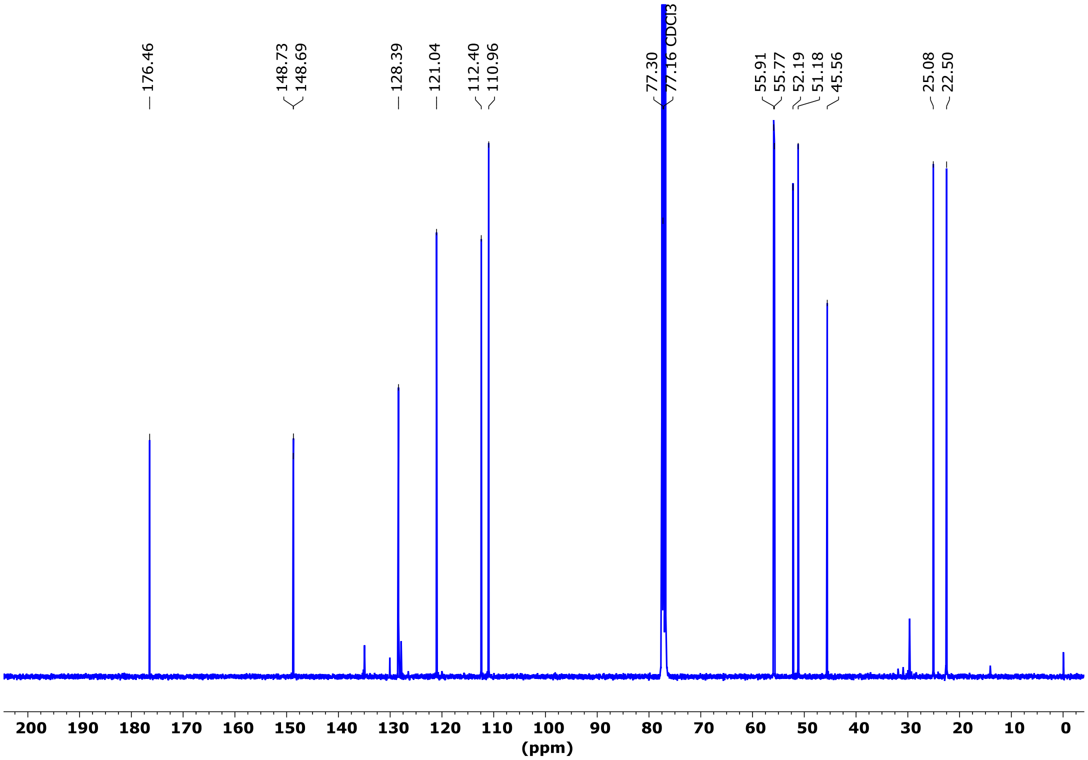


$^{13}C\{^{1}H\}$ NMR, $CDCl_3$, 100 MHz (ppm)

## Methyl (*E*)-4-(2-ethynylphenyl)-2,2-dimethylbut-3-enoate (6u)

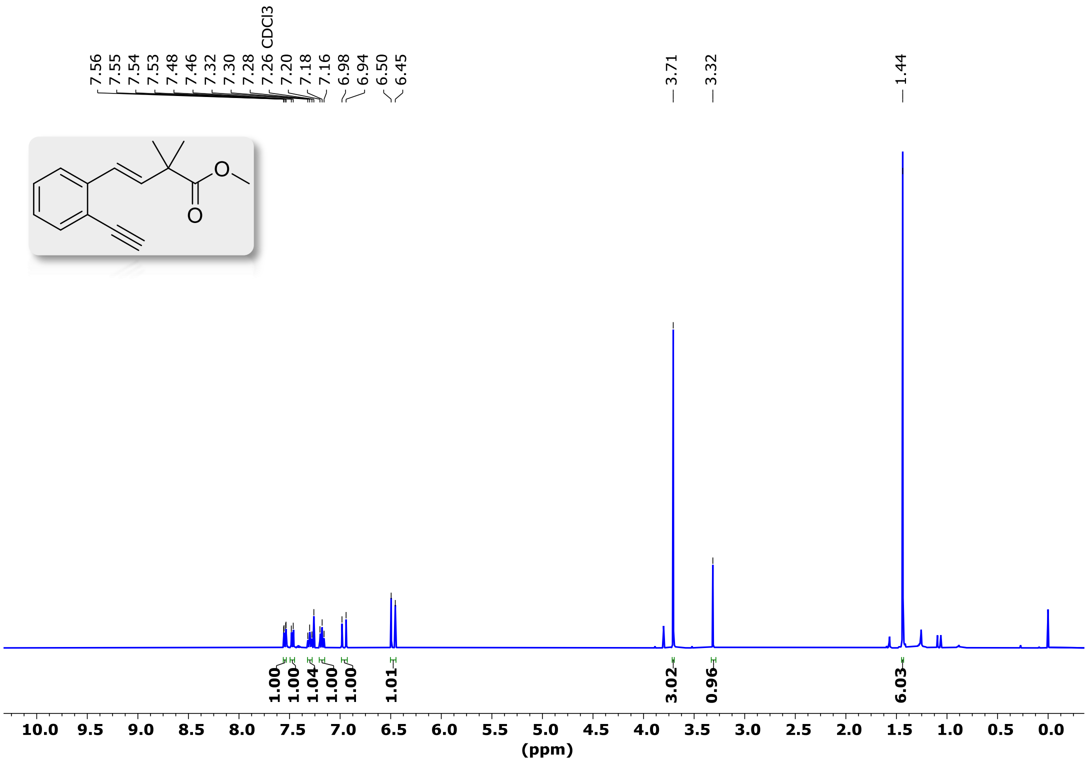


$^{1}$H NMR, $CDCl_3$, 400 MHz (ppm)

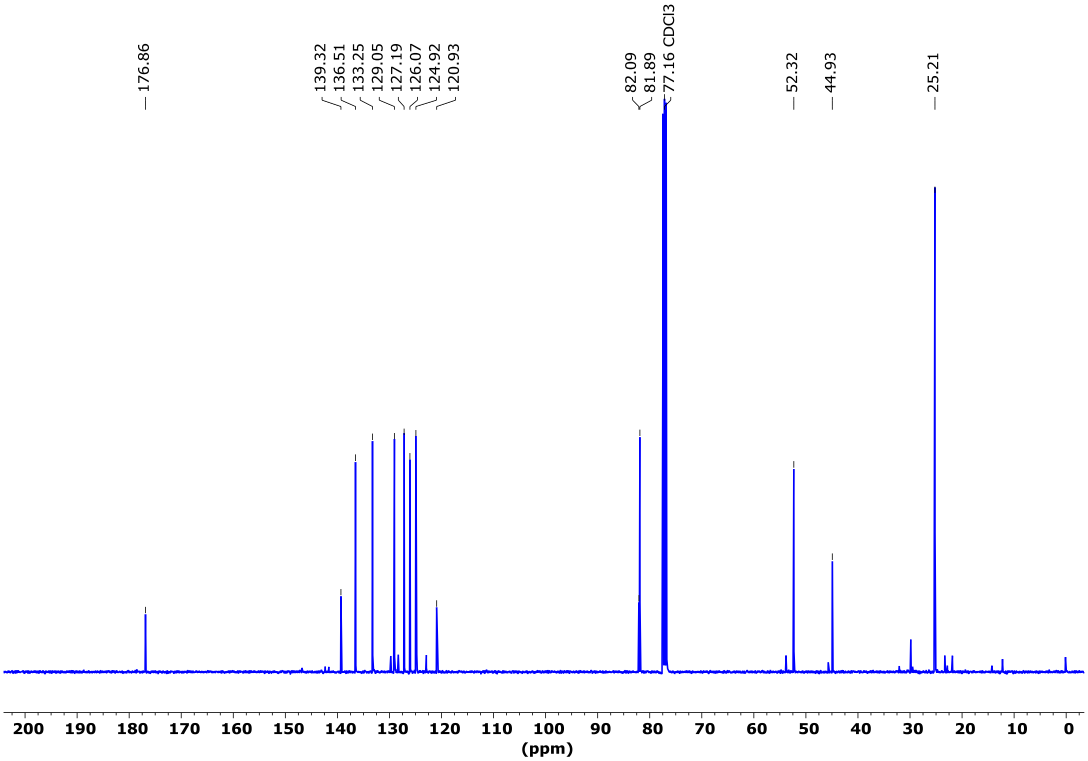


$^{13}C\{^{1}H\}$ NMR, $CDCl_3$, 100 MHz (ppm)

## Methyl 3-(2-ethynylphenyl)-2,2-dimethyl-4-nitrobutanoate (7u)

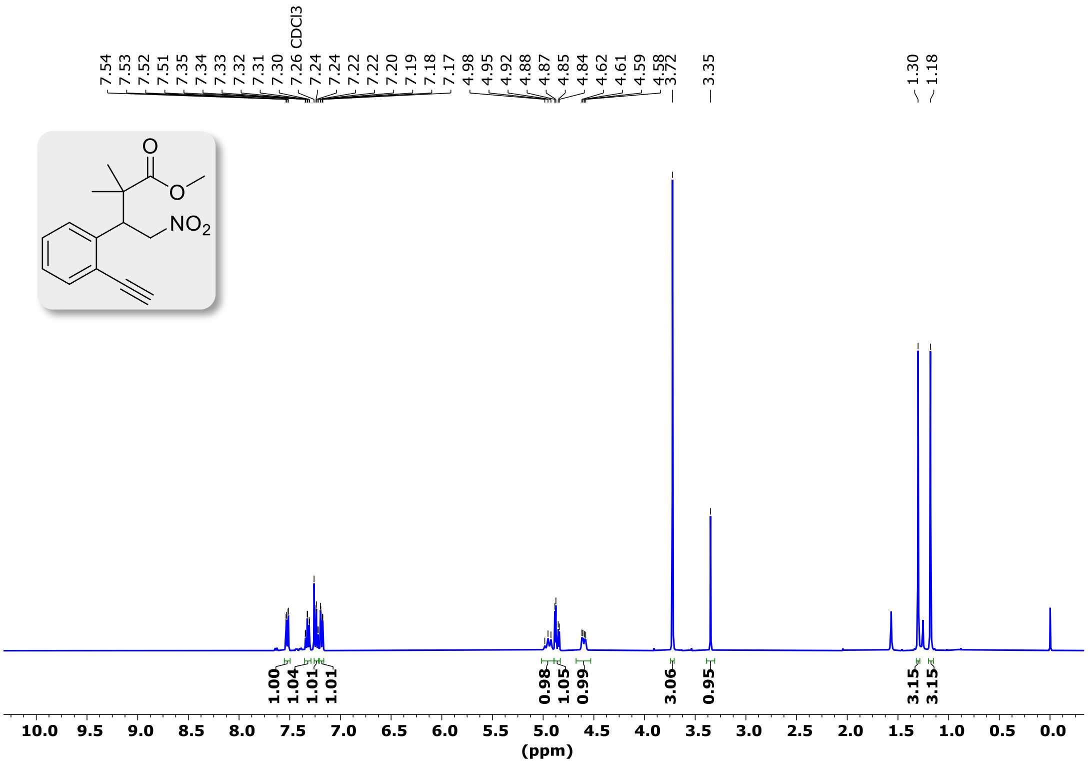


$^{1}H$ NMR, $CDCl_3$, 400 MHz (ppm)

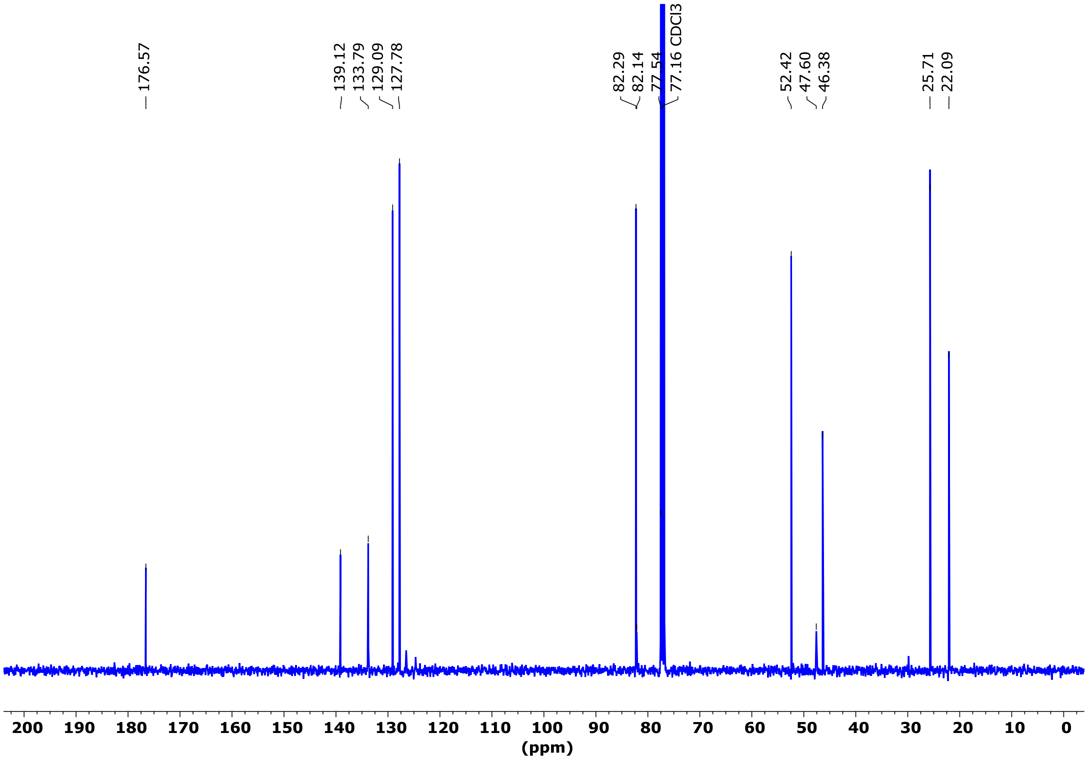


$^{13}C\{^{1}H\}$ NMR, $CDCl_3$, 100 MHz (ppm)

## Methyl (*E*)-4-(2,4-dinitrophenyl)-2,2-dimethylbut-3-enoate (6v)

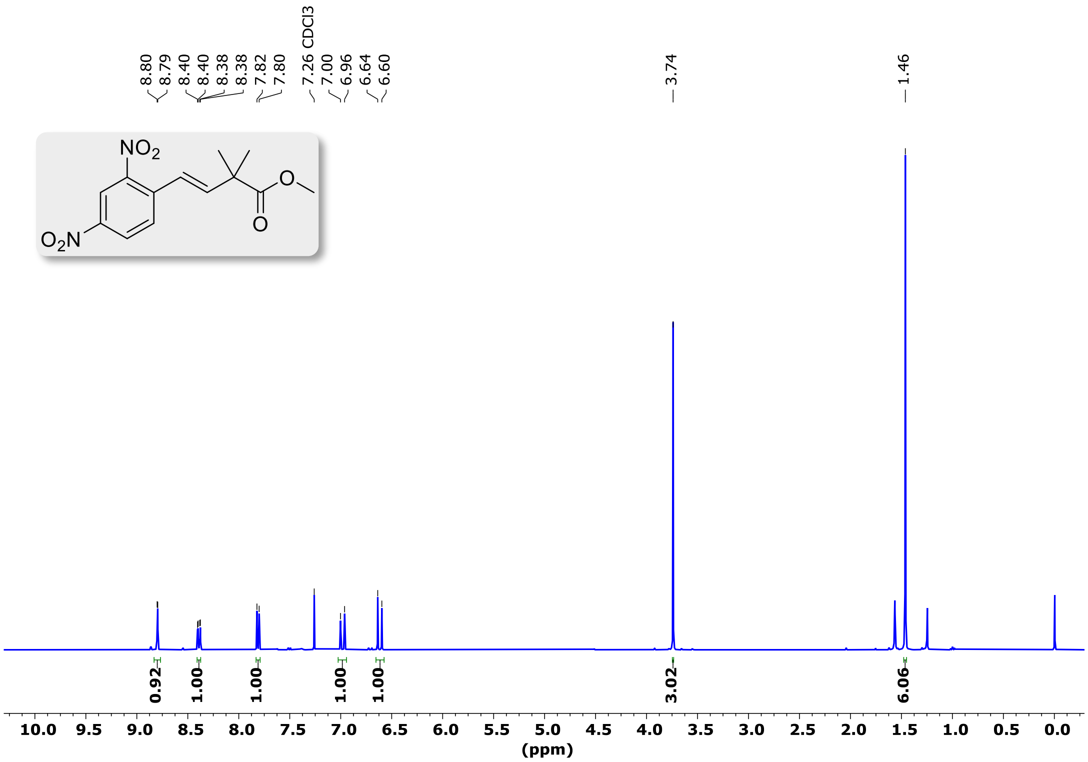


$^{1}H$ NMR, $CDCl_3$, 400 MHz (ppm)

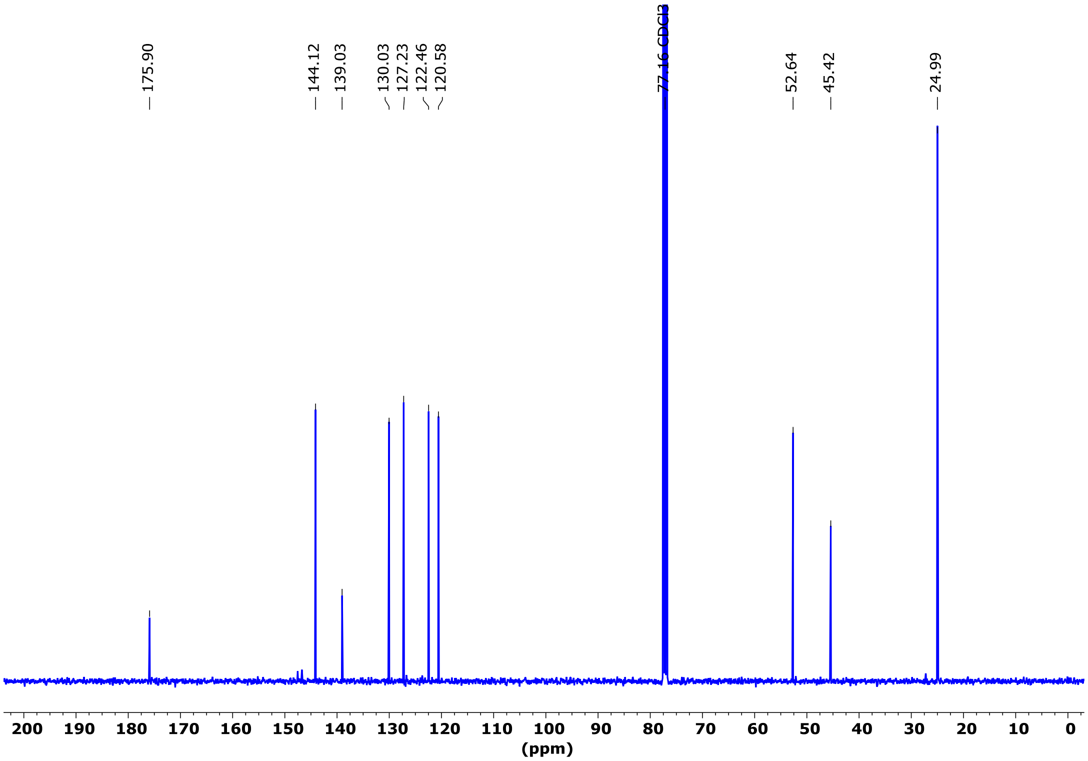


$^{13}C\{^{1}H\}$ NMR, $CDCl_3$, 100 MHz (ppm)

## Methyl 3-(2,4-dinitrophenyl)-2,2-dimethyl-4-nitrobutanoate (7v)

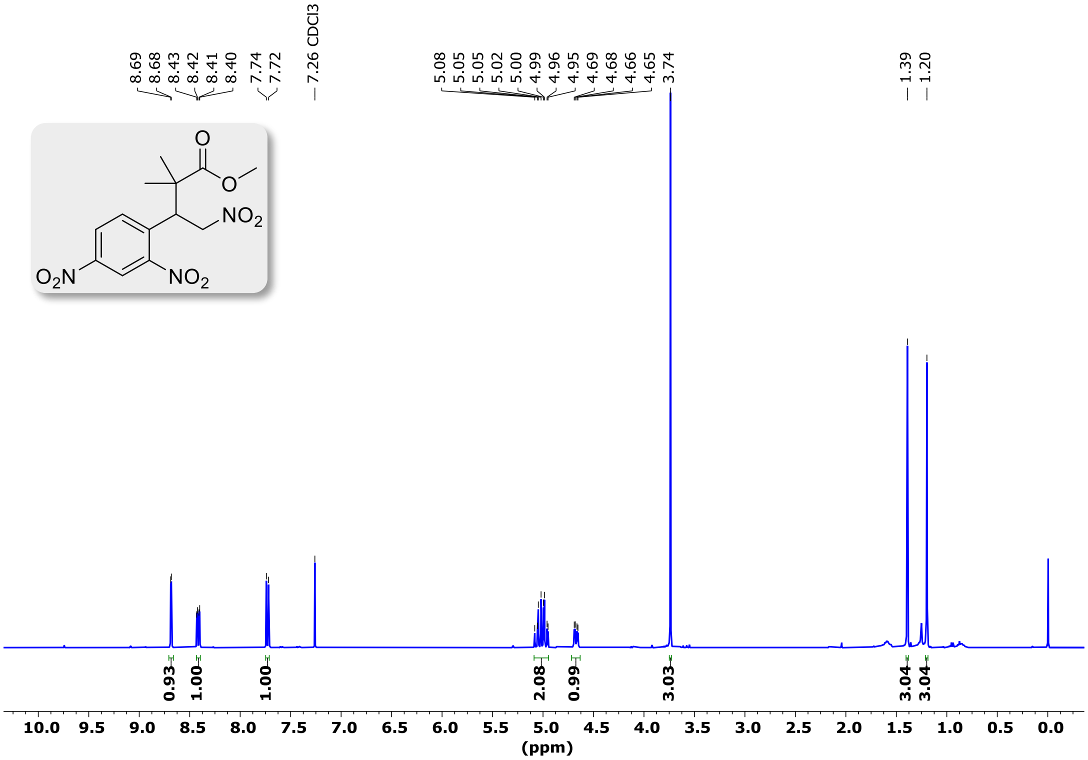


$^{1}$H NMR, $CDCl_3$, 400 MHz (ppm)

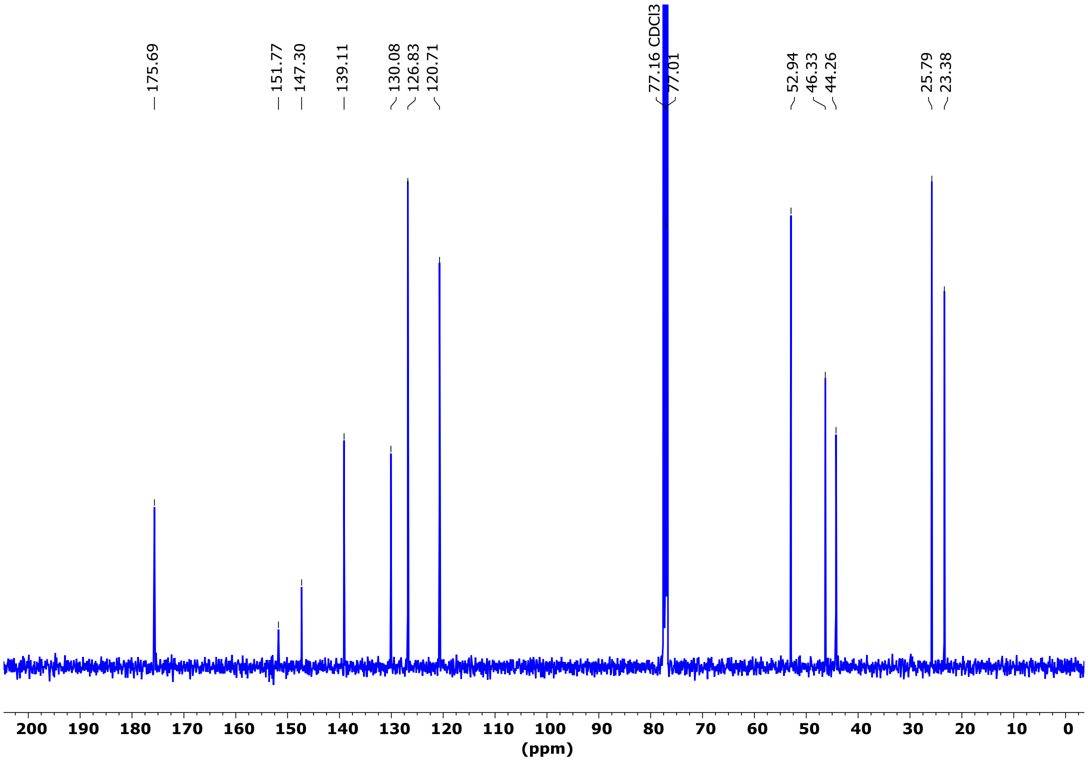


$^{13}$C{$^{1}$H} NMR, $CDCl_3$, 100 MHz (ppm)

## Methyl 2,2-dimethyl-3-(nitromethyl)-5-phenylpentanoate (7w)

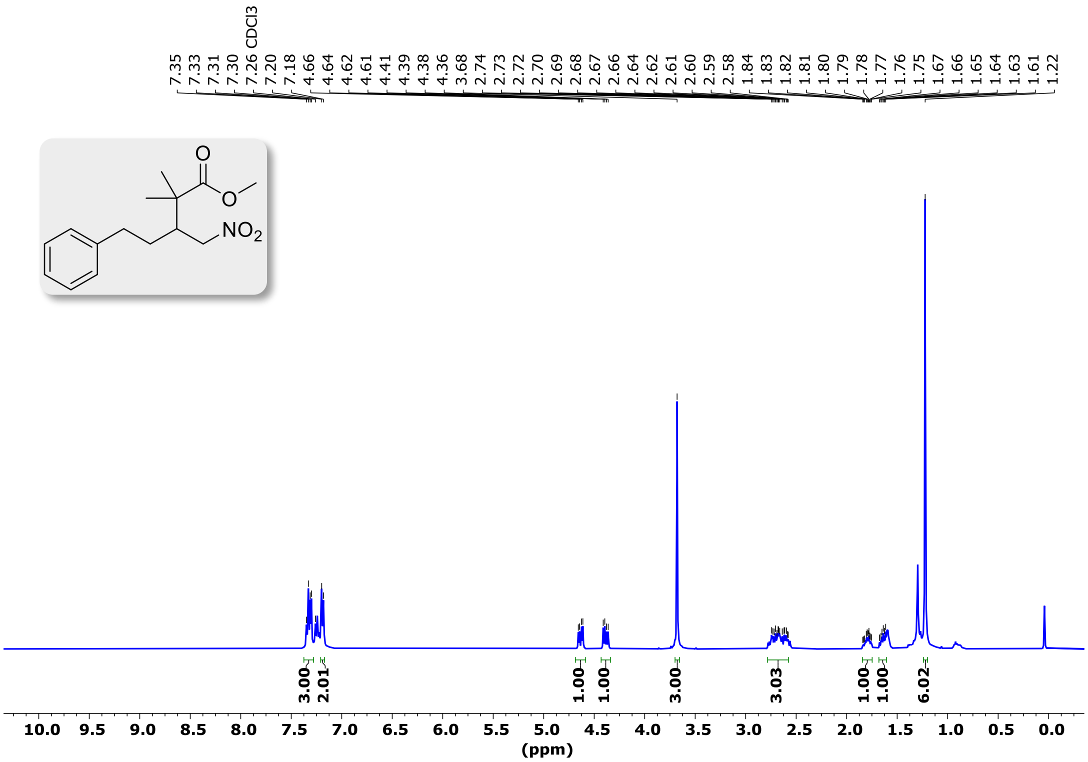


$^{1}H$ NMR, $CDCl_3$, 400 MHz (ppm)

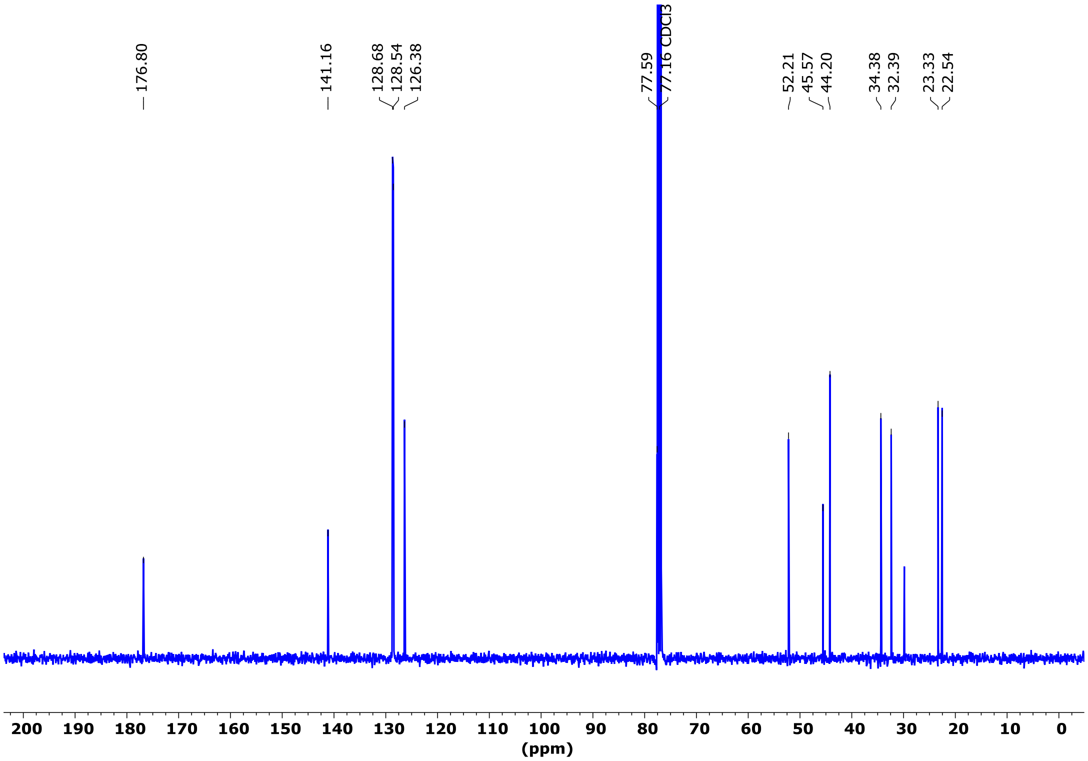


$^{13}C\{^{1}H\}$ NMR, $CDCl_3$, 100 MHz (ppm)

## Methyl 2,2-dimethyl-4-nitro-3-phenylpentanoate (7x)

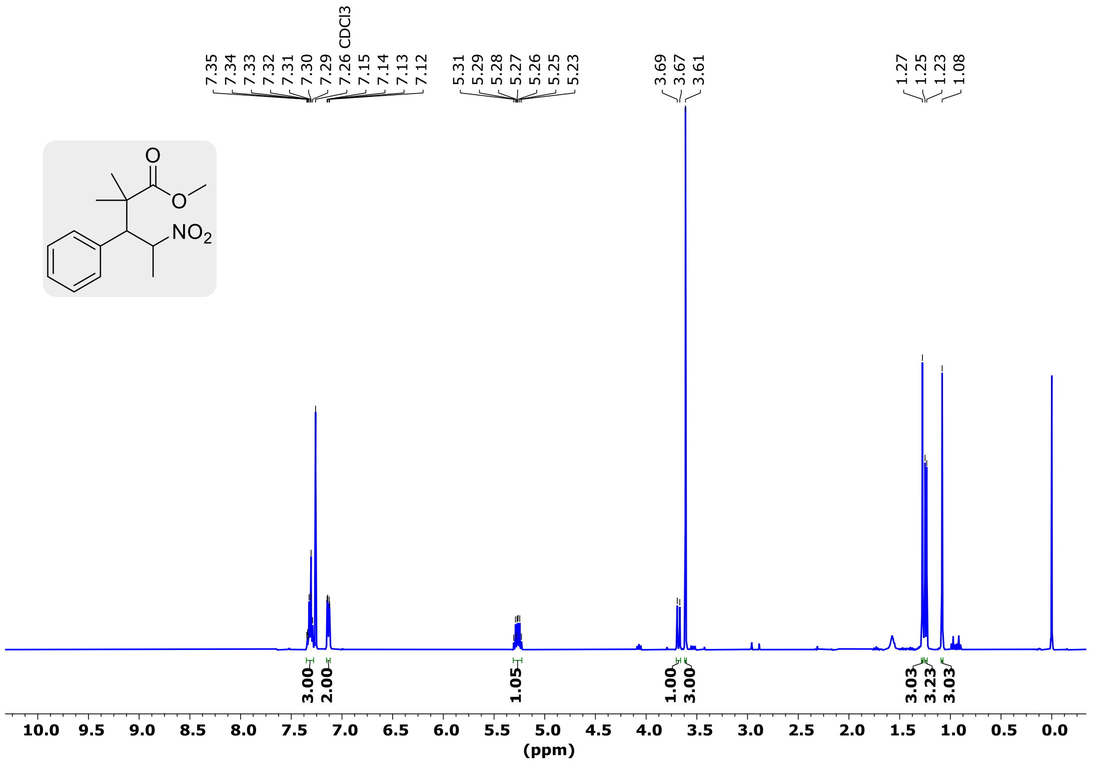


$^1$H NMR, $CDCl_3$, 400 MHz (ppm)

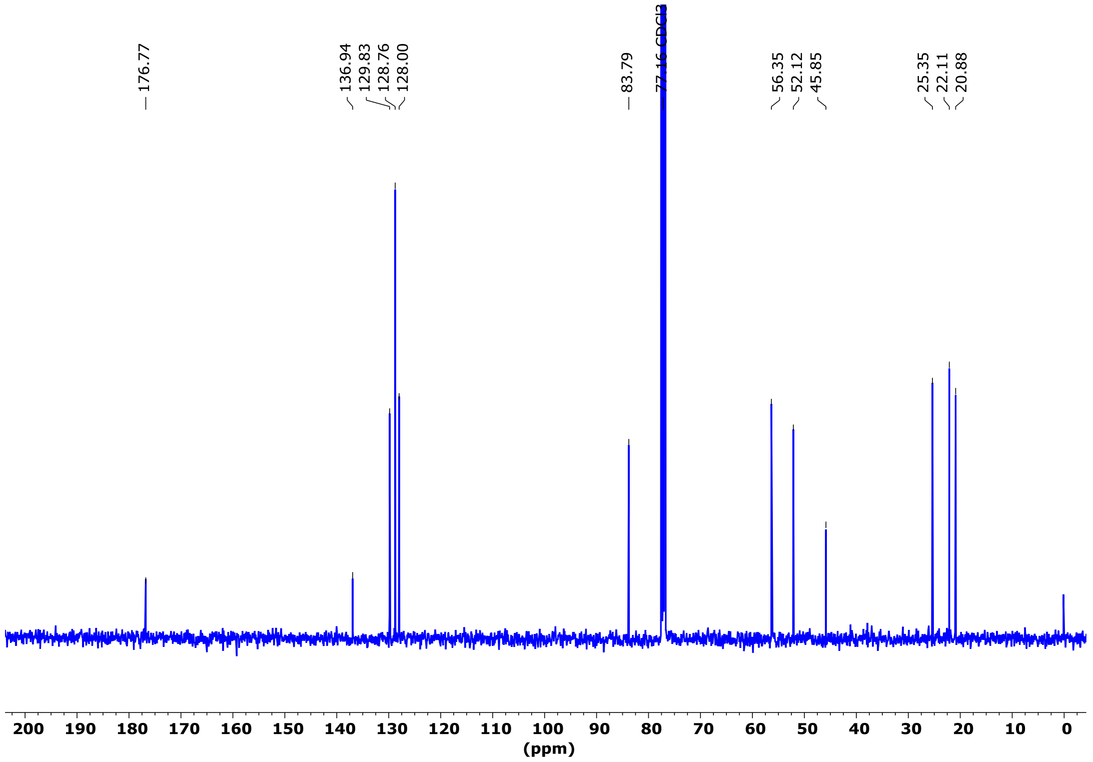


$^{13}$C{$^1$H} NMR, $CDCl_3$, 100 MHz (ppm)

## Methyl (*E*)-2,2,3-trimethyl-4-(2-nitrophenyl)but-3-enoate (6y)

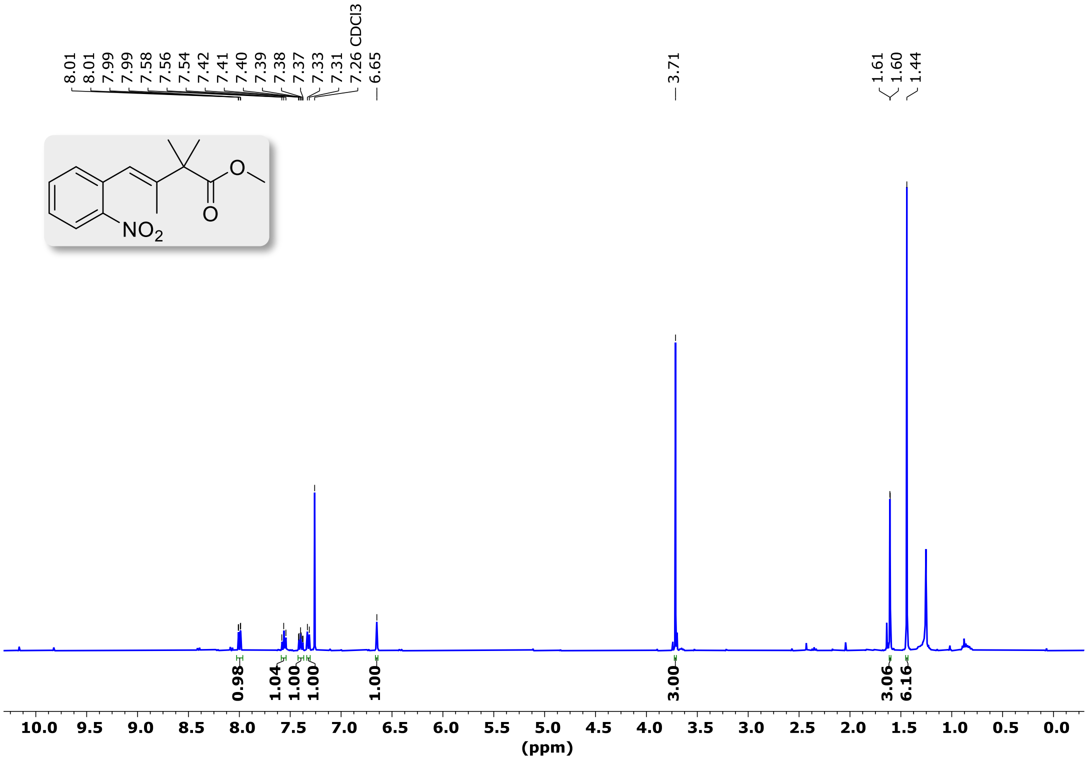


$^{1}$H NMR, $CDCl_3$, 400 MHz (ppm)

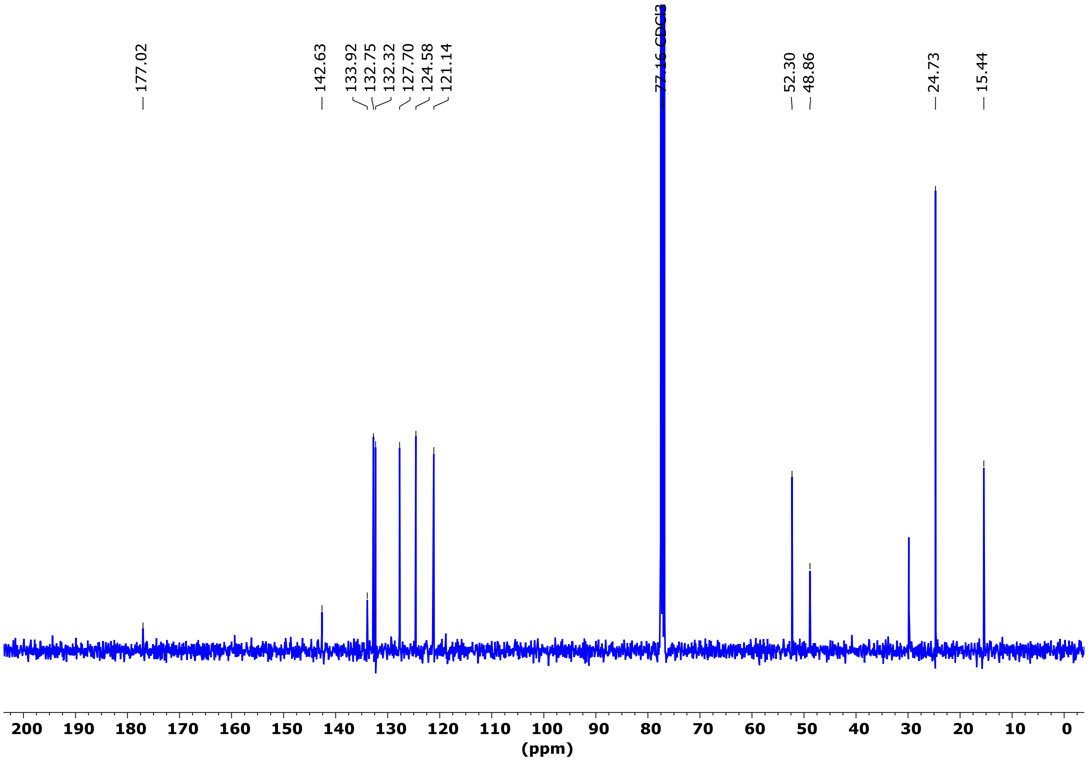


$^{13}$C{$^{1}$H} NMR, $CDCl_3$, 100 MHz (ppm)

**Methyl 2,2-dimethyl-4-nitro-3-(2-nitrophenyl)pentanoate (7y)**

**(diastereomer mixture, *d.r.* = 1:1)**

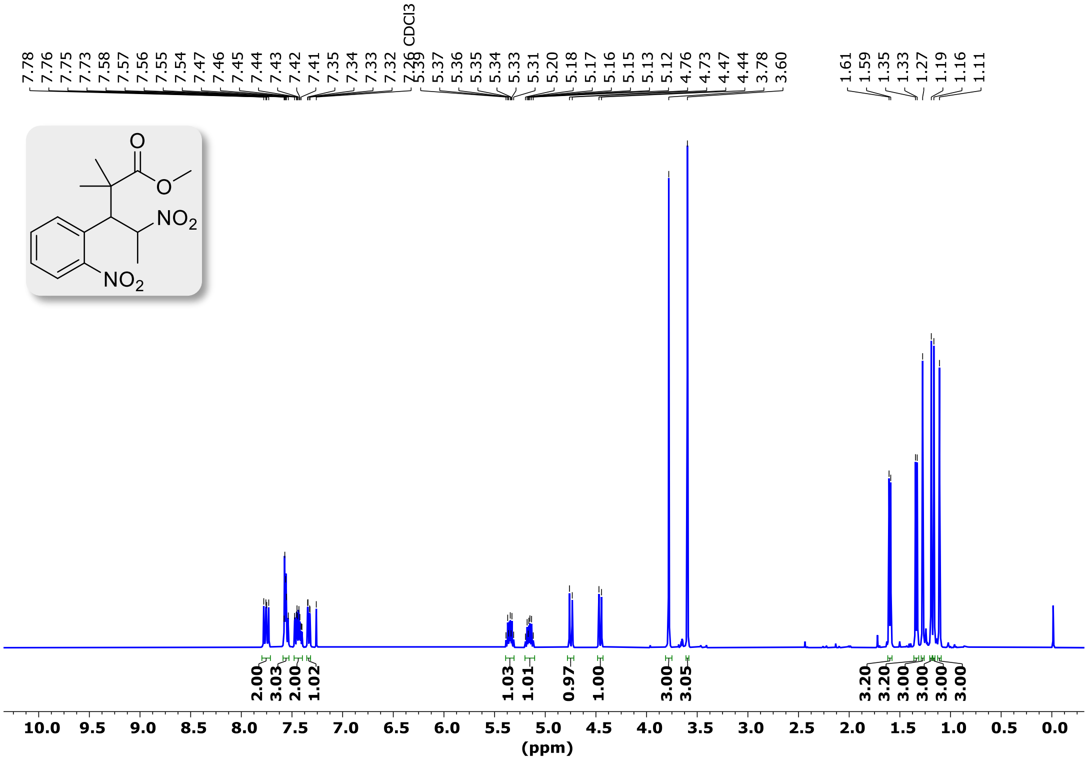


**$^1$H NMR, $CDCl_3$, 400 MHz (ppm)**

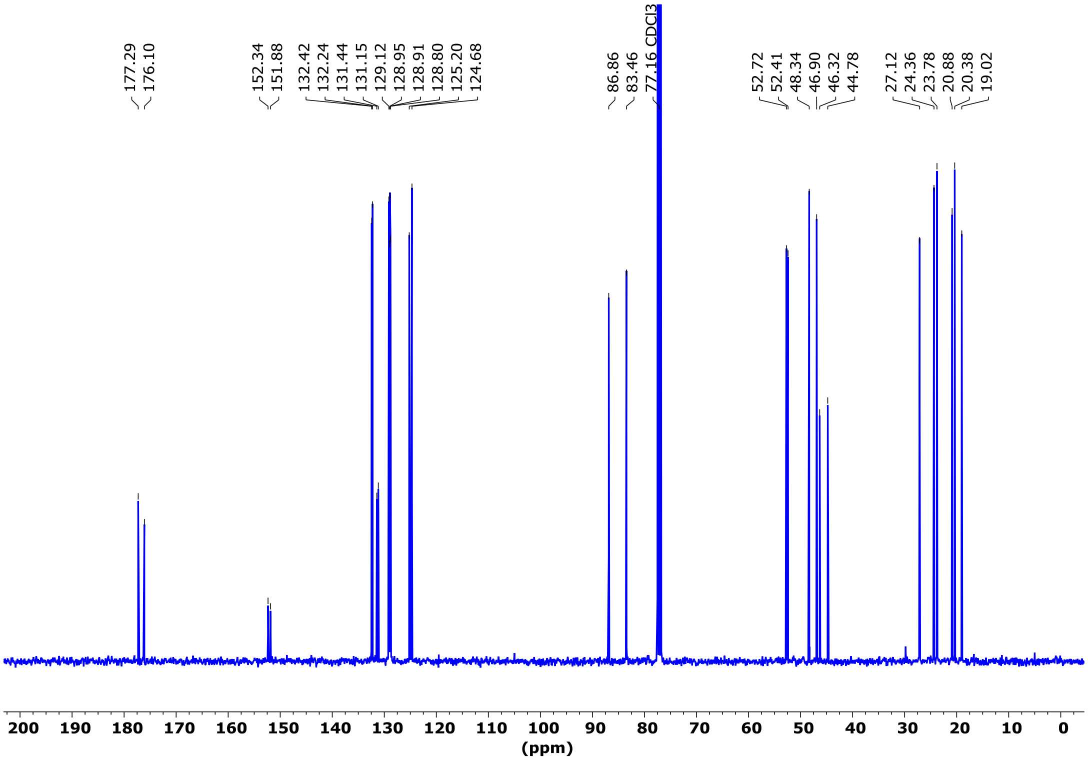


**$^{13}$C{$^1$H} NMR, $CDCl_3$, 100 MHz (ppm)**

# Methyl 2,2,3-trimethyl-4-(4-nitrophenyl)but-3-enoate (6z)
## (*E*/*Z* mixture, 1:1)

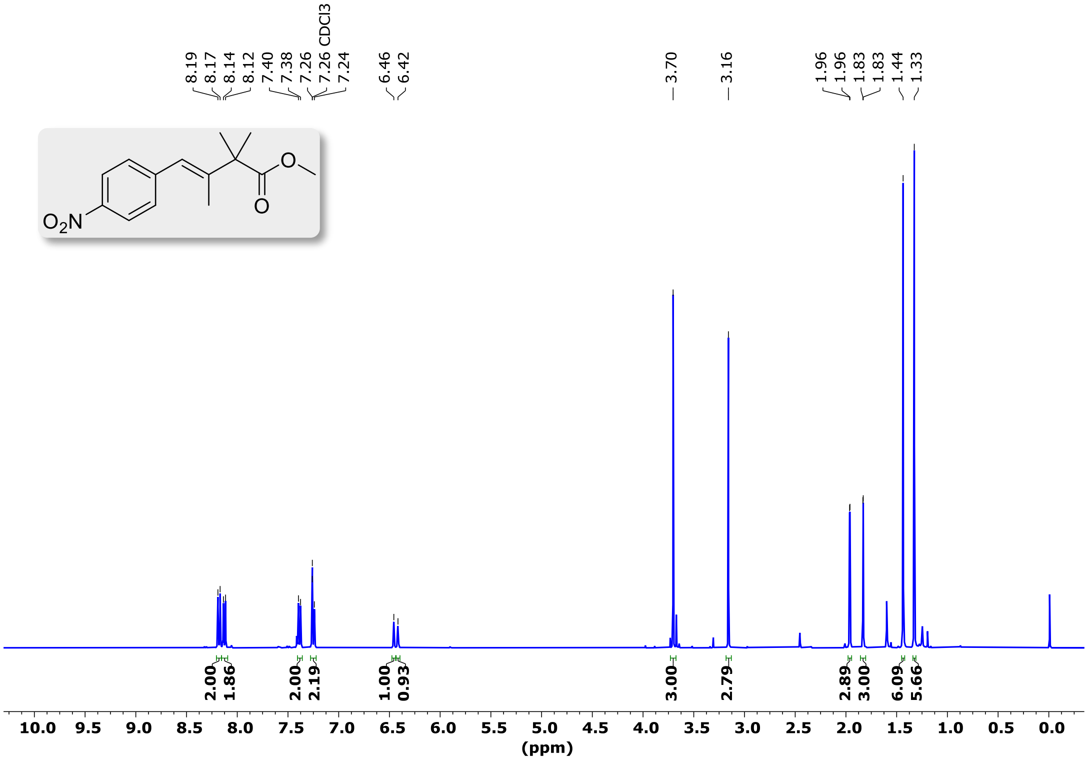


$^{1}$H NMR, CDCl$_3$, 400 MHz (ppm)

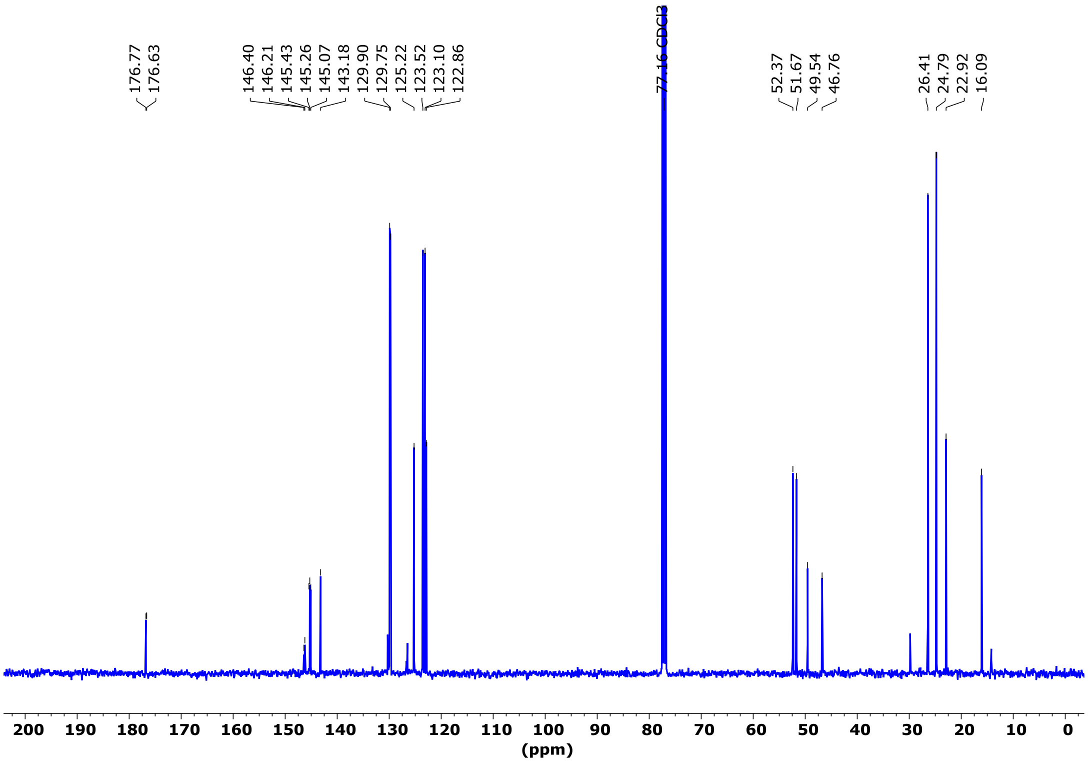


$^{13}$C{$^{1}$H} NMR, CDCl$_3$, 100 MHz (ppm)

## Methyl 2,2-dimethyl-4-nitro-3-(4-nitrophenyl)pentanoate (7z)
## minor diastereomer

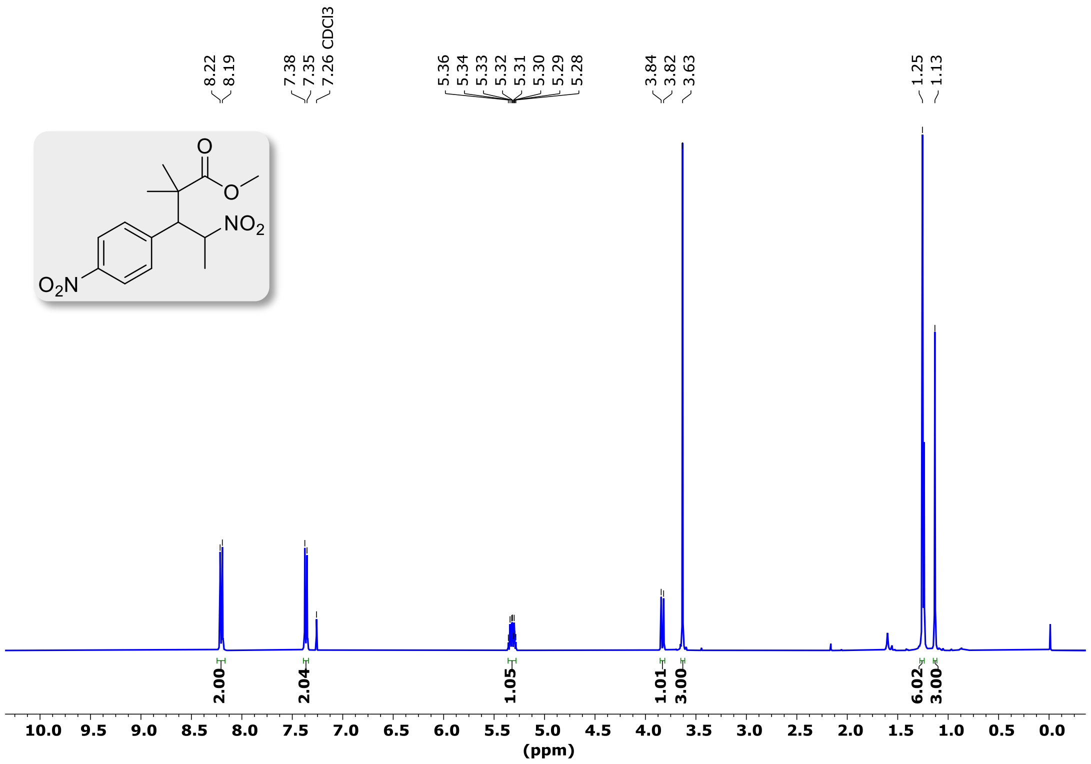


$^{1}H$ NMR, $CDCl_3$, 400 MHz (ppm)

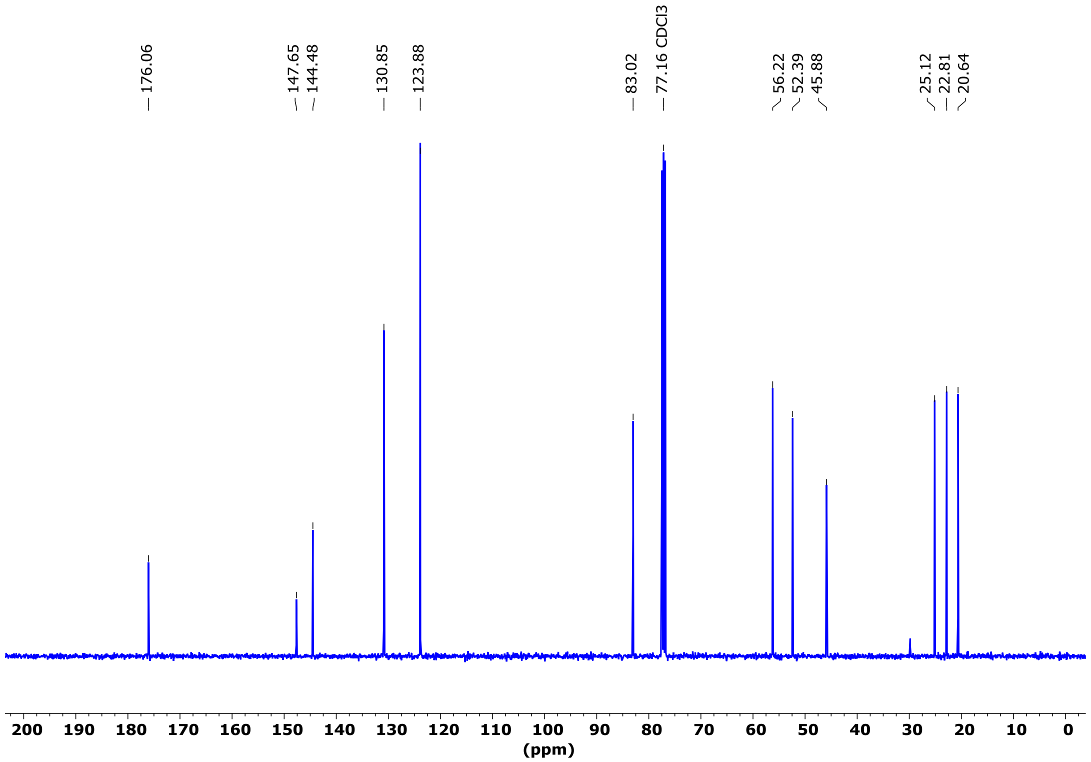


$^{13}C\{^{1}H\}$ NMR, $CDCl_3$, 100 MHz (ppm)

**Methyl 2,2-dimethyl-4-nitro-3-(4-nitrophenyl)pentanoate (7z)**
**major diastereomer**

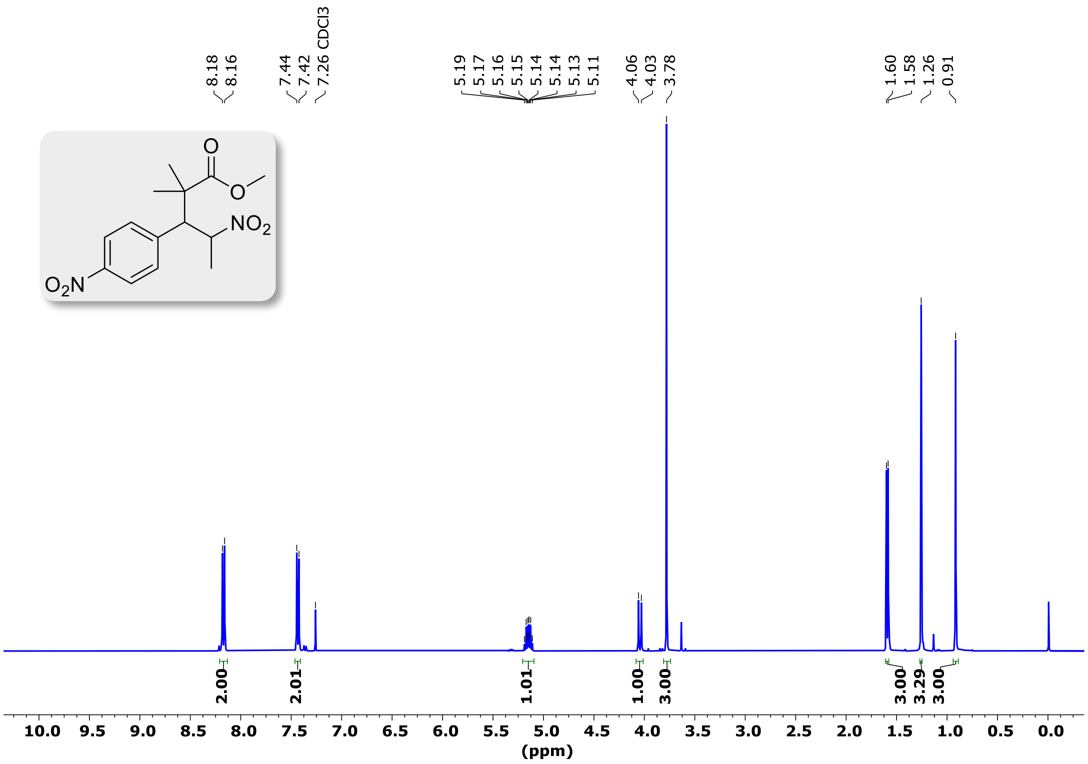


$^1$H NMR, $CDCl_3$, 400 MHz (ppm)

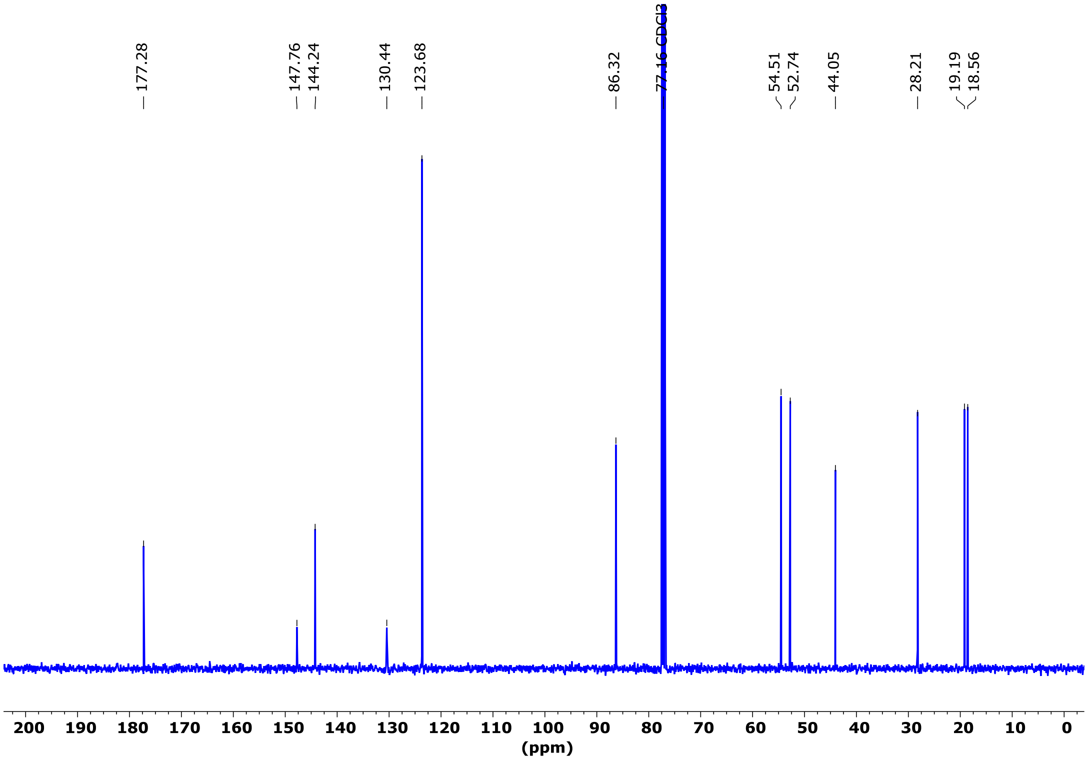


$^{13}C\{^1H\}$ NMR, $CDCl_3$, 100 MHz (ppm)

## Methyl (*E*)-2,2-dimethyl-4-(4-nitrophenyl)pent-3-enoate (6za)

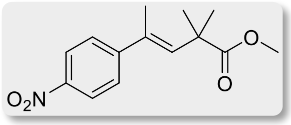

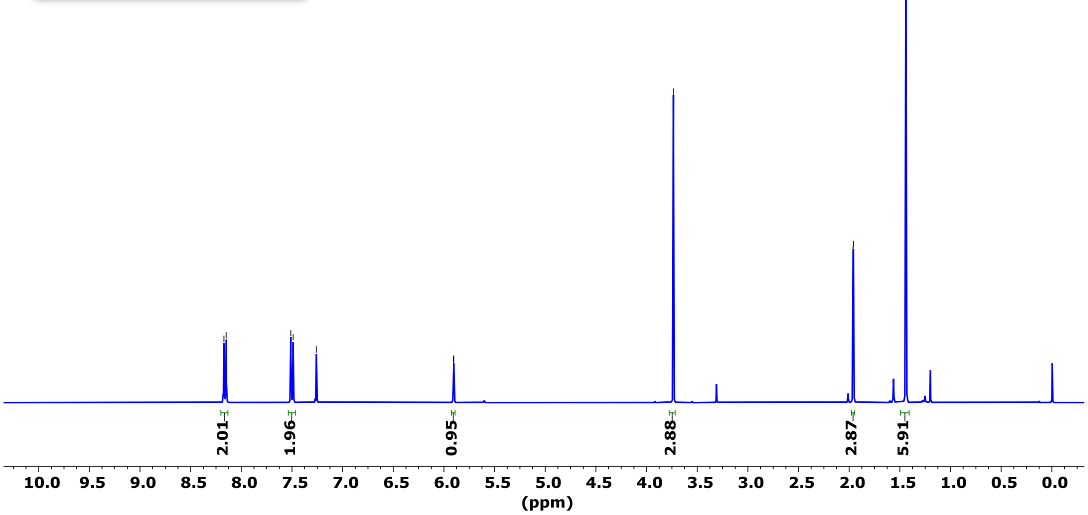


$^1$H NMR, $CDCl_3$, 400 MHz (ppm)

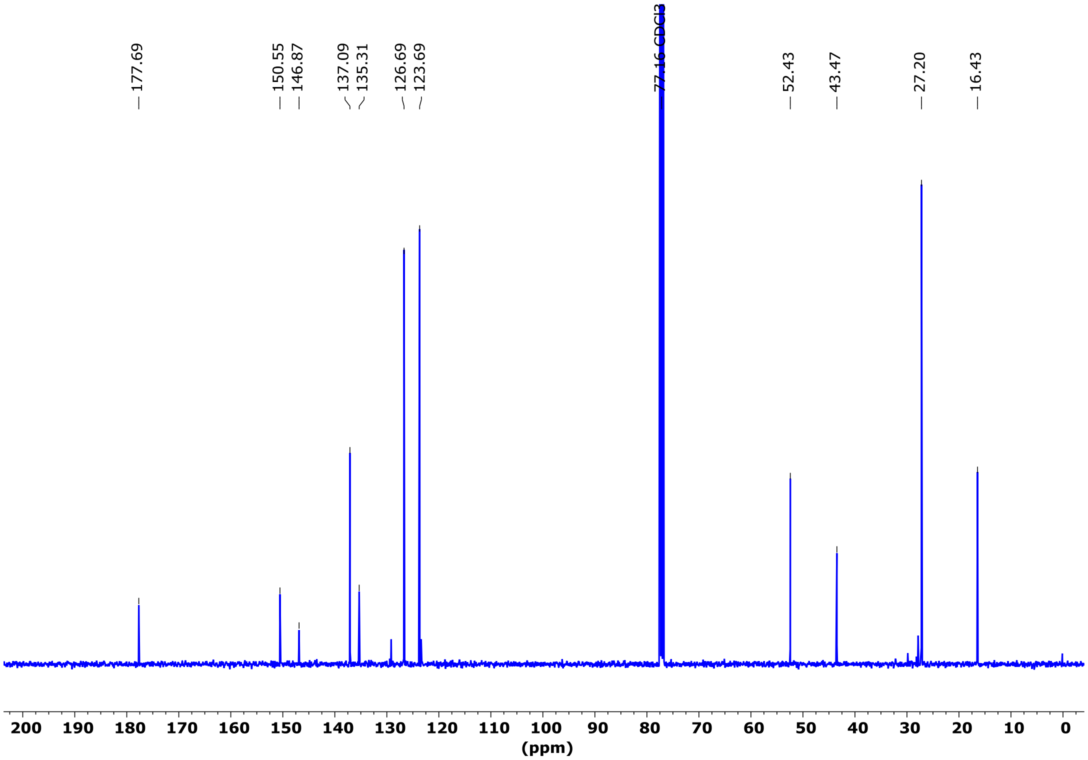


$^{13}$C{$^1$H} NMR, $CDCl_3$, 100 MHz (ppm)

## Methyl 2,2,3-trimethyl-4-nitro-3-(4-nitrophenyl)butanoate (7za)

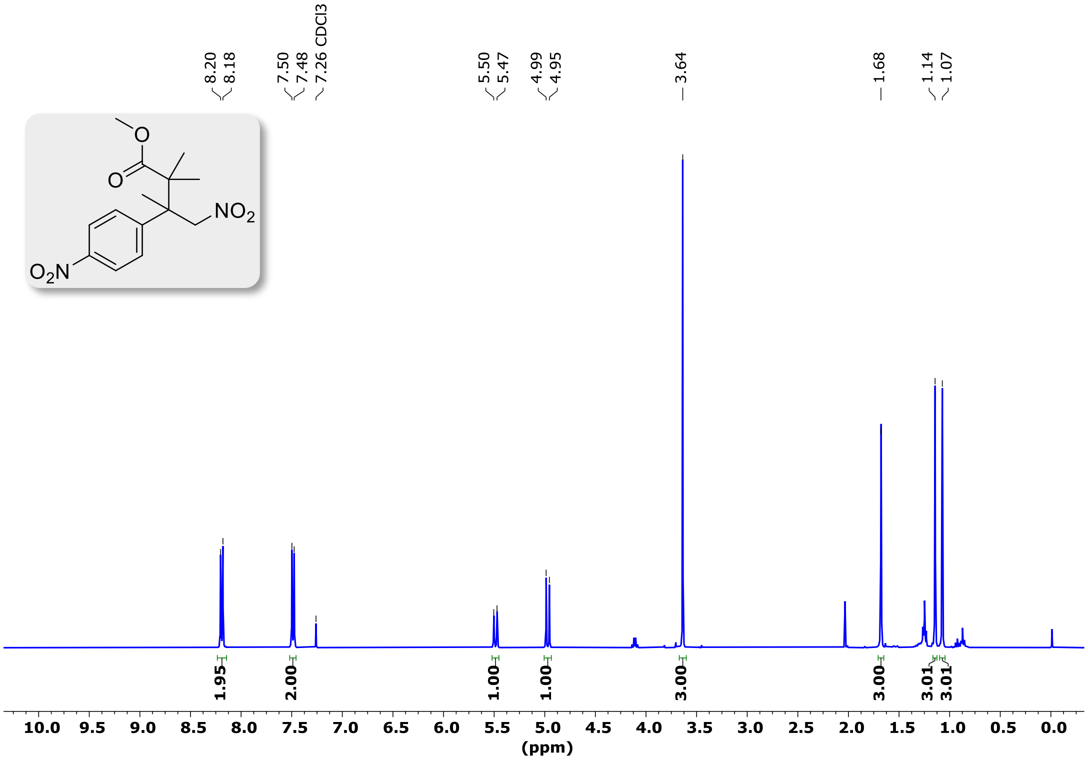


$^{1}H$ NMR, $CDCl_3$, 400 MHz (ppm)

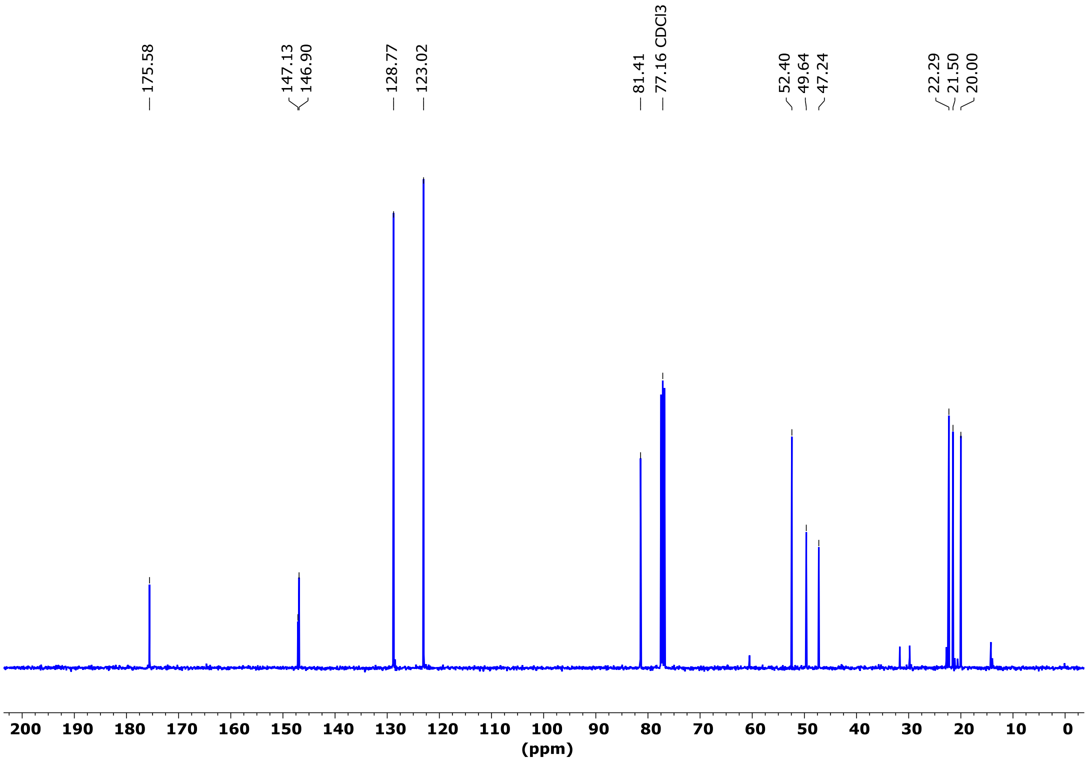


$^{13}C\{^{1}H\}$ NMR, $CDCl_3$, 100 MHz (ppm)

## (*E*)-2,2-Dimethyl-4-(2-nitrophenyl)but-3-enoic acid (8a)

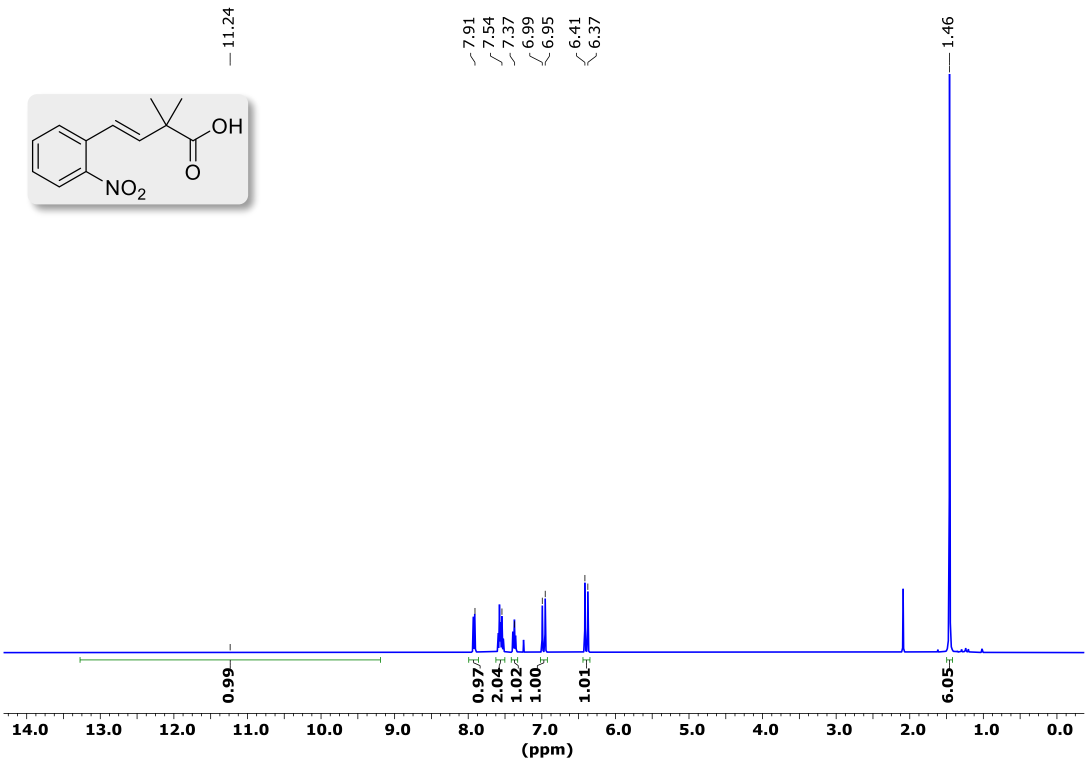


$^{1}$H NMR, $CDCl_3$, 400 MHz (ppm)

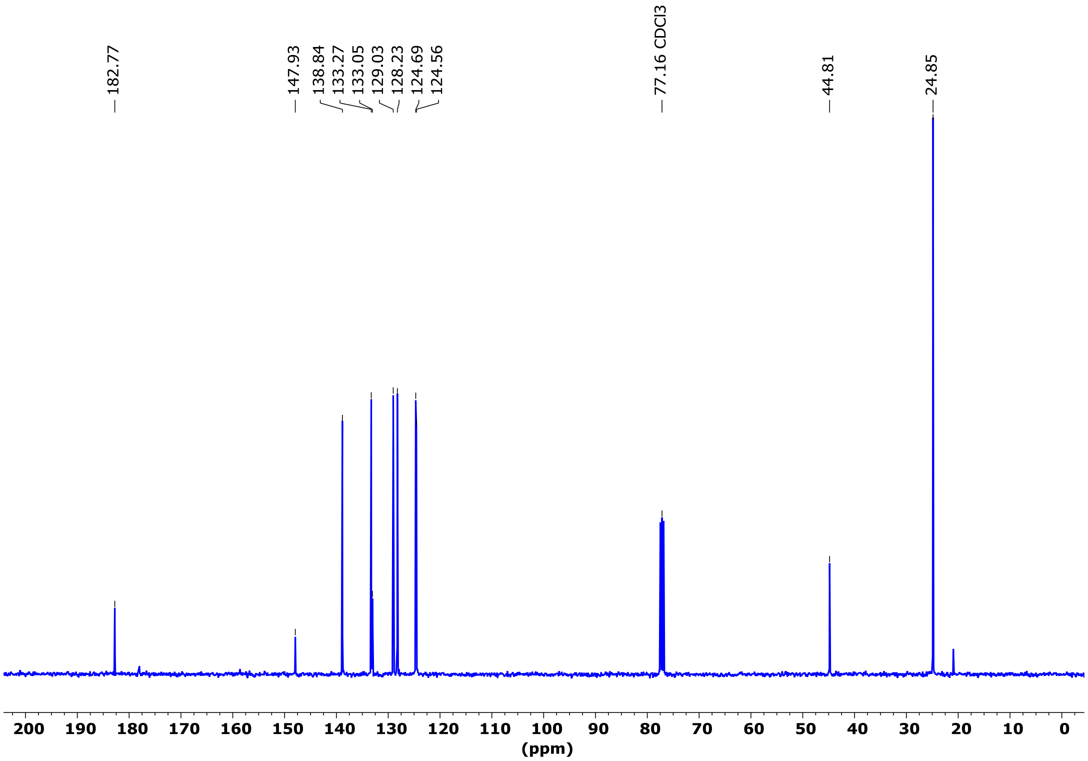


$^{13}$C{$^{1}$H} NMR, $CDCl_3$, 100 MHz (ppm)

## 2,2-Dimethyl-4-nitro-3-(2-nitrophenyl)butanoic acid (9a)

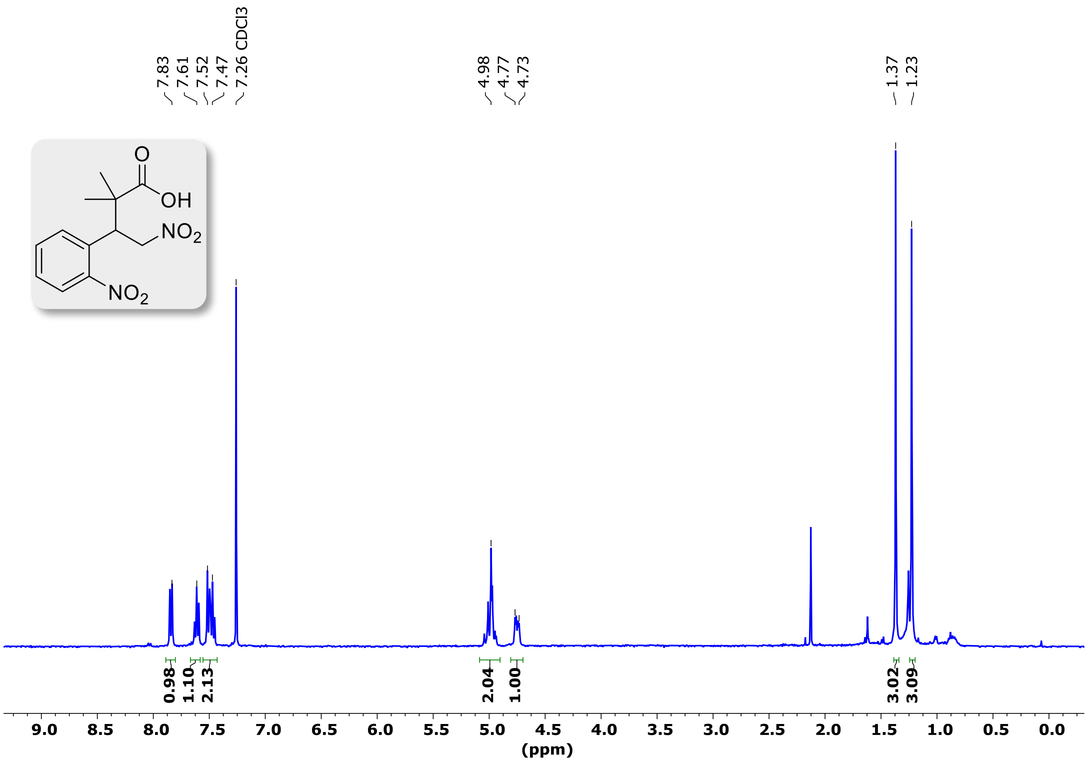


$^{1}$H NMR, $CDCl_3$, 400 MHz (ppm)

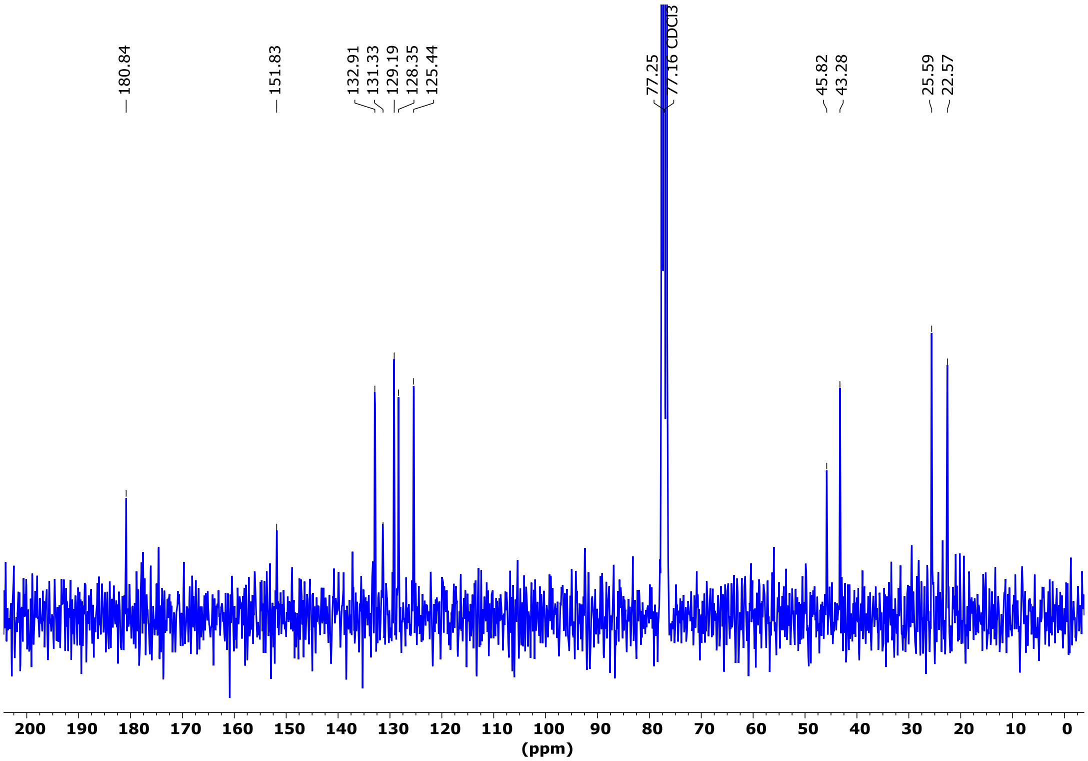


$^{13}$C{$^{1}$H} NMR, $CDCl_3$, 100 MHz (ppm)

## (*E*)-1-(2-Nitrostyryl)cyclopentane-1-carboxylic acid (8b)

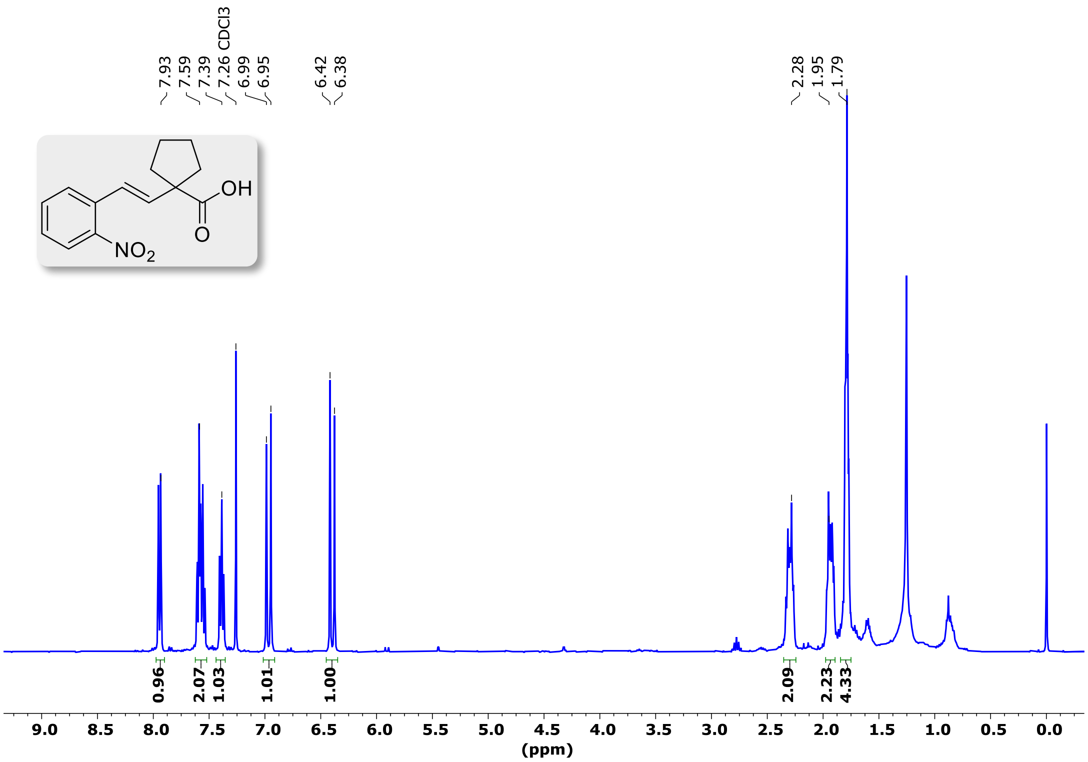


$^{1}$H NMR, $CDCl_3$, 400 MHz (ppm)

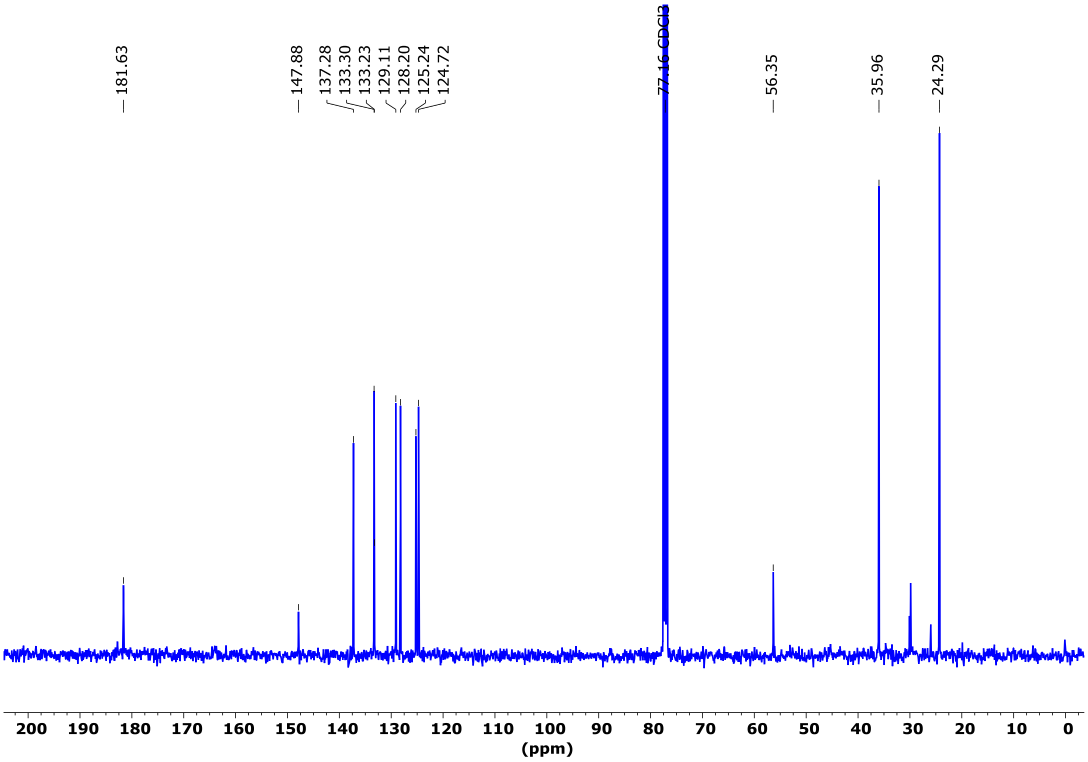


$^{13}$C{$^{1}$H} NMR, $CDCl_3$, 100 MHz (ppm)

## (*E*)-2-Ethyl-4-(2-nitrophenyl)but-3-enoic acid (8e)

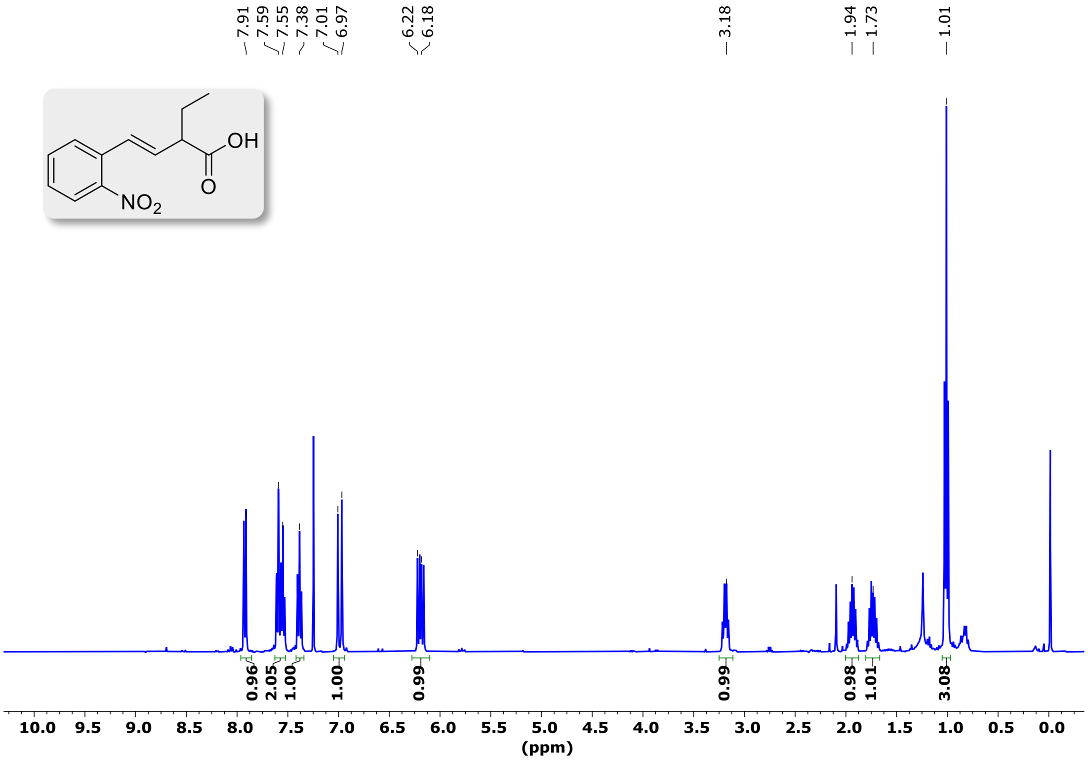


$^{1}$H NMR, CDCl$_3$, 400 MHz (ppm)

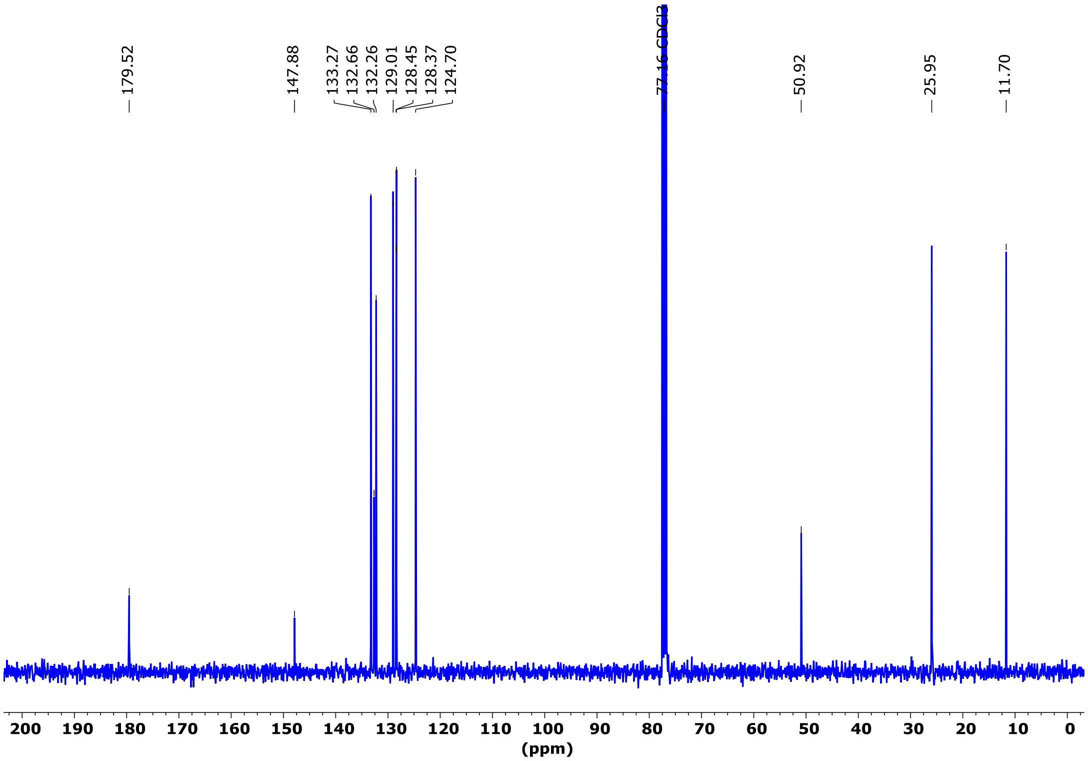


$^{13}$C{$^{1}$H} NMR, CDCl$_3$, 100 MHz (ppm)

## 2-Ethyl-4-nitro-3-(2-nitrophenyl)butanoic acid (9e)

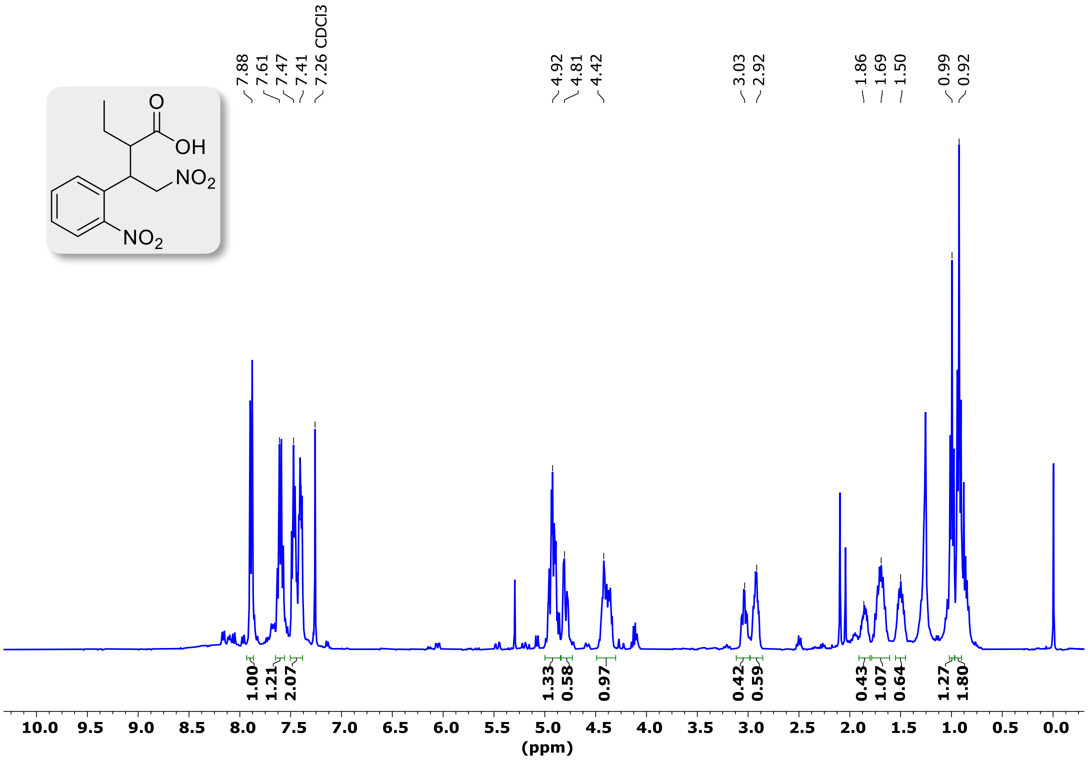


$^{1}$H NMR, $CDCl_3$, 400 MHz (ppm)

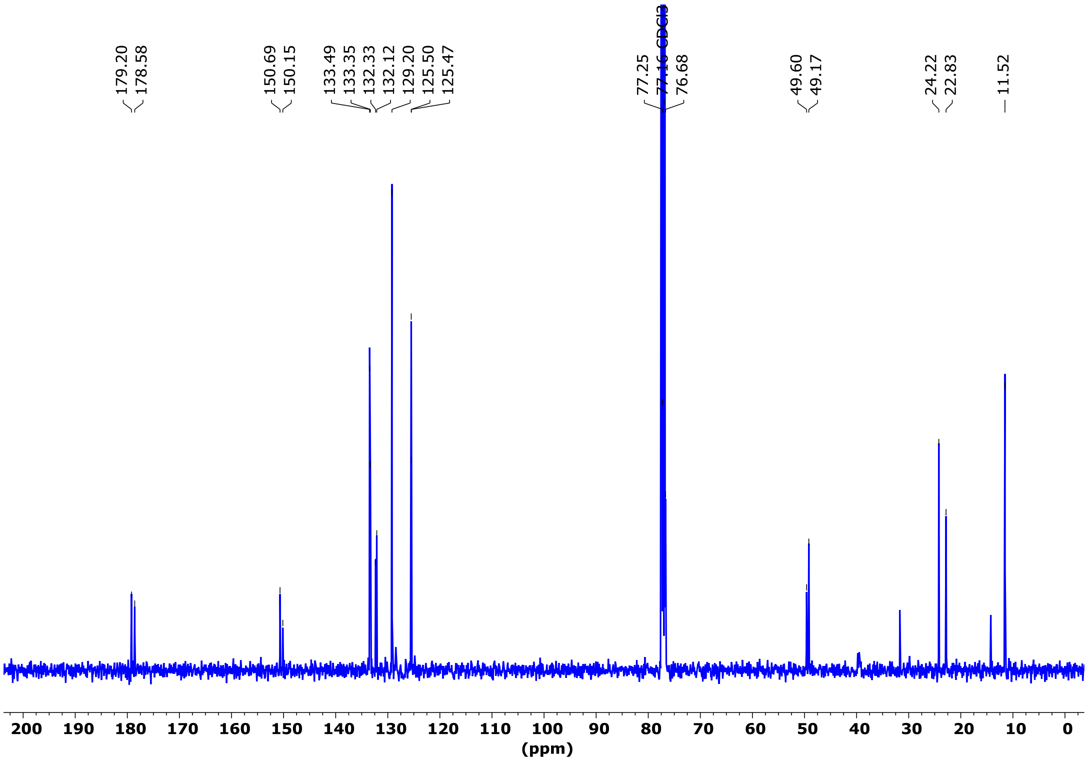


$^{13}$C{$^{1}$H} NMR, $CDCl_3$, 100 MHz (ppm)

**2-Methyl-2-(2-nitro-1-(2-nitrophenyl)ethyl)cyclopentan-1-one (9i)**
**major diastereomer**

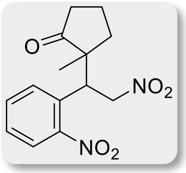


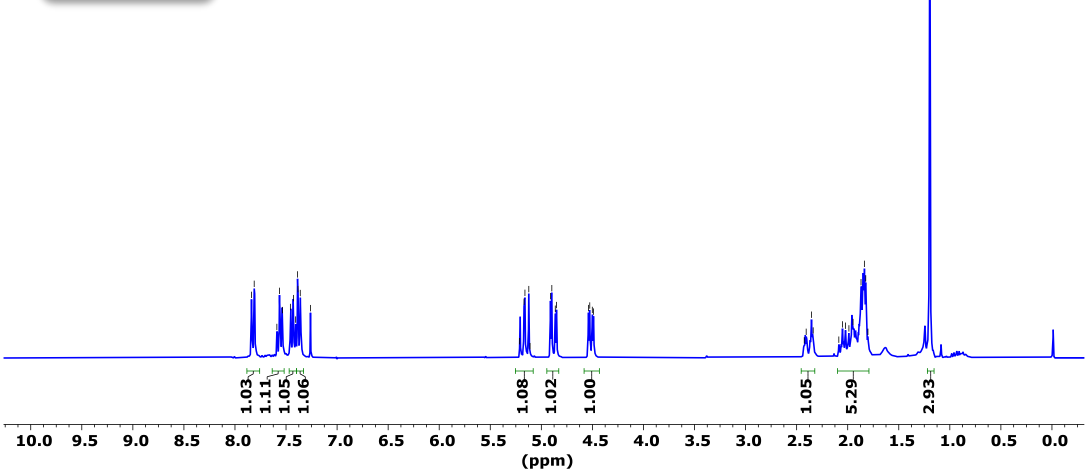


**$^{1}H$ NMR, $CDCl_3$, 400 MHz (ppm)**

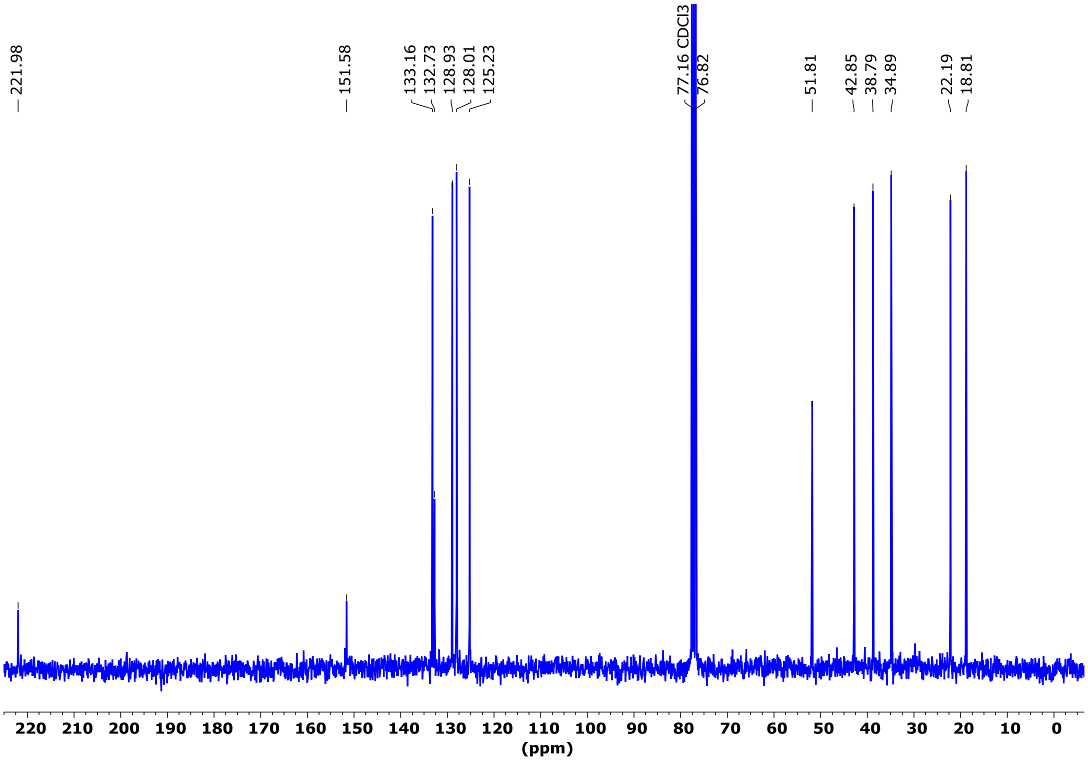


**$^{13}C\{^{1}H\}$ NMR, $CDCl_3$, 100 MHz (ppm)**

## 2-Methyl-2-(2-nitro-1-(2-nitrophenyl)ethyl)cyclopentan-1-one (9i)
## minor diastereomer

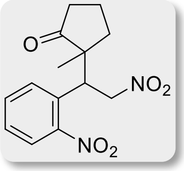


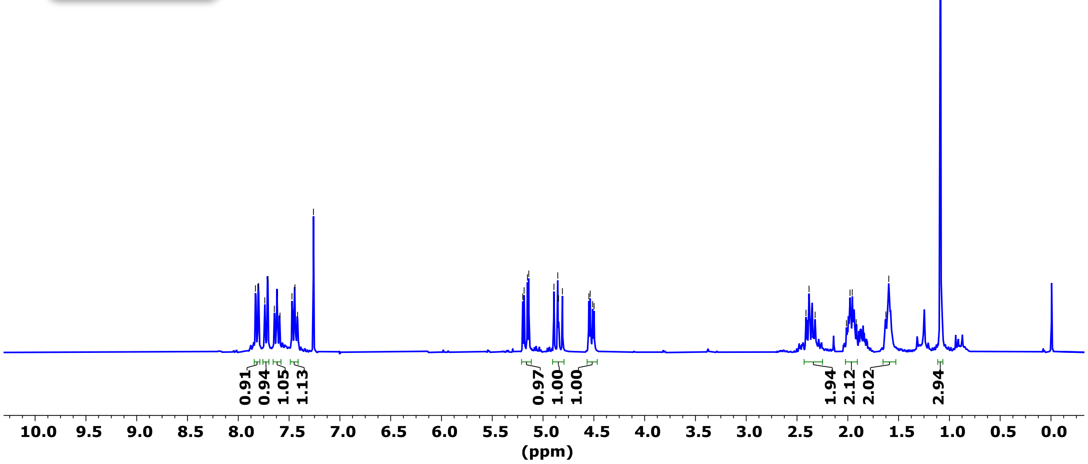


$^{1}H$ NMR, $CDCl_3$, 400 MHz (ppm)

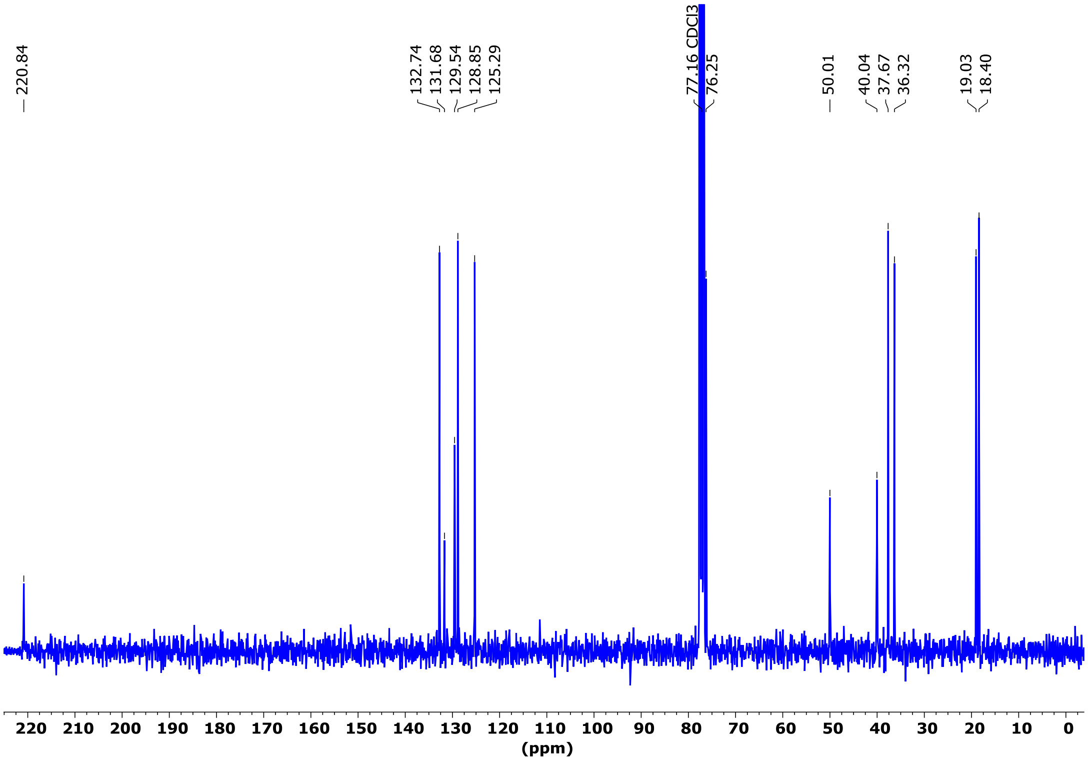


$^{13}C\{^{1}H\}$ NMR, $CDCl_3$, 100 MHz (ppm)

**Methyl 2-acetyl-4-nitro-3-(2-nitrophenyl)butanoate (9j)**

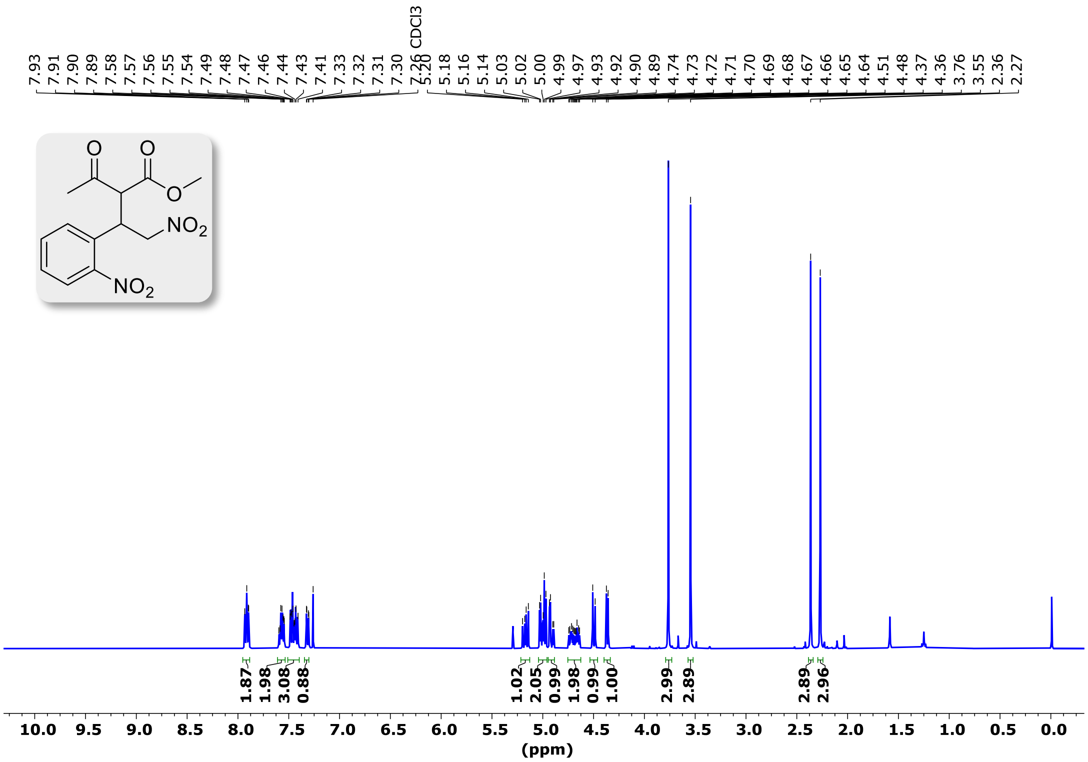


**$^{1}$H NMR, $CDCl_3$, 400 MHz (ppm)**

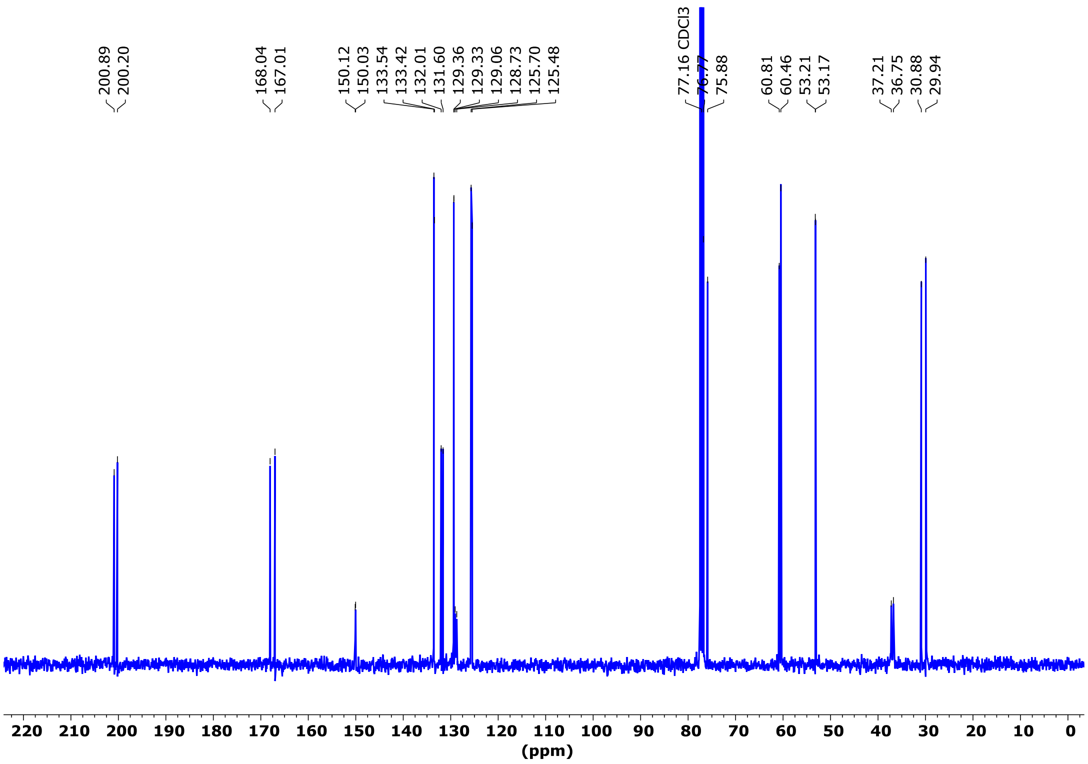


**$^{13}$C{$^{1}$H} NMR, $CDCl_3$, 100 MHz (ppm)**

## 9. References


1. Díaz-Salazar, H.; Rodríguez-Colín, J. C.; Vazquez-Chavez, J.; Hernández-Rodríguez, M. The Chameleonic Nature of the Nitro Group Applied to a Base-Promoted Cascade Reaction to Afford Indane-Fused Dihydrofurans. *J. Org. Chem.* **2023**, *88* (13), 8150–8162. https://doi.org/10.1021/acs.joc.3c00132.

2. Díaz-Salazar, H.; Rodríguez-Colín, J. C.; Vazquez-Chavez, J.; Hernández-Rodríguez, M. The Chameleonic Nature of the Nitro Group Applied to a Base-Promoted Cascade Reaction to Afford Indane-Fused Dihydrofurans. *J. Org. Chem.* **2023**, *88* (13), 8150–8162. https://doi.org/10.1021/acs.joc.3c00132.

3. Pechalrieu, D.; Dauzonne, D.; Arimondo, P. B.; Lopez, M. Synthesis of novel 3-halo-3-nitroflavanones and their activities as DNA methyltransferase inhibitors in cancer cells. *Eur. J. Med. Chem.* **2020**, *186*, 111829 https://doi.org/10.1016/j.ejmech.2019.111829

4. Liu, T. Q.; Zheng, F.; Ding, T. M.; Zhang, S. Y.; Lu Q. Design and synthesis of a novel quinoxaline diamine and its polyimides with high-Tg and red color. *Polymer*, **2019**, *179*, 121612. https://doi.org/10.1016/j.polymer.2019.121612

5. Karmarkar, S. N.; Kelkar, S. L.; Wadia, M. S.; A Simple Unusual One-Step Conversion of Aromatic Aldehydes into Nitriles. *Synthesis* **1985**, *5*, 510–512. https://doi.org/10.1055/s-1985-31253

6. Burkhard, J. A. New opportunities for four-membered heterocycles. **2012**. https://doi.org/10.3929/ethz-a-006834147

7. Yu, Y. B.; Cheng, Lei; Li, Y. P.; Fu, Y.; Zhu, S. F.; Zhou, Q. L. Enantioselective iridium-catalyzed hydrogenation of β, β-disubstituted nitroalkenes. *Chem. Commun.* **2016**, *52*, 4812–4815. https://doi.org/10.1039/C6CC01273F

8. (a) Ainsworth, C.; Kuo, Y. N. Ketene bis(trialkylsilyl) acetals: Synthesis, pyrolysis and spectral studies. *J. Organomet. Chem.*, **1972**, *46*, 73–87. https://doi.org/10.1016/S0022-328X(00)90476-5
(b) Paquette, L. A.; Parker, G. D.; Tei, T.; Dong S. Pestalotiopsin A. Enantioselective Construction of Potential Building Blocks Derived from Antipodal Cyclobutanol Intermediates, *J. Org. Chem.* **2007**, *72*, 19, 7125–7134. https://doi.org/10.1021/jo070861r

## 10. Internal coordinates of compounds 1.

**1a**
Img. Freq. = 0
E = -821.234460 Hartrees
HOMO = -0.28247 Hartrees
LUMO = -0.07506 Hartrees

| | | | |
|---|---|---|---|
| C | 0.04977 | -0.60156 | -0.31582 |
| H | -0.76303 | -1.26289 | -0.60348 |
| C | -0.26485 | 0.68249 | -0.16573 |
| H | 0.36885 | 1.49559 | 0.14170 |
| O | -1.80094 | 2.33287 | -0.40477 |
| O | -2.48337 | 0.31378 | -0.71730 |
| N | -1.62705 | 1.12962 | -0.45007 |
| C | 1.35117 | -1.23776 | -0.10342 |
| C | 1.34792 | -2.62723 | 0.07072 |
| C | 2.59125 | -0.55700 | -0.08044 |
| C | 2.51782 | -3.33533 | 0.29696 |
| H | 0.39968 | -3.15196 | 0.03535 |
| C | 3.76725 | -1.28618 | 0.13950 |
| C | 3.73344 | -2.65834 | 0.33346 |
| H | 2.48293 | -4.40841 | 0.43783 |
| H | 4.70948 | -0.75278 | 0.14995 |
| H | 4.65595 | -3.19992 | 0.50365 |
| C | 2.70162 | 0.84705 | -0.30905 |
| C | 2.81261 | 2.03195 | -0.51289 |
| C | 2.86962 | 3.44136 | -0.74634 |
| C | 1.68706 | 4.15522 | -0.98515 |
| C | 4.09579 | 4.11758 | -0.73232 |
| C | 1.73763 | 5.52480 | -1.20374 |
| H | 0.73532 | 3.63561 | -0.99799 |
| C | 4.13591 | 5.48692 | -0.95391 |
| H | 5.00589 | 3.56038 | -0.54724 |
| C | 2.95909 | 6.19255 | -1.18929 |
| H | 0.82022 | 6.07102 | -1.38610 |
| H | 5.08716 | 6.00512 | -0.94261 |
| H | 2.99391 | 7.26156 | -1.36169 |

**1b**
Img. Freq. = 0
E = -718.549817 Hartrees
HOMO = -0.34108 Hartrees
LUMO = -0.08709 Hartrees

| | | | |
|---|---|---|---|
| C | 0.01666 | -0.56873 | -0.06892 |
| H | -0.85790 | -1.10563 | -0.42332 |
| C | -0.17011 | 0.64114 | 0.43796 |
| H | 0.56124 | 1.31271 | 0.86505 |
| O | -1.60748 | 2.32305 | 0.94566 |
| O | -2.44249 | 0.56092 | 0.02793 |
| N | -1.51967 | 1.21336 | 0.46634 |
| C | 1.30211 | -1.28267 | -0.10125 |
| C | 1.28653 | -2.66202 | 0.12797 |
| C | 2.55683 | -0.69102 | -0.26758 |
| C | 2.46246 | -3.39628 | 0.21338 |
| H | 0.32997 | -3.15417 | 0.26063 |
| C | 3.74207 | -1.39742 | -0.16384 |
| C | 3.69322 | -2.76494 | 0.07611 |
| H | 2.41558 | -4.46312 | 0.39288 |
| H | 4.68147 | -0.87358 | -0.28605 |
| H | 4.61280 | -3.33186 | 0.14557 |
| O | 3.49015 | 1.39066 | -0.00834 |
| O | 1.99602 | 1.14266 | -1.54005 |
| N | 2.68287 | 0.73650 | -0.63206 |

**1c**
Img. Freq. = 0
E = -851.117703 Hartrees
HOMO = -0.33306 Hartrees
LUMO = -0.07796 Hartrees

| | | | |
|---|---|---|---|
| C | 0.07953 | -0.53506 | -0.16847 |
| H | -0.76312 | -1.11990 | -0.52678 |
| C | -0.19363 | 0.69746 | 0.23688 |
| H | 0.46687 | 1.43891 | 0.65624 |
| O | -1.74799 | 2.32832 | 0.52101 |
| O | -2.43423 | 0.45944 | -0.30074 |

**1d**
Img. Freq. = 0
E = -3087.639711 Hartrees
HOMO = -0.31844 Hartrees
LUMO = -0.07500 Hartrees

| | | | |
|---|---|---|---|
| C | 0.15689 | -0.48878 | 0.20019 |
| H | -0.66771 | -1.05311 | 0.62688 |
| C | -0.14873 | 0.73614 | -0.21465 |
| H | 0.46934 | 1.47185 | -0.69881 |
| O | -1.75023 | 2.32211 | -0.49000 |
| O | -2.34332 | 0.50299 | 0.49790 |

| | | | |
|---|---|---|---|
| N | -1.57274 | 1.19092 | 0.13863 |
| C | 1.37004 | -1.24477 | -0.10429 |
| C | 1.28534 | -2.61547 | 0.16395 |
| C | 2.64955 | -0.68916 | -0.28709 |
| C | 2.41930 | -3.40312 | 0.30320 |
| H | 0.30387 | -3.06130 | 0.27832 |
| C | 3.78418 | -1.48229 | -0.15563 |
| C | 3.67533 | -2.83397 | 0.14865 |
| H | 2.31816 | -4.45865 | 0.52349 |
| H | 4.75963 | -1.04032 | -0.30893 |
| H | 4.56918 | -3.43698 | 0.24703 |
| C | 2.85529 | 0.76246 | -0.64427 |
| F | 2.01800 | 1.17946 | -1.60032 |
| F | 2.67200 | 1.56818 | 0.42098 |
| F | 4.09676 | 0.99551 | -1.08356 |

**1e**
Img. Freq. = 0
E = -553.378362 Hartrees
HOMO = -0.31197 Hartrees
LUMO = -0.06944 Hartrees

| | | | |
|---|---|---|---|
| C | 0.11620 | -0.53000 | 0.11406 |
| H | -0.73605 | -1.13402 | 0.41504 |
| C | -0.17808 | 0.74434 | -0.14377 |
| H | 0.44966 | 1.55151 | -0.47492 |
| O | -1.76309 | 2.36538 | -0.25545 |
| O | -2.41506 | 0.41136 | 0.37084 |
| N | -1.56432 | 1.19560 | 0.00625 |
| C | 1.38638 | -1.25239 | 0.03237 |
| C | 1.29471 | -2.64445 | 0.18881 |
| C | 2.65422 | -0.66738 | -0.17772 |
| C | 2.41258 | -3.46009 | 0.12547 |
| H | 0.31887 | -3.08532 | 0.36022 |
| C | 3.76715 | -1.50510 | -0.23006 |
| C | 3.65875 | -2.88350 | -0.08672 |
| H | 2.31257 | -4.53161 | 0.24498 |
| H | 4.74423 | -1.06143 | -0.38601 |
| H | 4.54711 | -3.50192 | -0.13530 |
| C | 2.85528 | 0.81472 | -0.33470 |
| H | 2.41113 | 1.17953 | -1.26498 |
| H | 2.40729 | 1.36808 | 0.49421 |
| H | 3.91881 | 1.04988 | -0.36219 |

**1g**
Img. Freq. = 0
E = -718.559374 Hartrees
HOMO = -0.34041 Hartrees
LUMO = -0.09037 Hartrees

| | | | |
|---|---|---|---|
| C | 0.08304 | -0.59567 | 0.01566 |
| H | -0.82785 | -1.18441 | 0.07087 |

| | | | |
|---|---|---|---|
| N | -1.52685 | 1.21372 | -0.04934 |
| C | 1.42385 | -1.22217 | 0.10449 |
| C | 1.31327 | -2.62247 | 0.14037 |
| C | 2.71679 | -0.68845 | -0.01028 |
| C | 2.41540 | -3.45326 | 0.03155 |
| H | 0.32432 | -3.05384 | 0.24696 |
| C | 3.83097 | -1.51406 | -0.10765 |
| C | 3.68170 | -2.89454 | -0.09562 |
| H | 2.28750 | -4.52805 | 0.05326 |
| H | 4.81379 | -1.06855 | -0.18801 |
| H | 4.55766 | -3.52654 | -0.17540 |
| Br | 3.06177 | 1.17888 | 0.01888 |

**1f**
Img. Freq. = 0
E = -628.588791 Hartrees
HOMO = -0.29713 Hartrees
LUMO = -0.06333 Hartrees

| | | | |
|---|---|---|---|
| C | 0.15048 | -0.55720 | 0.05434 |
| H | -0.74861 | -1.13963 | 0.23616 |
| C | -0.03673 | 0.75366 | -0.10103 |
| H | 0.68979 | 1.52293 | -0.28819 |
| O | -1.49932 | 2.48857 | -0.16684 |
| O | -2.32653 | 0.53383 | 0.19210 |
| N | -1.39682 | 1.28683 | -0.01652 |
| C | 1.39042 | -1.32292 | 0.01471 |
| C | 1.30283 | -2.70569 | 0.20913 |
| C | 2.66710 | -0.76111 | -0.20623 |
| C | 2.42399 | -3.52206 | 0.18961 |
| H | 0.32300 | -3.13818 | 0.37905 |
| C | 3.79662 | -1.57715 | -0.22666 |
| C | 3.66954 | -2.94887 | -0.02931 |
| H | 2.32669 | -4.58885 | 0.34296 |
| H | 4.77719 | -1.15479 | -0.39516 |
| H | 4.55849 | -3.56805 | -0.04846 |
| O | 2.71400 | 0.57656 | -0.38959 |
| C | 3.97353 | 1.18590 | -0.61399 |
| H | 4.63909 | 1.03179 | 0.23947 |
| H | 4.43856 | 0.79709 | -1.52376 |
| H | 3.77347 | 2.24793 | -0.73262 |

**1h**
Img. Freq. = 0
E = -606.308586 Hartrees
HOMO = -0.33652 Hartrees
LUMO = -0.08797 Hartrees

| | | | |
|---|---|---|---|
| C | 0.07812 | -0.60236 | -0.01056 |
| H | -0.83594 | -1.18893 | -0.01386 |

| | | | |
|---|---|---|---|
| C | -0.05389 | 0.72485 | -0.01541 |
| H | 0.71544 | 1.48089 | -0.05757 |
| O | -1.43136 | 2.52793 | -0.03580 |
| O | -2.35822 | 0.58522 | 0.07151 |
| N | -1.39301 | 1.31643 | 0.00922 |
| C | 1.35455 | -1.32515 | -0.00241 |
| C | 1.33528 | -2.71146 | 0.18513 |
| C | 2.58231 | -0.68682 | -0.20106 |
| C | 2.51074 | -3.45368 | 0.18586 |
| H | 0.38405 | -3.21005 | 0.33391 |
| C | 3.73391 | -1.44879 | -0.19648 |
| H | 2.66111 | 0.37950 | -0.36568 |
| C | 3.73235 | -2.82401 | -0.00626 |
| H | 2.47497 | -4.52522 | 0.33470 |
| H | 4.66683 | -3.36817 | -0.01495 |
| N | 5.03107 | -0.76370 | -0.41078 |
| O | 6.02732 | -1.45038 | -0.41100 |
| O | 5.00782 | 0.43594 | -0.57073 |

**1i**

Img. Freq. = 0

E = -718.559436 Hartrees

HOMO = -0.34439 Hartrees

LUMO = -0.10277 Hartrees

| | | | |
|---|---|---|---|
| C | 0.08930 | -0.62531 | -0.02832 |
| H | -0.82562 | -1.20936 | -0.06364 |
| C | -0.03710 | 0.69475 | 0.04024 |
| H | 0.73972 | 1.44095 | 0.10857 |
| O | -1.39398 | 2.51311 | 0.07471 |
| O | -2.34367 | 0.57990 | -0.00763 |
| N | -1.37042 | 1.30084 | 0.03489 |
| C | 1.36101 | -1.35604 | -0.03234 |
| C | 1.33230 | -2.73738 | 0.18601 |
| C | 2.59185 | -0.72250 | -0.24888 |
| C | 2.50480 | -3.47877 | 0.20942 |
| H | 0.38173 | -3.23343 | 0.34332 |
| C | 3.77032 | -1.44817 | -0.23124 |
| H | 2.63198 | 0.34089 | -0.44937 |
| C | 3.70188 | -2.81489 | 0.00216 |
| H | 2.50343 | -4.54645 | 0.38088 |
| H | 4.73083 | -0.98070 | -0.39963 |
| O | 5.99117 | -2.99184 | -0.17449 |
| O | 4.87342 | -4.78482 | 0.22721 |
| N | 4.95981 | -3.59543 | 0.01942 |

**1k**

Img. Freq. = 0

E = -973.673190 Hartrees

HOMO = -0.31371 Hartrees

LUMO = -0.07926 Hartrees

| | | | |
|---|---|---|---|
| C | 0.07805 | -0.60173 | 0.05071 |

| | | | |
|---|---|---|---|
| C | -0.05386 | 0.71889 | 0.01718 |
| H | 0.71907 | 1.47174 | 0.04556 |
| O | -1.42222 | 2.52900 | 0.02383 |
| O | -2.36024 | 0.58881 | 0.01048 |
| N | -1.39026 | 1.31624 | 0.01689 |
| C | 1.34768 | -1.33566 | -0.01544 |
| C | 1.32183 | -2.71832 | 0.19274 |
| C | 2.57755 | -0.70604 | -0.22329 |
| C | 2.49660 | -3.46082 | 0.21123 |
| H | 0.36861 | -3.21208 | 0.34516 |
| C | 3.75131 | -1.45315 | -0.20769 |
| H | 2.63519 | 0.35877 | -0.41202 |
| C | 3.71753 | -2.83421 | 0.01329 |
| H | 2.45913 | -4.52987 | 0.37774 |
| H | 4.64203 | -3.39749 | 0.02089 |
| C | 5.01255 | -0.79791 | -0.42578 |
| N | 6.02222 | -0.27593 | -0.59900 |

**1j**

Img. Freq. = 0

E = -606.309060 Hartrees

HOMO = -0.33295 Hartrees

LUMO = -0.09470 Hartrees

| | | | |
|---|---|---|---|
| C | 0.07779 | -0.61605 | -0.00866 |
| H | -0.83822 | -1.19959 | -0.00799 |
| C | -0.04863 | 0.70602 | 0.01945 |
| H | 0.72833 | 1.45458 | 0.04529 |
| O | -1.40608 | 2.52406 | 0.02701 |
| O | -2.35549 | 0.58919 | 0.02247 |
| N | -1.38148 | 1.31086 | 0.02317 |
| C | 1.34610 | -1.35048 | -0.01772 |
| C | 1.31454 | -2.73236 | 0.19473 |
| C | 2.58034 | -0.72357 | -0.23329 |
| C | 2.48425 | -3.47629 | 0.21057 |
| H | 0.36248 | -3.22566 | 0.35322 |
| C | 3.75284 | -1.45655 | -0.22234 |
| H | 2.62681 | 0.34130 | -0.42534 |
| C | 3.70646 | -2.83657 | 0.00326 |
| H | 2.45826 | -4.54527 | 0.37910 |
| H | 4.70703 | -0.97411 | -0.39180 |
| C | 4.92422 | -3.59884 | 0.01433 |
| N | 5.89913 | -4.20869 | 0.02353 |

**1l**

Img. Freq. = 0

E = -3087.644673 Hartrees

HOMO = -0.31114 Hartrees

LUMO = -0.07991 Hartrees

| | | | |
|---|---|---|---|
| C | 0.07418 | -0.60559 | 0.01880 |

| | | | |
|---|---|---|---|
| H | -0.83215 | -1.18310 | 0.16658 |
| C | -0.06338 | 0.71676 | -0.04872 |
| H | 0.70234 | 1.46795 | -0.16421 |
| O | -1.43684 | 2.52125 | -0.10306 |
| O | -2.36294 | 0.58937 | 0.12713 |
| N | -1.39693 | 1.31155 | -0.00347 |
| C | 1.34213 | -1.33742 | 0.02245 |
| C | 1.30831 | -2.72613 | 0.18023 |
| C | 2.58315 | -0.71241 | -0.15647 |
| C | 2.47408 | -3.47952 | 0.16431 |
| H | 0.35484 | -3.22367 | 0.31788 |
| C | 3.75337 | -1.45109 | -0.17506 |
| H | 2.64251 | 0.36147 | -0.28592 |
| C | 3.68882 | -2.83273 | -0.01346 |
| H | 2.44549 | -4.55434 | 0.28696 |
| H | 4.71274 | -0.96992 | -0.31415 |
| Cl | 5.15689 | -3.76141 | -0.03763 |

**1m**

Img. Freq. = 0
E = -821.234293 Hartrees
HOMO = -0.28052 Hartrees
LUMO = -0.08082 Hartrees

| | | | |
|---|---|---|---|
| C | 0.14310 | -0.64958 | 0.01869 |
| H | -0.77138 | -1.23321 | 0.07384 |
| C | 0.00797 | 0.67381 | -0.01294 |
| H | 0.78047 | 1.42574 | -0.05421 |
| O | -1.35771 | 2.48496 | -0.03451 |
| O | -2.29681 | 0.54867 | 0.07005 |
| N | -1.32381 | 1.27132 | 0.00978 |
| C | 1.40603 | -1.38479 | 0.00027 |
| C | 1.37280 | -2.77263 | 0.17565 |
| C | 2.64781 | -0.76081 | -0.18545 |
| C | 2.53914 | -3.52004 | 0.17751 |
| H | 0.41789 | -3.26726 | 0.31480 |
| C | 3.81488 | -1.49977 | -0.18687 |
| H | 2.70304 | 0.31026 | -0.33905 |
| C | 3.77700 | -2.89176 | -0.00275 |
| H | 2.50438 | -4.59317 | 0.31638 |
| H | 4.77124 | -1.01360 | -0.33297 |
| C | 4.98351 | -3.65269 | -0.00474 |
| C | 6.00590 | -4.29434 | -0.00558 |
| C | 7.21650 | -5.05381 | -0.00741 |
| C | 7.17898 | -6.44454 | 0.15647 |
| C | 8.45134 | -4.41346 | -0.17281 |
| C | 8.35719 | -7.17769 | 0.15439 |
| H | 6.22252 | -6.93635 | 0.28404 |
| C | 9.62493 | -5.15392 | -0.17367 |
| H | 8.47646 | -3.33810 | -0.29909 |
| C | 9.58145 | -6.53576 | -0.01037 |
| H | 8.32058 | -8.25289 | 0.28167 |
| H | 10.57625 | -4.65181 | -0.30188 |

| | | | |
|---|---|---|---|
| H | -0.83983 | -1.18974 | 0.07506 |
| C | -0.06072 | 0.71681 | -0.01327 |
| H | 0.71119 | 1.46950 | -0.05469 |
| O | -1.42762 | 2.52703 | -0.03432 |
| O | -2.36529 | 0.58967 | 0.07049 |
| N | -1.39397 | 1.31387 | 0.00998 |
| C | 1.33924 | -1.34044 | 0.00063 |
| C | 1.30870 | -2.72542 | 0.18750 |
| C | 2.57796 | -0.71710 | -0.19642 |
| C | 2.47674 | -3.47645 | 0.19070 |
| H | 0.35654 | -3.22272 | 0.33538 |
| C | 3.75096 | -1.45278 | -0.19777 |
| H | 2.63361 | 0.35244 | -0.35991 |
| C | 3.69010 | -2.83032 | -0.00132 |
| H | 2.44586 | -4.54782 | 0.33851 |
| H | 4.70617 | -0.96864 | -0.35274 |
| Br | 5.29354 | -3.83739 | -0.00272 |

**1n**

Img. Freq. = 0
E = -741.935777 Hartrees
HOMO = -0.32358 Hartrees
LUMO = -0.08458 Hartrees

| | | | |
|---|---|---|---|
| C | 0.10331 | -0.61077 | 0.02983 |
| H | -0.82265 | -1.17469 | 0.09599 |
| C | 0.00286 | 0.71451 | 0.02903 |
| H | 0.79433 | 1.44682 | -0.00991 |
| O | -1.31525 | 2.56092 | 0.06845 |
| O | -2.30255 | 0.64789 | 0.16119 |
| N | -1.31402 | 1.34691 | 0.09156 |
| C | 1.35331 | -1.37278 | -0.02930 |
| C | 1.30000 | -2.75281 | 0.19410 |
| C | 2.59100 | -0.77502 | -0.30036 |
| C | 2.45550 | -3.51884 | 0.16287 |
| H | 0.34405 | -3.22241 | 0.39676 |
| C | 3.74621 | -1.53710 | -0.33510 |
| H | 2.65204 | 0.28818 | -0.49872 |
| C | 3.68035 | -2.91211 | -0.10003 |
| H | 2.42707 | -4.58732 | 0.33773 |
| H | 4.70239 | -1.07716 | -0.54735 |
| C | 4.89623 | -3.77913 | -0.12608 |
| O | 6.01142 | -3.08810 | -0.39139 |
| C | 7.21387 | -3.86120 | -0.43026 |
| H | 8.00977 | -3.15632 | -0.65343 |
| H | 7.38221 | -4.34279 | 0.53289 |
| H | 7.14466 | -4.62490 | -1.20482 |
| O | 4.88190 | -4.96538 | 0.06892 |

| | | | |
|---|---|---|---|
| H | 10.49941 | -7.11107 | -0.01143 |

**1o**

Img. Freq. = 0
E = -514.073235 Hartrees
HOMO = -0.31548 Hartrees
LUMO = -0.07288 Hartrees

| | | | |
|---|---|---|---|
| C | 0.07453 | -0.60512 | 0.01893 |
| H | -0.83905 | -1.19008 | 0.07535 |
| C | -0.06221 | 0.71732 | -0.01330 |
| H | 0.70934 | 1.47017 | -0.05517 |
| O | -1.42994 | 2.52730 | -0.03451 |
| O | -2.36731 | 0.59032 | 0.07031 |
| N | -1.39508 | 1.31376 | 0.00980 |
| C | 1.34050 | -1.34004 | 0.00053 |
| C | 1.30627 | -2.72534 | 0.18815 |
| C | 2.57837 | -0.71408 | -0.19650 |
| C | 2.47921 | -3.47040 | 0.18983 |
| H | 0.35091 | -3.21700 | 0.33552 |
| C | 3.74754 | -1.45771 | -0.19643 |
| H | 2.62864 | 0.35593 | -0.35959 |
| C | 3.70160 | -2.83758 | -0.00135 |
| H | 2.43760 | -4.54244 | 0.33847 |
| H | 4.69907 | -0.96402 | -0.35196 |
| H | 4.61807 | -3.41528 | -0.00298 |

**1p**

Img. Freq. = 0
E = -628.588251 Hartrees
HOMO = -0.29101 Hartrees
LUMO = -0.06525 Hartrees

| | | | |
|---|---|---|---|
| C | 0.05104 | -0.56167 | 0.07350 |
| H | -0.86793 | -1.11775 | 0.23632 |
| C | -0.07569 | 0.74775 | -0.13650 |
| H | 0.69996 | 1.47541 | -0.31619 |
| O | -1.42624 | 2.55813 | -0.33931 |
| O | -2.37562 | 0.66383 | 0.04971 |
| N | -1.39808 | 1.35916 | -0.14016 |
| C | 1.29818 | -1.31537 | 0.10625 |
| C | 1.24696 | -2.68730 | 0.35144 |
| C | 2.55878 | -0.72602 | -0.09813 |
| C | 2.39849 | -3.46626 | 0.39732 |
| H | 0.28427 | -3.16073 | 0.51105 |
| C | 3.70800 | -1.48217 | -0.05641 |
| H | 2.63859 | 0.33693 | -0.29271 |
| C | 3.63757 | -2.86135 | 0.19238 |
| H | 2.31596 | -4.52670 | 0.59048 |
| H | 4.68355 | -1.03926 | -0.21269 |
| O | 4.81880 | -3.51291 | 0.21321 |
| C | 4.80595 | -4.90634 | 0.46064 |
| H | 5.84497 | -5.22464 | 0.43463 |
| H | 4.38066 | -5.12581 | 1.44419 |
| H | 4.24162 | -5.43647 | -0.31197 |

**1q**

Img. Freq. = 0
E = -591.462426 Hartrees
HOMO = -0.29289 Hartrees
LUMO = -0.07779 Hartrees

| | | | |
|---|---|---|---|
| C | 0.06112 | -0.64880 | -0.03965 |
| H | -0.81851 | -1.26103 | -0.21636 |
| C | -0.10916 | 0.66760 | 0.09345 |
| H | 0.65521 | 1.40907 | 0.27124 |
| O | -1.49887 | 2.45918 | 0.13631 |
| O | -2.39126 | 0.52566 | -0.19649 |
| N | -1.43876 | 1.25260 | 0.00254 |
| C | 1.35196 | -1.29386 | 0.03915 |
| H | 2.21568 | -0.66085 | 0.21595 |
| C | 1.48125 | -2.62368 | -0.10137 |
| H | 0.57681 | -3.20249 | -0.27788 |
| C | 2.71923 | -3.40326 | -0.04505 |
| C | 2.64594 | -4.78904 | -0.22625 |
| C | 3.97524 | -2.82397 | 0.18275 |
| C | 3.78948 | -5.57697 | -0.18306 |
| H | 1.67952 | -5.24909 | -0.40287 |

**1r**

Img. Freq. = 0
E = -1010.240372 Hartrees
HOMO = -0.34394 Hartrees
LUMO = -0.09440 Hartrees

| | | | |
|---|---|---|---|
| C | 0.08930 | -0.57074 | 0.05761 |
| H | -0.80897 | -1.16356 | 0.19591 |
| C | -0.07118 | 0.74354 | -0.06265 |
| H | 0.66823 | 1.51379 | -0.20446 |
| O | -1.50776 | 2.49933 | -0.11382 |
| O | -2.36722 | 0.54129 | 0.15459 |
| N | -1.43098 | 1.29455 | -0.00071 |
| C | 1.34895 | -1.30744 | 0.02449 |
| C | 1.32005 | -2.69674 | 0.17617 |
| C | 2.60663 | -0.72645 | -0.15021 |
| C | 2.46843 | -3.46836 | 0.15667 |
| C | 3.76815 | -1.47632 | -0.17331 |
| C | 3.69870 | -2.85366 | -0.01907 |
| F | 0.15784 | -3.31662 | 0.34658 |
| F | 2.40063 | -4.78201 | 0.30400 |
| F | 4.80290 | -3.57623 | -0.03973 |

| | | | | | | | |
|---|---|---|---|---|---|---|---|
| C | 5.11623 | -3.60956 | 0.22608 | F | 4.94317 | -0.89000 | -0.34116 |
| H | 4.06408 | -1.75414 | 0.32795 | F | 2.71936 | 0.58935 | -0.30186 |
| C | 5.02831 | -4.98886 | 0.04327 | | | | |
| H | 3.71260 | -6.64801 | -0.32585 | | | | |
| H | 6.08005 | -3.14761 | 0.40320 | | | | |
| H | 5.92273 | -5.59909 | 0.07792 | | | | |

**1s**
Img. Freq. = 0
E = -530.109846 Hartrees
HOMO = -0.34597 Hartrees
LUMO = -0.08648 Hartrees

| | | | |
|---|---|---|---|
| C | 0.04583 | -0.60778 | -0.03048 |
| H | -0.87028 | -1.18961 | -0.07090 |
| C | -0.07051 | 0.71271 | 0.03656 |
| H | 0.71464 | 1.44987 | 0.10822 |
| O | -1.41018 | 2.54462 | 0.06620 |
| O | -2.37794 | 0.62118 | -0.02604 |
| N | -1.39757 | 1.33202 | 0.02419 |
| C | 1.31795 | -1.33967 | -0.02709 |
| C | 1.31087 | -2.71424 | 0.20681 |
| C | 2.55434 | -0.72825 | -0.24862 |
| C | 2.51999 | -3.40193 | 0.22602 |
| H | 0.37946 | -3.24170 | 0.37599 |
| C | 3.70164 | -1.50793 | -0.21039 |
| H | 2.63217 | 0.32998 | -0.46513 |
| H | 2.53671 | -4.47120 | 0.41076 |
| H | 4.67299 | -1.05564 | -0.38214 |
| N | 3.69989 | -2.82141 | 0.02414 |

**1t**
Img. Freq. = 0
E = -743.093188 Hartrees
HOMO = -0.28427 Hartrees
LUMO = -0.06773 Hartrees

| | | | |
|---|---|---|---|
| C | 0.06056 | -0.53322 | -0.09290 |
| H | -0.87519 | -1.08241 | -0.14665 |
| C | -0.02093 | 0.78968 | -0.22090 |
| H | 0.77666 | 1.51539 | -0.19591 |
| O | -1.30645 | 2.63204 | -0.53164 |
| O | -2.31598 | 0.72993 | -0.47219 |
| N | -1.31875 | 1.42156 | -0.42326 |
| C | 1.28034 | -1.30851 | 0.11270 |
| C | 1.15761 | -2.69892 | 0.24860 |
| C | 2.54884 | -0.73056 | 0.18441 |
| C | 2.27172 | -3.50334 | 0.45278 |
| H | 0.16936 | -3.13870 | 0.21046 |
| C | 3.66446 | -1.53083 | 0.36865 |
| H | 2.67505 | 0.34032 | 0.08666 |
| C | 3.54931 | -2.91144 | 0.49315 |
| H | 4.65968 | -1.10700 | 0.41936 |
| O | 4.68299 | -3.61852 | 0.71112 |
| C | 4.90375 | -4.80443 | -0.05380 |
| H | 5.98431 | -4.90500 | -0.14099 |
| H | 4.48292 | -5.67444 | 0.44677 |
| H | 4.46343 | -4.70648 | -1.04898 |
| O | 2.22289 | -4.84536 | 0.64512 |
| C | 0.95182 | -5.46668 | 0.64488 |
| H | 1.13500 | -6.52308 | 0.82538 |
| H | 0.31919 | -5.06497 | 1.44171 |
| H | 0.45246 | -5.34282 | -0.32060 |

**1u**
Img. Freq. = 0
E = -590.210203 Hartrees
HOMO = -0.31170 Hartrees
LUMO = -0.07550 Hartrees

| | | | |
|---|---|---|---|
| C | 0.06699 | -0.55733 | -0.00528 |
| H | -0.80967 | -1.19916 | -0.00210 |
| C | -0.17884 | 0.74939 | 0.03671 |
| H | 0.51496 | 1.57118 | 0.05933 |
| O | -1.72230 | 2.41220 | 0.11659 |
| O | -2.46962 | 0.39410 | 0.04544 |
| N | -1.57031 | 1.20836 | 0.06840 |

**1v**
Img. Freq. = 0
E = -923.034987 Hartrees
HOMO = -0.37067 Hartrees
LUMO = -0.07550 Hartrees

| | | | |
|---|---|---|---|
| C | 0.01438 | -0.61210 | -0.22149 |
| H | -0.86505 | -1.05169 | -0.67799 |
| C | -0.05422 | 0.63843 | 0.21090 |
| H | 0.70335 | 1.19887 | 0.73898 |
| O | -1.27320 | 2.53276 | 0.48366 |
| O | -2.21299 | 0.89780 | -0.56249 |
| N | -1.28760 | 1.41054 | 0.02456 |

| | | | |
|---|---|---|---|
| C | 1.35439 | -1.25500 | -0.02114 |
| C | 1.30667 | -2.64105 | 0.17725 |
| C | 2.61605 | -0.65237 | -0.22186 |
| C | 2.45554 | -3.41648 | 0.19777 |
| H | 0.34013 | -3.11018 | 0.32237 |
| C | 3.76931 | -1.44501 | -0.20429 |
| C | 3.69433 | -2.81370 | 0.00704 |
| H | 2.38407 | -4.48501 | 0.35841 |
| H | 4.72674 | -0.96482 | -0.36286 |
| H | 4.60032 | -3.40710 | 0.01740 |
| C | 2.78948 | 0.74794 | -0.46674 |
| C | 3.00736 | 1.91024 | -0.68231 |
| H | 3.18438 | 2.94269 | -0.87301 |

**1w**
Img. Freq. = 0
E = -592.676536 Hartrees
HOMO = -0.31075 Hartrees
LUMO = -0.05599 Hartrees

| | | | |
|---|---|---|---|
| C | -0.30008 | -1.34050 | -0.00154 |
| C | 1.02248 | -0.91026 | 0.09540 |
| C | 1.32730 | 0.44821 | 0.14731 |
| C | 0.27457 | 1.36743 | 0.10156 |
| C | -1.04298 | 0.94320 | 0.00487 |
| C | -1.33584 | -0.41782 | -0.04738 |
| H | -0.51587 | -2.40177 | -0.04038 |
| H | 1.81158 | -1.65207 | 0.13047 |
| H | 0.49692 | 2.42929 | 0.14301 |
| H | -1.84315 | 1.67317 | -0.02917 |
| H | -2.36331 | -0.75273 | -0.12239 |
| C | 2.74341 | 0.97552 | 0.24937 |
| H | 2.94728 | 1.60555 | -0.62332 |
| H | 2.81240 | 1.63375 | 1.12139 |
| C | 3.83279 | -0.09717 | 0.35361 |
| H | 3.79522 | -0.73975 | -0.53288 |
| H | 3.66333 | -0.73057 | 1.22794 |
| C | 5.18731 | 0.52969 | 0.42240 |
| H | 5.50755 | 1.16323 | -0.40097 |
| C | 6.01750 | 0.37338 | 1.44173 |
| H | 5.85850 | -0.21540 | 2.33332 |
| N | 7.32742 | 1.03127 | 1.44445 |
| O | 7.63459 | 1.73554 | 0.50670 |
| O | 8.02433 | 0.81414 | 2.41417 |

**1y**
Img. Freq. = 0
E = -530.109846 Hartrees
HOMO = -0.34597 Hartrees
LUMO = -0.08648 Hartrees

| | | | |
|---|---|---|---|
| C | 1.25694 | -1.39807 | -0.11010 |
| C | 1.28905 | -2.76033 | 0.21347 |
| C | 2.48306 | -0.78508 | -0.39560 |
| C | 2.46000 | -3.49475 | 0.26479 |
| C | 3.67812 | -1.48529 | -0.33450 |
| H | 2.48811 | 0.25452 | -0.69790 |
| C | 3.64086 | -2.83038 | -0.00637 |
| H | 2.44797 | -4.54739 | 0.51351 |
| H | 4.62635 | -1.01140 | -0.55024 |
| N | 0.04492 | -3.48523 | 0.55886 |
| N | 4.91087 | -3.59093 | 0.05489 |
| O | -0.88871 | -2.82429 | 0.95732 |
| O | 0.05391 | -4.68624 | 0.43117 |
| O | 4.83975 | -4.76108 | 0.34827 |
| O | 5.92687 | -2.98357 | -0.19377 |

**1x**
Img. Freq. = 0
E = -553.382012 Hartrees
HOMO = -0.30999 Hartrees
LUMO = -0.06147 Hartrees

| | | | |
|---|---|---|---|
| C | 0.12028 | -0.55889 | -0.23522 |
| H | -0.77987 | -1.12446 | -0.45349 |
| C | -0.02355 | 0.75550 | -0.05946 |
| O | -1.50383 | 2.50306 | -0.26496 |
| O | -2.31280 | 0.52400 | -0.46395 |
| N | -1.39528 | 1.29436 | -0.27855 |
| C | 1.37405 | -1.31880 | -0.13281 |
| C | 1.33382 | -2.60519 | 0.41703 |
| C | 2.59537 | -0.82289 | -0.60197 |
| C | 2.49335 | -3.35873 | 0.53922 |
| H | 0.38615 | -3.00666 | 0.75904 |
| C | 3.75222 | -1.58461 | -0.49367 |
| H | 2.63182 | 0.14684 | -1.08370 |
| C | 3.70650 | -2.84836 | 0.08654 |
| H | 2.44967 | -4.34749 | 0.97958 |
| H | 4.68976 | -1.19387 | -0.87054 |
| H | 4.61053 | -3.43906 | 0.17277 |
| C | 0.97834 | 1.78325 | 0.34927 |
| H | 1.85486 | 1.29021 | 0.76604 |
| H | 1.28241 | 2.40690 | -0.49448 |
| H | 0.54687 | 2.44519 | 1.09977 |

**1z**
Img. Freq. = 0
E = -757.869442 Hartrees
HOMO = -0.33784 Hartrees
LUMO = -0.09083 Hartrees

| | | | | | | | |
|---|---|---|---|---|---|---|---|
| C | 0.06727 | -0.48968 | -0.10370 | C | 0.14405 | -0.56228 | -0.22380 |
| H | -0.77442 | -0.97704 | -0.58371 | H | -0.75214 | -1.11379 | -0.48894 |
| C | -0.16027 | 0.66890 | 0.50756 | C | 0.00245 | 0.73633 | 0.03865 |
| O | -1.72589 | 2.32708 | 0.79546 | O | -1.47307 | 2.49596 | -0.08696 |
| O | -2.41002 | 0.49282 | -0.08904 | O | -2.28664 | 0.52941 | -0.38006 |
| N | -1.55029 | 1.19839 | 0.39184 | N | -1.37106 | 1.29016 | -0.15723 |
| C | 1.34299 | -1.22918 | -0.10841 | C | 1.40107 | -1.32300 | -0.15377 |
| C | 1.29763 | -2.59081 | 0.21249 | C | 1.36686 | -2.62880 | 0.35016 |
| C | 2.60737 | -0.69341 | -0.36686 | C | 2.61511 | -0.79949 | -0.61306 |
| C | 2.45181 | -3.35674 | 0.29852 | C | 2.52451 | -3.38569 | 0.43957 |
| H | 0.33236 | -3.04035 | 0.41506 | H | 0.42476 | -3.04735 | 0.68447 |
| C | 3.77495 | -1.43553 | -0.27013 | C | 3.78004 | -1.54897 | -0.54213 |
| C | 3.69568 | -2.77946 | 0.06350 | H | 2.64267 | 0.18787 | -1.05669 |
| H | 2.37920 | -4.40698 | 0.55282 | C | 3.71163 | -2.82529 | -0.00650 |
| H | 4.72232 | -0.95230 | -0.47005 | H | 2.52086 | -4.39084 | 0.83851 |
| H | 4.59896 | -3.37233 | 0.12964 | H | 4.72680 | -1.16668 | -0.89866 |
| O | 3.73031 | 1.31045 | -0.41192 | O | 5.97515 | -3.11463 | -0.31865 |
| O | 1.93582 | 1.14307 | -1.58662 | O | 4.86374 | -4.74290 | 0.53995 |
| N | 2.76440 | 0.70382 | -0.82296 | N | 4.95216 | -3.62718 | 0.07798 |
| C | 0.77354 | 1.54413 | 1.26887 | C | 0.99938 | 1.73596 | 0.51993 |
| H | 1.67157 | 0.98709 | 1.53750 | H | 1.88281 | 1.22289 | 0.89572 |
| H | 1.05979 | 2.41451 | 0.67272 | H | 1.29119 | 2.42385 | -0.27673 |
| H | 0.29759 | 1.90711 | 2.17909 | H | 0.56601 | 2.33634 | 1.31968 |

**1za**
Img. Freq. = 0
E = -757.864917 Hartrees
HOMO = -0.34312 Hartrees
LUMO = -0.09002 Hartrees

| | | | |
|---|---|---|---|
| C | -0.03573 | -0.72263 | -0.07265 |
| C | -0.02123 | 0.56215 | 0.29740 |
| H | 0.86277 | 1.10631 | 0.59433 |
| O | -0.97949 | 2.61032 | 0.40064 |
| O | -2.30201 | 0.91361 | 0.27689 |
| N | -1.19778 | 1.41857 | 0.32338 |
| C | 1.27234 | -1.43038 | -0.05440 |
| C | 1.34316 | -2.73437 | 0.44875 |
| C | 2.43538 | -0.81575 | -0.52877 |
| C | 2.55453 | -3.40582 | 0.50615 |
| H | 0.44761 | -3.22081 | 0.81643 |
| C | 3.65313 | -1.47942 | -0.48902 |
| H | 2.38236 | 0.17733 | -0.95802 |
| C | 3.68766 | -2.76172 | 0.03387 |
| H | 2.63309 | -4.40772 | 0.90558 |
| H | 4.56179 | -1.02574 | -0.86067 |
| O | 5.95494 | -2.89493 | -0.34731 |
| O | 4.98505 | -4.59493 | 0.54341 |
| N | 4.98269 | -3.47626 | 0.08001 |
| C | -1.24811 | -1.51685 | -0.48012 |
| H | -1.86656 | -1.74521 | 0.39115 |
| H | -1.87810 | -0.94559 | -1.15981 |
| H | -0.94278 | -2.44487 | -0.96069 |
